# Supplementary material for: Proteomics of extracellular vesicles produced by Granulicatella adiacens, which causes infective endocarditis
Source: PLoS One. 2020 Nov 20;15(11):e0227657. doi: 10.1371/journal.pone.0227657 (PMC7679012; doi:10.1371/journal.pone.0227657)
Supplement: S3 File — (PDF) [file pone.0227657.s003.pdf]

MATRIX SCIENCE MASCOT Search Results

User : Ulf Bergmann
E-mail : ub@pfa
Search title : LTQ_19B022_Kuweit_Sample-GA-EVS
MS data file : LTQ_19B022_Kuweit_Sample-GA-EVS.mgf
Databases : **1:** contaminants 20160129 (249 sequences; 128,730 residues)
2: UB_target (17,006 sequences; 5,494,838 residues)
Timestamp : 5 May 2020 at 11:59:17 GMT

Re-search ◉ All ○ Non-significant ○ Unassigned [help] Export As XML

Not what you expected? Try the select summary.

▸ **Search parameters**

▸ **Score distribution**

▸ **Modification statistics**

▸ **Legend**

Protein Family Summary

Format | Significance threshold p< 0.0035 | Max. number of families AUTO | [help]
Display non-sig. matches ☐ | Dendrograms cut at 0
Show Percolator scores ☐
Preferred taxonomy: All entries

▸ **Sensitivity and FDR (reversed protein sequences)**

Proteins (124) | Report Builder | Unassigned (22579) | § permalink

Protein hits (112 proteins)

▸ **Columns: Standard (11 out of 16)**

▸ **Filters: (NOT(Database is contaminants) AND Member = 1)**

Export as CSV

↑Family	M	DB	Accession	Score	emPAI	Mass	Cov	Match(sig)	Seq(uniq+sig)	Description
1	1	UB_target	2::WP_005607266.1	1892	1.10	56817	0.46	45	14	chaperonin GroEL [Granulicatella adiacens]
2	1	UB_target	2::WP_005607328.1	1856	1.36	66598	0.50	54	13	oligopeptide ABC transporter substrate-binding protein [Granulicatella adiacens]
3	1	UB_target	2::WP_005607135.1	1322	1.67	27143	0.53	30	9	triose-phosphate isomerase [Granulicatella]
4	1	UB_target	2::WP_005606562.1	1192	0.93	45775	0.43	34	10	CHAP domain-containing protein [Granulicatella adiacens]
5	1	UB_target	2::WP_005607132.1	1128	1.29	46972	0.52	30	12	phosphopyruvate hydratase [Granulicatella adiacens]
6	1	UB_target	2::WP_005607139.1	988	2.21	35885	0.67	31	11	type I glyceraldehyde-3-phosphate dehydrogenase [Granulicatella]
7	1	UB_target	2::RKW27198.1	682	0.44	49846	0.26	18	6	NADH oxidase [Granulicatella sp.]
8	1	UB_target	2::WP_005606196.1	595	1.34	17251	0.37	16	4	DNA starvation/stationary phase protection protein [Granulicatella adiacens]
9	1	UB_target	2::WP_005606451.1	540	0.75	37403	0.34	17	7	tagatose-bisphosphate aldolase [Granulicatella adiacens]
10	1	UB_target	2::WP_005605418.1	517	0.62	36960	0.30	11	6	lactate dehydrogenase [Granulicatella]
11	1	UB_target	2::WP_005607062.1	508	0.38	66175	0.20	15	7	M3 family oligoendopeptidase [Granulicatella adiacens]
12	1	UB_target	2::RKW27215.1	503	0.48	61821	0.21	17	8	phospho-sugar mutase [Granulicatella sp.]
13	1	UB_target	2::WP_005606663.1	498	0.87	48005	0.37	18	10	extracellular solute-binding protein [Granulicatella]
14	1	UB_target	2::WP_005606180.1	446	0.55	47778	0.20	15	6	extracellular solute-binding protein [Granulicatella adiacens]

16	1	UB_target	2::WP_005605501.1	426	0.75	32000	0.36	17	6	elongation factor Ts [Granulicatella adiacens]
17	1	UB_target	2::WP_005605525.1	413	0.32	65434	0.18	14	6	molecular chaperone DnaK [Granulicatella adiacens]
18	1	UB_target	2::WP_005606841.1	396	0.62	49779	0.33	19	8	glucose-6-phosphate isomerase [Granulicatella adiacens]
19	1	UB_target	2::WP_005606823.1	388	0.40	62778	0.16	11	7	phosphoenolpyruvate--protein phosphotransferase [Granulicatella adiacens]
20	1	UB_target	2::WP_005607047.1	377	0.61	50367	0.29	16	8	C1 family peptidase [Granulicatella adiacens]
21	1	UB_target	2::WP_005606636.1	346	0.35	39940	0.15	11	4	glutamyl aminopeptidase [Granulicatella adiacens]
22	1	UB_target	2::WP_005607201.1	301	0.44	49088	0.20	10	6	carbohydrate ABC transporter substrate-binding protein [Granulicatella]
23	1	UB_target	2::WP_005607487.1	286	0.74	43376	0.33	12	8	elongation factor Tu [Granulicatella]
24	1	UB_target	2::WP_005605278.1	274	1.62	8810	0.41	6	3	acyl carrier protein [Granulicatella adiacens]
25	1	UB_target	2::EEW36863.1	269	0.18	54184	0.12	7	3	peptidase, C69 family [Granulicatella adiacens ATCC 49175 ATCC 49175]
26	1	UB_target	2::WP_005607854.1	264	0.18	55053	0.11	8	3	citrate lyase subunit alpha [Granulicatella adiacens]
27	1	UB_target	2::RKW29380.1	262	0.14	68179	0.09	6	3	oligoendopeptidase F [Granulicatella sp.]
28	1	UB_target	2::RKW26694.1	258	0.17	38311	0.10	7	2	M42 family peptidase [Granulicatella sp.]
29	1	UB_target	2::WP_005605012.1	237	0.14	68834	0.08	6	3	V-type ATP synthase subunit A [Granulicatella adiacens]
30	1	UB_target	2::WP_005605729.1	231	0.19	34592	0.11	3	2	6-phosphofructokinase [Granulicatella]
31	1	UB_target	2::WP_005605283.1	231	0.32	43140	0.22	9	3	beta-ketoacyl-ACP synthase II [Granulicatella adiacens]
32	1	UB_target	2::WP_005605130.1	230	0.18	72728	0.11	7	4	transketolase [Granulicatella adiacens]
33	1	UB_target	2::RKW26844.1	224	0.26	51702	0.16	7	4	pyruvate kinase [Granulicatella sp.]
34	1	UB_target	2::RKW29833.1	221	0.25	53480	0.14	6	4	flavocytochrome c [Granulicatella sp.]
35	1	UB_target	2::WP_005605703.1	217	0.76	10047	0.33	7	2	HU family DNA-binding protein [Lactobacillales]
36	1	UB_target	2::WP_005605626.1	212	0.36	19293	0.19	3	2	adenine phosphoribosyltransferase [Granulicatella adiacens]
37	1	UB_target	2::RKW29377.1	202	0.30	45692	0.17	7	4	toxic anion resistance protein [Granulicatella sp.]
38	1	UB_target	2::WP_005605225.1	192	0.28	49313	0.15	6	4	dihydrolipoyl dehydrogenase [Granulicatella adiacens]
39	1	UB_target	2::WP_005607427.1	192	0.30	45504	0.14	10	4	aminopeptidase [Granulicatella adiacens]
40	1	UB_target	2::WP_005606850.1	191	0.17	18408	0.11	3	1	phosphatidylglycerophosphatase A [Granulicatella adiacens]
41	1	UB_target	2::WP_005607816.1	191	0.47	15065	0.23	6	2	50S ribosomal protein L11 [Lactobacillales]
42	1	UB_target	2::EEW36428.1	186	0.20	50035	0.11	7	3	hypothetical protein [Granulicatella adiacens ATCC 49175 ATCC 49175]
43	1	UB_target	2::EEW37841.1	185	0.15	21088	0.09	2	1	co-chaperone GrpE [Granulicatella adiacens ATCC 49175 ATCC 49175]
44	1	UB_target	2::WP_005606105.1	183	0.40	26612	0.11	8	3	SDR family oxidoreductase [Granulicatella]
45	1	UB_target	2::EEW37245.1	172	0.22	45195	0.10	8	3	phosphoglycerate kinase [Granulicatella adiacens ATCC 49175 ATCC 49175]
47	1	UB_target	2::WP_005605924.1	167	0.09	73198	0.05	5	2	S9 family peptidase [Granulicatella adiacens]
48	1	UB_target	2::WP_005607398.1	165	0.29	35309	0.17	4	3	metal ABC transporter substrate-binding protein [Granulicatella adiacens]
49	1	UB_target	2::WP_005606772.1	161	0.11	88259	0.06	5	3	phenylalanine--tRNA ligase subunit beta [Granulicatella adiacens]
50	1	UB_target	2::WP_005607810.1	157	0.95	17495	0.34	6	4	50S ribosomal protein L10 [Granulicatella]
51	1	UB_target	2::RKW29980.1	157	0.87	9025	0.21	4	2	30S ribosomal protein S20 [Granulicatella sp.]
52	1	UB_target	2::WP_005606514.1	148	0.11	59098	0.04	4	2	peptide ABC transporter substrate-binding protein [Granulicatella adiacens]
53	1	UB_target	2::EEW38181.1	145	0.08	39481	0.05	2	1	alcohol dehydrogenase, iron-dependent [Granulicatella adiacens ATCC 49175 ATCC 49175]
54	1	UB_target	2::RKW25768.1	142	0.18	35450	0.13	5	2	alpha-ketoacid dehydrogenase subunit beta [Granulicatella sp.]
55	1	UB_target	2::RKW28577.1	139	0.29	11214	0.17	3	1	50S ribosomal protein L21 [Granulicatella sp.]
56	1	UB_target	2::WP_005605696.1	138	0.38	18014	0.27	6	2	hypothetical protein [Granulicatella]
57	1	UB_target	2::WP_005605745.1	134	0.18	35619	0.14	4	2	phosphate acetyltransferase [Granulicatella adiacens]
58	1	UB_target	2::RKW29614.1	132	0.26	26015	0.14	3	2	2-C-methyl-D-erythritol 4-phosphate cytidylyltransferase [Granulicatella sp.]
59	1	UB_target	2::WP_005605221.1	131	0.25	40763	0.13	5	3	pyruvate dehydrogenase (acetyl-transferring) E1 component subunit alpha [Granulicatella]
61	1	UB_target	2::WP_005607807.1	127	0.99	12547	0.35	5	3	50S ribosomal protein L7/L12 [Granulicatella]
62	1	UB_target	2::RKW27282.1	124	0.25	53085	0.13	6	4	NADP-dependent phosphogluconate dehydrogenase [Granulicatella sp.]
63	1	UB_target	2::EEW37447.1	121	0.27	12113	0.12	3	1	thioredoxin [Granulicatella adiacens ATCC 49175 ATCC 49175]
64	1	UB_target	2::RKW29913.1	120	0.05	62818	0.02	4	1	flavocytochrome c [Granulicatella sp.]
65	1	UB_target	2::WP_005605411.1	119	0.07	42264	0.04	2	1	Nif3-like dinuclear metal center hexameric protein [Granulicatella adiacens]
66	1	UB_target	2::RKW28818.1	115	0.17	38684	0.10	3	2	phosphonate ABC transporter substrate-binding protein [Granulicatella sp.]
67	1	UB_target	2::WP_005606374.1	112	0.08	36829	0.05	2	1	hypothetical protein [Granulicatella]
68	1	UB_target	2::WP_005605890.1	109	0.16	59900	0.13	5	3	NAD-dependent malic enzyme [Granulicatella]
69	1	UB_target	2::WP_005605842.1	107	0.06	49485	0.04	2	1	hypothetical protein [Granulicatella adiacens]
70	1	UB_target	2::RKW29369.1	104	0.12	27154	0.09	1	1	hypothetical protein [Granulicatella sp.]

71	1	UB_target	2::EEW36930.1	103	0.10	30173	0.13	4	1	3D domain protein [Granulicatella adiacens ATCC 49175 ATCC 49175]
72	1	UB_target	2::RKW29737.1	97	0.03	91688	0.02	2	1	PBP1A family penicillin-binding protein [Granulicatella sp.]
74	1	UB_target	2::RKW25989.1	92	0.09	34574	0.03	4	1	foldase [Granulicatella sp.]
75	1	UB_target	2::RKW26849.1	92	0.10	29847	0.07	2	1	purine-nucleoside phosphorylase [Granulicatella sp.]
76	1	UB_target	2::RKW26696.1	90	0.15	20471	0.06	2	1	DUF1307 domain-containing protein [Granulicatella sp.]
77	1	UB_target	2::EEW37494.1	88	0.28	11562	0.09	2	1	phosphocarrier protein HPr [Granulicatella adiacens ATCC 49175 ATCC 49175]
78	1	UB_target	2::OFT00695.1	77	0.09	33577	0.04	3	1	L-ribulose-5-phosphate 4-epimerase [Granulicatella sp. HMSC31F03]
79	1	UB_target	2::WP_005608182.1	77	0.29	11336	0.25	3	1	30S ribosomal protein S6 [Granulicatella]
80	1	UB_target	2::RKW29337.1	76	0.13	23910	0.06	2	1	biotin/lipoyl-binding protein, partial [Granulicatella sp.]
81	1	UB_target	2::RKW27683.1	75	0.10	32054	0.05	3	1	ROK family protein [Granulicatella sp.]
82	1	UB_target	2::QDC26953.1	74	0.22	14729	0.15	1	1	translation superoxide dismutase, partial [Granulicatella adiacens]
83	1	UB_target	2::WP_005605710.1	74	0.15	20808	0.09	3	1	LysM peptidoglycan-binding domain-containing protein [Granulicatella adiacens]
84	1	UB_target	2::RKW26695.1	73	0.19	16653	0.09	2	1	DUF1307 domain-containing protein [Granulicatella sp.]
85	1	UB_target	2::RKW29417.1	73	0.39	8479	0.29	1	1	thiol reductase thioredoxin, partial [Granulicatella sp.]
86	1	UB_target	2::WP_005605248.1	72	0.16	39499	0.09	2	2	extracellular solute-binding protein [Granulicatella adiacens]
87	1	UB_target	2::WP_005604985.1	72	0.24	13326	0.11	2	1	YlbF family regulator [Granulicatella]
89	1	UB_target	2::EEW37660.1	70	0.16	39287	0.09	2	2	putative dihydrodipicolinate reductase domain protein [Granulicatella adiacens ATCC 49175 ATCC 49175]
90	1	UB_target	2::WP_005605180.1	70	0.19	68351	0.12	4	4	oligoendopeptidase F [Granulicatella adiacens]
91	1	UB_target	2::EEW37316.1	65	0.39	8506	0.23	1	1	cold-shock DNA-binding domain protein [Granulicatella adiacens ATCC 49175 ATCC 49175]
92	1	UB_target	2::WP_005605412.1	64	0.14	45833	0.06	2	2	peptidase T [Granulicatella]
94	1	UB_target	2::WP_005607452.1	63	0.18	17422	0.14	2	1	30S ribosomal protein S5 [Granulicatella]
95	1	UB_target	2::RKW29368.1	60	0.12	27190	0.06	1	1	hypothetical protein [Granulicatella sp.]
96	1	UB_target	2::WP_005604991.1	60	0.18	36183	0.07	2	2	HD domain-containing protein [Granulicatella adiacens]
97	1	UB_target	2::WP_005605054.1	58	0.09	34495	0.07	1	1	ROK family glucokinase [Granulicatella]
99	1	UB_target	2::EEW38033.1	55	0.09	32826	0.09	1	1	putative enoyl-[acyl-carrier-protein] reductase II [Granulicatella adiacens ATCC 49175 ATCC 49175]
100	1	UB_target	2::WP_005607387.1	54	0.05	57937	0.04	2	1	zinc ABC transporter substrate-binding protein AdcA [Granulicatella adiacens]
101	1	UB_target	2::RKW25673.1	54	0.34	9792	0.16	2	1	hypothetical protein [Granulicatella sp.]
103	1	UB_target	2::RKW28361.1	53	0.09	34421	0.06	1	1	DUF1002 domain-containing protein [Granulicatella sp.]
104	1	UB_target	2::EEW38042.1	51	0.24	42334	0.15	3	3	Creatinase [Granulicatella adiacens ATCC 49175 ATCC 49175]
105	1	UB_target	2::RKW27369.1	48	0.11	27936	0.04	2	1	oxaloacetate decarboxylase subunit alpha, partial [Granulicatella sp.]
106	1	UB_target	2::RKW28418.1	43	0.09	67246	0.06	2	2	oligopeptide ABC transporter substrate-binding protein [Granulicatella sp.]
107	1	UB_target	2::WP_005607857.1	40	0.20	32299	0.09	2	2	citrate (pro-3S)-lyase subunit beta [Granulicatella adiacens]
108	1	UB_target	2::WP_005607469.1	40	0.42	7960	0.19	1	1	50S ribosomal protein L29 [Lactobacillales]
109	1	UB_target	2::RKW27467.1	39	0.10	60907	0.05	2	2	ATP-binding cassette domain-containing protein [Granulicatella sp.]
110	1	UB_target	2::EEW36998.1	39	0.11	28444	0.07	1	1	ribosomal protein L2 [Granulicatella adiacens ATCC 49175 ATCC 49175]
112	1	UB_target	2::RKW29296.1	36	0.16	19355	0.12	1	1	FMN-binding protein [Granulicatella sp.]
113	1	UB_target	2::RKW25792.1	35	0.07	42369	0.04	1	1	acetate kinase, partial [Granulicatella sp.]
115	1	UB_target	2::WP_005607235.1	34	0.17	18963	0.12	1	1	Lrp/AsnC family transcriptional regulator [Granulicatella adiacens]
117	1	UB_target	2::EEW36408.1	34	0.31	10436	0.19	1	1	hypothetical protein [Granulicatella adiacens ATCC 49175 ATCC 49175]
118	1	UB_target	2::RKW25990.1	33	0.12	27189	0.06	2	1	methionine ABC transporter substrate-binding protein, partial [Granulicatella sp.]
119	1	UB_target	2::WP_005605154.1	32	0.06	48647	0.03	1	1	glutathione-disulfide reductase [Granulicatella adiacens]
120	1	UB_target	2::WP_005607237.1	30	0.09	35756	0.04	1	1	phosphate acyltransferase PlsX [Granulicatella adiacens]
121	1	UB_target	2::RKW29474.1	30	0.06	55089	0.03	1	1	F0F1 ATP synthase subunit alpha [Granulicatella sp.]
122	1	UB_target	2::WP_005607463.1	28	0.30	10851	0.10	1	1	50S ribosomal protein L24 [Granulicatella adiacens]
124	1	UB_target	2::WP_005607461.1	26	0.16	20147	0.11	1	1	50S ribosomal protein L5 [Granulicatella adiacens]

Export as CSV

Not what you expected? Try the select summary.

Mascot: http://www.matrixscience.com/

MASCOT Search Results

Protein View: WP_005606850.1

phosphatidylglycerophosphatase A [Granulicatella adiacens]

**Database:**	UB_target
**Score:**	191
**Monoisotopic mass (M_r):**	18408
**Calculated pI:**	4.88

Sequence similarity is available as an NCBI BLAST search of WP_005606850.1 against nr.

Search parameters

**MS data file:**	LTQ_19B022_Kuweit_Sample-GA-EVS.mgf
**Enzyme:**	Trypsin: cuts C-term side of KR unless next residue is P.
**Fixed modifications:**	Carbamidomethyl (C)
**Variable modifications:**	Deamidated (NQ), Oxidation (M)

Protein sequence coverage: 11%

Matched peptides shown in ***bold red***.

```
  1 MKSPKELHER SLELLSERGV DVDQIADLVY FLQEKYVPGL TKEECLSNVH
 51 SVLKKREVQN AVITGIEMDK LAEEKKLHPL LNDILSEDEP LYGVDEILAL
101 SIVNVYGSIG FTNYGYIDKV KPGILKNLDD KSTGKVNIFL DDLVGAIAAA
151 AASRLAHSIP AKEELD
```

Unformatted sequence string: 166 residues (for pasting into other applications).

Sort by ◉ residue number ○ increasing mass ○ decreasing mass
Show matched peptides only predicted peptides also

Query	Start – End	Observed	Mr(expt)	Mr(calc)	ppm	M	Score	Expect	Rank	U	Peptide
12947	136 – 154	629.6824	1886.0254	1886.0261	-0.42	0	49	4.3e-005	1	U	K.VNIFLDDLVGAIAAAAASR.L
12948	136 – 154	629.6826	1886.0260	1886.0261	-0.090	0	66	7.9e-007	1	U	K.VNIFLDDLVGAIAAAAASR.L
12949	136 – 154	944.0218	1886.0291	1886.0261	1.58	0	133	1.4e-013	1	U	K.VNIFLDDLVGAIAAAAASR.L

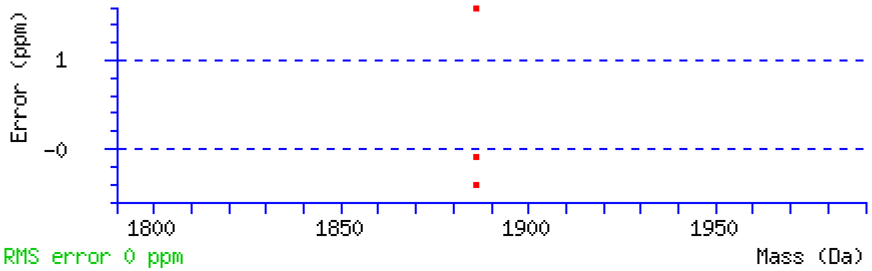

Mascot: http://www.matrixscience.com/

MATRIX SCIENCE Mascot Search Results

Peptide View

MS/MS Fragmentation of **VNIFLDDLVGAIAAAAASR**
Found in **WP_005606850.1** in **UB_target**, phosphatidylglycerophosphatase A [Granulicatella adiacens]

Match to Query 12949: 1886.029120 from(944.021836,2+) index(23205)
Title: Elution from: 119.278 to 119.278 period: 0 experiment: 1 cycles: 1 precIntensity: 921366.0 FinneganScanNumber: 28769 MStype: enumIsNormalMS rawFile: 19B022_Kuweit_Sample-GA-EVS.raw
Data file LTQ_19B022_Kuweit_Sample-GA-EVS.mgf

Click mouse within plot area to zoom in by factor of two about that point
Or, [Plot from] 100 to 2000 Da [Full range]
Show Y-axis ☐

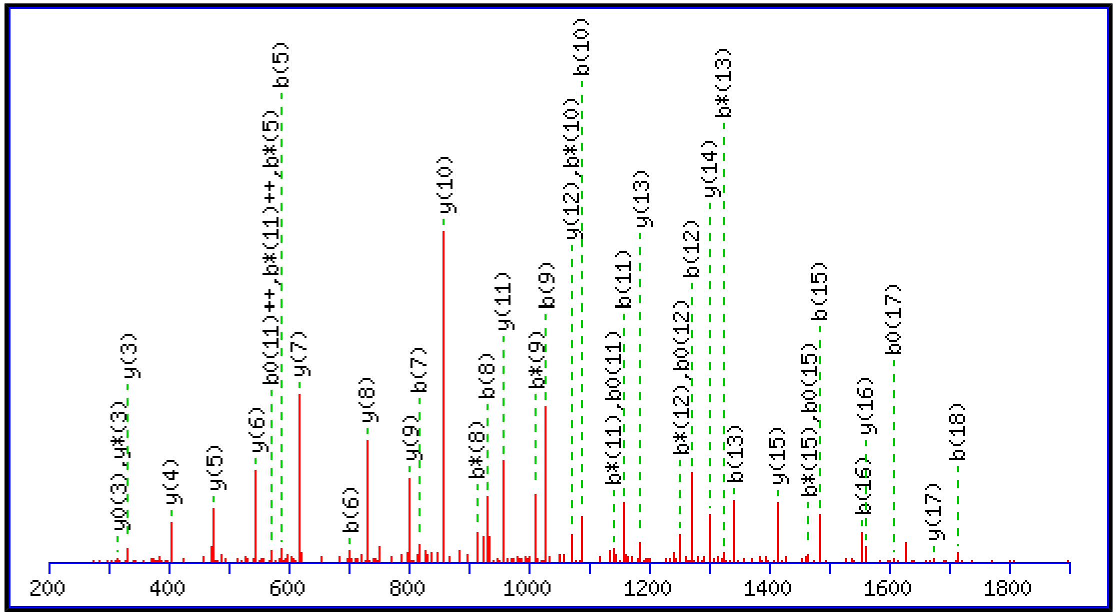

Label all possible matches ○ Label matches used for scoring ⦿

Monoisotopic mass of neutral peptide Mr(calc): 1886.0261
Fixed modifications: Carbamidomethyl (C) (apply to specified residues or termini only)
Ions Score: 133 **Expect:** 1.4e-013

Matches : 43/202 fragment ions using 47 most intense peaks (help)

#	b	b^{++}	b*	b^{*++}	b^0	b^{0++}	Seq.	y	y^{++}	y*	y^{*++}	y^0	y^{0++}	#
1	100.0757	50.5415					V							19
2	214.1186	107.5629	197.0921	99.0497			N	1787.9650	894.4862	1770.9385	885.9729	1769.9545	885.4809	18
3	327.2027	164.1050	310.1761	155.5917			I	1673.9221	837.4647	1656.8955	828.9514	1655.9115	828.4594	17
4	474.2711	237.6392	457.2445	229.1259			F	1560.8380	780.9227	1543.8115	772.4094	1542.8275	771.9174	16
5	587.3552	294.1812	570.3286	285.6679			L	1413.7696	707.3884	1396.7431	698.8752	1395.7591	698.3832	15
6	702.3821	351.6947	685.3556	343.1814	684.3715	342.6894	D	1300.6856	650.8464	1283.6590	642.3331	1282.6750	641.8411	14
7	817.4090	409.2082	800.3825	400.6949	799.3985	400.2029	D	1185.6586	593.3329	1168.6321	584.8197	1167.6480	584.3277	13
8	930.4931	465.7502	913.4666	457.2369	912.4825	456.7449	L	1070.6317	535.8195	1053.6051	527.3062	1052.6211	526.8142	12
9	1029.5615	515.2844	1012.5350	506.7711	1011.5510	506.2791	V	957.5476	479.2774	940.5211	470.7642	939.5370	470.2722	11
10	1086.5830	543.7951	1069.5564	535.2819	1068.5724	534.7898	G	858.4792	429.7432	841.4526	421.2300	840.4686	420.7380	10
11	1157.6201	579.3137	1140.5936	570.8004	1139.6095	570.3084	A	801.4577	401.2325	784.4312	392.7192	783.4472	392.2272	9
12	1270.7042	635.8557	1253.6776	627.3424	1252.6936	626.8504	I	730.4206	365.7139	713.3941	357.2007	712.4100	356.7087	8
13	1341.7413	671.3743	1324.7147	662.8610	1323.7307	662.3690	A	617.3365	309.1719	600.3100	300.6586	599.3260	300.1666	7
14	1412.7784	706.8928	1395.7518	698.3796	1394.7678	697.8876	A	546.2994	273.6534	529.2729	265.1401	528.2889	264.6481	6
15	1483.8155	742.4114	1466.7890	733.8981	1465.8049	733.4061	A	475.2623	238.1348	458.2358	229.6215	457.2518	229.1295	5
16	1554.8526	777.9299	1537.8261	769.4167	1536.8421	768.9247	A	404.2252	202.6162	387.1987	194.1030	386.2146	193.6110	4
17	1625.8897	813.4485	1608.8632	804.9352	1607.8792	804.4432	A	333.1881	167.0977	316.1615	158.5844	315.1775	158.0924	3
18	1712.9218	856.9645	1695.8952	848.4512	1694.9112	847.9592	S	262.1510	131.5791	245.1244	123.0659	244.1404	122.5738	2
19							R	175.1190	88.0631	158.0924	79.5498			1

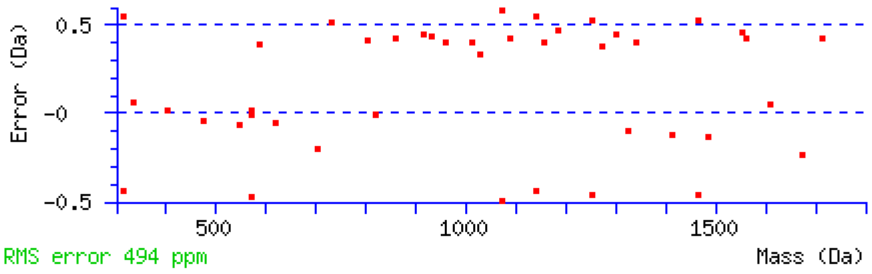

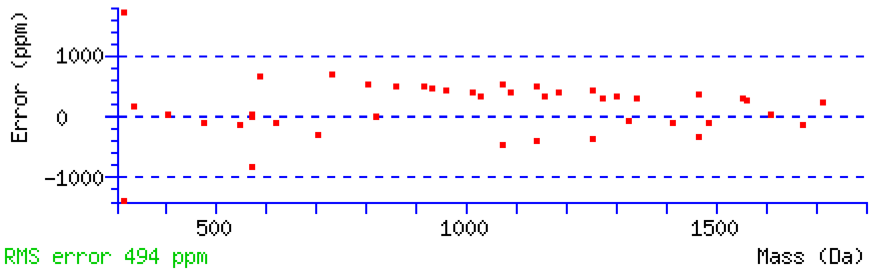

NCBI **BLAST** search of VNIFLDDLVGAIAAAAASR
(Parameters: blastp, nr protein database, expect=20000, no filter, PAM30)

Other BLAST web gateways

All matches to this query

Score	Mr(calc)	Delta	Sequence
133.4	1886.0261	0.0030	VNIFLDDLVGAIAAAAASR
2.8	1886.0275	0.0017	SLFKPKTNLQRHFNR

Mascot: http://www.matrixscience.com/

MASCOT Search Results

Protein View: EEW37841.1

co-chaperone GrpE [Granulicatella adiacens ATCC 49175 ATCC 49175]

**Database:**	UB_target
**Score:**	185
**Monoisotopic mass (M_r):**	21088
**Calculated pI:**	4.56

Sequence similarity is available as an NCBI BLAST search of EEW37841.1 against nr.

Search parameters

**MS data file:**	LTQ_19B022_Kuweit_Sample-GA-EVS.mgf
**Enzyme:**	Trypsin: cuts C-term side of KR unless next residue is P.
**Fixed modifications:**	Carbamidomethyl (C)
**Variable modifications:**	Deamidated (NQ), Oxidation (M)

Protein sequence coverage: 9%

Matched peptides shown in ***bold red***.

```
  1 METNEHNTKE ELIKENVVTD ETPQEEVTEE TTTELSNEEK LQQEVERLND
 51 QVYRLSAEIS NIQKRNAKER QDAAKYRSQS LAQNLLNVID NLERAIASPS
101 ESEDAQNLKK GIEMVYESFL YALKEEGIEE IDALDQPFDP TLHHAVQTVP
151 VEEGQEADKV VQVFQKGYKL KDRVLRPAMV IVSQ
```

Unformatted sequence string: 184 residues (for pasting into other applications).

Sort by ⦿ residue number ○ increasing mass ○ decreasing mass
Show matched peptides only predicted peptides also

Query	Start – End	Observed	Mr(expt)	Mr(calc)	ppm	M	Score	Expect	Rank	U	Peptide
13349	78 – 94	964.0143	1926.0140	1926.0170	-1.55	0	105	6.6e-011	1	U	R.SQSLAQNLLNVIDNLER.A
13350	78 – 94	964.0164	1926.0183	1926.0170	0.66	0	107	4.8e-011	1	U	R.SQSLAQNLLNVIDNLER.A

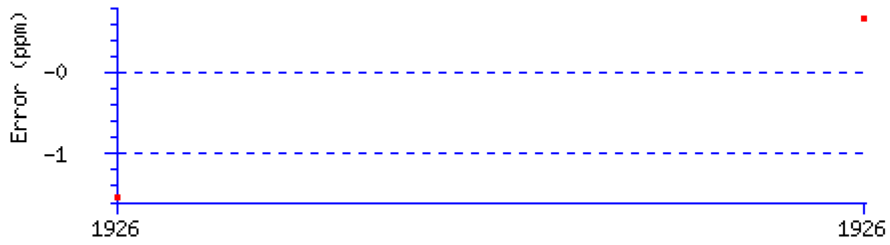

RMS error 1 ppm

Mascot: http://www.matrixscience.com/

MATRIX SCIENCE Mascot Search Results

Peptide View

MS/MS Fragmentation of **SQSLAQNLLNVIDNLER**
Found in **EEW37841.1** in **UB_target**, co-chaperone GrpE [Granulicatella adiacens ATCC 49175 ATCC 49175]

Match to Query 13350: 1926.018280 from(964.016416,2+) index(22845)
Title: Elution from: 117.770 to 117.770 period: 0 experiment: 1 cycles: 1 precIntensity: 328703.0 FinneganScanNumber: 28331 MStype: enumIsNormalMS rawFile: 19B022_Kuweit_Sample-GA-EVS.raw
Data file LTQ_19B022_Kuweit_Sample-GA-EVS.mgf

Click mouse within plot area to zoom in by factor of two about that point
Or, Plot from 150 to 1100 Da Full range
Show Y-axis ☐

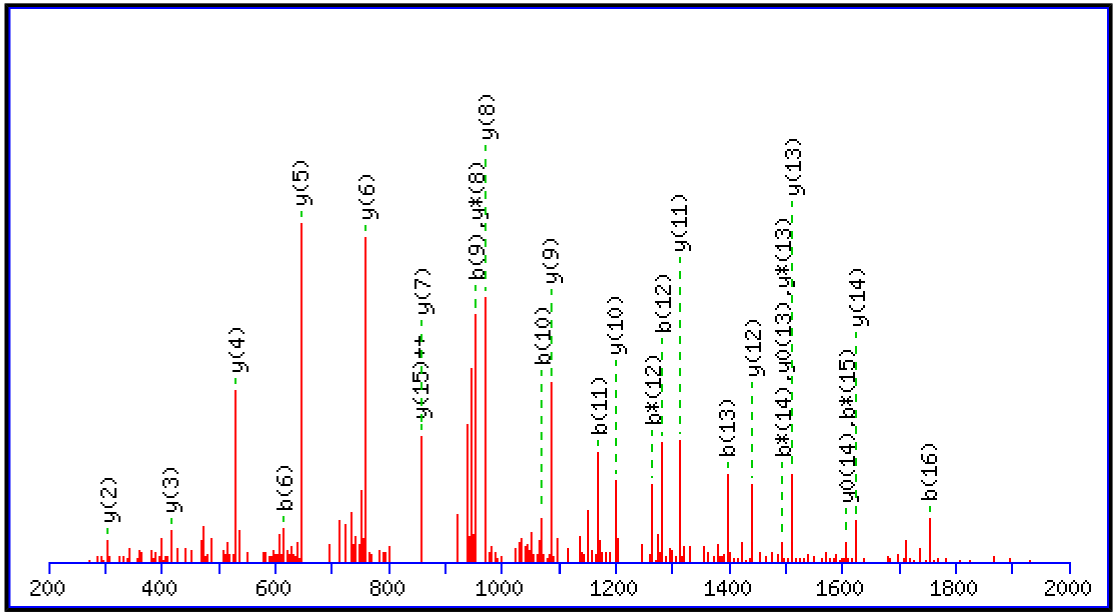

Label all possible matches ○ Label matches used for scoring ⦿

Monoisotopic mass of neutral peptide Mr(calc): 1926.0170
Fixed modifications: Carbamidomethyl (C) (apply to specified residues or termini only)
Ions Score: 107 **Expect:** 4.8e-011

Matches : 28/188 fragment ions using 33 most intense peaks (help)

#	b	b^{++}	b^{*}	b^{*++}	b^{0}	b^{0++}	Seq.	y	y^{++}	y^{*}	y^{*++}	y^{0}	y^{0++}	#
1	88.0393	44.5233			70.0287	35.5180	S							17
2	216.0979	108.5526	199.0713	100.0393	198.0873	99.5473	Q	1839.9923	920.4998	1822.9658	911.9865	1821.9817	911.4945	16
3	303.1299	152.0686	286.1034	143.5553	285.1193	143.0633	S	1711.9337	856.4705	1694.9072	847.9572	1693.9232	847.4652	15
4	416.2140	208.6106	399.1874	200.0974	398.2034	199.6053	L	***1624.9017***	812.9545	1607.8751	804.4412	1606.8911	803.9492	14
5	487.2511	244.1292	470.2245	235.6159	469.2405	235.1239	A	***1511.8176***	756.4125	1494.7911	747.8992	1493.8071	747.4072	13
6	**615.3097**	308.1585	598.2831	299.6452	597.2991	299.1532	Q	***1440.7805***	720.8939	1423.7540	712.3806	1422.7700	711.8886	12
7	729.3526	365.1799	712.3260	356.6667	711.3420	356.1747	N	***1312.7219***	656.8646	1295.6954	648.3513	1294.7114	647.8593	11
8	842.4367	421.7220	825.4101	413.2087	824.4261	412.7167	L	***1198.6790***	599.8431	1181.6525	591.3299	1180.6684	590.8379	10
9	**955.5207**	478.2640	938.4942	469.7507	937.5102	469.2587	L	***1085.5949***	543.3011	1068.5684	534.7878	1067.5844	534.2958	9
10	**1069.5636**	535.2855	1052.5371	526.7722	1051.5531	526.2802	N	***972.5109***	486.7591	955.4843	478.2458	954.5003	477.7538	8
11	**1168.6321**	584.8197	1151.6055	576.3064	1150.6215	575.8144	V	***858.4680***	429.7376	841.4414	421.2243	840.4574	420.7323	7
12	**1281.7161**	641.3617	1264.6896	632.8484	1263.7056	632.3564	I	***759.3995***	380.2034	742.3730	371.6901	741.3890	371.1981	6
13	**1396.7431**	698.8752	1379.7165	690.3619	1378.7325	689.8699	D	***646.3155***	323.6614	629.2889	315.1481	628.3049	314.6561	5
14	1510.7860	755.8966	1493.7594	747.3834	1492.7754	746.8914	N	***531.2885***	266.1479	514.2620	257.6346	513.2780	257.1426	4
15	1623.8701	812.4387	1606.8435	803.9254	1605.8595	803.4334	L	***417.2456***	209.1264	400.2191	200.6132	399.2350	200.1212	3
16	**1752.9127**	876.9600	1735.8861	868.4467	1734.9021	867.9547	E	***304.1615***	152.5844	287.1350	144.0711	286.1510	143.5791	2
17							R	175.1190	88.0631	158.0924	79.5498			1

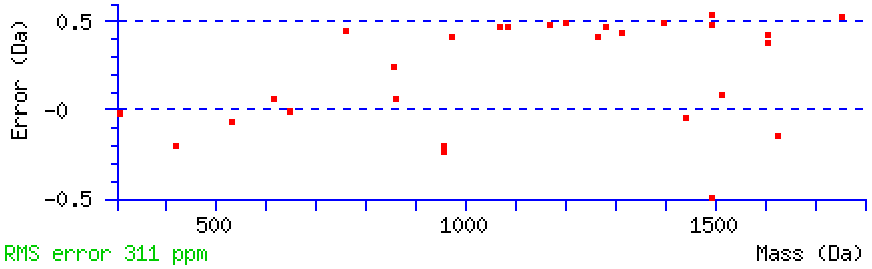

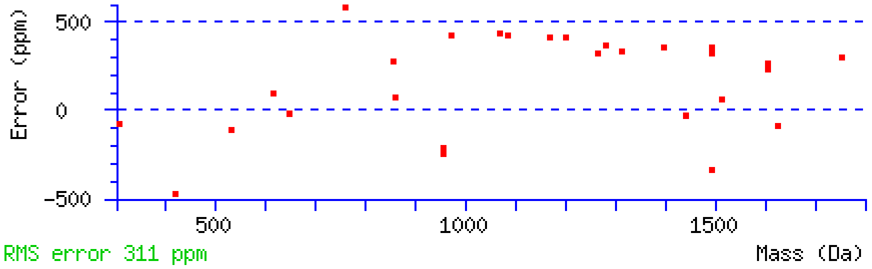

NCBI **BLAST** search of SQSLAQNLLNVIDNLER
(Parameters: blastp, nr protein database, expect=20000, no filter, PAM30)
Other BLAST web gateways

All matches to this query

Score	Mr(calc)	Delta	Sequence
107.3	1926.0170	0.0013	SQSLAQNLLNVIDNLER
5.5	1926.0170	0.0013	RLSNEQNIIAALAIDQR
5.5	1926.0170	0.0013	RLSNEQNIIAALAIDQR

Mascot: http://www.matrixscience.com/

MATRIX SCIENCE MASCOT Search Results

Protein View: EEW38181.1

alcohol dehydrogenase, iron-dependent [Granulicatella adiacens ATCC 49175 ATCC 49175]

**Database:**	UB_target
**Score:**	145
**Monoisotopic mass (M_r):**	39481
**Calculated pI:**	5.13

Sequence similarity is available as an NCBI BLAST search of EEW38181.1 against nr.

Search parameters

**MS data file:**	LTQ_19B022_Kuweit_Sample-GA-EVS.mgf
**Enzyme:**	Trypsin: cuts C-term side of KR unless next residue is P.
**Fixed modifications:**	Carbamidomethyl (C)
**Variable modifications:**	Deamidated (NQ), Oxidation (M)

Protein sequence coverage: 4%

Matched peptides shown in ***bold red***.

```
  1 MIMEKVFASP SRYVQGKDVL KTGLSHVLSL GDRHLLLCDP IVYDLVGKEL
 51 EENLLKEGAF VHREIFHGEA TNDEVHRVAE VVKEHHLNVV IGLGGGKSID
101 TAKAIADDSN CPVAILPTIA STDAPTSALS VIYSAEGVFE RYRFYKKNPE
151 LVLVDTKVIA NSPVRLLISG IADALATWVE ARAVIEAQGG TMVGQVPTLA
201 AEAIARVCES TLFENGLQAV AAANAKVVTP ALEAVVEANT LLSGIGFESA
251 GLAAAHAIHN GFTAIHGDIH SLTHGEKVAY GTLTQLVLEN RPKEELDKYI
301 TFYKALGLPT TLKEVKLDSV PYEDLLKIGT LATQEGETIH QMAVDYTAED
351 VANALLALDQ YVTTRF
```

Unformatted sequence string: 366 residues (for pasting into other applications).

Sort by	residue number	increasing mass	decreasing mass
Show	matched peptides only	predicted peptides also	

Query	Start – End	Observed	Mr(expt)	Mr(calc)	ppm	M	Score	Expect	Rank	U	Peptide
12014	166 – 182	900.0052	1797.9958	1797.9989	-1.72	0	63	6.7e-007	1	U	R.LLISGIADALATWVEAR.A

12015 166 – 182 900.0056 1797.9966 1797.9989 -1.24 0 107 2.8e-011 1 U R.LLISGIADALATWVEAR.A

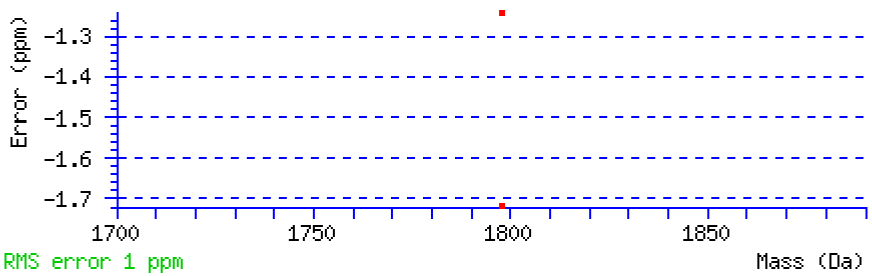

Mascot: http://www.matrixscience.com/

Mascot Search Results

Peptide View

MS/MS Fragmentation of **LLISGIADALATWVEAR**
Found in **EEW38181.1** in **UB_target**, alcohol dehydrogenase, iron-dependent [Granulicatella adiacens ATCC 49175 ATCC 49175]

Match to Query 12014: 1797.995766 from(900.005159,2+) index(23209)
Title: Elution from: 119.287 to 119.287 period: 0 experiment: 1 cycles: 1 precIntensity: 593707.0 FinneganScanNumber: 28773 MStype: enumIsNormalMS rawFile: 19B022_Kuweit_Sample-GA-EVS.raw
Data file LTQ_19B022_Kuweit_Sample-GA-EVS.mgf

Click mouse within plot area to zoom in by factor of two about that point
Or, Plot from 150 to 1100 Da Full range
Show Y-axis

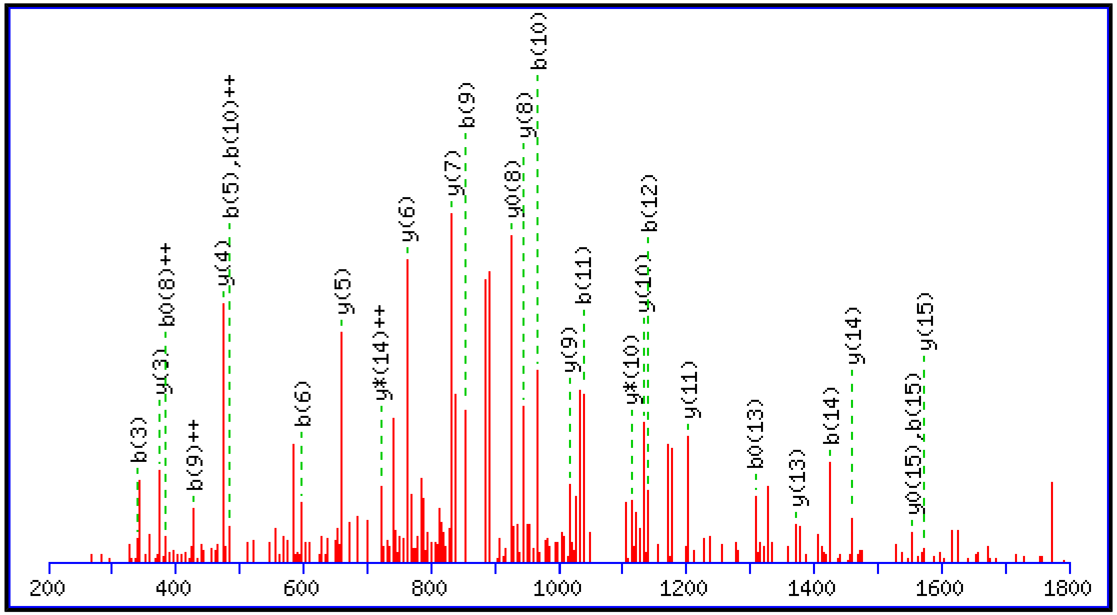

Label all possible matches Label matches used for scoring

Monoisotopic mass of neutral peptide Mr(calc): 1797.9989
Fixed modifications: Carbamidomethyl (C) (apply to specified residues or termini only)
Ions Score: 63 **Expect:** 6.7e-007

Matches : 29/150 fragment ions using 62 most intense peaks (help)

#	b	b^{++}	b^{0}	b^{0++}	Seq.	y	y^{++}	y*	y^{*++}	y^{0}	y^{0++}	#
1	114.0913	57.5493			L							17
2	227.1754	114.0913			L	1685.9221	843.4647	1668.8955	834.9514	1667.9115	834.4594	16
3	**340.2595**	170.6334			I	***1572.8380***	786.9227	1555.8115	778.4094	1554.8275	777.9174	15
4	427.2915	214.1494	409.2809	205.1441	S	***1459.7540***	730.3806	1442.7274	721.8673	1441.7434	721.3753	14
5	**484.3130**	242.6601	466.3024	233.6548	G	***1372.7219***	686.8646	1355.6954	678.3513	1354.7114	677.8593	13
6	**597.3970**	299.2022	579.3865	290.1969	I	1315.7005	658.3539	1298.6739	649.8406	1297.6899	649.3486	12
7	668.4341	334.7207	650.4236	325.7154	A	***1202.6164***	601.8118	1185.5899	593.2986	1184.6058	592.8066	11
8	783.4611	392.2342	765.4505	383.2289	D	***1131.5793***	566.2933	1114.5528	557.7800	1113.5687	557.2880	10
9	**854.4982**	427.7527	836.4876	418.7475	A	***1016.5524***	508.7798	999.5258	500.2665	998.5418	499.7745	9
10	**967.5823**	484.2948	949.5717	475.2895	L	***945.5152***	473.2613	928.4887	464.7480	927.5047	464.2560	8
11	**1038.6194**	519.8133	1020.6088	510.8080	A	***832.4312***	416.7192	815.4046	408.2060	814.4206	407.7139	7
12	**1139.6671**	570.3372	1121.6565	561.3319	T	***761.3941***	381.2007	744.3675	372.6874	743.3835	372.1954	6
13	1325.7464	663.3768	1307.7358	654.3715	W	***660.3464***	330.6768	643.3198	322.1636	642.3358	321.6715	5
14	**1424.8148**	712.9110	1406.8042	703.9057	V	***474.2671***	237.6372	457.2405	229.1239	456.2565	228.6319	4
15	**1553.8574**	777.4323	1535.8468	768.4270	E	***375.1987***	188.1030	358.1721	179.5897	357.1881	179.0977	3
16	1624.8945	812.9509	1606.8839	803.9456	A	246.1561	123.5817	229.1295	115.0684			2
17					R	175.1190	88.0631	158.0924	79.5498			1

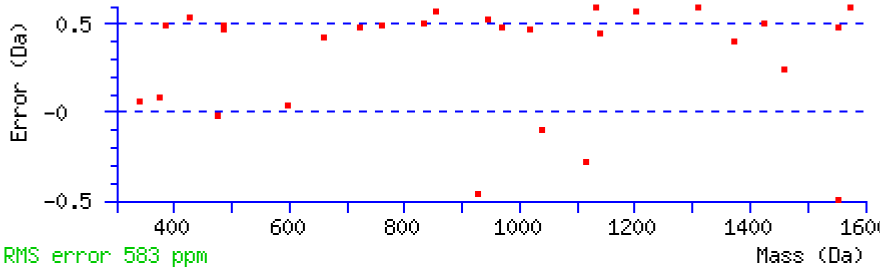

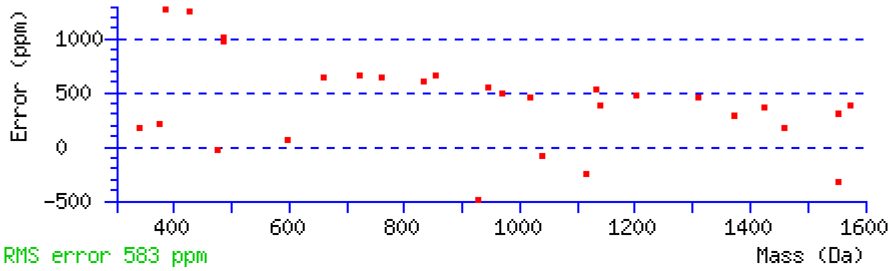

NCBI **BLAST** search of LLISGIADALATWVEAR
(Parameters: blastp, nr protein database, expect=20000, no filter, PAM30)
Other BLAST web gateways

All matches to this query

Score	Mr(calc)	Delta	Sequence
63.0	1797.9989	-0.0031	LLISGIADALATWVEAR

Mascot: http://www.matrixscience.com/

MASCOT Search Results

Protein View: RKW28577.1

50S ribosomal protein L21 [Granulicatella sp.]

**Database:**	UB_target
**Score:**	139
**Monoisotopic mass (M_r):**	11214
**Calculated pI:**	9.80

Sequence similarity is available as an NCBI BLAST search of RKW28577.1 against nr.

Search parameters

**MS data file:**	LTQ_19B022_Kuweit_Sample-GA-EVS.mgf
**Enzyme:**	Trypsin: cuts C-term side of KR unless next residue is P.
**Fixed modifications:**	Carbamidomethyl (C)
**Variable modifications:**	Deamidated (NQ), Oxidation (M)

Protein sequence coverage: 16%

Matched peptides shown in ***bold red***.

```
  1 MYAIVKTGGK QVKVEVGQAI YVEKLNAEAG DKVTFEEVVF VGGDDVKVGA
 51 PFVAGATVEG TVEKQGRQKK VVTFKYKRRK DSHRKQGHRQ PYTKVVINAI
101 NA
```

Unformatted sequence string: 102 residues (for pasting into other applications).

Sort by	residue number	increasing mass	decreasing mass
Show	matched peptides only	predicted peptides also	

Query	Start – End	Observed	Mr(expt)	Mr(calc)	ppm	M	Score	Expect	Rank	U	Peptide
10484	48 – 64	816.4335	1630.8525	1630.8567	-2.57	0	73	2.1e-007	1	U	K.VGAPFVAGATVEGTVEK.Q
10485	48 – 64	816.4339	1630.8533	1630.8567	-2.04	0	77	9.3e-008	1	U	K.VGAPFVAGATVEGTVEK.Q
10486	48 – 64	816.4355	1630.8564	1630.8567	-0.17	0	50	4.4e-005	1	U	K.VGAPFVAGATVEGTVEK.Q

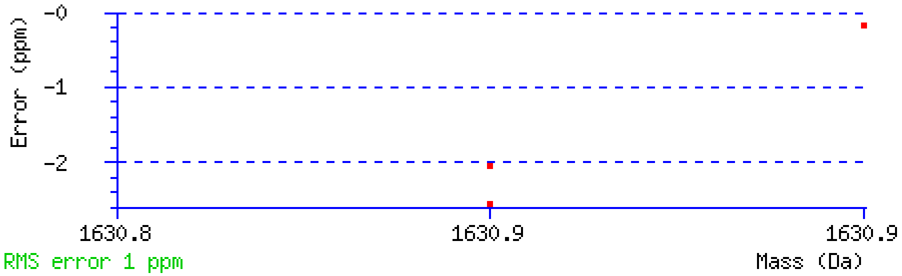

Mascot: http://www.matrixscience.com/

MATRIX SCIENCE Mascot Search Results

Peptide View

MS/MS Fragmentation of **VGAPFVAGATVEGTVEK**
Found in **RKW28577.1** in **UB_target**, 50S ribosomal protein L21 [Granulicatella sp.]

Match to Query 10485: 1630.853336 from(816.433944,2+) index(13502)
Title: Elution from: 82.403 to 82.403 period: 0 experiment: 1 cycles: 1 precIntensity: 487568.0 FinneganScanNumber: 17396 MStype: enumIsNormalMS
rawFile: 19B022_Kuweit_Sample-GA-EVS.raw
Data file LTQ_19B022_Kuweit_Sample-GA-EVS.mgf

Click mouse within plot area to zoom in by factor of two about that point
Or, Plot from 150 to 1100 Da Full range
Show Y-axis

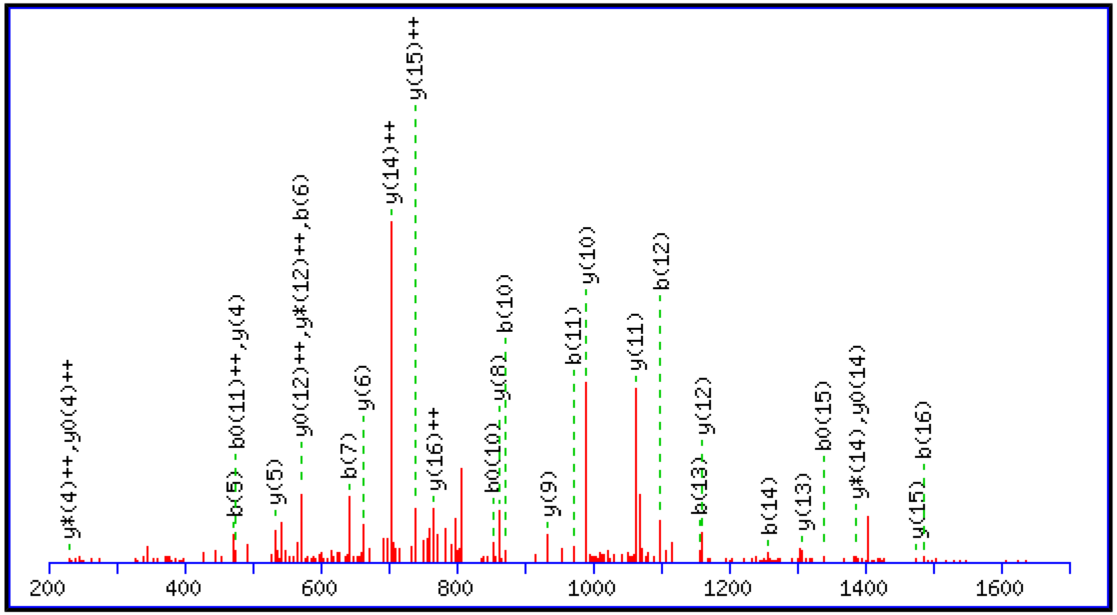

Label all possible matches Label matches used for scoring

Monoisotopic mass of neutral peptide Mr(calc): 1630.8567
Fixed modifications: Carbamidomethyl (C) (apply to specified residues or termini only)
Ions Score: 77 **Expect:** 9.3e-008

Matches : 31/140 fragment ions using 42 most intense peaks (help)

#	b	b^{++}	b^0	b^{0++}	Seq.	y	y^{++}	y*	y^{*++}	y^0	y^{0++}	#
1	100.0757	50.5415			V							17
2	157.0972	79.0522			G	1532.7955	**766.9014**	1515.7690	758.3881	1514.7849	757.8961	16
3	228.1343	114.5708			A	***1475.7740***	**738.3907**	1458.7475	729.8774	1457.7635	729.3854	15
4	325.1870	163.0972			P	1404.7369	**702.8721**	1387.7104	694.3588	1386.7264	693.8668	14
5	***472.2554***	236.6314			F	***1307.6842***	654.3457	1290.6576	645.8324	1289.6736	645.3404	13
6	***571.3239***	286.1656			V	***1160.6157***	580.8115	1143.5892	572.2982	1142.6052	571.8062	12
7	***642.3610***	321.6841			A	***1061.5473***	531.2773	1044.5208	522.7640	1043.5368	522.2720	11
8	699.3824	350.1949			G	***990.5102***	495.7587	973.4837	487.2455	972.4997	486.7535	10
9	770.4196	385.7134			A	***933.4888***	467.2480	916.4622	458.7347	915.4782	458.2427	9
10	***871.4672***	436.2373	853.4567	427.2320	T	***862.4516***	431.7295	845.4251	423.2162	844.4411	422.7242	8
11	***970.5356***	485.7715	952.5251	476.7662	V	761.4040	381.2056	744.3774	372.6923	743.3934	372.2003	7
12	***1099.5782***	550.2928	1081.5677	541.2875	E	***662.3355***	331.6714	645.3090	323.1581	644.3250	322.6661	6
13	***1156.5997***	578.8035	1138.5891	569.7982	G	***533.2930***	267.1501	516.2664	258.6368	515.2824	258.1448	5
14	***1257.6474***	629.3273	1239.6368	620.3220	T	***476.2715***	238.6394	459.2449	230.1261	458.2609	229.6341	4
15	1356.7158	678.8615	1338.7052	669.8563	V	375.2238	188.1155	358.1973	179.6023	357.2132	179.1103	3
16	***1485.7584***	743.3828	1467.7478	734.3775	E	276.1554	138.5813	259.1288	130.0681	258.1448	129.5761	2
17					K	147.1128	74.0600	130.0863	65.5468			1

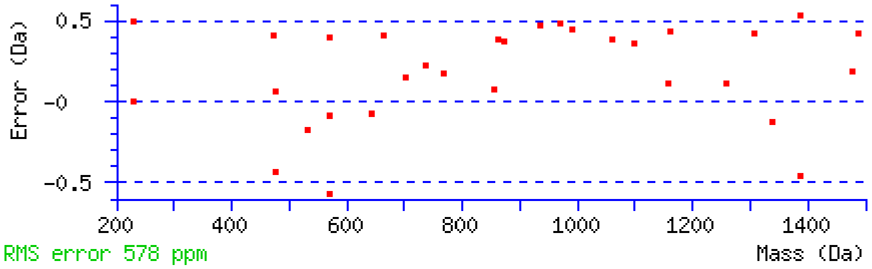

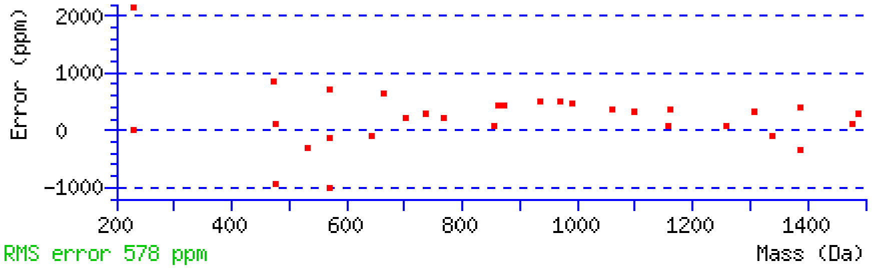

NCBI **BLAST** search of VGAPFVAGATVEGTVEK
(Parameters: blastp, nr protein database, expect=20000, no filter, PAM30)
Other BLAST web gateways

All matches to this query

Score	Mr(calc)	Delta	Sequence
76.6	1630.8567	-0.0033	VGAPFVAGATVEGTVEK
4.8	1630.8525	0.0008	SNRIKEISLEEANK

Mascot: http://www.matrixscience.com/

MASCOT Search Results

Protein View: EEW37447.1

thioredoxin [Granulicatella adiacens ATCC 49175 ATCC 49175]

**Database:**	UB_target
**Score:**	121
**Monoisotopic mass (M_r):**	12113
**Calculated pI:**	4.62

Sequence similarity is available as an NCBI BLAST search of EEW37447.1 against nr.

Search parameters

**MS data file:**	LTQ_19B022_Kuweit_Sample-GA-EVS.mgf
**Enzyme:**	Trypsin: cuts C-term side of KR unless next residue is P.
**Fixed modifications:**	Carbamidomethyl (C)
**Variable modifications:**	Deamidated (NQ), Oxidation (M)

Protein sequence coverage: 12%

Matched peptides shown in ***bold red***.

```
  1 MIRMVKSLTD KNFVEETKDG VVLVDFWATW CGPCRMQGPV IDQLDEEMGD
 51 KVTFTKVDVD ENPETARAFG IMSIPTLLIK KDGEVVNKVV GYHAKEQLEE
101 LLAQYL
```

Unformatted sequence string: 106 residues (for pasting into other applications).

Sort by	residue number	increasing mass	decreasing mass
Show	matched peptides only	predicted peptides also	

Query	Start – End	Observed	Mr(expt)	Mr(calc)	ppm	M	Score	Expect	Rank	U	Peptide
8535	68 – 80	702.4199	1402.8252	1402.8258	-0.41	0	65	2.9e-007	1	U	R.AFGIMSIPTLLIK.K
8536	68 – 80	702.4200	1402.8255	1402.8258	-0.17	0	54	5.3e-006	1	U	R.AFGIMSIPTLLIK.K
8537	68 – 80	702.4201	1402.8256	1402.8258	-0.12	0	51	8.7e-006	1	U	R.AFGIMSIPTLLIK.K

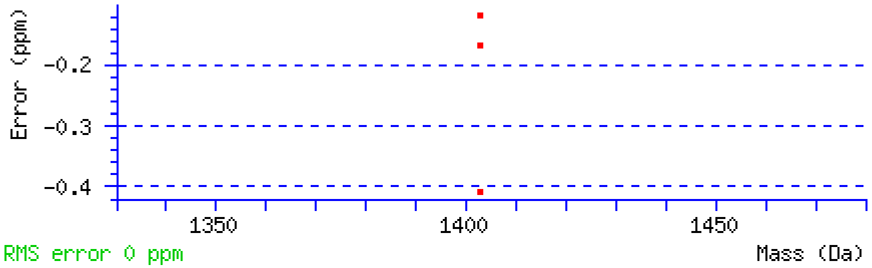

Mascot: http://www.matrixscience.com/

Mascot Search Results

Peptide View

MS/MS Fragmentation of **AFGIMSIPTLLIK**
Found in **EEW37447.1** in **UB_target**, thioredoxin [Granulicatella adiacens ATCC 49175 ATCC 49175]

Match to Query 8535: 1402.825200 from(702.419876,2+) index(21643)
Title: Elution from: 112.973 to 112.973 period: 0 experiment: 1 cycles: 1 precIntensity: 510296.0 FinneganScanNumber: 26909 MStype: enumIsNormalMS rawFile: 19B022_Kuweit_Sample-GA-EVS.raw
Data file LTQ_19B022_Kuweit_Sample-GA-EVS.mgf

Click mouse within plot area to zoom in by factor of two about that point
Or, Plot from 100 to 1400 Da Full range
Show Y-axis

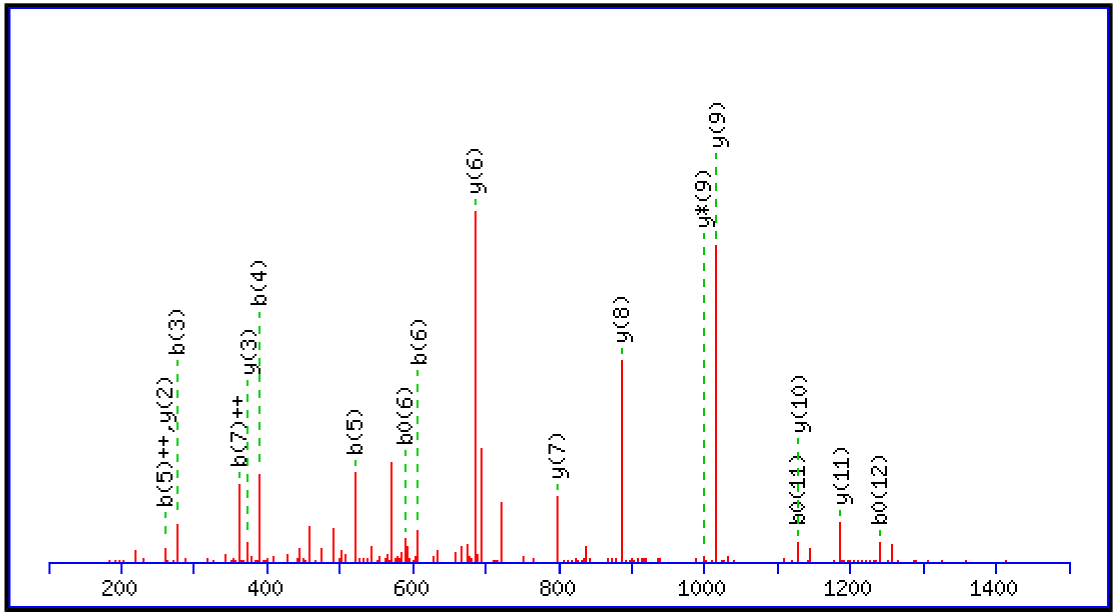

Label all possible matches Label matches used for scoring

Monoisotopic mass of neutral peptide Mr(calc): 1402.8258
Fixed modifications: Carbamidomethyl (C) (apply to specified residues or termini only)
Ions Score: 65 **Expect:** 2.9e-007

Matches : 18/102 fragment ions using 24 most intense peaks (help)

#	b	b^{++}	b^{0}	b^{0++}	Seq.	y	y^{++}	y*	y^{*++}	y^{0}	y^{0++}	#
1	72.0444	36.5258			A							13
2	219.1128	110.0600			F	1332.7960	666.9016	1315.7694	658.3883	1314.7854	657.8963	12
3	**276.1343**	138.5708			G	***1185.7275***	593.3674	1168.7010	584.8541	1167.7170	584.3621	11
4	**389.2183**	195.1128			I	***1128.7061***	564.8567	1111.6795	556.3434	1110.6955	555.8514	10
5	**520.2588**	**260.6330**			M	***1015.6220***	508.3146	998.5955	499.8014	997.6115	499.3094	9
6	**607.2908**	304.1491	589.2803	295.1438	S	***884.5815***	442.7944	867.5550	434.2811	866.5710	433.7891	8
7	720.3749	**360.6911**	702.3643	351.6858	I	***797.5495***	399.2784	780.5230	390.7651	779.5389	390.2731	7
8	817.4277	409.2175	799.4171	400.2122	P	***684.4654***	342.7364	667.4389	334.2231	666.4549	333.7311	6
9	918.4754	459.7413	900.4648	450.7360	T	587.4127	294.2100	570.3861	285.6967	569.4021	285.2047	5
10	1031.5594	516.2833	1013.5489	507.2781	L	486.3650	243.6861	469.3384	235.1729			4
11	1144.6435	572.8254	1126.6329	563.8201	L	***373.2809***	187.1441	356.2544	178.6308			3
12	1257.7275	629.3674	1239.7170	620.3621	I	***260.1969***	130.6021	243.1703	122.0888			2
13					K	147.1128	74.0600	130.0863	65.5468			1

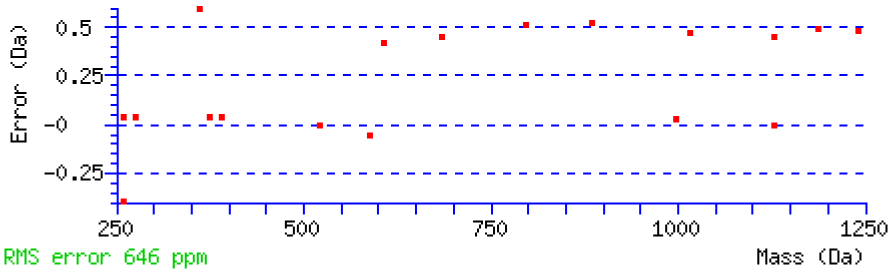

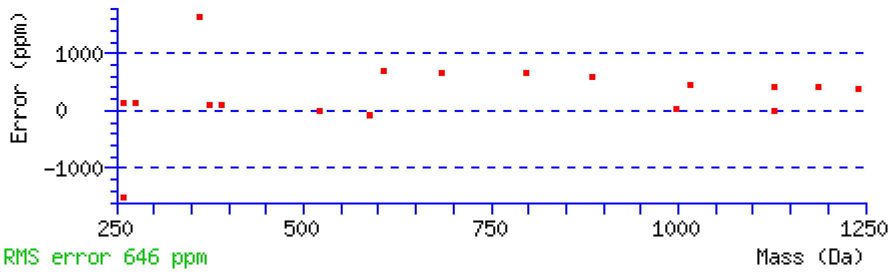

NCBI **BLAST** search of AFGIMSIPTLLIK
(Parameters: blastp, nr protein database, expect=20000, no filter, PAM30)
Other BLAST web gateways

All matches to this query

Score	Mr(calc)	Delta	Sequence
65.3	1402.8258	-0.0006	AFGIMSIPTLLIK

Mascot: http://www.matrixscience.com/

MATRIX SCIENCE MASCOT Search Results

Protein View: RKW29913.1

flavocytochrome c [Granulicatella sp.]

**Database:**	UB_target
**Score:**	120
**Monoisotopic mass (M_r):**	62818
**Calculated pI:**	5.81

Sequence similarity is available as an NCBI BLAST search of RKW29913.1 against nr.

Search parameters

**MS data file:**	LTQ_19B022_Kuweit_Sample-GA-EVS.mgf
**Enzyme:**	Trypsin: cuts C-term side of KR unless next residue is P.
**Fixed modifications:**	Carbamidomethyl (C)
**Variable modifications:**	Deamidated (NQ), Oxidation (M)

Protein sequence coverage: 2%

Matched peptides shown in ***bold red***.

```
  1 MKKSLNVLLL SILSILLLFG CSTAKQSGKF EGVGTGKHGE IKVAVTITDA
 51 KITNIEVIEQ GENKGLSEPV YEDLKEAIIA QNSADVDVVS GASATSEGYL
101 AAVKDAITKS GIKLVASKQT KTKKSELPTE QTFDVVIVGS GGAGLSAAIE
151 AAKAGKSVAI VEKMPTVGGN TLISGGEMNA PGNWVQKNLG ITGDSVEAYY
201 NDTMKGGDNI GDPKLVHLMA EKALESAEWL RDDVHVEFLS DQLFQFGGHS
251 FKRALIPKGH TGAELVSKLK AKAEELGVKI FLNVKAEELI QDANGRVTGV
301 KATDKTKKEV TFLATNGVVL TTGGFGSNIE MRKQYNKEYD ERYKSTDTVG
351 TTGDGIVMAQ KAGAALKNME YIQTYPIANP KTGMISLLAD TRFDGAILVN
401 QEGKRFVEEL DRRDVISKAI LAQTGGYTYQ IWNDKIDAIS KTKEAHKAEY
451 DELIREGLLV KADTIEEAAK FFDIDINNLK ETIAKVNEYA KNKDDKDFHH
501 RGGLVSLEEG PYYIQKAVPS VHHTMGGLVI NEKAEVLNDK GEAIPGLYAA
551 GELTGVIQGK NRLGGNAITD IITFGRIAGK EVSH
```

Unformatted sequence string: 584 residues (for pasting into other applications).

Sort by	residue number	increasing mass	decreasing mass
Show	matched peptides only	predicted peptides also	

Query	Start – End	Observed	Mr(expt)	Mr(calc)	ppm	M	Score	Expect	Rank	U	Peptide
10919	368 – 381	841.4143	1680.8140	1680.8181	-2.46	0	55	1.2e-005	1	U	K.NMEYIQTYPIANPK.T
10920	368 – 381	841.4147	1680.8149	1680.8181	-1.88	0	68	5.6e-007	1	U	K.NMEYIQTYPIANPK.T
10921	368 – 381	841.4163	1680.8181	1680.8181	-0.0024	0	55	1.5e-005	1	U	K.NMEYIQTYPIANPK.T
10922	368 – 381	841.4164	1680.8183	1680.8181	0.100	0	33	0.0024	1	U	K.NMEYIQTYPIANPK.T

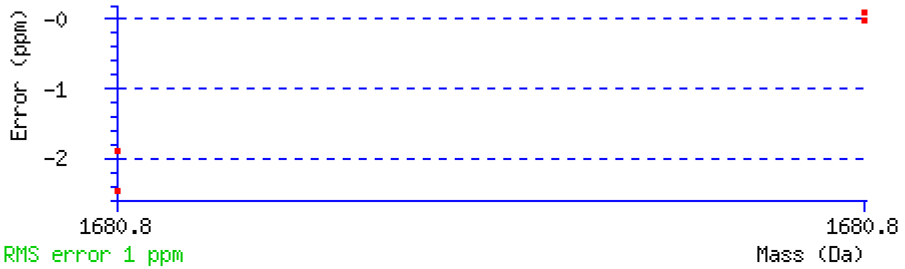

RMS error 1 ppm

Mascot: http://www.matrixscience.com/

MATRIX SCIENCE Mascot Search Results

Peptide View

MS/MS Fragmentation of **NMEYIQTYPIANPK**
Found in **RKW29913.1** in **UB_target**, flavocytochrome c [Granulicatella sp.]

Match to Query 10920: 1680.814942 from(841.414747,2+) index(13720)
Title: Elution from: 83.227 to 83.227 period: 0 experiment: 1 cycles: 1 precIntensity: 271705.0 FinneganScanNumber: 17653 MStype: enumIsNormalMS rawFile: 19B022_Kuweit_Sample-GA-EVS.raw
Data file LTQ_19B022_Kuweit_Sample-GA-EVS.mgf

Click mouse within plot area to zoom in by factor of two about that point
Or, Plot from 150 to 1100 Da Full range
Show Y-axis

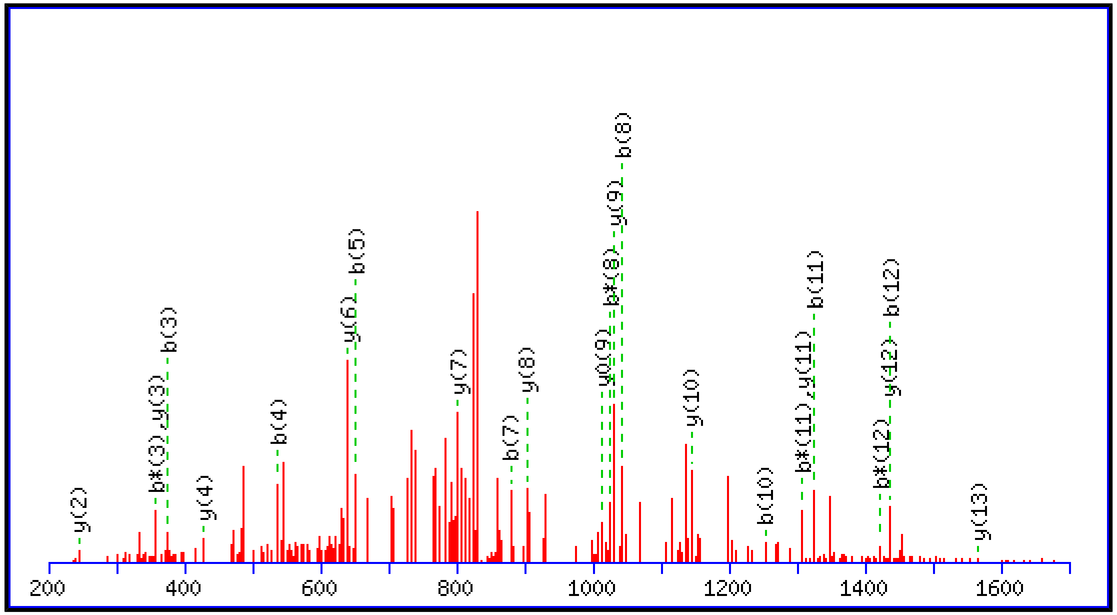

Label all possible matches Label matches used for scoring

Monoisotopic mass of neutral peptide Mr(calc): 1680.8181
Fixed modifications: Carbamidomethyl (C) (apply to specified residues or termini only)
Ions Score: 68 **Expect:** 5.6e-007

Matches : 24/138 fragment ions using 45 most intense peaks (help)

#	b	b^{++}	b*	b*$^{++}$	b^{0}	b^{0++}	Seq.	y	y^{++}	y*	y*$^{++}$	y^{0}	y^{0++}	#
1	115.0502	58.0287	98.0237	49.5155			N							14
2	246.0907	123.5490	229.0641	115.0357			M	***1567.7825***	784.3949	1550.7559	775.8816	1549.7719	775.3896	13
3	**375.1333**	188.0703	358.1067	179.5570	357.1227	179.0650	E	***1436.7420***	718.8746	1419.7155	710.3614	1418.7314	709.8694	12
4	**538.1966**	269.6019	521.1701	261.0887	520.1860	260.5967	Y	***1307.6994***	654.3533	1290.6729	645.8401	1289.6889	645.3481	11
5	**651.2807**	326.1440	634.2541	317.6307	633.2701	317.1387	I	***1144.6361***	572.8217	1127.6095	564.3084	1126.6255	563.8164	10
6	779.3393	390.1733	762.3127	381.6600	761.3287	381.1680	Q	***1031.5520***	516.2796	1014.5255	507.7664	1013.5415	507.2744	9
7	**880.3869**	440.6971	863.3604	432.1838	862.3764	431.6918	T	***903.4934***	452.2504	886.4669	443.7371	885.4829	443.2451	8
8	**1043.4503**	522.2288	1026.4237	513.7155	1025.4397	513.2235	Y	***802.4458***	401.7265	785.4192	393.2132			7
9	1140.5030	570.7552	1123.4765	562.2419	1122.4925	561.7499	P	***639.3824***	320.1949	622.3559	311.6816			6
10	**1253.5871**	627.2972	1236.5605	618.7839	1235.5765	618.2919	I	542.3297	271.6685	525.3031	263.1552			5
11	**1324.6242**	662.8157	1307.5977	654.3025	1306.6136	653.8105	A	***429.2456***	215.1264	412.2191	206.6132			4
12	**1438.6671**	719.8372	1421.6406	711.3239	1420.6566	710.8319	N	***358.2085***	179.6079	341.1819	171.0946			3
13	1535.7199	768.3636	1518.6933	759.8503	1517.7093	759.3583	P	***244.1656***	122.5864	227.1390	114.0731			2
14							K	147.1128	74.0600	130.0863	65.5468			1

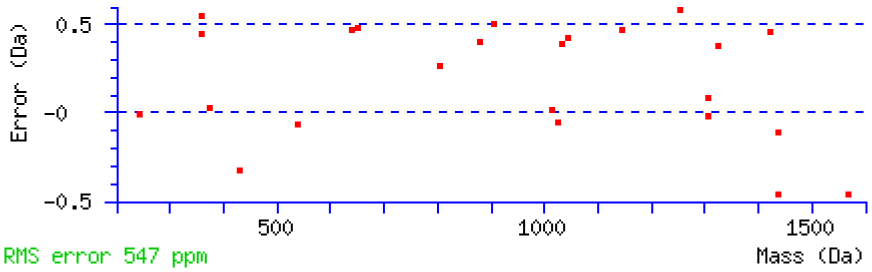

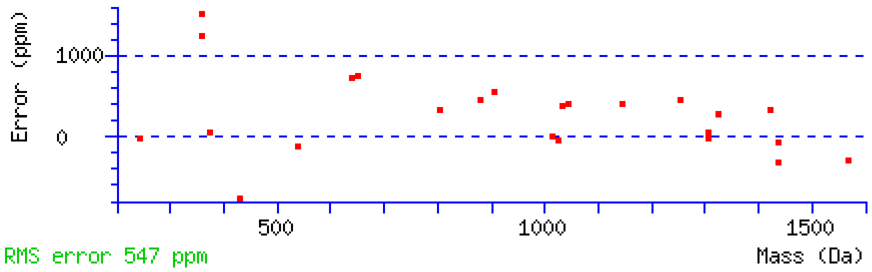

NCBI **BLAST** search of NMEYIQTYPIANPK
(Parameters: blastp, nr protein database, expect=20000, no filter, PAM30)
Other BLAST web gateways

All matches to this query

Score	Mr(calc)	Delta	Sequence
67.8	1680.8181	-0.0032	NMEYIQTYPIANPK

Mascot: http://www.matrixscience.com/

MATRIX SCIENCE MASCOT Search Results

Protein View: WP_005605411.1

Nif3-like dinuclear metal center hexameric protein [Granulicatella adiacens]

**Database:**	UB_target
**Score:**	119
**Monoisotopic mass (M_r):**	42264
**Calculated pI:**	5.30

Sequence similarity is available as an NCBI BLAST search of WP_005605411.1 against nr.

Search parameters

**MS data file:**	LTQ_19B022_Kuweit_Sample-GA-EVS.mgf
**Enzyme:**	Trypsin: cuts C-term side of KR unless next residue is P.
**Fixed modifications:**	Carbamidomethyl (C)
**Variable modifications:**	Deamidated (NQ), Oxidation (M)

Protein sequence coverage: 3%

Matched peptides shown in ***bold red***.

```
  1 MPTVRDVVSR FEKRVPKSLS VPKDPIGLHF GDWNQEVKVI MTTLDIRPSV
 51 IEEAIAKNVD LIIAHHPPIF RPVALFDLTV PQNKMFQQIL KHDIAVYAAH
101 TNLDVVKGGV NDWLADELLL TDVTVMSPTT TIPSVKVITY VPKNNAEKVL
151 EAMFEAGAGA IGDNYKDCAF QTSGVGQFTP VKGANPAIGS LNKPEFVEEV
201 KLEVVCSEEV LSDVLKSLRA SHPYEEPAID VFSLKNAGKT LGFGRVGNLP
251 KAMTGDEFIA FVTERFKLKG LRYVPSRVNP DGLISRVAVM GGSGGNYYLD
301 ALKNGADAFV TGDIFYHTAH DIQETSLFLV DAGHHIEVVC RKQLAKWARE
351 WKEENNWDVT VIESETFTDP FEFYKAGEEN A
```

Unformatted sequence string: 381 residues (for pasting into other applications).

Sort by	residue number	increasing mass	decreasing mass
Show	matched peptides only	predicted peptides also	

Query	Start – End	Observed	Mr(expt)	Mr(calc)	ppm	M	Score	Expect	Rank	U	Peptide
10060	252 – 265	793.8784	1585.7423	1585.7446	-1.46	0	72	1.2e-007	1	U	K.AMTGDEFIAFVTER.F

10061	252 - 265	793.8801	1585.7457	1585.7446	0.65	0	75	9.1e-008	1	U	K.AMTGDEFIAFVTER.F

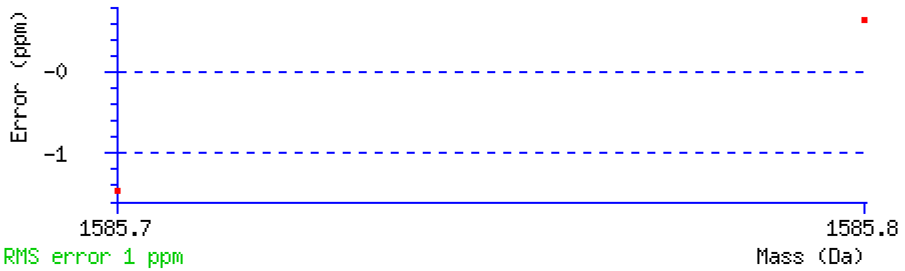

Mascot: http://www.matrixscience.com/

MATRIX SCIENCE Mascot Search Results

Peptide View

MS/MS Fragmentation of **AMTGDEFIAFVTER**
Found in **WP_005605411.1** in **UB_target**, Nif3-like dinuclear metal center hexameric protein [Granulicatella adiacens]

Match to Query 10061: 1585.745672 from(793.880112,2+) index(19951)
Title: Elution from: 106.513 to 106.513 period: 0 experiment: 1 cycles: 1 precIntensity: 266834.0 FinneganScanNumber: 24929 MStype: enumIsNormalMS rawFile: 19B022_Kuweit_Sample-GA-EVS.raw
Data file LTQ_19B022_Kuweit_Sample-GA-EVS.mgf

Click mouse within plot area to zoom in by factor of two about that point
Or, Plot from 100 to 2000 Da Full range
Show Y-axis ☐

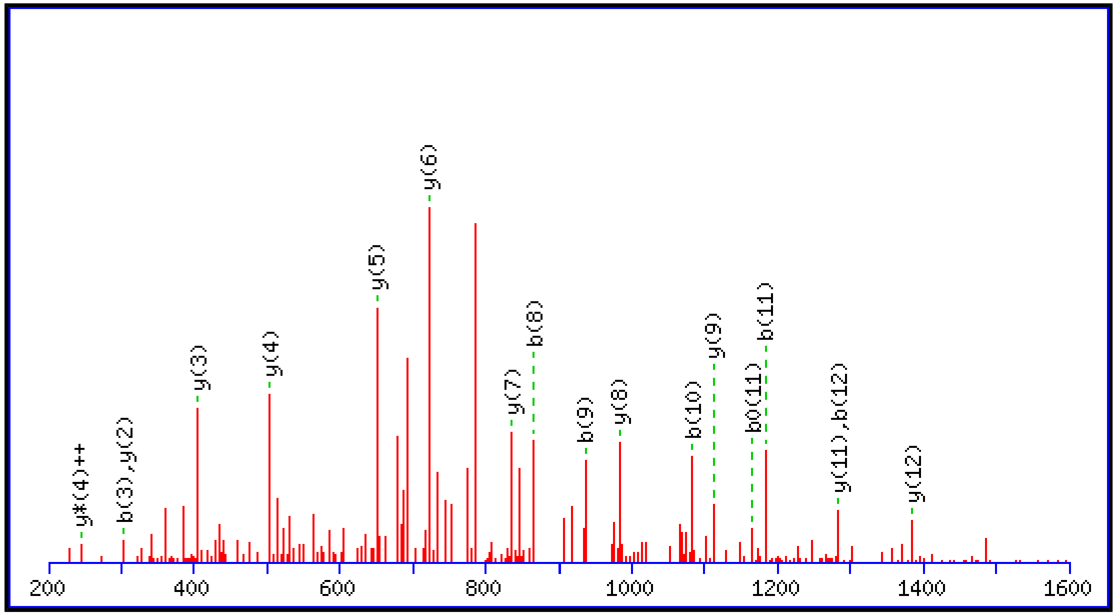

Label all possible matches   Label matches used for scoring

Monoisotopic mass of neutral peptide Mr(calc): 1585.7446
Fixed modifications: Carbamidomethyl (C) (apply to specified residues or termini only)
Ions Score: 75 **Expect:** 9.1e-008

Matches : 18/124 fragment ions using 28 most intense peaks (help)

#	b	b^{++}	b^{0}	b^{0++}	Seq.	y	y^{++}	y*	y^{*++}	y^{0}	y^{0++}	#
1	72.0444	36.5258			A							14
2	203.0849	102.0461			M	1515.7148	758.3610	1498.6883	749.8478	1497.7042	749.3558	13
3	**304.1326**	152.5699	286.1220	143.5646	T	***1384.6743***	692.8408	1367.6478	684.3275	1366.6638	683.8355	12
4	361.1540	181.0806	343.1435	172.0754	G	***1283.6266***	642.3170	1266.6001	633.8037	1265.6161	633.3117	11
5	476.1810	238.5941	458.1704	229.5888	D	1226.6052	613.8062	1209.5786	605.2930	1208.5946	604.8009	10
6	605.2236	303.1154	587.2130	294.1101	E	***1111.5782***	556.2928	1094.5517	547.7795	1093.5677	547.2875	9
7	752.2920	376.6496	734.2814	367.6443	F	***982.5356***	491.7715	965.5091	483.2582	964.5251	482.7662	8
8	**865.3760**	433.1917	847.3655	424.1864	I	***835.4672***	418.2373	818.4407	409.7240	817.4567	409.2320	7
9	**936.4131**	468.7102	918.4026	459.7049	A	***722.3832***	361.6952	705.3566	353.1819	704.3726	352.6899	6
10	**1083.4816**	542.2444	1065.4710	533.2391	F	***651.3461***	326.1767	634.3195	317.6634	633.3355	317.1714	5
11	**1182.5500**	591.7786	1164.5394	582.7733	V	***504.2776***	252.6425	487.2511	244.1292	486.2671	243.6372	4
12	**1283.5977**	642.3025	1265.5871	633.2972	T	***405.2092***	203.1083	388.1827	194.5950	387.1987	194.1030	3
13	1412.6402	706.8238	1394.6297	697.8185	E	***304.1615***	152.5844	287.1350	144.0711	286.1510	143.5791	2
14					R	175.1190	88.0631	158.0924	79.5498			1

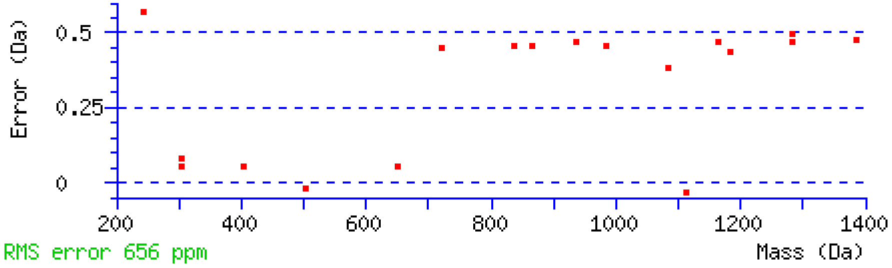

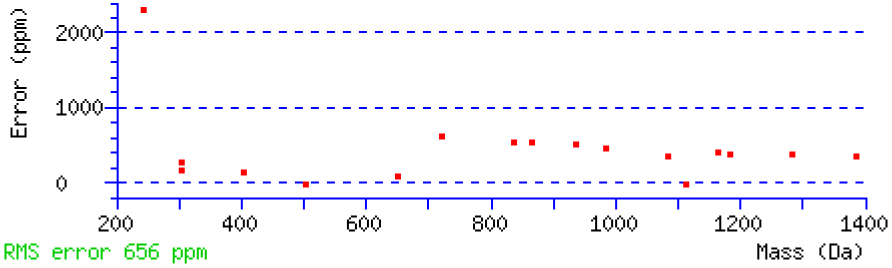

NCBI **BLAST** search of AMTGDEFIAFVTER
(Parameters: blastp, nr protein database, expect=20000, no filter, PAM30)
Other BLAST web gateways

All matches to this query

Score	Mr(calc)	Delta	Sequence
74.8	1585.7446	0.0010	AMTGDEFIAFVTER

9.8	1585.7480	-0.0023	MKEVMQEINKFR
2.0	1585.7446	0.0011	MGFFKQLNNSIER

Mascot: http://www.matrixscience.com/

MASCOT Search Results

Protein View: WP_005606374.1

hypothetical protein [Granulicatella]

**Database:**	UB_target
**Score:**	112
**Monoisotopic mass (M_r):**	36829
**Calculated pI:**	8.46

Sequence similarity is available as an NCBI BLAST search of WP_005606374.1 against nr.

Search parameters

**MS data file:**	LTQ_19B022_Kuweit_Sample-GA-EVS.mgf
**Enzyme:**	Trypsin: cuts C-term side of KR unless next residue is P.
**Fixed modifications:**	Carbamidomethyl (C)
**Variable modifications:**	Deamidated (NQ), Oxidation (M)

Protein sequence coverage: 5%

Matched peptides shown in ***bold red***.

```
  1 MKKKLLTFLC LMATVVLAAC GFKKVDAGNY LKTSFSGVDT KGRITYQFNT
 51 EELITAFLVE NPKADAKTES ELKAAIAEVK ISPSKTENLS NDEEVTLTFA
101 NTKNLEKFVT IPSEKKVKVT GLTAVKKLNS EELAKLVSLE ATGFNKKGKA
151 KVRINDPRVA SIRFVVENDG QLENGKDAKI KIDGNFDKVL ESNGYILEGD
201 GSFTLPVKGL KTVADKFEDA KNKDEVVKKL KEEINKKYTD ATITFDKTYY
251 RGLSSGVGES GYGDGLIEGN GNLVMLVRVE YKYAGKRTLA IGLSNLVTNA
301 EGNIELKDAQ LVDKYFDDFA TAAQKLEAIG YTEVK
```

Unformatted sequence string: 335 residues (for pasting into other applications).

Sort by	residue number	increasing mass	decreasing mass
Show	matched peptides only	predicted peptides also	

Query	Start – End	Observed	Mr(expt)	Mr(calc)	ppm	M	Score	Expect	Rank	U	Peptide
14300	86 – 103	1013.4840	2024.9534	2024.9538	-0.19	0	59	4.1e-006	1	U	K.TENLSNDEEVTLTFANTK.N
14301	86 – 103	1013.4847	2024.9548	2024.9538	0.47	0	81	1.8e-008	1	U	K.TENLSNDEEVTLTFANTK.N

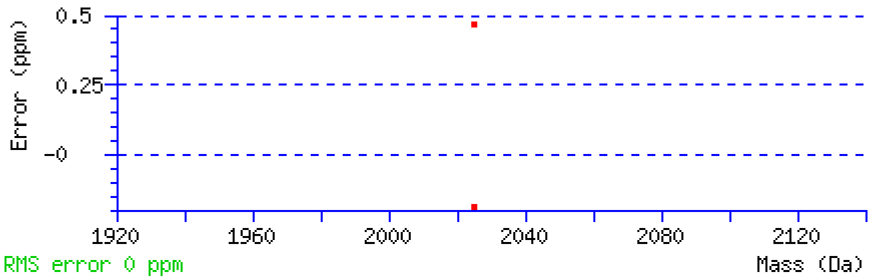

Mascot: http://www.matrixscience.com/

MATRIX SCIENCE Mascot Search Results

Peptide View

MS/MS Fragmentation of **TENLSNDEEVTLTFANTK**
Found in **WP_005606374.1** in **UB_target**, hypothetical protein [Granulicatella]

Match to Query 14301: 2024.954780 from(1013.484666,2+) index(15360)
Title: Elution from: 89.582 to 89.582 period: 0 experiment: 1 cycles: 1 precIntensity: 147802.0 FinneganScanNumber: 19591 MStype: enumIsNormalMS
rawFile: 19B022_Kuweit_Sample-GA-EVS.raw
Data file LTQ_19B022_Kuweit_Sample-GA-EVS.mgf

Click mouse within plot area to zoom in by factor of two about that point
Or, Plot from 100 to 2000 Da Full range
Show Y-axis

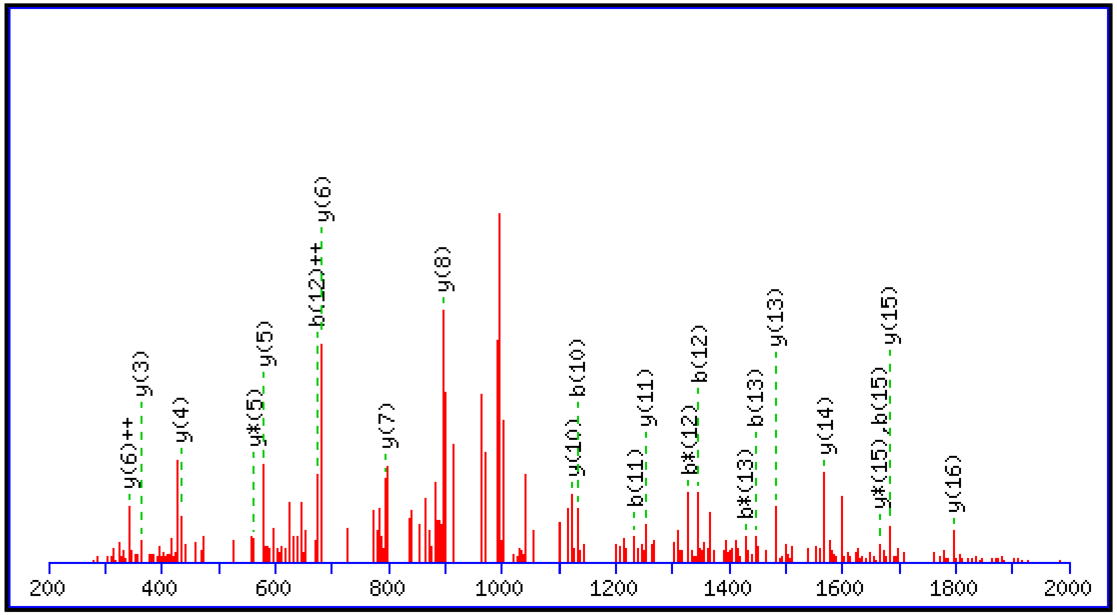

Label all possible matches Label matches used for scoring

Monoisotopic mass of neutral peptide Mr(calc): 2024.9538
Fixed modifications: Carbamidomethyl (C) (apply to specified residues or termini only)
Ions Score: 81 **Expect:** 1.8e-008

Mascot Search Results: Peptide View

Matches : 23/198 fragment ions using 35 most intense peaks (help)

#	b	b^{++}	b*	b*$^{++}$	b^{0}	b^{0++}	Seq.	y	y^{++}	y*	y*$^{++}$	y^{0}	y^{0++}	#
1	102.0550	51.5311			84.0444	42.5258	T							18
2	231.0975	116.0524			213.0870	107.0471	E	1924.9134	962.9604	1907.8869	954.4471	1906.9029	953.9551	17
3	345.1405	173.0739	328.1139	164.5606	327.1299	164.0686	N	***1795.8708***	898.4391	1778.8443	889.9258	1777.8603	889.4338	16
4	458.2245	229.6159	441.1980	221.1026	440.2140	220.6106	L	***1681.8279***	841.4176	1664.8014	832.9043	1663.8174	832.4123	15
5	545.2566	273.1319	528.2300	264.6186	527.2460	264.1266	S	***1568.7439***	784.8756	1551.7173	776.3623	1550.7333	775.8703	14
6	659.2995	330.1534	642.2729	321.6401	641.2889	321.1481	N	***1481.7118***	741.3596	1464.6853	732.8463	1463.7013	732.3543	13
7	774.3264	387.6669	757.2999	379.1536	756.3159	378.6616	D	1367.6689	684.3381	1350.6424	675.8248	1349.6583	675.3328	12
8	903.3690	452.1882	886.3425	443.6749	885.3585	443.1829	E	***1252.6420***	626.8246	1235.6154	618.3113	1234.6314	617.8193	11
9	1032.4116	516.7094	1015.3851	508.1962	1014.4011	507.7042	E	***1123.5994***	562.3033	1106.5728	553.7900	1105.5888	553.2980	10
10	**1131.4800**	566.2437	1114.4535	557.7304	1113.4695	557.2384	V	994.5568	497.7820	977.5302	489.2688	976.5462	488.7767	9
11	**1232.5277**	616.7675	1215.5012	608.2542	1214.5172	607.7622	T	***895.4884***	448.2478	878.4618	439.7345	877.4778	439.2425	8
12	**1345.6118**	673.3095	1328.5852	664.7963	1327.6012	664.3042	L	***794.4407***	397.7240	777.4141	389.2107	776.4301	388.7187	7
13	**1446.6595**	723.8334	1429.6329	715.3201	1428.6489	714.8281	T	***681.3566***	341.1819	664.3301	332.6687	663.3461	332.1767	6
14	1593.7279	797.3676	1576.7013	788.8543	1575.7173	788.3623	F	***580.3089***	290.6581	563.2824	282.1448	562.2984	281.6528	5
15	**1664.7650**	832.8861	1647.7384	824.3729	1646.7544	823.8808	A	***433.2405***	217.1239	416.2140	208.6106	415.2300	208.1186	4
16	1778.8079	889.9076	1761.7814	881.3943	1760.7973	880.9023	N	***362.2034***	181.6053	345.1769	173.0921	344.1928	172.6001	3
17	1879.8556	940.4314	1862.8290	931.9182	1861.8450	931.4262	T	248.1605	124.5839	231.1339	116.0706	230.1499	115.5786	2
18							K	147.1128	74.0600	130.0863	65.5468			1

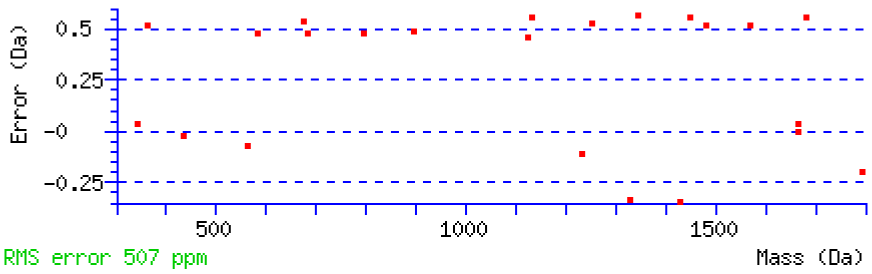

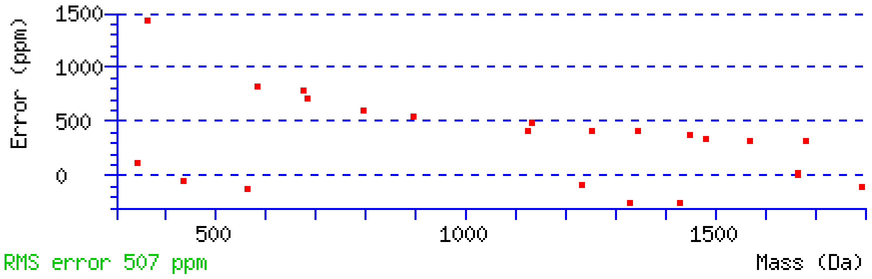

NCBI **BLAST** search of TENLSNDEEVTLTFANTK
(Parameters: blastp, nr protein database, expect=20000, no filter, PAM30)
Other BLAST web gateways

All matches to this query

Score	Mr(calc)	Delta	Sequence
81.5	2024.9538	0.0010	TENLSNDEEVTLTFANTK

Mascot: http://www.matrixscience.com/

MASCOT Search Results

Protein View: WP_005605842.1

hypothetical protein [Granulicatella adiacens]

**Database:**	UB_target
**Score:**	107
**Monoisotopic mass (M_r):**	49485
**Calculated pI:**	5.48

Sequence similarity is available as an NCBI BLAST search of WP_005605842.1 against nr.

Search parameters

**MS data file:**	LTQ_19B022_Kuweit_Sample-GA-EVS.mgf
**Enzyme:**	Trypsin: cuts C-term side of KR unless next residue is P.
**Fixed modifications:**	Carbamidomethyl (C)
**Variable modifications:**	Deamidated (NQ), Oxidation (M)

Protein sequence coverage: 4%

Matched peptides shown in ***bold red***.

```
  1 MFRHYKKLFL AFVALFSVFV LASCSQDQSN SQSAAQTEAP KVETIDGDWE
 51 LVDAVDALSY SIGAYTLKAI NFARLLDSVK DFKMDMKIEN NTATIKYDYN
101 IDNFIKAFYT FSKKAEGKTE EEYKKLLYDS HEEFAGEFKK YKVSMNKETG
151 VYSYEATGSI DQDAKTMTFD EGITVTNSFF FSFGENRVSP NTYHYELKDD
201 MLYITIDGKG PKDNLPVHYE LHFKRKGSTT QKDPVPIEGK WQAIDFRPAL
251 QRSLAYKDFK NDDSAIKLIY PEALKDLKPT LNITGTSVEF DYTVSLTEGF
301 GMFYDYLKQK DAAKVTQTKD EYIKNQFIRL STTLQGAAKD YPNTTYEFDK
351 DNNTIHSVLK NGKLDTANQT IVFPEAINIV QLAIMSIGPV NKETTYKYSI
401 DGDILTLTIE QRDGKNNLNS VISAKFKKVS DATSN
```

Unformatted sequence string: 435 residues (for pasting into other applications).

Sort by	residue number	increasing mass	decreasing mass
Show	matched peptides only	predicted peptides also	

Query	Start – End	Observed	Mr(expt)	Mr(calc)	ppm	M	Score	Expect	Rank	U	Peptide

13432	148 - 165	967.4367	1932.8589	1932.8589	0.018	0	77	3.2e-008	1	U	K.ETGVYSYEATGSIDQDAK.T
13433	148 - 165	967.4385	1932.8624	1932.8589	1.85	0	57	3.1e-006	1	U	K.ETGVYSYEATGSIDQDAK.T

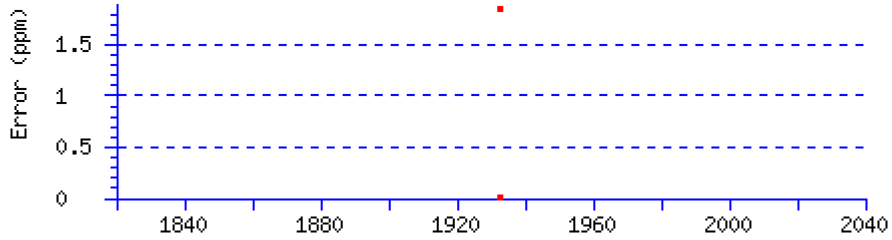

RMS error 1 ppm

Mascot: http://www.matrixscience.com/

MATRIX SCIENCE Mascot Search Results

Peptide View

MS/MS Fragmentation of **ETGVYSYEATGSIDQDAK**
Found in **WP_005605842.1** in **UB_target**, hypothetical protein [Granulicatella adiacens]

Match to Query 13432: 1932.858906 from(967.436729,2+) index(7499)
Title: Elution from: 58.837 to 58.837 period: 0 experiment: 1 cycles: 1 precIntensity: 265348.0 FinneganScanNumber: 10236 MStype: enumIsNormalMS rawFile: 19B022_Kuweit_Sample-GA-EVS.raw
Data file LTQ_19B022_Kuweit_Sample-GA-EVS.mgf

Click mouse within plot area to zoom in by factor of two about that point

Or, Plot from 100 to 2000 Da Full range

Show Y-axis ☐

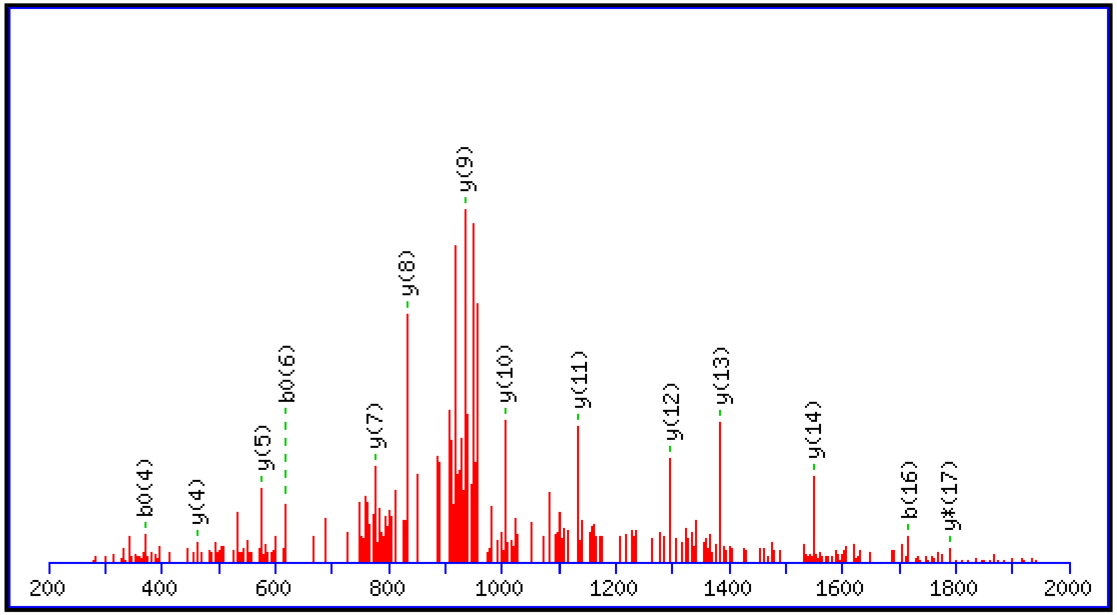

Label all possible matches   Label matches used for scoring

Monoisotopic mass of neutral peptide Mr(calc): 1932.8589
Fixed modifications: Carbamidomethyl (C) (apply to specified residues or termini only)
Ions Score: 77 **Expect:** 3.2e-008

Matches : 14/172 fragment ions using 17 most intense peaks (help)

#	b	b^{++}	b^{*}	b^{*++}	b^{0}	b^{0++}	Seq.	y	y^{++}	y^{*}	y^{*++}	y^{0}	y^{0++}	#
1	130.0499	65.5286			112.0393	56.5233	E							18
2	231.0975	116.0524			213.0870	107.0471	T	1804.8236	902.9154	1787.7970	894.4021	1786.8130	893.9101	17
3	288.1190	144.5631			270.1084	135.5579	G	1703.7759	852.3916	1686.7493	843.8783	1685.7653	843.3863	16
4	387.1874	194.0974			369.1769	185.0921	V	1646.7544	823.8808	1629.7279	815.3676	1628.7439	814.8756	15
5	550.2508	275.6290			532.2402	266.6237	Y	***1547.6860***	774.3466	1530.6595	765.8334	1529.6754	765.3414	14
6	637.2828	319.1450			619.2722	310.1397	S	***1384.6227***	692.8150	1367.5961	684.3017	1366.6121	683.8097	13
7	800.3461	400.6767			782.3355	391.6714	Y	***1297.5907***	649.2990	1280.5641	640.7857	1279.5801	640.2937	12
8	929.3887	465.1980			911.3781	456.1927	E	***1134.5273***	567.7673	1117.5008	559.2540	1116.5168	558.7620	11
9	1000.4258	500.7165			982.4153	491.7113	A	***1005.4847***	503.2460	988.4582	494.7327	987.4742	494.2407	10
10	1101.4735	551.2404			1083.4629	542.2351	T	***934.4476***	467.7274	917.4211	459.2142	916.4371	458.7222	9
11	1158.4950	579.7511			1140.4844	570.7458	G	***833.3999***	417.2036	816.3734	408.6903	815.3894	408.1983	8
12	1245.5270	623.2671			1227.5164	614.2619	S	***776.3785***	388.6929	759.3519	380.1796	758.3679	379.6876	7
13	1358.6111	679.8092			1340.6005	670.8039	I	689.3464	345.1769	672.3199	336.6636	671.3359	336.1716	6
14	1473.6380	737.3226			1455.6274	728.3174	D	***576.2624***	288.6348	559.2358	280.1216	558.2518	279.6295	5
15	1601.6966	801.3519	1584.6700	792.8387	1583.6860	792.3466	Q	***461.2354***	231.1214	444.2089	222.6081	443.2249	222.1161	4
16	1716.7235	858.8654	1699.6970	850.3521	1698.7130	849.8601	D	333.1769	167.0921	316.1503	158.5788	315.1663	158.0868	3
17	1787.7606	894.3840	1770.7341	885.8707	1769.7501	885.3787	A	218.1499	109.5786	201.1234	101.0653			2
18							K	147.1128	74.0600	130.0863	65.5468			1

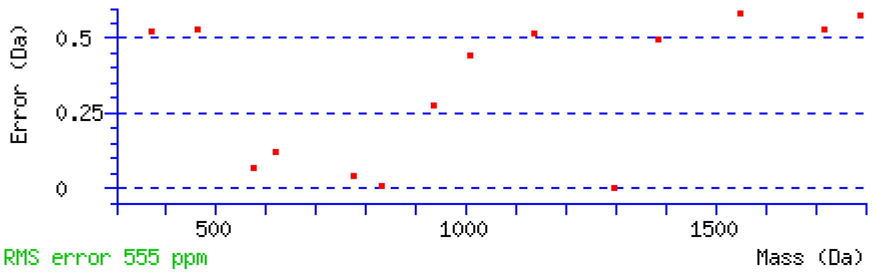

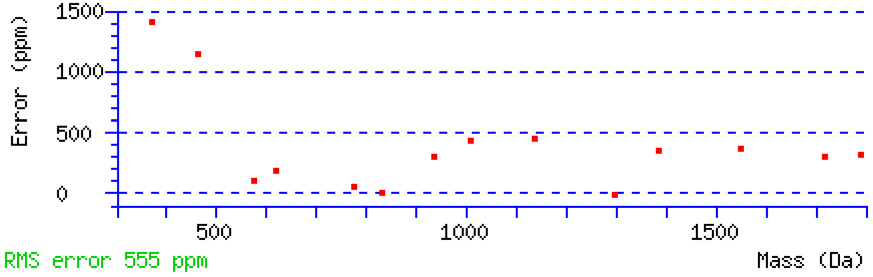

NCBI **BLAST** search of ETGVYSYEATGSIDQDAK
(Parameters: blastp, nr protein database, expect=20000, no filter, PAM30)
Other BLAST web gateways

All matches to this query

Score	Mr(calc)	Delta	Sequence
76.9	1932.8589	0.0000	ETGVYSYEATGSIDQDAK

Mascot: http://www.matrixscience.com/

MASCOT Search Results

Protein View: RKW29369.1

hypothetical protein [Granulicatella sp.]

**Database:**	UB_target
**Score:**	104
**Monoisotopic mass (M_r):**	27154
**Calculated pI:**	8.33

Sequence similarity is available as an NCBI BLAST search of RKW29369.1 against nr.

Search parameters

**MS data file:**	LTQ_19B022_Kuweit_Sample-GA-EVS.mgf
**Enzyme:**	Trypsin: cuts C-term side of KR unless next residue is P.
**Fixed modifications:**	Carbamidomethyl (C)
**Variable modifications:**	Deamidated (NQ), Oxidation (M)

Protein sequence coverage: 8%

Matched peptides shown in ***bold red***.

```
  1 MIRQTKKLFL GLLSLLAVFV IAGCGQNQAS DSNANNAQKS QQEQKDPNNL
 51 AGEWESVYEL DSLQKAFFPK GMKSYTFAKF IEAFKDFKMK LSVDGTTAKL
101 SYQYDSKKFA KAFYEISRDK KEMTEDAFVS RYINGQVDFV KNFKKYKASM
151 DTSTGTYSYE ATGTVDEKAK TVTFDEGIII LDSFPLTTAD KDHRFDSVTY
201 NYEVKDGILT IYADMKTKDN LPVHFELNFK RVPSAEKK
```

Unformatted sequence string: 238 residues (for pasting into other applications).

Sort by	residue number	increasing mass	decreasing mass
Show	matched peptides only	predicted peptides also	

Query	Start – End	Observed	Mr(expt)	Mr(calc)	ppm	M	Score	Expect	Rank	U	Peptide
16318	148 – 168	1107.4733	2212.9321	2212.9318	0.15	0	104	4.4e-011	1	U	K.ASMDTSTGTYSYEATGTVDEK.A

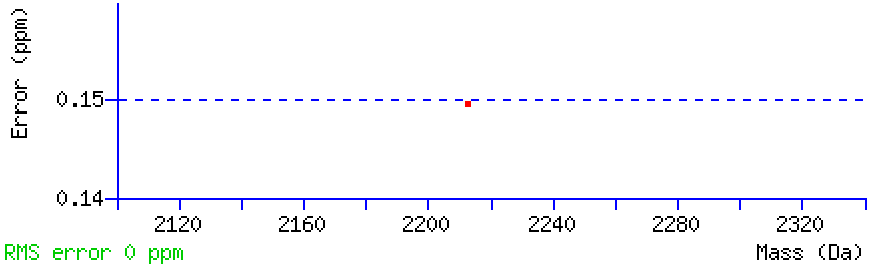

Mascot: http://www.matrixscience.com/

Mascot Search Results

Peptide View

MS/MS Fragmentation of **ASMDTSTGTYSYEATGTVDEK**
Found in **RKW29369.1** in **UB_target**, hypothetical protein [Granulicatella sp.]

Match to Query 16318: 2212.932124 from(1107.473338,2+) index(6036)
Title: Elution from: 53.242 to 53.242 period: 0 experiment: 1 cycles: 1 precIntensity: 534500.0 FinneganScanNumber: 8514 MStype: enumIsNormalMS rawFile: 19B022_Kuweit_Sample-GA-EVS.raw
Data file LTQ_19B022_Kuweit_Sample-GA-EVS.mgf

Click mouse within plot area to zoom in by factor of two about that point
Or, Plot from 150 to 1100 Da Full range
Show Y-axis

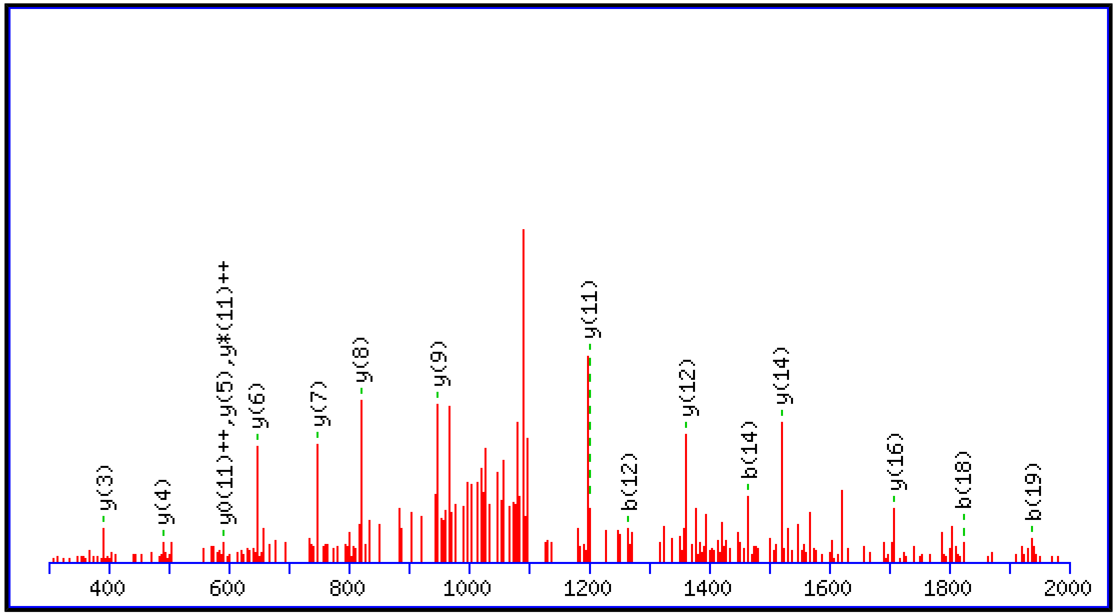

Label all possible matches Label matches used for scoring

Monoisotopic mass of neutral peptide Mr(calc): 2212.9318
Fixed modifications: Carbamidomethyl (C) (apply to specified residues or termini only)
Ions Score: 104 **Expect:** 4.4e-011

Matches : 17/196 fragment ions using 17 most intense peaks (help)

#	b	b^{++}	b^{0}	b^{0++}	Seq.	y	y^{++}	y*	y^{*++}	y^{0}	y^{0++}	#
1	72.0444	36.5258			A							21
2	159.0764	80.0418	141.0659	71.0366	S	2142.9020	1071.9546	2125.8754	1063.4413	2124.8914	1062.9493	20
3	290.1169	145.5621	272.1063	136.5568	M	2055.8699	1028.4386	2038.8434	1019.9253	2037.8594	1019.4333	19
4	405.1438	203.0756	387.1333	194.0703	D	1924.8294	962.9184	1907.8029	954.4051	1906.8189	953.9131	18
5	506.1915	253.5994	488.1810	244.5941	T	1809.8025	905.4049	1792.7759	896.8916	1791.7919	896.3996	17
6	593.2236	297.1154	575.2130	288.1101	S	***1708.7548***	854.8810	1691.7283	846.3678	1690.7443	845.8758	16
7	694.2712	347.6393	676.2607	338.6340	T	1621.7228	811.3650	1604.6962	802.8518	1603.7122	802.3598	15
8	751.2927	376.1500	733.2821	367.1447	G	***1520.6751***	760.8412	1503.6486	752.3279	1502.6645	751.8359	14
9	852.3404	426.6738	834.3298	417.6685	T	1463.6536	732.3305	1446.6271	723.8172	1445.6431	723.3252	13
10	1015.4037	508.2055	997.3931	499.2002	Y	***1362.6060***	681.8066	1345.5794	673.2933	1344.5954	672.8013	12
11	1102.4357	551.7215	1084.4252	542.7162	S	***1199.5426***	600.2750	1182.5161	591.7617	1181.5321	591.2697	11
12	**1265.4991**	633.2532	1247.4885	624.2479	Y	1112.5106	556.7589	1095.4841	548.2457	1094.5000	547.7537	10
13	1394.5417	697.7745	1376.5311	688.7692	E	***949.4473***	475.2273	932.4207	466.7140	931.4367	466.2220	9
14	**1465.5788**	733.2930	1447.5682	724.2877	A	***820.4047***	410.7060	803.3781	402.1927	802.3941	401.7007	8
15	1566.6264	783.8169	1548.6159	774.8116	T	***749.3676***	375.1874	732.3410	366.6742	731.3570	366.1821	7
16	1623.6479	812.3276	1605.6373	803.3223	G	***648.3199***	324.6636	631.2933	316.1503	630.3093	315.6583	6
17	1724.6956	862.8514	1706.6850	853.8462	T	***591.2984***	296.1529	574.2719	287.6396	573.2879	287.1476	5
18	**1823.7640**	912.3856	1805.7534	903.3804	V	***490.2508***	245.6290	473.2242	237.1157	472.2402	236.6237	4
19	**1938.7909**	969.8991	1920.7804	960.8938	D	***391.1823***	196.0948	374.1558	187.5815	373.1718	187.0895	3
20	2067.8335	1034.4204	2049.8230	1025.4151	E	276.1554	138.5813	259.1288	130.0681	258.1448	129.5761	2
21					K	147.1128	74.0600	130.0863	65.5468			1

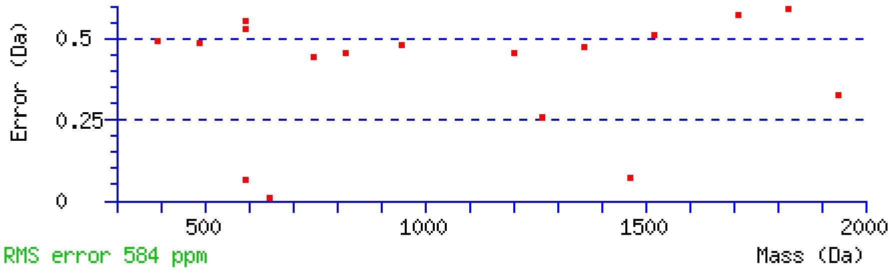

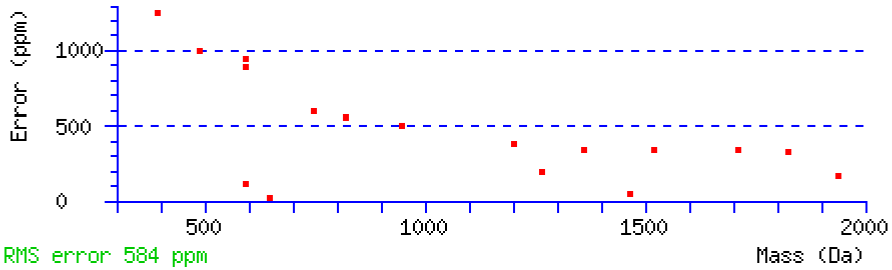

NCBI **BLAST** search of ASMDTSTGTYSYEATGTVDEK
(Parameters: blastp, nr protein database, expect=20000, no filter, PAM30)
Other BLAST web gateways

All matches to this query

Score	Mr(calc)	Delta	Sequence
103.5	2212.9318	0.0003	ASMDTSTGTYSYEATGTVDEK

Mascot: http://www.matrixscience.com/

MASCOT Search Results

Protein View: EEW36930.1

3D domain protein [Granulicatella adiacens ATCC 49175 ATCC 49175]

**Database:**	UB_target
**Score:**	103
**Monoisotopic mass (M_r):**	30173
**Calculated pI:**	5.15

Sequence similarity is available as an NCBI BLAST search of EEW36930.1 against nr.

Search parameters

**MS data file:**	LTQ_19B022_Kuweit_Sample-GA-EVS.mgf
**Enzyme:**	Trypsin: cuts C-term side of KR unless next residue is P.
**Fixed modifications:**	Carbamidomethyl (C)
**Variable modifications:**	Deamidated (NQ), Oxidation (M)

Protein sequence coverage: 12%

Matched peptides shown in ***bold red***.

```
  1 MRTRFLNSIK ESKVGKENMK PIKKQLMMVT ASIALFGYAG FTANSASANE
 51 VEWTARTVEQ VKQDVKKDDK GVQEYTIKWG DTLSVISEAT GASLDSLVQV
101 NEIQNANLIY PGTVLRFSAD QKEVTVNNGS QEHSYRVQDN KEVKEVEKSE
151 ATTSASNETA QATQATETTQ AAQTTQAASS SQKGYYLTVE ATAYSYNEAG
201 LSSYTADGTN LVNEPNVIAV DPSVIPLGSY VEIPGYGIFR AADTGGAIYG
251 NRIDVHLVNL NDVYNFGRRT ITIRVLQ
```

Unformatted sequence string: 277 residues (for pasting into other applications).

Sort by	residue number	increasing mass	decreasing mass
Show	matched peptides only	predicted peptides also	

Query	Start – End	Observed	Mr(expt)	Mr(calc)	ppm	M	Score	Expect	Rank	U	Peptide
21269	149 – 183	1163.1995	3486.5766	3486.5830	-1.84	0	56	2.8e-006	1	U	K.SEATTSASNETAQATQATETTQAAQTTQAASSSQK.G
21270	149 – 183	1163.1995	3486.5767	3486.5830	-1.80	0	28	0.0015	1	U	K.SEATTSASNETAQATQATETTQAAQTTQAASSSQK.G
21271	149 – 183	1163.2003	3486.5790	3486.5830	-1.15	0	39	0.00012	1	U	K.SEATTSASNETAQATQATETTQAAQTTQAASSSQK.G
21272	149 – 183	1163.2015	3486.5827	3486.5830	-0.092	0	52	8.4e-006	1	U	K.SEATTSASNETAQATQATETTQAAQTTQAASSSQK.G

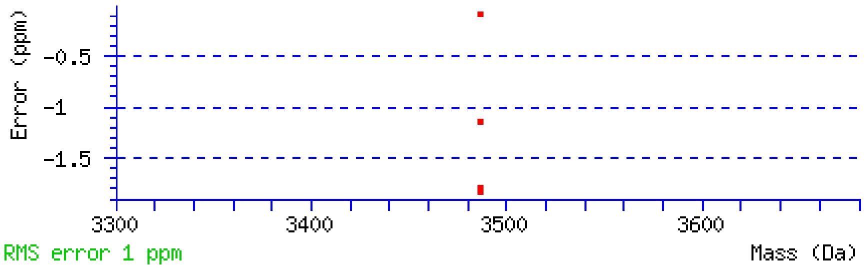

Mascot: http://www.matrixscience.com/

MATRIX SCIENCE Mascot Search Results

Peptide View

MS/MS Fragmentation of **SEATTSASNETAQATQATETTQAAQTTQAASSSQK**
Found in **EEW36930.1** in **UB_target**, 3D domain protein [Granulicatella adiacens ATCC 49175 ATCC 49175]

Match to Query 21269: 3486.576573 from(1163.199467,3+) index(2709)
Title: Elution from: 40.982 to 40.982 period: 0 experiment: 1 cycles: 1 precIntensity: 193855.0 FinneganScanNumber: 4744 MStype: enumIsNormalMS rawFile: 19B022_Kuweit_Sample-GA-EVS.raw
Data file LTQ_19B022_Kuweit_Sample-GA-EVS.mgf

Click mouse within plot area to zoom in by factor of two about that point
Or, Plot from 150 to 1100 Da Full range
Show Y-axis ☐

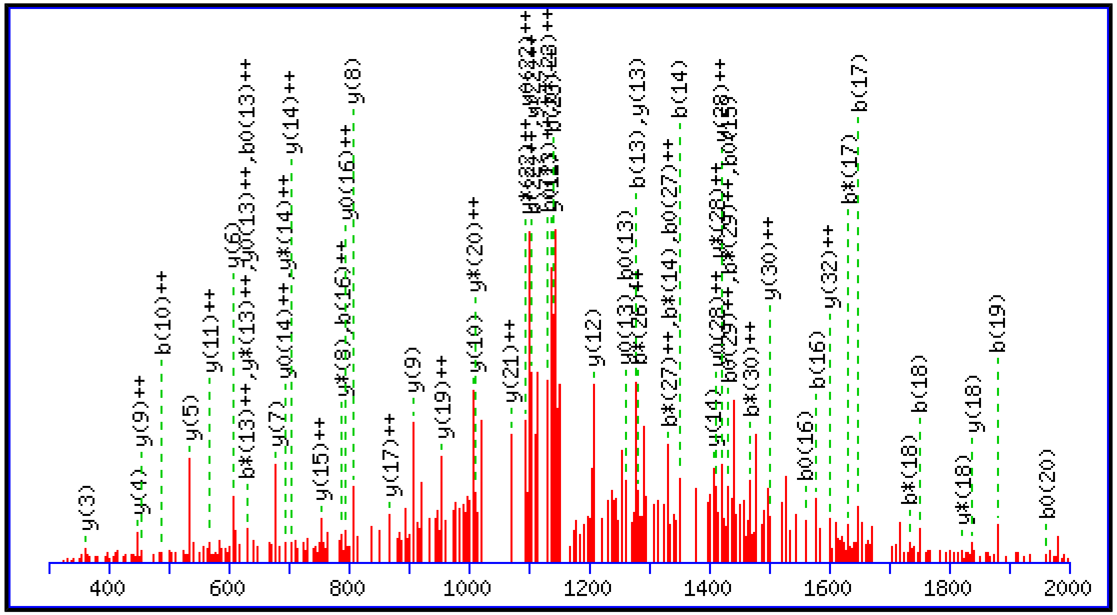

Label all possible matches   Label matches used for scoring

Monoisotopic mass of neutral peptide Mr(calc): 3486.5830
Fixed modifications: Carbamidomethyl (C) (apply to specified residues or termini only)
Ions Score: 56 **Expect:** 2.8e-006

Matches : 67/388 fragment ions using 102 most intense peaks (help)

#	b	b^{++}	b*	b^{*++}	b^0	b^{0++}	Seq.	y	y^{++}	y*	y^{*++}	y^0	y^{0++}	#
1	88.0393	44.5233			70.0287	35.5180	S							35
2	217.0819	109.0446			199.0713	100.0393	E	3400.5583	1700.7828	3383.5317	1692.2695	3382.5477	1691.7775	34
3	288.1190	144.5631			270.1084	135.5579	A	3271.5157	1636.2615	3254.4891	1627.7482	3253.5051	1627.2562	33
4	389.1667	195.0870			371.1561	186.0817	T	3200.4785	1600.7429	3183.4520	1592.2296	3182.4680	1591.7376	32
5	490.2144	245.6108			472.2038	236.6055	T	3099.4309	1550.2191	3082.4043	1541.7058	3081.4203	1541.2138	31
6	577.2464	289.1268			559.2358	280.1216	S	2998.3832	1499.6952	2981.3566	1491.1820	2980.3726	1490.6900	30
7	648.2835	324.6454			630.2729	315.6401	A	2911.3512	1456.1792	2894.3246	1447.6659	2893.3406	1447.1739	29
8	735.3155	368.1614			717.3050	359.1561	S	2840.3140	1420.6607	2823.2875	1412.1474	2822.3035	1411.6554	28
9	849.3585	425.1829	832.3319	416.6696	831.3479	416.1776	N	2753.2820	1377.1446	2736.2555	1368.6314	2735.2715	1368.1394	27
10	978.4011	489.7042	961.3745	481.1909	960.3905	480.6989	E	2639.2391	1320.1232	2622.2125	1311.6099	2621.2285	1311.1179	26
11	1079.4487	540.2280	1062.4222	531.7147	1061.4382	531.2227	T	2510.1965	1255.6019	2493.1700	1247.0886	2492.1859	1246.5966	25
12	1150.4859	575.7466	1133.4593	567.2333	1132.4753	566.7413	A	2409.1488	1205.0780	2392.1223	1196.5648	2391.1383	1196.0728	24
13	1278.5444	639.7759	1261.5179	631.2626	1260.5339	630.7706	Q	2338.1117	1169.5595	2321.0852	1161.0462	2320.1011	1160.5542	23
14	1349.5815	675.2944	1332.5550	666.7811	1331.5710	666.2891	A	2210.0531	1105.5302	2193.0266	1097.0169	2192.0426	1096.5249	22
15	1450.6292	725.8182	1433.6027	717.3050	1432.6187	716.8130	T	2139.0160	1070.0116	2121.9895	1061.4984	2121.0055	1061.0064	21
16	1578.6878	789.8475	1561.6613	781.3343	1560.6772	780.8423	Q	2037.9683	1019.4878	2020.9418	1010.9745	2019.9578	1010.4825	20
17	1649.7249	825.3661	1632.6984	816.8528	1631.7144	816.3608	A	1909.9098	955.4585	1892.8832	946.9452	1891.8992	946.4532	19
18	1750.7726	875.8899	1733.7460	867.3767	1732.7620	866.8847	T	1838.8726	919.9400	1821.8461	911.4267	1820.8621	910.9347	18
19	1879.8152	940.4112	1862.7886	931.8980	1861.8046	931.4059	E	1737.8250	869.4161	1720.7984	860.9028	1719.8144	860.4108	17
20	1980.8629	990.9351	1963.8363	982.4218	1962.8523	981.9298	T	1608.7824	804.8948	1591.7558	796.3815	1590.7718	795.8895	16
21	2081.9105	1041.4589	2064.8840	1032.9456	2063.9000	1032.4536	T	1507.7347	754.3710	1490.7081	745.8577	1489.7241	745.3657	15
22	2209.9691	1105.4882	2192.9426	1096.9749	2191.9586	1096.4829	Q	1406.6870	703.8471	1389.6605	695.3339	1388.6764	694.8419	14
23	2281.0062	1141.0068	2263.9797	1132.4935	2262.9957	1132.0015	A	1278.6284	639.8179	1261.6019	631.3046	1260.6179	630.8126	13
24	2352.0434	1176.5253	2335.0168	1168.0120	2334.0328	1167.5200	A	1207.5913	604.2993	1190.5648	595.7860	1189.5808	595.2940	12
25	2480.1019	1240.5546	2463.0754	1232.0413	2462.0914	1231.5493	Q	1136.5542	568.7807	1119.5277	560.2675	1118.5436	559.7755	11
26	2581.1496	1291.0784	2564.1231	1282.5652	2563.1390	1282.0732	T	1008.4956	504.7515	991.4691	496.2382	990.4851	495.7462	10
27	2682.1973	1341.6023	2665.1707	1333.0890	2664.1867	1332.5970	T	907.4480	454.2276	890.4214	445.7143	889.4374	445.2223	9
28	2810.2559	1405.6316	2793.2293	1397.1183	2792.2453	1396.6263	Q	806.4003	403.7038	789.3737	395.1905	788.3897	394.6985	8

**29**	2881.2930	1441.1501	2864.2664	1432.6369	2863.2824	1432.1448	**A**	***678.3417***	339.6745	661.3151	331.1612	660.3311	330.6692	**7**
**30**	2952.3301	1476.6687	2935.3035	1468.1554	2934.3195	1467.6634	**A**	***607.3046***	304.1559	590.2780	295.6427	589.2940	295.1506	**6**
**31**	3039.3621	1520.1847	3022.3356	1511.6714	3021.3516	1511.1794	**S**	***536.2675***	268.6374	519.2409	260.1241	518.2569	259.6321	**5**
**32**	3126.3941	1563.7007	3109.3676	1555.1874	3108.3836	1554.6954	**S**	***449.2354***	225.1214	432.2089	216.6081	431.2249	216.1161	**4**
**33**	3213.4262	1607.2167	3196.3996	1598.7035	3195.4156	1598.2114	**S**	***362.2034***	181.6053	345.1769	173.0921	344.1928	172.6001	**3**
**34**	3341.4848	1671.2460	3324.4582	1662.7327	3323.4742	1662.2407	**Q**	275.1714	138.0893	258.1448	129.5761			**2**
**35**							**K**	147.1128	74.0600	130.0863	65.5468			**1**

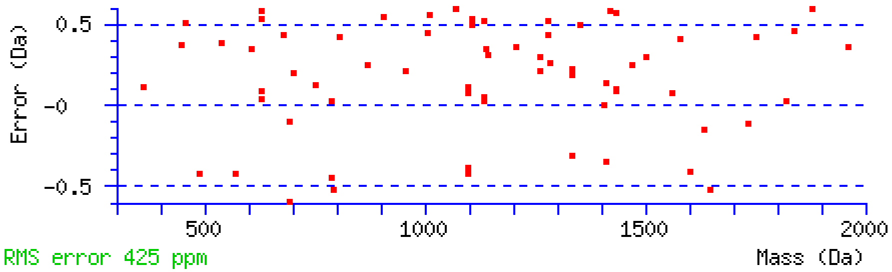

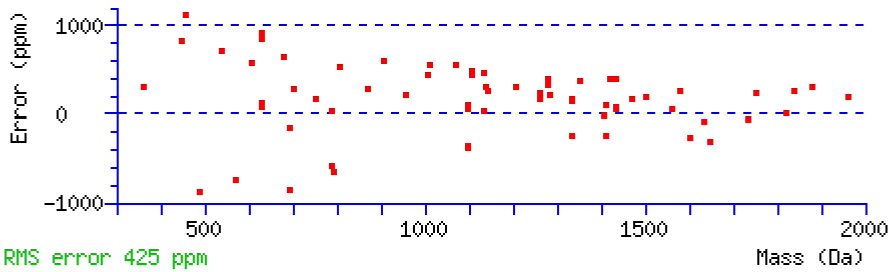

NCBI **BLAST** search of SEATTSASNETAQATQATETTQAAQTTQAASSSQK
(Parameters: blastp, nr protein database, expect=20000, no filter, PAM30)
Other BLAST web gateways

All matches to this query

Score	Mr(calc)	Delta	Sequence
56.0	3486.5830	-0.0064	SEATTSASNETAQATQATETTQAAQTTQAASSSQK

Mascot: http://www.matrixscience.com/

MASCOT Search Results

Protein View: RKW29737.1

PBP1A family penicillin-binding protein [Granulicatella sp.]

**Database:**	UB_target
**Score:**	97
**Monoisotopic mass (M_r):**	91688
**Calculated pI:**	6.30

Sequence similarity is available as an NCBI BLAST search of RKW29737.1 against nr.

Search parameters

**MS data file:**	LTQ_19B022_Kuweit_Sample-GA-EVS.mgf
**Enzyme:**	Trypsin: cuts C-term side of KR unless next residue is P.
**Fixed modifications:**	Carbamidomethyl (C)
**Variable modifications:**	Deamidated (NQ), Oxidation (M)

Protein sequence coverage: 2%

Matched peptides shown in ***bold red***.

```
  1 MTENNTGQSR SSQHRQPKKK ASSPSNKKKI LKKVLIGLGA FIGVALIAII
 51 AIFAYYGSTA PEIKASDLQG ATETKIYDKD GELISSLGGE KRDVITSDQV
101 PQLLKDAVTS IEDKRFYSHM GIDPIRILGS FFRNAKAGQI TQGGSTITQQ
151 LIKLSVFSTK KEDQTYQRKI QEAILALKLE REFSKEQILT FYLNKVYMAN
201 SVYGFGTASH YYFNKELSEL TLPQVALLAG MPQAPNSYDP YAHPEEAKER
251 RDTVLYTMKT NGKITNEQYE QALATPINDG LIAHNNNVDS SDKALVYDSF
301 VTMVLKEVQD KTGLDPYNDG LVIETTIDSK AQQKLNDIVN TNDYINYVND
351 KIQSASVMLD SKTGAVRAVS GGRKQTTLFA YNRATDNQRS TGSTIKPIID
401 YGPAIEYLNY STGQTLLDQK TTYSNGVELN NWDFRHNGPM TLRRALVYSR
451 NTTALEAFKA VGETNIKSFL NNLDIQIKND GQDYLVESNA IGAEISPIKM
501 AAAYATFSNA GTYSKPYTVT KITTRDGQVY EFKPEQKQAM KDSTAYMITN
551 VLKDSFTYGF ATEVAIPGLS TAAKTGSSNY TIEQKRAMGA SDYEDIIPDS
601 WFIGYSPDYT ISVWTGYDNP YEKGGGVDTT EQGYAKLIYY HLMKYMAQYS
651 SGEDWVQPDS VVQQQIEVGS IPLSLPGPRT PANMIATELF VKGSTPTQQS
    SNYGVTIEGP TGLKATYNKE KKELTVTWDR YNNTGQGTPQ FKVTANGQSQ
```

701

751 TVTGNSVKFQ NITGPSVSVS IVVTVGRNSS DPITNEFQIE QPTTTQEQTT

801 RQSETTTENN RRNDSNNEQT TQERRNR

Unformatted sequence string: 827 residues (for pasting into other applications).

Sort by residue number increasing mass decreasing mass
Show matched peptides only predicted peptides also

Query	Start – End	Observed	Mr(expt)	Mr(calc)	ppm	M	Score	Expect	Rank	U	Peptide
11449	137 – 153	872.4823	1742.9501	1742.9527	-1.46	0	54	9.5e-006	1	U	K.AGQITQGGSTITQQLIK.L
11450	137 – 153	872.4837	1742.9528	1742.9527	0.091	0	71	2e-007	1	U	K.AGQITQGGSTITQQLIK.L

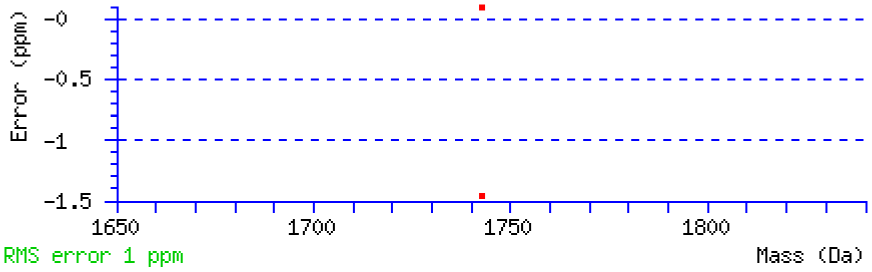

Mascot: http://www.matrixscience.com/

Mascot Search Results

Peptide View

MS/MS Fragmentation of **AGQITQGGSTITQQLIK**
Found in **RKW29737.1** in **UB_target**, PBP1A family penicillin-binding protein [Granulicatella sp.]

Match to Query 11450: 1742.952826 from(872.483689,2+) index(12563)
Title: Elution from: 78.780 to 78.780 period: 0 experiment: 1 cycles: 1 precIntensity: 187744.0 FinneganScanNumber: 16286 MStype: enumIsNormalMS rawFile: 19B022_Kuweit_Sample-GA-EVS.raw
Data file LTQ_19B022_Kuweit_Sample-GA-EVS.mgf

Click mouse within plot area to zoom in by factor of two about that point
Or, Plot from 150 to 1100 Da Full range
Show Y-axis

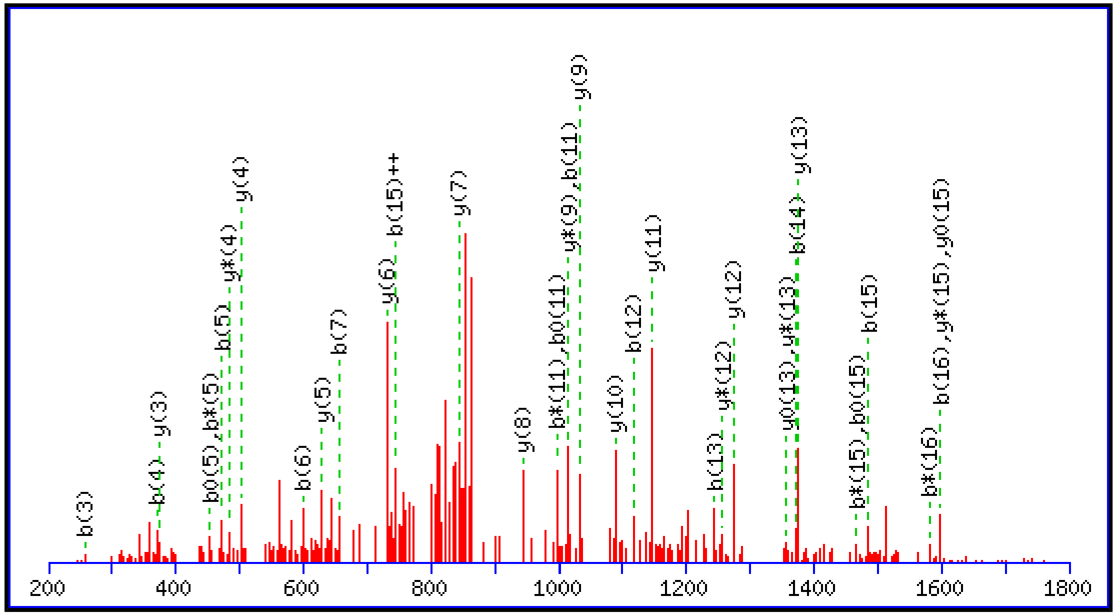

Label all possible matches Label matches used for scoring

Monoisotopic mass of neutral peptide Mr(calc): 1742.9527
Fixed modifications: Carbamidomethyl (C) (apply to specified residues or termini only)
Ions Score: 71 **Expect:** 2e-007

Matches : 37/170 fragment ions using 60 most intense peaks (help)

#	b	b++	b*	b*++	b0	b0++	Seq.	y	y++	y*	y*++	y0	y0++	#
1	72.0444	36.5258					A							17
2	129.0659	65.0366					G	1672.9228	836.9651	1655.8963	828.4518	1654.9123	827.9598	16
3	257.1244	129.0659	240.0979	120.5526			Q	1615.9014	808.4543	1598.8748	799.9410	1597.8908	799.4490	15
4	370.2085	185.6079	353.1819	177.0946			I	1487.8428	744.4250	1470.8162	735.9118	1469.8322	735.4197	14
5	471.2562	236.1317	454.2296	227.6185	453.2456	227.1264	T	1374.7587	687.8830	1357.7322	679.3697	1356.7482	678.8777	13
6	599.3148	300.1610	582.2882	291.6477	581.3042	291.1557	Q	1273.7110	637.3592	1256.6845	628.8459	1255.7005	628.3539	12
7	656.3362	328.6717	639.3097	320.1585	638.3257	319.6665	G	1145.6525	573.3299	1128.6259	564.8166	1127.6419	564.3246	11
8	713.3577	357.1825	696.3311	348.6692	695.3471	348.1772	G	1088.6310	544.8191	1071.6045	536.3059	1070.6204	535.8139	10
9	800.3897	400.6985	783.3632	392.1852	782.3791	391.6932	S	1031.6095	516.3084	1014.5830	507.7951	1013.5990	507.3031	9
10	901.4374	451.2223	884.4108	442.7091	883.4268	442.2170	T	944.5775	472.7924	927.5510	464.2791	926.5669	463.7871	8
11	1014.5215	507.7644	997.4949	499.2511	996.5109	498.7591	I	843.5298	422.2686	826.5033	413.7553	825.5193	413.2633	7
12	1115.5691	558.2882	1098.5426	549.7749	1097.5586	549.2829	T	730.4458	365.7265	713.4192	357.2132	712.4352	356.7212	6
13	1243.6277	622.3175	1226.6012	613.8042	1225.6171	613.3122	Q	629.3981	315.2027	612.3715	306.6894			5
14	1371.6863	686.3468	1354.6597	677.8335	1353.6757	677.3415	Q	501.3395	251.1734	484.3130	242.6601			4
15	1484.7704	742.8888	1467.7438	734.3755	1466.7598	733.8835	L	373.2809	187.1441	356.2544	178.6308			3
16	1597.8544	799.4308	1580.8279	790.9176	1579.8439	790.4256	I	260.1969	130.6021	243.1703	122.0888			2
17							K	147.1128	74.0600	130.0863	65.5468			1

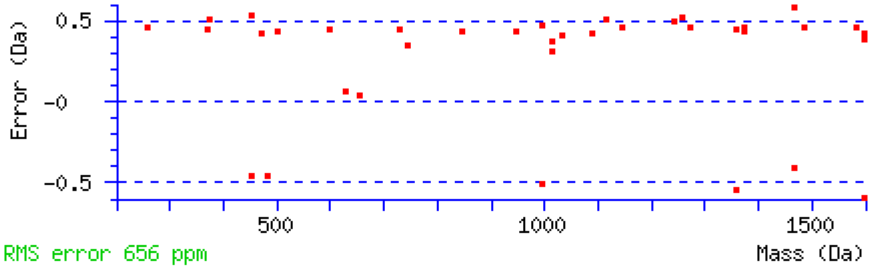

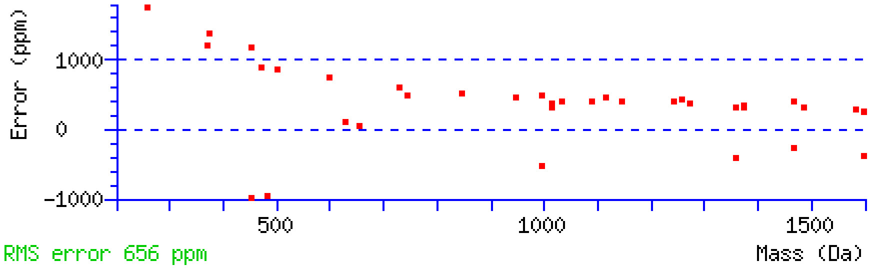

NCBI **BLAST** search of AGQITQGGSTITQQLIK
(Parameters: blastp, nr protein database, expect=20000, no filter, PAM30)
Other BLAST web gateways

All matches to this query

Score	Mr(calc)	Delta	Sequence
71.4	1742.9527	0.0002	AGQITQGGSTITQQLIK

Mascot: http://www.matrixscience.com/

MATRIX SCIENCE MASCOT Search Results

Protein View: RKW25989.1

foldase [Granulicatella sp.]

**Database:**	UB_target
**Score:**	92
**Monoisotopic mass (M_r):**	34574
**Calculated pI:**	5.24

Sequence similarity is available as an NCBI BLAST search of RKW25989.1 against nr.

Search parameters

**MS data file:**	LTQ_19B022_Kuweit_Sample-GA-EVS.mgf
**Enzyme:**	Trypsin: cuts C-term side of KR unless next residue is P.
**Fixed modifications:**	Carbamidomethyl (C)
**Variable modifications:**	Deamidated (NQ), Oxidation (M)

Protein sequence coverage: 3%

Matched peptides shown in ***bold red***.

```
  1 MKKKFTGMLV LASTLALAAC SNNTNSETIA TTKYGNVTRE EFVNAMKDTV
 51 GEQTLQRLVL TKVLEGSVED SKKLKEDAEQ EVAKLVAQYG GENGMLAALK
101 QSGIASVDAY RQTIYLNKLM TAAVKKAAAF TDEDIKKYYD EWEPQIKVQH
151 ILIAAKATAS DEEKAAAKAK AEELIQKLKD GADFSELAKE NSADTGTASK
201 GGEIGPFKRS DMVKEFSEAS YNLKNVGDIT ETPVETQFGY HIIKMLDKGE
251 KKPFDEVKSQ MEEEMLQAKL KDSAYLHQTM VDLLKGADVK ISDESLQNAL
301 KNFLDAADST TTSSK
```

Unformatted sequence string: 315 residues (for pasting into other applications).

Sort by	residue number	increasing mass	decreasing mass
Show	matched peptides only	predicted peptides also	

Query	Start – End	Observed	Mr(expt)	Mr(calc)	ppm	M	Score	Expect	Rank	U	Peptide
6906	291 – 301	609.3217	1216.6289	1216.6299	-0.85	0	50	4.7e-005	1	U	K.ISDESLQNALK.N
6907	291 – 301	609.3217	1216.6289	1216.6299	-0.79	0	38	0.00089	1	U	K.ISDESLQNALK.N

6908	291 – 301	609.3225	1216.6305	1216.6299	0.48	0	52	3.5e-005	1	U	K.ISDESLQNALK.N
6909	291 – 301	609.3225	1216.6305	1216.6299	0.48	0	47	0.00011	1	U	K.ISDESLQNALK.N

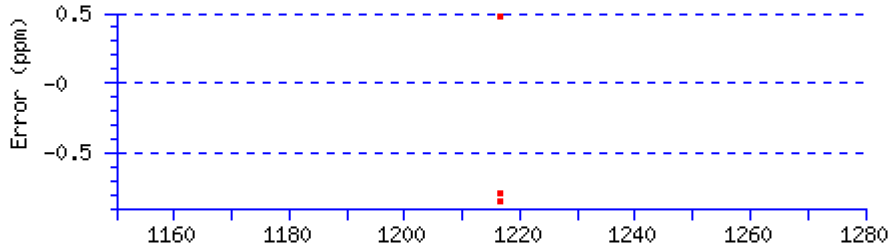

RMS error 0 ppm

Mascot: http://www.matrixscience.com/

Mascot Search Results

Peptide View

MS/MS Fragmentation of **ISDESLQNALK**
Found in **RKW25989.1** in **UB_target**, foldase [Granulicatella sp.]

Match to Query 6908: 1216.630476 from(609.322514,2+) index(9510)
Title: Elution from: 66.872 to 66.872 period: 0 experiment: 1 cycles: 1 precIntensity: 339405.0 FinneganScanNumber: 12668 MStype: enumIsNormalMS rawFile: 19B022_Kuweit_Sample-GA-EVS.raw
Data file LTQ_19B022_Kuweit_Sample-GA-EVS.mgf

Click mouse within plot area to zoom in by factor of two about that point
Or, Plot from 100 to 1400 Da Full range
Show Y-axis

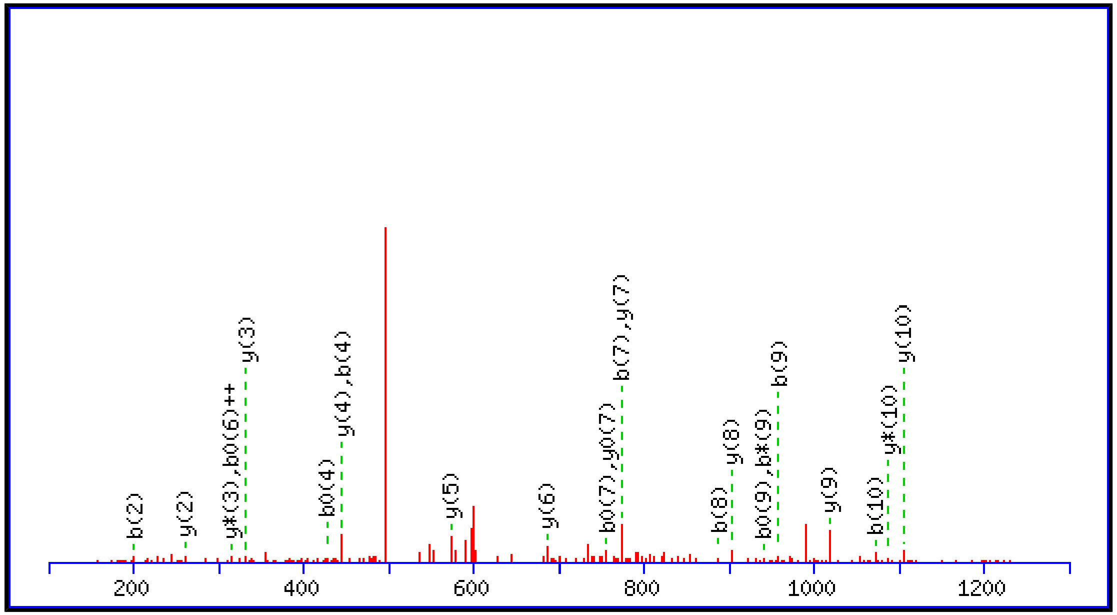

Label all possible matches Label matches used for scoring

Monoisotopic mass of neutral peptide Mr(calc): 1216.6299
Fixed modifications: Carbamidomethyl (C) (apply to specified residues or termini only)
Ions Score: 52 **Expect:** 3.5e-005

Matches : 23/94 fragment ions using 44 most intense peaks (help)

#	b	b^{++}	b*	b*$^{++}$	b^{0}	b^{0++}	Seq.	y	y^{++}	y*	y*$^{++}$	y^{0}	y^{0++}	#
1	114.0913	57.5493					I							11
2	**201.1234**	101.0653			183.1128	92.0600	S	***1104.5531***	552.7802	1087.5266	544.2669	1086.5426	543.7749	10
3	316.1503	158.5788			298.1397	149.5735	D	***1017.5211***	509.2642	1000.4946	500.7509	999.5105	500.2589	9
4	**445.1929**	223.1001			427.1823	214.0948	E	***902.4942***	451.7507	885.4676	443.2374	884.4836	442.7454	8
5	532.2249	266.6161			514.2144	257.6108	S	***773.4516***	387.2294	756.4250	378.7162	755.4410	378.2241	7
6	645.3090	323.1581			627.2984	314.1529	L	***686.4196***	343.7134	669.3930	335.2001			6
7	**773.3676**	387.1874	756.3410	378.6742	755.3570	378.1821	Q	***573.3355***	287.1714	556.3089	278.6581			5
8	**887.4105**	444.2089	870.3840	435.6956	869.3999	435.2036	N	***445.2769***	223.1421	428.2504	214.6288			4
9	**958.4476**	479.7274	941.4211	471.2142	940.4371	470.7222	A	***331.2340***	166.1206	314.2074	157.6074			3
10	**1071.5317**	536.2695	1054.5051	527.7562	1053.5211	527.2642	L	***260.1969***	130.6021	243.1703	122.0888			2
11							K	147.1128	74.0600	130.0863	65.5468			1

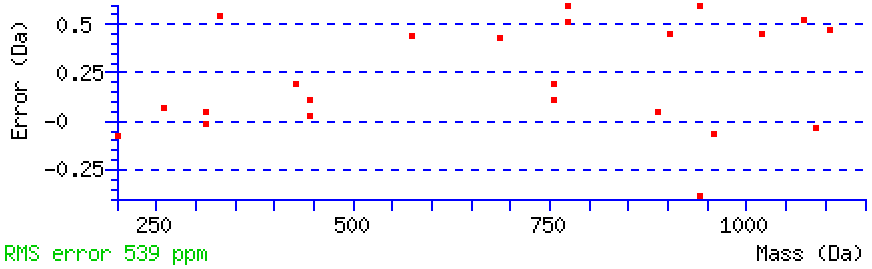

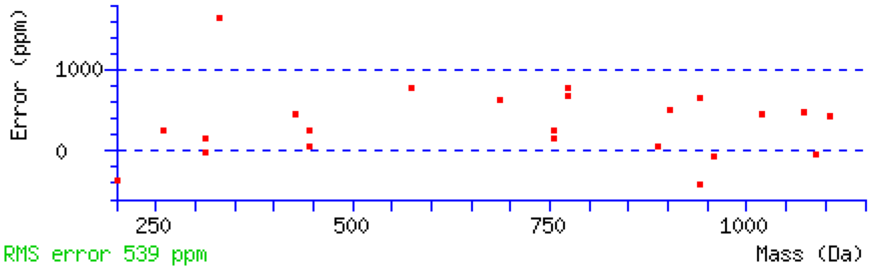

NCBI **BLAST** search of ISDESLQNALK
(Parameters: blastp, nr protein database, expect=20000, no filter, PAM30)
Other BLAST web gateways

All matches to this query

Score	Mr(calc)	Delta	Sequence
52.1	1216.6299	0.0006	ISDESLQNALK
15.8	1216.6299	0.0006	DEAEKAAKNLK
5.3	1216.6299	0.0006	LSENDQKIAAK
4.0	1216.6299	0.0006	LSENDQKIAAK

2.6	1216.6299	0.0006	DLQKVEEQTK
2.2	1216.6299	0.0006	LDQVASESVIR
2.0	1216.6299	0.0006	DLSDKEEILR
2.0	1216.6299	0.0006	DLSLDERIEK
1.9	1216.6299	0.0006	EDAKKAIDEAK
1.8	1216.6299	0.0006	EVTLEDRLDK

Mascot: http://www.matrixscience.com/

MASCOT Search Results

Protein View: RKW26849.1

purine-nucleoside phosphorylase [Granulicatella sp.]

**Database:**	UB_target
**Score:**	92
**Monoisotopic mass (M_r):**	29847
**Calculated pI:**	5.20

Sequence similarity is available as an NCBI BLAST search of RKW26849.1 against nr.

Search parameters

**MS data file:**	LTQ_19B022_Kuweit_Sample-GA-EVS.mgf
**Enzyme:**	Trypsin: cuts C-term side of KR unless next residue is P.
**Fixed modifications:**	Carbamidomethyl (C)
**Variable modifications:**	Deamidated (NQ), Oxidation (M)

Protein sequence coverage: 7%

Matched peptides shown in ***bold red***.

```
  1 MSKYEQLVET KTFLQEKGIG HIDFGMILGS GLGELAGEVK NPLIFDYKDI
 51 PNFPVSTVVG HAGRLVYGEL EGKQVLIMDG RFHYYEGYDM ETVTFPIRLM
101 KLLDVATIIV TNSAGGANPT FEPGDLMIIT DQINYTGTNP LIGPNDDRFG
151 PRFPDMSHAY HEYGQEVVRK AAKELNINIK EGVYMGYSGP TYETPAEIRF
201 TQAVGGDAVG MSTVPEVIVA NHAGIKVIGI SCITNLAAGM QANLNHEEVV
251 ETTQRVKEVF KSLVRKVLVL Y
```

Unformatted sequence string: 271 residues (for pasting into other applications).

Sort by	residue number	increasing mass	decreasing mass
Show	matched peptides only	predicted peptides also	

Query	Start – End	Observed	Mr(expt)	Mr(calc)	ppm	M	Score	Expect	Rank	U	Peptide
15385	181 – 199	1060.4859	2118.9572	2118.9568	0.19	0	70	1.7e-007	1	U	K.EGVYMGYSGPTYETPAEIR.F
15386	181 – 199	1060.4865	2118.9584	2118.9568	0.75	0	49	3.2e-005	1	U	K.EGVYMGYSGPTYETPAEIR.F

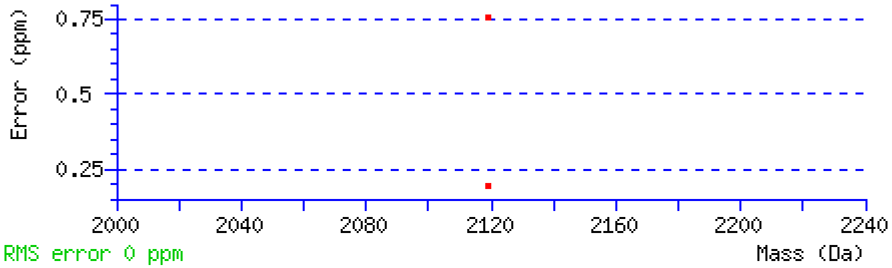

Mascot: http://www.matrixscience.com/

MATRIX SCIENCE Mascot Search Results

Peptide View

MS/MS Fragmentation of **EGVYMGYSGPTYETPAEIR**
Found in **RKW26849.1** in **UB_target**, purine-nucleoside phosphorylase [Granulicatella sp.]

Match to Query 15385: 2118.957210 from(1060.485881,2+) index(16056)
Title: Elution from: 92.617 to 92.617 period: 0 experiment: 1 cycles: 1 precIntensity: 339322.0 FinneganScanNumber: 20479 MStype: enumIsNormalMS rawFile: 19B022_Kuweit_Sample-GA-EVS.raw
Data file LTQ_19B022_Kuweit_Sample-GA-EVS.mgf

Click mouse within plot area to zoom in by factor of two about that point
Or, Plot from 150 to 1100 Da Full range
Show Y-axis ☐

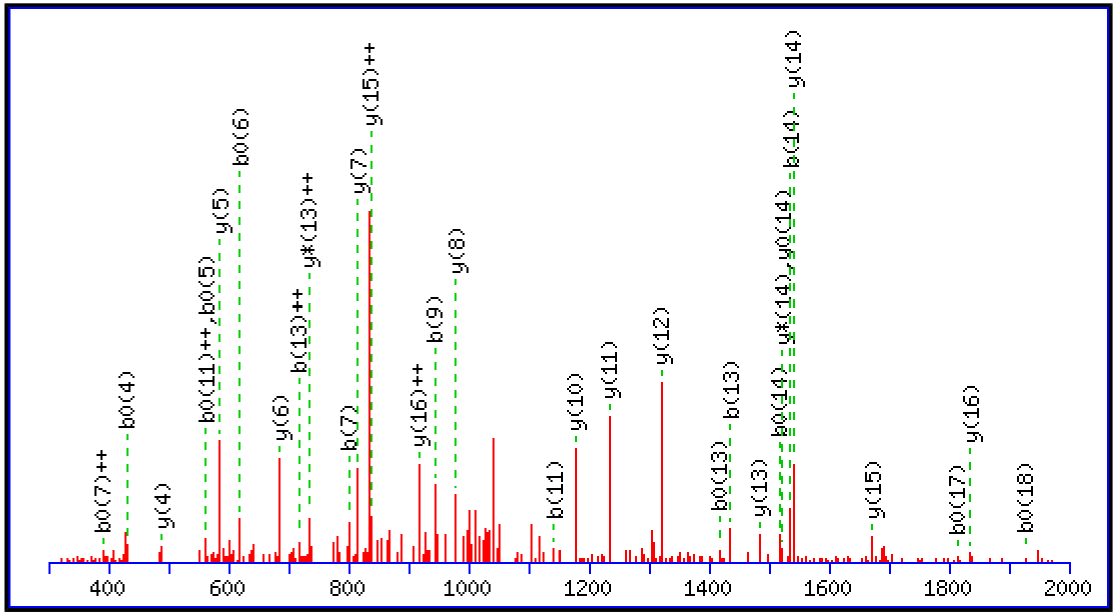

Label all possible matches Label matches used for scoring

Monoisotopic mass of neutral peptide Mr(calc): 2118.9568
Fixed modifications: Carbamidomethyl (C) (apply to specified residues or termini only)
Ions Score: 70 **Expect:** 1.7e-007

Matches : 32/176 fragment ions using 51 most intense peaks (help)

#	b	b^{++}	b^0	b^{0++}	Seq.	y	y^{++}	y*	y^{*++}	y^0	y^{0++}	#
1	130.0499	65.5286	112.0393	56.5233	E							19
2	187.0713	94.0393	169.0608	85.0340	G	1990.9215	995.9644	1973.8950	987.4511	1972.9109	986.9591	18
3	286.1397	143.5735	268.1292	134.5682	V	1933.9000	967.4537	1916.8735	958.9404	1915.8895	958.4484	17
4	449.2031	225.1052	431.1925	216.0999	Y	***1834.8316***	917.9195	1817.8051	909.4062	1816.8211	908.9142	16
5	580.2436	290.6254	562.2330	281.6201	M	***1671.7683***	836.3878	1654.7418	827.8745	1653.7577	827.3825	15
6	637.2650	319.1362	619.2545	310.1309	G	***1540.7278***	770.8675	1523.7013	762.3543	1522.7173	761.8623	14
7	**800.3284**	400.6678	782.3178	391.6625	Y	***1483.7064***	742.3568	1466.6798	733.8435	1465.6958	733.3515	13
8	887.3604	444.1838	869.3498	435.1785	S	***1320.6430***	660.8251	1303.6165	652.3119	1302.6325	651.8199	12
9	**944.3818**	472.6946	926.3713	463.6893	G	***1233.6110***	617.3091	1216.5844	608.7959	1215.6004	608.3039	11
10	1041.4346	521.2209	1023.4240	512.2157	P	***1176.5895***	588.7984	1159.5630	580.2851	1158.5790	579.7931	10
11	**1142.4823**	571.7448	1124.4717	562.7395	T	1079.5368	540.2720	1062.5102	531.7587	1061.5262	531.2667	9
12	1305.5456	653.2764	1287.5351	644.2712	Y	***978.4891***	489.7482	961.4625	481.2349	960.4785	480.7429	8
13	**1434.5882**	717.7977	1416.5776	708.7925	E	***815.4258***	408.2165	798.3992	399.7032	797.4152	399.2112	7
14	**1535.6359**	768.3216	1517.6253	759.3163	T	***686.3832***	343.6952	669.3566	335.1819	668.3726	334.6899	6
15	1632.6887	816.8480	1614.6781	807.8427	P	***585.3355***	293.1714	568.3089	284.6581	567.3249	284.1661	5
16	1703.7258	852.3665	1685.7152	843.3612	A	***488.2827***	244.6450	471.2562	236.1317	470.2722	235.6397	4
17	1832.7684	916.8878	1814.7578	907.8825	E	417.2456	209.1264	400.2191	200.6132	399.2350	200.1212	3
18	1945.8524	973.4299	1927.8419	964.4246	I	288.2030	144.6051	271.1765	136.0919			2
19					R	175.1190	88.0631	158.0924	79.5498			1

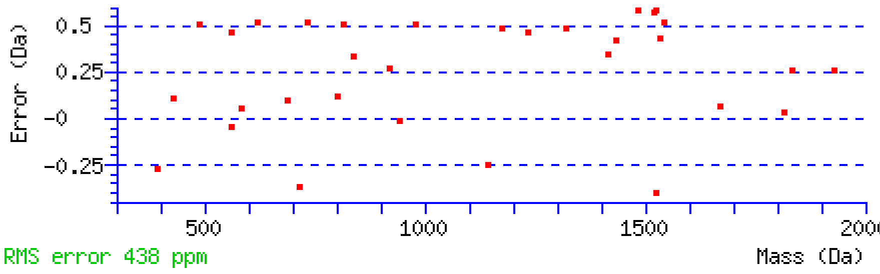

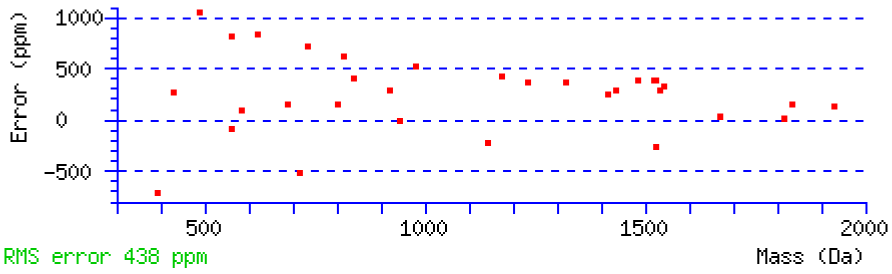

NCBI **BLAST** search of EGVYMGYSGPTYETPAEIR
(Parameters: blastp, nr protein database, expect=20000, no filter, PAM30)

Other BLAST web gateways

All matches to this query

Score	Mr(calc)	Delta	Sequence
70.5	2118.9568	0.0004	EGVYMGYSGPTYETPAEIR

Mascot: http://www.matrixscience.com/

MATRIX SCIENCE MASCOT Search Results

Protein View: RKW26696.1

DUF1307 domain-containing protein [Granulicatella sp.]

**Database:**	UB_target
**Score:**	90
**Monoisotopic mass (M_r):**	20471
**Calculated pI:**	7.68

Sequence similarity is available as an NCBI BLAST search of RKW26696.1 against nr.

Search parameters

**MS data file:**	LTQ_19B022_Kuweit_Sample-GA-EVS.mgf
**Enzyme:**	Trypsin: cuts C-term side of KR unless next residue is P.
**Fixed modifications:**	Carbamidomethyl (C)
**Variable modifications:**	Deamidated (NQ), Oxidation (M)

Protein sequence coverage: 5%

Matched peptides shown in ***bold red***.

```
  1 MNKKSKVLAI LFTTGLVLAG CGQKDNSTTT TTSSTTKETT TTATTTAPTT
 51 TTVATTTAAT DGEKKTVLEQ SDKGVTSRVI MYSKGDVLVK QTTENVYNVK
101 EMETQATEEQ IKTQLETAFA AYKGVEGISS SVELKDGKVI QNFTIDYSKT
151 DFAKLKELVP SFKPKDDNTV SYEVTKNFLV KEGFKVVQ
```

Unformatted sequence string: 188 residues (for pasting into other applications).

Sort by	residue number	increasing mass	decreasing mass
Show	matched peptides only	predicted peptides also	

Query	Start – End	Observed	Mr(expt)	Mr(calc)	ppm	M	Score	Expect	Rank	U	Peptide
8007	139 – 149	664.3479	1326.6813	1326.6820	-0.53	0	57	6.6e-006	1	U	K.VIQNFTIDYSK.T
8008	139 – 149	664.3480	1326.6815	1326.6820	-0.31	0	62	2.2e-006	1	U	K.VIQNFTIDYSK.T

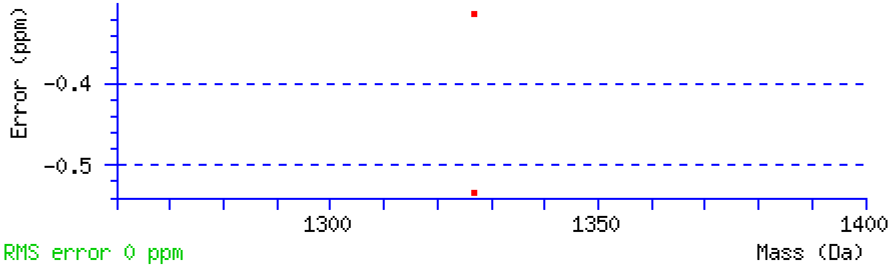

Mascot: http://www.matrixscience.com/

Mascot Search Results

Peptide View

MS/MS Fragmentation of **VIQNFTIDYSK**
Found in **RKW26696.1** in **UB_target**, DUF1307 domain-containing protein [Granulicatella sp.]

Match to Query 8008: 1326.681546 from(664.348049,2+) index(12381)
Title: Elution from: 78.078 to 78.078 period: 0 experiment: 1 cycles: 1 precIntensity: 530248.0 FinneganScanNumber: 16070 MStype: enumIsNormalMS rawFile: 19B022_Kuweit_Sample-GA-EVS.raw
Data file LTQ_19B022_Kuweit_Sample-GA-EVS.mgf

Click mouse within plot area to zoom in by factor of two about that point
Or, Plot from 100 to 1400 Da Full range
Show Y-axis

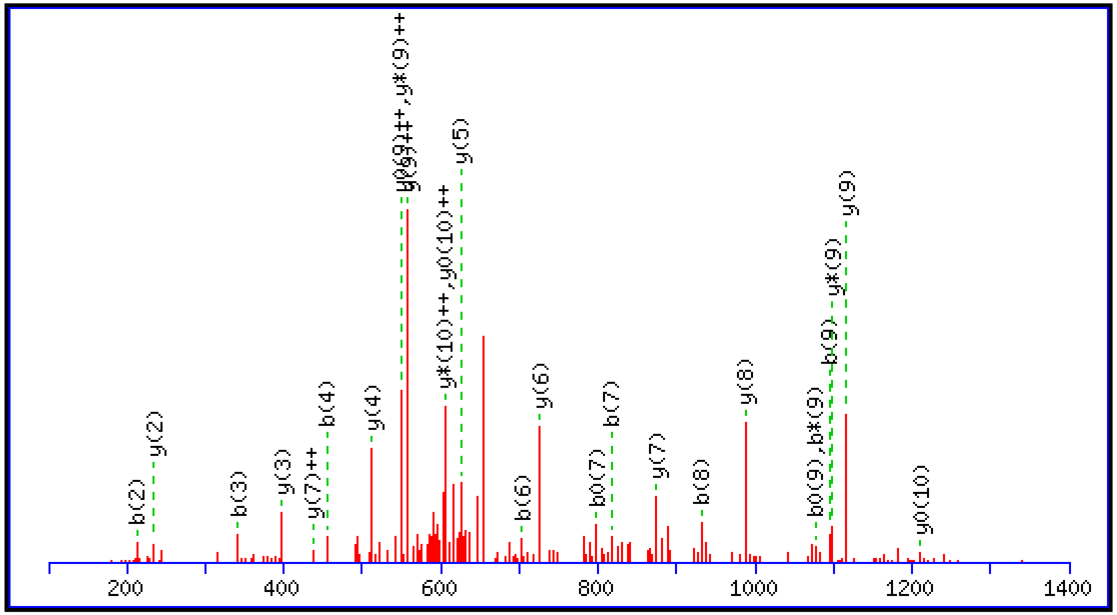

Label all possible matches Label matches used for scoring

Monoisotopic mass of neutral peptide Mr(calc): 1326.6820
Fixed modifications: Carbamidomethyl (C) (apply to specified residues or termini only)
Ions Score: 62 **Expect:** 2.2e-006

Matches : 26/104 fragment ions using 33 most intense peaks (help)

#	b	b^{++}	b*	b^{*++}	b^0	b^{0++}	Seq.	y	y^{++}	y*	y^{*++}	y^0	y^{0++}	#
1	100.0757	50.5415					V							11
2	213.1598	107.0835					I	1228.6208	614.8141	1211.5943	606.3008	1210.6103	605.8088	10
3	341.2183	171.1128	324.1918	162.5995			Q	1115.5368	558.2720	1098.5102	549.7587	1097.5262	549.2667	9
4	455.2613	228.1343	438.2347	219.6210			N	987.4782	494.2427	970.4516	485.7295	969.4676	485.2374	8
5	602.3297	301.6685	585.3031	293.1552			F	873.4353	437.2213	856.4087	428.7080	855.4247	428.2160	7
6	703.3774	352.1923	686.3508	343.6790	685.3668	343.1870	T	726.3668	363.6871	709.3403	355.1738	708.3563	354.6818	6
7	816.4614	408.7343	799.4349	400.2211	798.4509	399.7291	I	625.3192	313.1632	608.2926	304.6499	607.3086	304.1579	5
8	931.4884	466.2478	914.4618	457.7345	913.4778	457.2425	D	512.2351	256.6212	495.2086	248.1079	494.2245	247.6159	4
9	1094.5517	547.7795	1077.5251	539.2662	1076.5411	538.7742	Y	397.2082	199.1077	380.1816	190.5944	379.1976	190.1024	3
10	1181.5837	591.2955	1164.5572	582.7822	1163.5732	582.2902	S	234.1448	117.5761	217.1183	109.0628	216.1343	108.5708	2
11							K	147.1128	74.0600	130.0863	65.5468			1

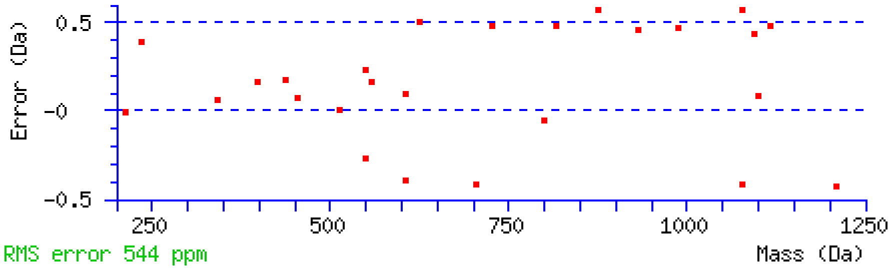

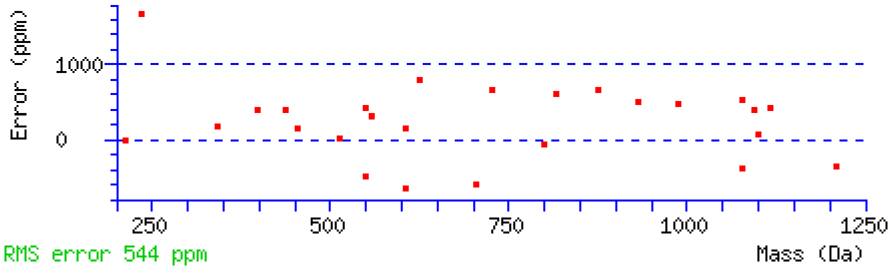

NCBI **BLAST** search of VIQNFTIDYSK
(Parameters: blastp, nr protein database, expect=20000, no filter, PAM30)
Other BLAST web gateways

All matches to this query

Score	Mr(calc)	Delta	Sequence
62.1	1326.6820	-0.0004	VIQNFTIDYSK

Mascot: http://www.matrixscience.com/

MASCOT Search Results

Protein View: EEW37494.1

phosphocarrier protein HPr [Granulicatella adiacens ATCC 49175 ATCC 49175]

**Database:**	UB_target
**Score:**	88
**Monoisotopic mass (M_r):**	11562
**Calculated pI:**	5.36

Sequence similarity is available as an NCBI BLAST search of EEW37494.1 against nr.

Search parameters

**MS data file:**	LTQ_19B022_Kuweit_Sample-GA-EVS.mgf
**Enzyme:**	Trypsin: cuts C-term side of KR unless next residue is P.
**Fixed modifications:**	Carbamidomethyl (C)
**Variable modifications:**	Deamidated (NQ), Oxidation (M)

Protein sequence coverage: 9%

Matched peptides shown in ***bold red***.

```
  1 MLSNVHFLKQ NLLSKETFNM EKKEYHVIAE TGIHARPATL LVQTASKYSS
 51 DIQLEYKGKS VNLKSIMGVM SLGVGQGADV VITAEGADEA EALAGIDETM
101 KKEGLAE
```

Unformatted sequence string: 107 residues (for pasting into other applications).

Sort by	residue number	increasing mass	decreasing mass
Show	matched peptides only	predicted peptides also	

Query	Start – End	Observed	Mr(expt)	Mr(calc)	ppm	M	Score	Expect	Rank	U	Peptide
7180	48 – 57	623.3031	1244.5917	1244.5925	-0.62	0	62	7.4e-007	1	U	K.YSSDIQLEYK.G
7181	48 – 57	623.3031	1244.5917	1244.5925	-0.60	0	51	1.1e-005	1	U	K.YSSDIQLEYK.G

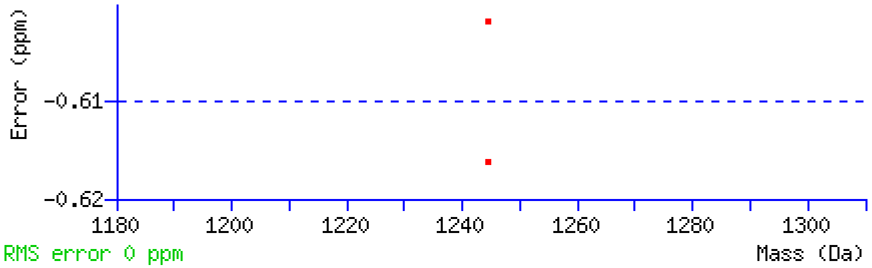

Mascot: http://www.matrixscience.com/

Mascot Search Results

Peptide View

MS/MS Fragmentation of **YSSDIQLEYK**
Found in **EEW37494.1** in **UB_target**, phosphocarrier protein HPr [Granulicatella adiacens ATCC 49175 ATCC 49175]

Match to Query 7180: 1244.591686 from(623.303119,2+) index(8157)
Title: Elution from: 61.285 to 61.285 period: 0 experiment: 1 cycles: 1 precIntensity: 431278.0 FinneganScanNumber: 10991 MStype: enumIsNormalMS rawFile: 19B022_Kuweit_Sample-GA-EVS.raw
Data file LTQ_19B022_Kuweit_Sample-GA-EVS.mgf

Click mouse within plot area to zoom in by factor of two about that point
Or, Plot from 100 to 1400 Da Full range
Show Y-axis

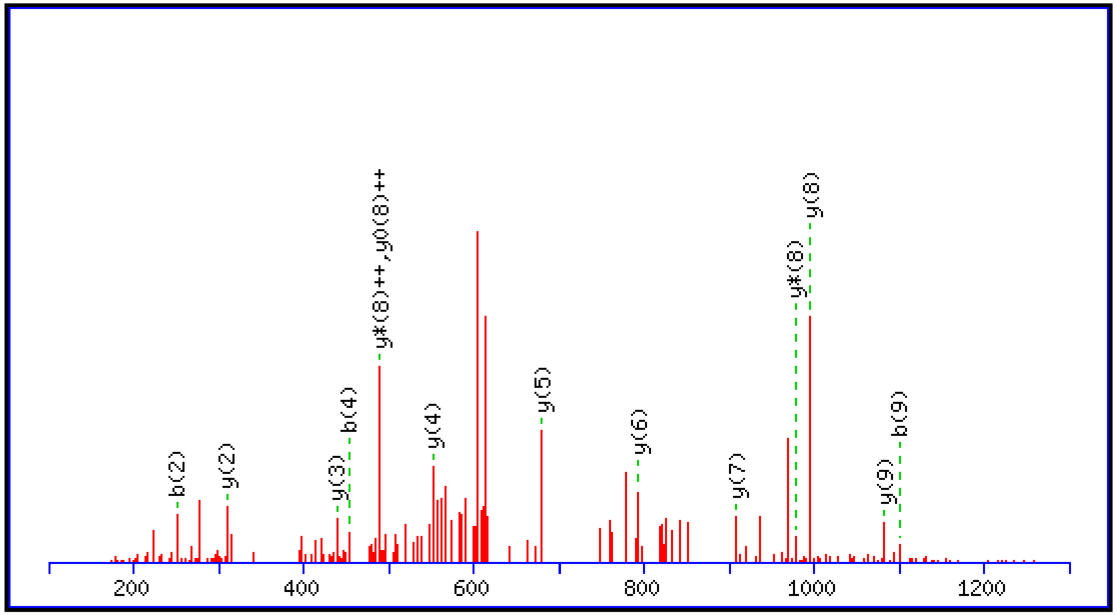

Label all possible matches Label matches used for scoring

Monoisotopic mass of neutral peptide Mr(calc): 1244.5925
Fixed modifications: Carbamidomethyl (C) (apply to specified residues or termini only)
Ions Score: 62 **Expect:** 7.4e-007

Matches : 14/92 fragment ions using 22 most intense peaks (help)

#	b	b^{++}	b*	b^{*++}	b^{0}	b^{0++}	Seq.	y	y^{++}	y*	y^{*++}	y^{0}	y^{0++}	#
**1**	164.0706	82.5389					**Y**							**10**
**2**	**251.1026**	126.0550			233.0921	117.0497	**S**	***1082.5364***	541.7719	1065.5099	533.2586	1064.5259	532.7666	**9**
**3**	338.1347	169.5710			320.1241	160.5657	**S**	***995.5044***	498.2558	978.4779	489.7426	977.4938	489.2506	**8**
**4**	**453.1616**	227.0844			435.1510	218.0792	**D**	***908.4724***	454.7398	891.4458	446.2266	890.4618	445.7345	**7**
**5**	566.2457	283.6265			548.2351	274.6212	**I**	***793.4454***	397.2264	776.4189	388.7131	775.4349	388.2211	**6**
**6**	694.3042	347.6558	677.2777	339.1425	676.2937	338.6505	**Q**	***680.3614***	340.6843	663.3348	332.1710	662.3508	331.6790	**5**
**7**	807.3883	404.1978	790.3618	395.6845	789.3777	395.1925	**L**	***552.3028***	276.6550	535.2762	268.1418	534.2922	267.6498	**4**
**8**	936.4309	468.7191	919.4044	460.2058	918.4203	459.7138	**E**	***439.2187***	220.1130	422.1922	211.5997	421.2082	211.1077	**3**
**9**	**1099.4942**	550.2508	1082.4677	541.7375	1081.4837	541.2455	**Y**	***310.1761***	155.5917	293.1496	147.0784			**2**
**10**							**K**	147.1128	74.0600	130.0863	65.5468			**1**

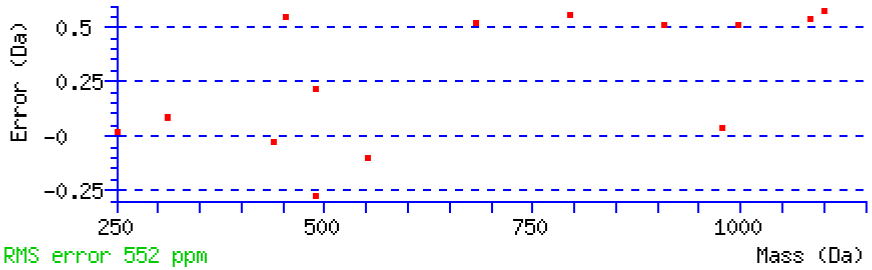

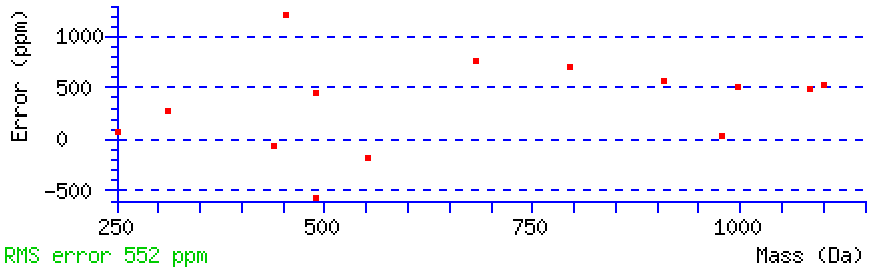

NCBI **BLAST** search of YSSDIQLEYK
(Parameters: blastp, nr protein database, expect=20000, no filter, PAM30)
Other BLAST web gateways

All matches to this query

Score	Mr(calc)	Delta	Sequence
62.3	1244.5925	-0.0008	YSSDIQLEYK
0.3	1244.5884	0.0033	LQQENDALADK

Mascot: http://www.matrixscience.com/

MASCOT Search Results

Protein View: OFT00695.1

L-ribulose-5-phosphate 4-epimerase [Granulicatella sp. HMSC31F03]

**Database:**	UB_target
**Score:**	77
**Monoisotopic mass (M_r):**	33577
**Calculated pI:**	5.18

Sequence similarity is available as an NCBI BLAST search of OFT00695.1 against nr.

Search parameters

**MS data file:**	LTQ_19B022_Kuweit_Sample-GA-EVS.mgf
**Enzyme:**	Trypsin: cuts C-term side of KR unless next residue is P.
**Fixed modifications:**	Carbamidomethyl (C)
**Variable modifications:**	Deamidated (NQ), Oxidation (M)

Protein sequence coverage: 3%

Matched peptides shown in ***bold red***.

```
  1 MERMLTMDNK KFYELGLYEK SMPNTLSFRE KLETVKATGF DFLEISIDET
 51 DEKLSRLEWT KEERQQLVND MFETRVPIRS MCLSGHRKYP FGSHDEAIRA
101 RSLEIMEKAI QLADDLGVRV IQLAGYDVYY EEGDAVTLDY FIQNLKKATE
151 MASQKGILLG FETMETPFMN TVEKSMRFVE LVKSPYLQVY PDSGNLMNAS
201 LEPGAKNVYE DIELGRGHIV AAHLKETIPG HYREIPFGTG QIDFKRMVDT
251 FLSIGVNRFT GEFWYVGQEN WLEDIKFANQ FLRSHFPNK
```

Unformatted sequence string: 289 residues (for pasting into other applications).

Sort by	residue number	increasing mass	decreasing mass
Show	matched peptides only	predicted peptides also	

Query	Start – End	Observed	Mr(expt)	Mr(calc)	ppm	M	Score	Expect	Rank	U	Peptide
6438	109 – 119	585.8273	1169.6400	1169.6404	-0.41	0	57	3.5e-006	1	U	K.AIQLADDLGVR.V
6439	109 – 119	585.8279	1169.6413	1169.6404	0.70	0	38	0.00043	1	U	K.AIQLADDLGVR.V
6440	109 – 119	585.8281	1169.6417	1169.6404	1.07	0	38	0.00042	1	U	K.AIQLADDLGVR.V

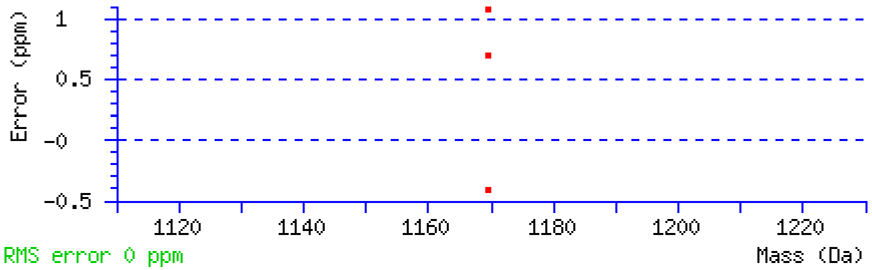

Mascot: http://www.matrixscience.com/

MATRIX SCIENCE Mascot Search Results

Peptide View

MS/MS Fragmentation of **AIQLADDLGVR**
Found in **OFT00695.1** in **UB_target**, L-ribulose-5-phosphate 4-epimerase [Granulicatella sp. HMSC31F03]

Match to Query 6438: 1169.639950 from(585.827251,2+) index(14606)
Title: Elution from: 86.758 to 86.758 period: 0 experiment: 1 cycles: 1 precIntensity: 197258.0 FinneganScanNumber: 18723 MStype: enumIsNormalMS rawFile: 19B022_Kuweit_Sample-GA-EVS.raw
Data file LTQ_19B022_Kuweit_Sample-GA-EVS.mgf

Click mouse within plot area to zoom in by factor of two about that point
Or, Plot from 100 to 1400 Da Full range
Show Y-axis

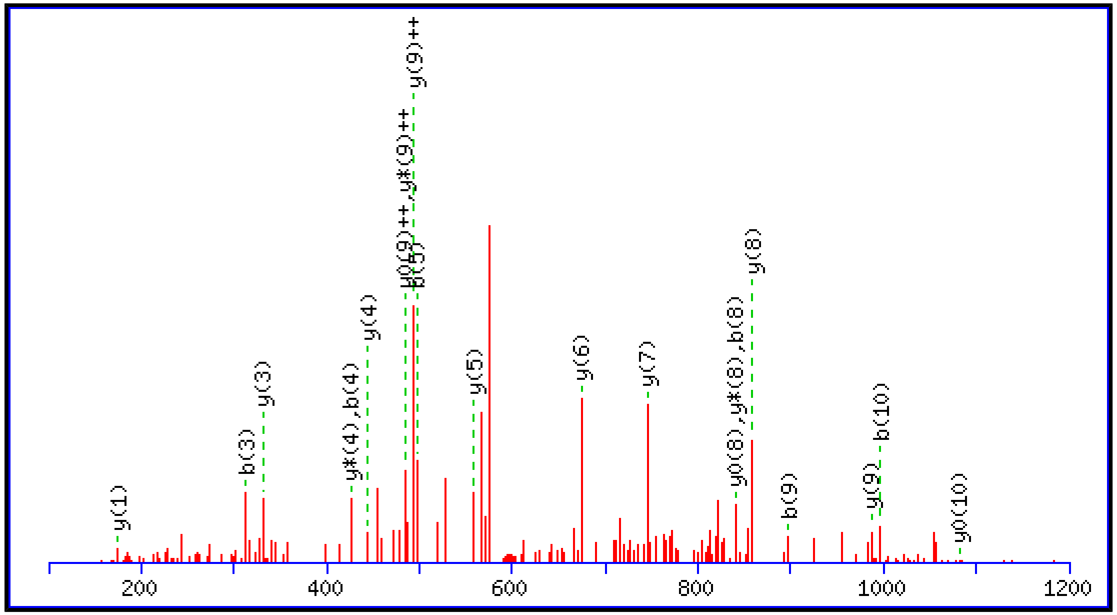

Label all possible matches Label matches used for scoring

Monoisotopic mass of neutral peptide Mr(calc): 1169.6404
Fixed modifications: Carbamidomethyl (C) (apply to specified residues or termini only)
Ions Score: 57 **Expect:** 3.5e-006

Matches : 21/98 fragment ions using 30 most intense peaks (help)

#	b	b^{++}	b*	b^{*++}	b^{0}	b^{0++}	Seq.	y	y^{++}	y*	y^{*++}	y^{0}	y^{0++}	#
1	72.0444	36.5258					A							11
2	185.1285	93.0679					I	1099.6106	550.3089	1082.5841	541.7957	1081.6000	541.3037	10
3	**313.1870**	157.0972	296.1605	148.5839			Q	***986.5265***	493.7669	969.5000	485.2536	968.5160	484.7616	9
4	**426.2711**	213.6392	409.2445	205.1259			L	***858.4680***	429.7376	841.4414	421.2243	840.4574	420.7323	8
5	**497.3082**	249.1577	480.2817	240.6445			A	***745.3839***	373.1956	728.3573	364.6823	727.3733	364.1903	7
6	612.3352	306.6712	595.3086	298.1579	594.3246	297.6659	D	***674.3468***	337.6770	657.3202	329.1638	656.3362	328.6717	6
7	727.3621	364.1847	710.3355	355.6714	709.3515	355.1794	D	***559.3198***	280.1636	542.2933	271.6503	541.3093	271.1583	5
8	**840.4462**	420.7267	823.4196	412.2134	822.4356	411.7214	L	***444.2929***	222.6501	427.2663	214.1368			4
9	**897.4676**	449.2375	880.4411	440.7242	879.4571	440.2322	G	***331.2088***	166.1081	314.1823	157.5948			3
10	**996.5360**	498.7717	979.5095	490.2584	978.5255	489.7664	V	274.1874	137.5973	257.1608	129.0840			2
11							R	***175.1190***	88.0631	158.0924	79.5498			1

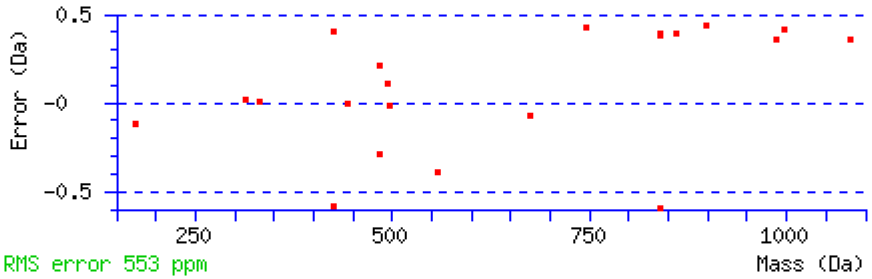

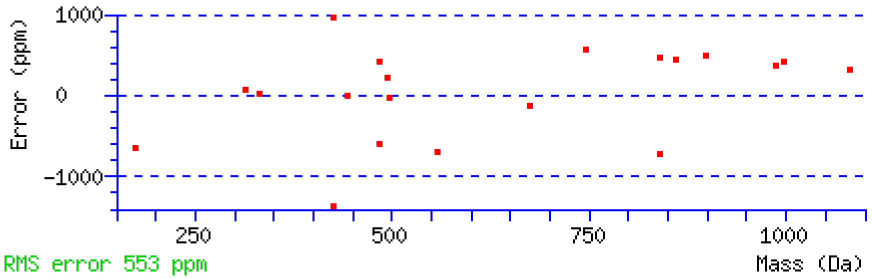

NCBI **BLAST** search of AIQLADDLGVR
(Parameters: blastp, nr protein database, expect=20000, no filter, PAM30)
Other BLAST web gateways

All matches to this query

Score	Mr(calc)	Delta	Sequence
57.4	1169.6404	-0.0005	AIQLADDLGVR

Mascot: http://www.matrixscience.com/

MATRIX SCIENCE MASCOT Search Results

Protein View: WP_005608182.1

30S ribosomal protein S6 [Granulicatella]

**Database:**	UB_target
**Score:**	77
**Monoisotopic mass (M_r):**	11336
**Calculated pI:**	4.95

Sequence similarity is available as an NCBI BLAST search of WP_005608182.1 against nr.

Search parameters

**MS data file:**	LTQ_19B022_Kuweit_Sample-GA-EVS.mgf
**Enzyme:**	Trypsin: cuts C-term side of KR unless next residue is P.
**Fixed modifications:**	Carbamidomethyl (C)
**Variable modifications:**	Deamidated (NQ), Oxidation (M)

Protein sequence coverage: 25%

Matched peptides shown in ***bold red***.

```
 1 MSQTSKYEIL YIIRPNIDEA AKAELVARFD AVLTDNGAVV VESKDWAKRR
51 FAYEIKDFQE GIYHLVNITA EDAAAIDEFD RLAKINNDIL RHMIVKLEA
```

Unformatted sequence string: 99 residues (for pasting into other applications).

Sort by	residue number	increasing mass	decreasing mass
Show	matched peptides only	predicted peptides also	

Query	Start – End	Observed	Mr(expt)	Mr(calc)	ppm	M	Score	Expect	Rank	U	Peptide
19980	57 – 81	951.4484	2851.3235	2851.3300	-2.30	0	42	0.00018	1	U	K.DFQEGIYHLVNITAEDAAAIDEFDR.L
19982	57 – 81	951.4498	2851.3275	2851.3300	-0.91	0	51	3e-005	1	U	K.DFQEGIYHLVNITAEDAAAIDEFDR.L
19983	57 – 81	951.4499	2851.3279	2851.3300	-0.74	0	43	0.00017	1	U	K.DFQEGIYHLVNITAEDAAAIDEFDR.L

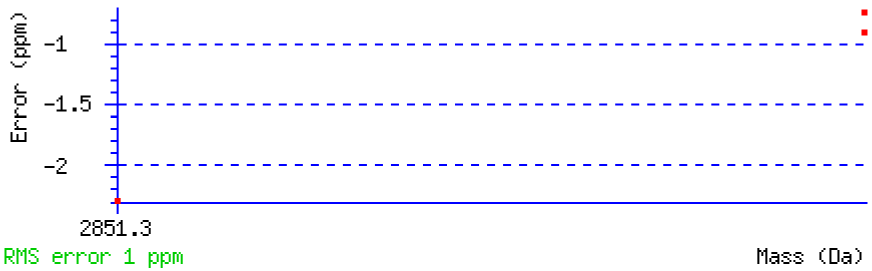

Mascot: http://www.matrixscience.com/

MATRIX SCIENCE Mascot Search Results

Peptide View

MS/MS Fragmentation of **DFQEGIYHLVNITAEDAAAIDEFDR**
Found in **WP_005608182.1** in **UB_target**, 30S ribosomal protein S6 [Granulicatella]

Match to Query 19982: 2851.327458 from(951.449762,3+) index(22051)
Title: Elution from: 114.538 to 114.538 period: 0 experiment: 1 cycles: 1 precIntensity: 268013.0 FinneganScanNumber: 27379 MStype: enumIsNormalMS rawFile: 19B022_Kuweit_Sample-GA-EVS.raw
Data file LTQ_19B022_Kuweit_Sample-GA-EVS.mgf

Click mouse within plot area to zoom in by factor of two about that point
Or, Plot from 100 to 2000 Da Full range
Show Y-axis ☐

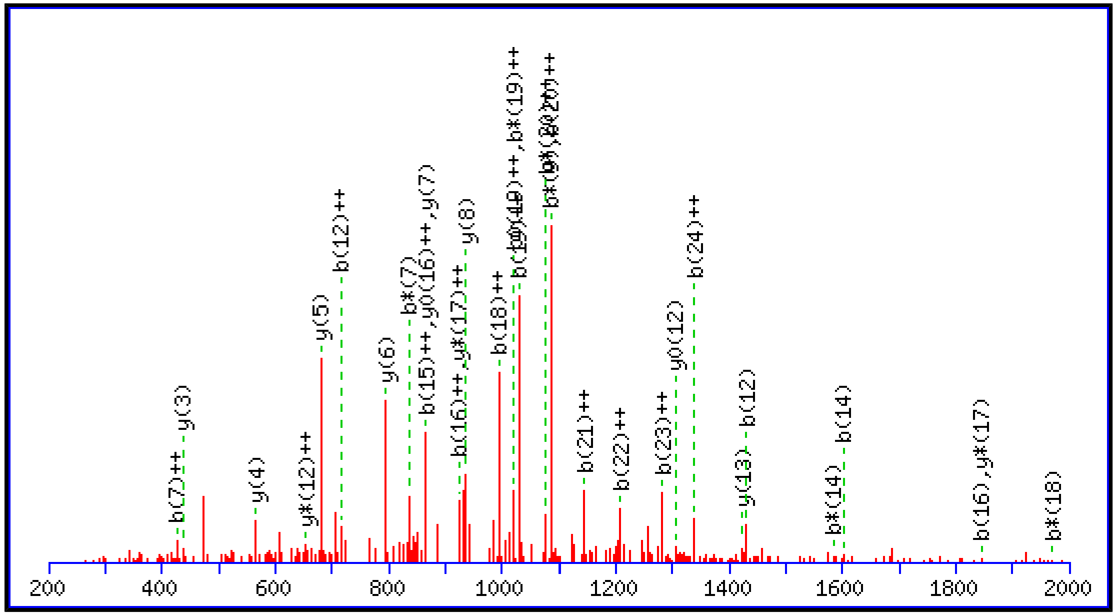

Label all possible matches Label matches used for scoring

Monoisotopic mass of neutral peptide Mr(calc): 2851.3300
Fixed modifications: Carbamidomethyl (C) (apply to specified residues or termini only)
Ions Score: 51 **Expect:** 3e-005

Matches : 33/282 fragment ions using 53 most intense peaks (help)

#	b	b^{++}	b^{*}	b^{*++}	b^{0}	b^{0++}	Seq.	y	y^{++}	y^{*}	y^{*++}	y^{0}	y^{0++}	#
1	116.0342	58.5207			98.0237	49.5155	D							25
2	263.1026	132.0550			245.0921	123.0497	F	2737.3104	1369.1588	2720.2839	1360.6456	2719.2998	1360.1536	24
3	391.1612	196.0842	374.1347	187.5710	373.1506	187.0790	Q	2590.2420	1295.6246	2573.2154	1287.1114	2572.2314	1286.6193	23
4	520.2038	260.6055	503.1773	252.0923	502.1932	251.6003	E	2462.1834	1231.5953	2445.1569	1223.0821	2444.1728	1222.5901	22
5	577.2253	289.1163	560.1987	280.6030	559.2147	280.1110	G	2333.1408	1167.0740	2316.1143	1158.5608	2315.1303	1158.0688	21
6	690.3093	345.6583	673.2828	337.1450	672.2988	336.6530	I	2276.1194	1138.5633	2259.0928	1130.0500	2258.1088	1129.5580	20
7	853.3727	***427.1900***	836.3461	418.6767	835.3621	418.1847	Y	2163.0353	1082.0213	2146.0087	1073.5080	2145.0247	1073.0160	19
8	990.4316	495.7194	973.4050	487.2062	972.4210	486.7141	H	1999.9720	1000.4896	1982.9454	991.9763	1981.9614	991.4843	18
9	1103.5156	552.2615	1086.4891	543.7482	1085.5051	543.2562	L	1862.9130	931.9602	1845.8865	923.4469	1844.9025	922.9549	17
10	1202.5841	601.7957	1185.5575	593.2824	1184.5735	592.7904	V	1749.8290	875.4181	1732.8024	866.9049	1731.8184	866.4128	16
11	1316.6270	658.8171	1299.6004	650.3039	1298.6164	649.8118	N	1650.7606	825.8839	1633.7340	817.3706	1632.7500	816.8786	15
12	**1429.7110**	***715.3592***	1412.6845	706.8459	1411.7005	706.3539	I	1536.7176	768.8625	1519.6911	760.3492	1518.7071	759.8572	14
13	1530.7587	765.8830	1513.7322	757.3697	1512.7482	756.8777	T	**1423.6336**	712.3204	1406.6070	703.8072	1405.6230	703.3151	13
14	**1601.7958**	801.4016	1584.7693	792.8883	1583.7853	792.3963	A	1322.5859	661.7966	1305.5594	653.2833	1304.5753	652.7913	12
15	1730.8384	***865.9229***	1713.8119	857.4096	1712.8279	856.9176	E	1251.5488	626.2780	1234.5222	617.7648	1233.5382	617.2727	11
16	**1845.8654**	***923.4363***	1828.8388	914.9230	1827.8548	914.4310	D	1122.5062	561.7567	1105.4796	553.2435	1104.4956	552.7515	10
17	1916.9025	958.9549	1899.8759	950.4416	1898.8919	949.9496	A	1007.4793	504.2433	990.4527	495.7300	989.4687	495.2380	9
18	1987.9396	***994.4734***	1970.9130	985.9602	1969.9290	985.4682	A	**936.4421**	468.7247	919.4156	460.2114	918.4316	459.7194	8
19	2058.9767	***1029.9920***	2041.9502	1021.4787	2040.9661	1020.9867	A	**865.4050**	433.2061	848.3785	424.6929	847.3945	424.2009	7
20	2172.0608	***1086.5340***	2155.0342	1078.0208	2154.0502	1077.5287	I	**794.3679**	397.6876	777.3414	389.1743	776.3573	388.6823	6
21	2287.0877	***1144.0475***	2270.0612	1135.5342	2269.0772	1135.0422	D	**681.2838**	341.1456	664.2573	332.6323	663.2733	332.1403	5
22	2416.1303	***1208.5688***	2399.1038	1200.0555	2398.1197	1199.5635	E	**566.2569**	283.6321	549.2304	275.1188	548.2463	274.6268	4
23	2563.1987	***1282.1030***	2546.1722	1273.5897	2545.1882	1273.0977	F	**437.2143**	219.1108	420.1878	210.5975	419.2037	210.1055	3
24	2678.2257	***1339.6165***	2661.1991	1331.1032	2660.2151	1330.6112	D	290.1459	145.5766	273.1193	137.0633	272.1353	136.5713	2
25							R	175.1190	88.0631	158.0924	79.5498			1

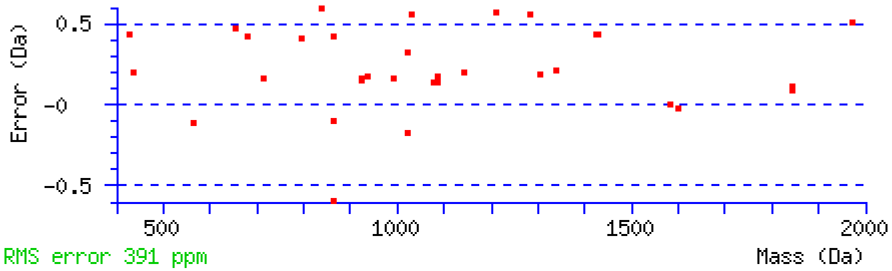

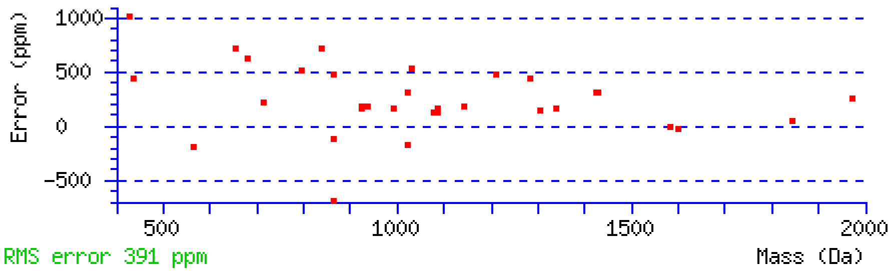

NCBI **BLAST** search of DFQEGIYHLVNITAEDAAAIDEFDR
(Parameters: blastp, nr protein database, expect=20000, no filter, PAM30)
Other BLAST web gateways

All matches to this query

Score	Mr(calc)	Delta	Sequence
50.9	2851.3300	-0.0026	DFQEGIYHLVNITAEDAAAIDEFDR
2.1	2851.3260	0.0014	SWDDANNQDGKRPATVTVTLYADGQK

Mascot: http://www.matrixscience.com/

MASCOT Search Results

Protein View: RKW29337.1

biotin/lipoyl-binding protein, partial [Granulicatella sp.]

**Database:**	UB_target
**Score:**	76
**Monoisotopic mass (M_r):**	23910
**Calculated pI:**	4.28

Sequence similarity is available as an NCBI BLAST search of RKW29337.1 against nr.

Search parameters

**MS data file:**	LTQ_19B022_Kuweit_Sample-GA-EVS.mgf
**Enzyme:**	Trypsin: cuts C-term side of KR unless next residue is P.
**Fixed modifications:**	Carbamidomethyl (C)
**Variable modifications:**	Deamidated (NQ), Oxidation (M)

Protein sequence coverage: 6%

Matched peptides shown in ***bold red***.

```
  1 MAFQFKMPDI GEGIAEGEIV KIDIKVGDTI QEDDILFEVQ NDKSVEEIPS
 51 PVSGKVLEVK VQEGTVARVG DIIVVIDDGS GPAEASAPAA APAAALAPAA
101 PAASSTFQFK LPDIGEGIAE GEIVKIDIKV GDKIAEDDIL FEVQNDKSVE
151 SIPSPVSGTV TAVLVSEGTV AHVGDVIVEI ATEGGSHAPA AAAPAAPAAP
201 AAAPAAPAAP TGVPAASNPG KLVLAMPSVR QYAREKGV
```

Unformatted sequence string: 238 residues (for pasting into other applications).

Sort by	residue number	increasing mass	decreasing mass
Show	matched peptides only	predicted peptides also	

Query	Start – End	Observed	Mr(expt)	Mr(calc)	ppm	M	Score	Expect	Rank	U	Peptide
9648	111 – 125	770.4153	1538.8161	1538.8192	-1.99	0	61	2.9e-006	1	U	K.LPDIGEGIAEGEIVK.I
9649	111 – 125	770.4155	1538.8165	1538.8192	-1.75	0	45	0.00011	1	U	K.LPDIGEGIAEGEIVK.I

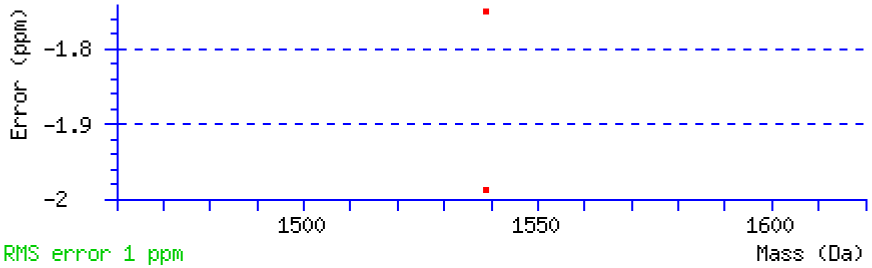

Mascot: http://www.matrixscience.com/

Mascot Search Results

Peptide View

MS/MS Fragmentation of **LPDIGEGIAEGEIVK**
Found in **RKW29337.1** in **UB_target**, biotin/lipoyl-binding protein, partial [Granulicatella sp.]

Match to Query 9648: 1538.816108 from(770.415330,2+) index(17213)
Title: Elution from: 96.935 to 96.935 period: 0 experiment: 1 cycles: 1 precIntensity: 301056.0 FinneganScanNumber: 21849 MStype: enumIsNormalMS
rawFile: 19B022_Kuweit_Sample-GA-EVS.raw
Data file LTQ_19B022_Kuweit_Sample-GA-EVS.mgf

Click mouse within plot area to zoom in by factor of two about that point
Or, Plot from 100 to 1400 Da Full range
Show Y-axis ☐

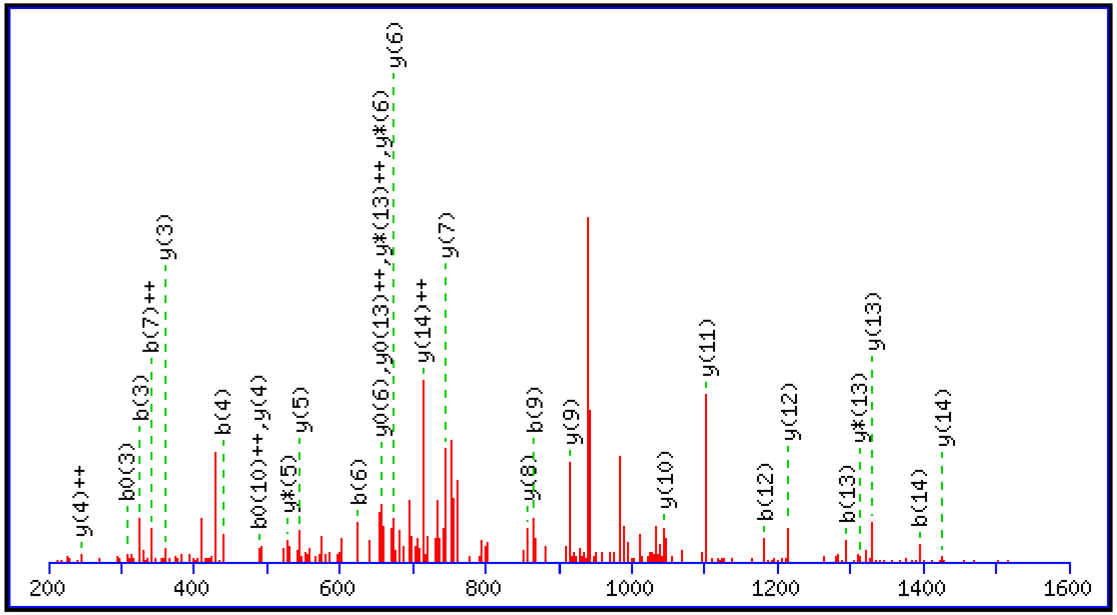

Label all possible matches Label matches used for scoring

Monoisotopic mass of neutral peptide Mr(calc): 1538.8192
Fixed modifications: Carbamidomethyl (C) (apply to specified residues or termini only)
Ions Score: 61 **Expect:** 2.9e-006

Matches : 30/130 fragment ions using 66 most intense peaks (help)

#	b	b^{++}	b^{0}	b^{0++}	Seq.	y	y^{++}	y*	y^{*++}	y^{0}	y^{0++}	#
1	114.0913	57.5493			L							15
2	211.1441	106.0757			P	***1426.7424***	713.8748	1409.7159	705.3616	1408.7318	704.8696	14
3	**326.1710**	163.5892	308.1605	154.5839	D	***1329.6896***	665.3485	1312.6631	656.8352	1311.6791	656.3432	13
4	**439.2551**	220.1312	421.2445	211.1259	I	***1214.6627***	607.8350	1197.6361	599.3217	1196.6521	598.8297	12
5	496.2766	248.6419	478.2660	239.6366	G	***1101.5786***	551.2930	1084.5521	542.7797	1083.5681	542.2877	11
6	**625.3192**	313.1632	607.3086	304.1579	E	***1044.5572***	522.7822	1027.5306	514.2689	1026.5466	513.7769	10
7	682.3406	341.6740	664.3301	332.6687	G	***915.5146***	458.2609	898.4880	449.7477	897.5040	449.2556	9
8	795.4247	398.2160	777.4141	389.2107	I	***858.4931***	429.7502	841.4666	421.2369	840.4825	420.7449	8
9	**866.4618**	433.7345	848.4512	424.7293	A	***745.4090***	373.2082	728.3825	364.6949	727.3985	364.2029	7
10	995.5044	498.2558	977.4938	489.2506	E	***674.3719***	337.6896	657.3454	329.1763	656.3614	328.6843	6
11	1052.5259	526.7666	1034.5153	517.7613	G	***545.3293***	273.1683	528.3028	264.6550	527.3188	264.1630	5
12	**1181.5685**	591.2879	1163.5579	582.2826	E	***488.3079***	244.6576	471.2813	236.1443	470.2973	235.6523	4
13	**1294.6525**	647.8299	1276.6420	638.8246	I	***359.2653***	180.1363	342.2387	171.6230			3
14	**1393.7209**	697.3641	1375.7104	688.3588	V	246.1812	123.5942	229.1547	115.0810			2
15					K	147.1128	74.0600	130.0863	65.5468			1

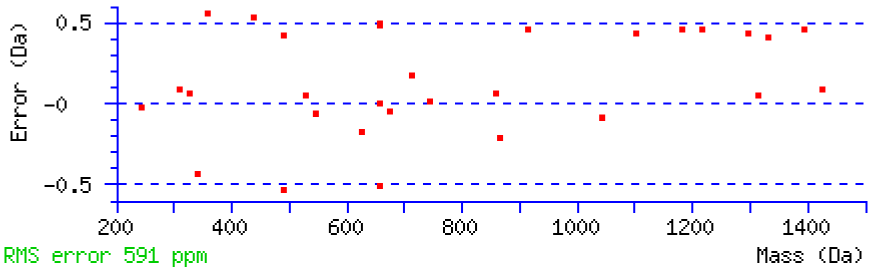

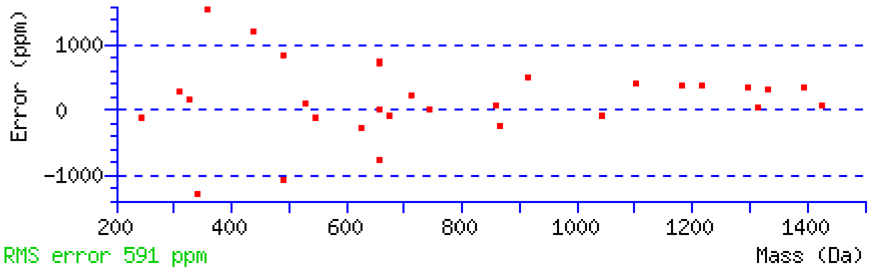

NCBI **BLAST** search of LPDIGEGIAEGEIVK
(Parameters: blastp, nr protein database, expect=20000, no filter, PAM30)
Other BLAST web gateways

All matches to this query

Score	Mr(calc)	Delta	Sequence

61.1	1538.8192	-0.0031	LPDIGEGIAEGEIVK

Mascot: http://www.matrixscience.com/

MATRIX SCIENCE MASCOT Search Results

Protein View: RKW27683.1

ROK family protein [Granulicatella sp.]

**Database:**	UB_target
**Score:**	75
**Monoisotopic mass (M_r):**	32054
**Calculated pI:**	5.67

Sequence similarity is available as an NCBI BLAST search of RKW27683.1 against nr.

Search parameters

**MS data file:**	LTQ_19B022_Kuweit_Sample-GA-EVS.mgf
**Enzyme:**	Trypsin: cuts C-term side of KR unless next residue is P.
**Fixed modifications:**	Carbamidomethyl (C)
**Variable modifications:**	Deamidated (NQ), Oxidation (M)

Protein sequence coverage: 5%

Matched peptides shown in ***bold red***.

```
  1 MTNLYGSLEA GGTKFICAVA DEDFNTVEEL QFPTTTPKET LKKTADFFAK
 51 FKNLAAIGIG SFGPIDVDPK SKTYGYITTT PKPNWANVDV VGALKKRVDV
101 PIYFTTDVNS SAYGEVYARN NRGENIETLV YYTIGTGIGA GVIQRGEFIG
151 GTSHPEMGHV YVSKHPIDVA NNFDGVCPFH KGCLEGLAAG PSLEARTGVR
201 GEHIDIASDV WDVQASYIAQ AAIQATLTFR PEKIVFGGGV MAQNHMLERV
251 HRMFEELLNG YVPTPPVKDF IVTPAVDNNG SATLGNYVLA KSLVK
```

Unformatted sequence string: 295 residues (for pasting into other applications).

Sort by	residue number	increasing mass	decreasing mass
Show	matched peptides only	predicted peptides also	

Query	Start – End	Observed	Mr(expt)	Mr(calc)	ppm	M	Score	Expect	Rank	U	Peptide
9278	182 – 196	750.8768	1499.7391	1499.7402	-0.72	0	39	0.00044	1	U	K.GCLEGLAAGPSLEAR.T
9279	182 – 196	750.8771	1499.7397	1499.7402	-0.32	0	60	4.1e-006	1	U	K.GCLEGLAAGPSLEAR.T
9280	182 – 196	750.8773	1499.7400	1499.7402	-0.11	0	35	0.0013	1	U	K.GCLEGLAAGPSLEAR.T

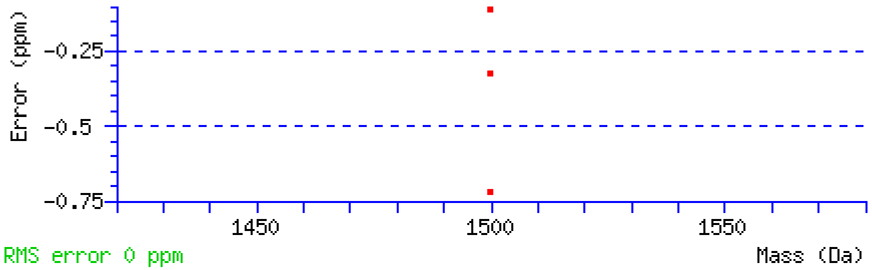

Mascot: http://www.matrixscience.com/

Mascot Search Results

Peptide View

MS/MS Fragmentation of **GCLEGLAAGPSLEAR**
Found in **RKW27683.1** in **UB_target**, ROK family protein [Granulicatella sp.]

Match to Query 9279: 1499.739722 from(750.877137,2+) index(13011)
Title: Elution from: 80.493 to 80.493 period: 0 experiment: 1 cycles: 1 precIntensity: 307912.0 FinneganScanNumber: 16808 MStype: enumIsNormalMS rawFile: 19B022_Kuweit_Sample-GA-EVS.raw
Data file LTQ_19B022_Kuweit_Sample-GA-EVS.mgf

Click mouse within plot area to zoom in by factor of two about that point
Or, Plot from 100 to 1400 Da Full range
Show Y-axis

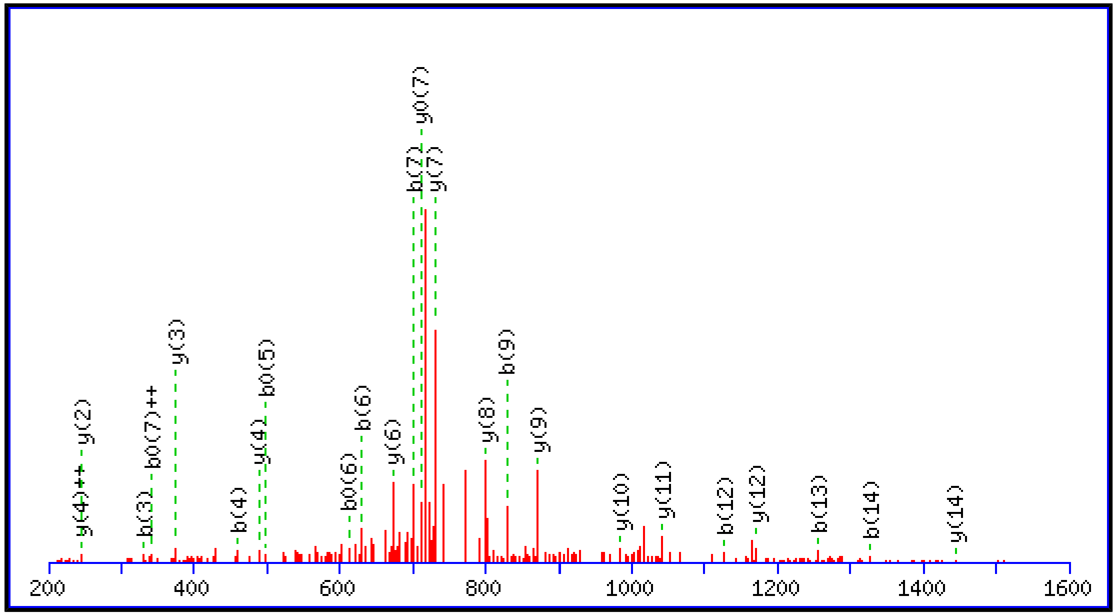

Label all possible matches Label matches used for scoring

Monoisotopic mass of neutral peptide Mr(calc): 1499.7402
Fixed modifications: Carbamidomethyl (C) (apply to specified residues or termini only)
Ions Score: 60 **Expect:** 4.1e-006

Matches : 24/130 fragment ions using 52 most intense peaks (help)

#	b	b^{++}	b^0	b^{0++}	Seq.	y	y^{++}	y*	y^{*++}	y^0	y^{0++}	#
1	58.0287	29.5180			G							15
2	218.0594	109.5333			C	1443.7260	722.3667	1426.6995	713.8534	1425.7155	713.3614	14
3	331.1435	166.0754			L	1283.6954	642.3513	1266.6688	633.8381	1265.6848	633.3461	13
4	460.1860	230.5967	442.1755	221.5914	E	1170.6113	585.8093	1153.5848	577.2960	1152.6008	576.8040	12
5	517.2075	259.1074	499.1969	250.1021	G	1041.5687	521.2880	1024.5422	512.7747	1023.5582	512.2827	11
6	630.2916	315.6494	612.2810	306.6441	L	984.5473	492.7773	967.5207	484.2640	966.5367	483.7720	10
7	701.3287	351.1680	683.3181	342.1627	A	871.4632	436.2352	854.4367	427.7220	853.4526	427.2300	9
8	772.3658	386.6865	754.3552	377.6813	A	800.4261	400.7167	783.3995	392.2034	782.4155	391.7114	8
9	829.3873	415.1973	811.3767	406.1920	G	729.3890	365.1981	712.3624	356.6849	711.3784	356.1928	7
10	926.4400	463.7237	908.4295	454.7184	P	672.3675	336.6874	655.3410	328.1741	654.3569	327.6821	6
11	1013.4721	507.2397	995.4615	498.2344	S	575.3148	288.1610	558.2882	279.6477	557.3042	279.1557	5
12	1126.5561	563.7817	1108.5456	554.7764	L	488.2827	244.6450	471.2562	236.1317	470.2722	235.6397	4
13	1255.5987	628.3030	1237.5881	619.2977	E	375.1987	188.1030	358.1721	179.5897	357.1881	179.0977	3
14	1326.6358	663.8216	1308.6253	654.8163	A	246.1561	123.5817	229.1295	115.0684			2
15					R	175.1190	88.0631	158.0924	79.5498			1

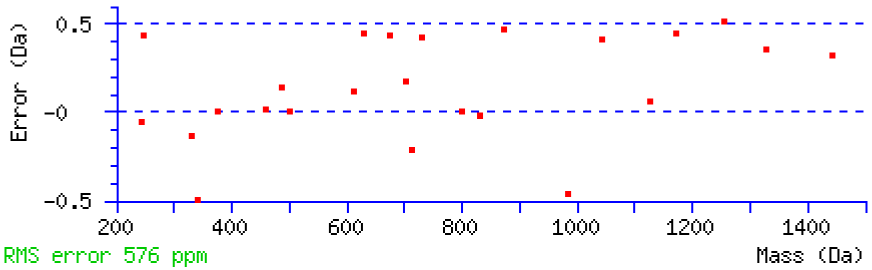

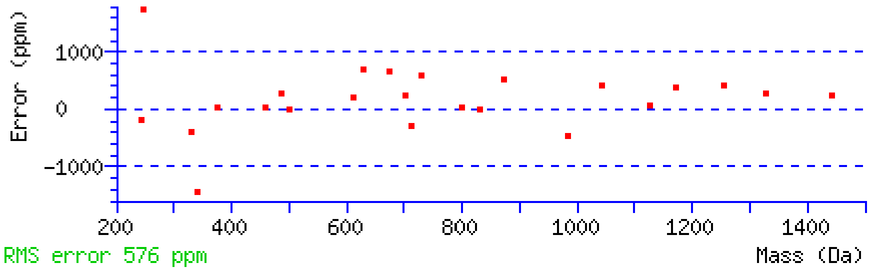

NCBI **BLAST** search of GCLEGLAAGPSLEAR
(Parameters: blastp, nr protein database, expect=20000, no filter, PAM30)
Other BLAST web gateways

All matches to this query

Score	Mr(calc)	Delta	Sequence

60.1	1499.7402	-0.0005	GCLEGLAAGPSLEAR

Mascot: http://www.matrixscience.com/

MASCOT Search Results

Protein View: QDC26953.1

translation superoxide dismutase, partial [Granulicatella adiacens]

**Database:**	UB_target
**Score:**	74
**Monoisotopic mass (M_r):**	14729
**Calculated pI:**	5.38

Sequence similarity is available as an NCBI BLAST search of QDC26953.1 against nr.

Search parameters

**MS data file:**	LTQ_19B022_Kuweit_Sample-GA-EVS.mgf
**Enzyme:**	Trypsin: cuts C-term side of KR unless next residue is P.
**Fixed modifications:**	Carbamidomethyl (C)
**Variable modifications:**	Deamidated (NQ), Oxidation (M)

Protein sequence coverage: 15%

Matched peptides shown in ***bold red***.

```
  1 HHDKHHNAYV TNLNAAVEKH PELFEKTVEE LVSDLNAVPE DIRVAVRNNG
 51 GGHANHSLFW TQLSLDGAKA PEGALLAAIN EAFGSFDEFK AAFAQAAATR
101 FGSGWAWLVL SNGKLEVVST PNQDNPLSEG KTPLLGL
```

Unformatted sequence string: 137 residues (for pasting into other applications).

Sort by	residue number	increasing mass	decreasing mass
Show	matched peptides only	predicted peptides also	

Query	Start – End	Observed	Mr(expt)	Mr(calc)	ppm	M	Score	Expect	Rank	U	Peptide
16163	70 – 90	733.0311	2196.0714	2196.0739	-1.13	0	74	1.9e-007	1	U	K.APEGALLAAINEAFGSFDEFK.A

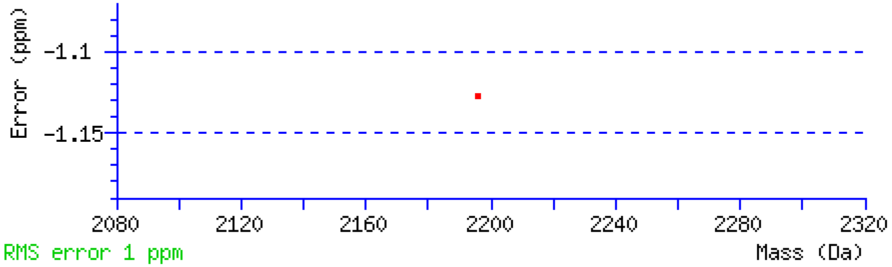

Mascot: http://www.matrixscience.com/

MATRIX SCIENCE Mascot Search Results

Peptide View

MS/MS Fragmentation of **APEGALLAAINEAFGSFDEFK**
Found in **QDC26953.1** in **UB_target**, translation superoxide dismutase, partial [Granulicatella adiacens]

Match to Query 16163: 2196.071391 from(733.031073,3+) index(23169)
Title: Elution from: 119.160 to 119.160 period: 0 experiment: 1 cycles: 1 precIntensity: 777383.0 FinneganScanNumber: 28731 MStype: enumIsNormalMS rawFile: 19B022_Kuweit_Sample-GA-EVS.raw
Data file LTQ_19B022_Kuweit_Sample-GA-EVS.mgf

Click mouse within plot area to zoom in by factor of two about that point
Or, Plot from 150 to 1100 Da Full range
Show Y-axis

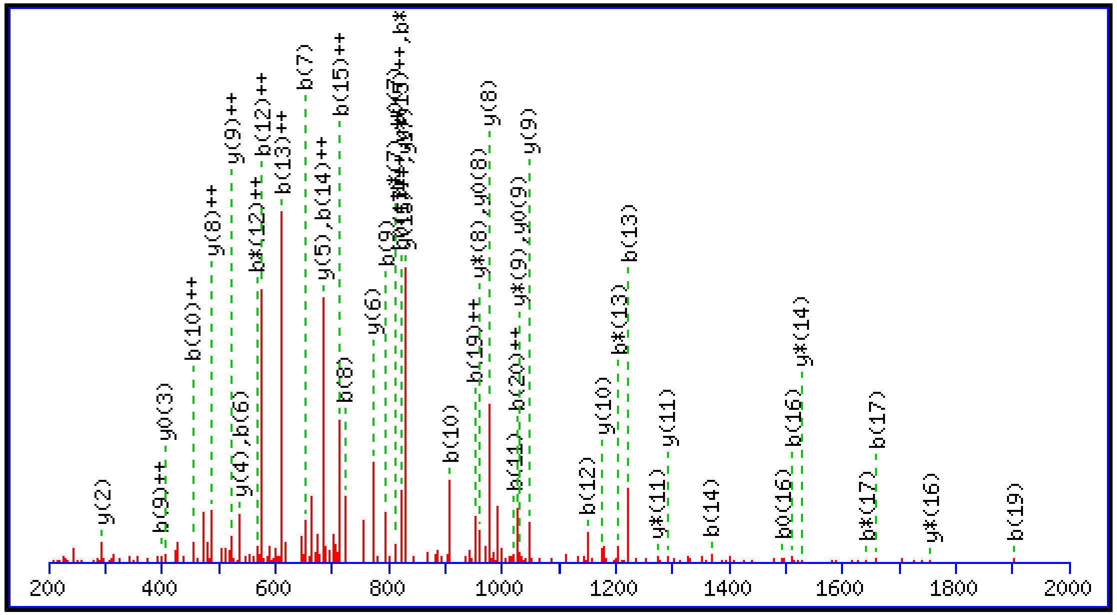

Label all possible matches Label matches used for scoring

Monoisotopic mass of neutral peptide Mr(calc): 2196.0739
Fixed modifications: Carbamidomethyl (C) (apply to specified residues or termini only)
Ions Score: 74 **Expect:** 1.9e-007

Matches : 49/212 fragment ions using 64 most intense peaks (help)

#	b	b++	b*	b*++	b0	b0++	Seq.	y	y++	y*	y*++	y0	y0++	#
1	72.0444	36.5258					A							21
2	169.0972	85.0522					P	2126.0441	1063.5257	2109.0175	1055.0124	2108.0335	1054.5204	20
3	298.1397	149.5735			280.1292	140.5682	E	2028.9913	1014.9993	2011.9648	1006.4860	2010.9807	1005.9940	19
4	355.1612	178.0842			337.1506	169.0790	G	1899.9487	950.4780	1882.9222	941.9647	1881.9381	941.4727	18
5	426.1983	213.6028			408.1878	204.5975	A	1842.9272	921.9673	1825.9007	913.4540	1824.9167	912.9620	17
6	539.2824	270.1448			521.2718	261.1396	L	1771.8901	886.4487	1754.8636	877.9354	1753.8796	877.4434	16
7	652.3665	326.6869			634.3559	317.6816	L	1658.8061	829.9067	1641.7795	821.3934	1640.7955	820.9014	15
8	723.4036	362.2054			705.3930	353.2001	A	1545.7220	773.3646	1528.6955	764.8514	1527.7114	764.3594	14
9	794.4407	397.7240			776.4301	388.7187	A	1474.6849	737.8461	1457.6583	729.3328	1456.6743	728.8408	13
10	907.5247	454.2660			889.5142	445.2607	I	1403.6478	702.3275	1386.6212	693.8142	1385.6372	693.3222	12
11	1021.5677	511.2875	1004.5411	502.7742	1003.5571	502.2822	N	1290.5637	645.7855	1273.5372	637.2722	1272.5531	636.7802	11
12	1150.6103	575.8088	1133.5837	567.2955	1132.5997	566.8035	E	1176.5208	588.7640	1159.4942	580.2508	1158.5102	579.7587	10
13	1221.6474	611.3273	1204.6208	602.8141	1203.6368	602.3220	A	1047.4782	524.2427	1030.4516	515.7295	1029.4676	515.2374	9
14	1368.7158	684.8615	1351.6892	676.3483	1350.7052	675.8563	F	976.4411	488.7242	959.4145	480.2109	958.4305	479.7189	8
15	1425.7373	713.3723	1408.7107	704.8590	1407.7267	704.3670	G	829.3727	415.1900	812.3461	406.6767	811.3621	406.1847	7
16	1512.7693	756.8883	1495.7427	748.3750	1494.7587	747.8830	S	772.3512	386.6792	755.3246	378.1660	754.3406	377.6740	6
17	1659.8377	830.4225	1642.8112	821.9092	1641.8271	821.4172	F	685.3192	343.1632	668.2926	334.6499	667.3086	334.1579	5
18	1774.8646	887.9360	1757.8381	879.4227	1756.8541	878.9307	D	538.2508	269.6290	521.2242	261.1157	520.2402	260.6237	4
19	1903.9072	952.4573	1886.8807	943.9440	1885.8967	943.4520	E	423.2238	212.1155	406.1973	203.6023	405.2132	203.1103	3
20	2050.9756	1025.9915	2033.9491	1017.4782	2032.9651	1016.9862	F	294.1812	147.5942	277.1547	139.0810			2
21							K	147.1128	74.0600	130.0863	65.5468			1

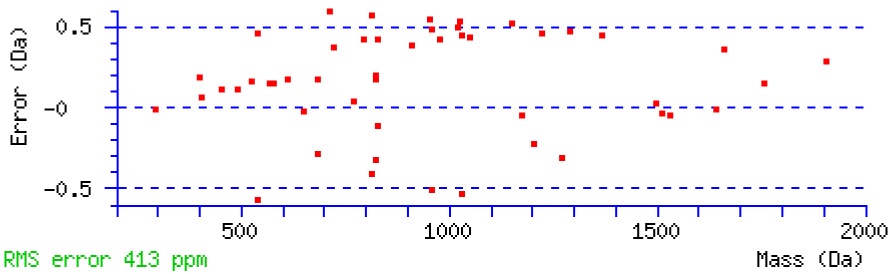

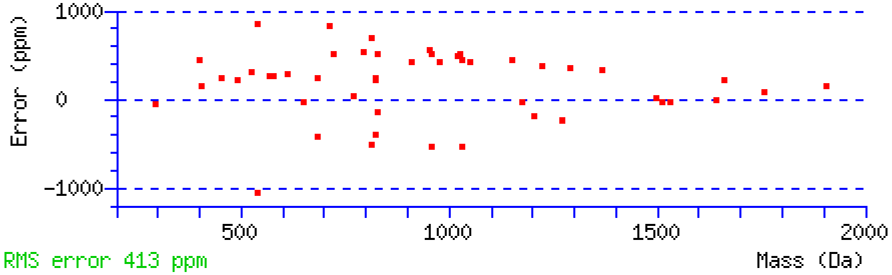

NCBI **BLAST** search of APEGALLAAINEAFGSFDEFK
(Parameters: blastp, nr protein database, expect=20000, no filter, PAM30)
Other BLAST web gateways

All matches to this query

Score	Mr(calc)	Delta	Sequence
74.4	2196.0739	-0.0025	APEGALLAAINEAFGSFDEFK
5.6	2196.0674	0.0040	LFSTHFDKFLMENPQVAR
5.5	2196.0674	0.0040	LFSTHFDKFLMENPQVAR
1.0	2196.0660	0.0054	MEIDPFKDYLIESEPDKK
0.5	2196.0680	0.0034	NEWIYDKQYKGWYYLK
0.2	2196.0698	0.0016	DVLFIDASNEFEKGKNQNK

Mascot: http://www.matrixscience.com/

MATRIX SCIENCE MASCOT Search Results

Protein View: WP_005605710.1

LysM peptidoglycan-binding domain-containing protein [Granulicatella adiacens]

**Database:**	UB_target
**Score:**	74
**Monoisotopic mass (M_r):**	20808
**Calculated pI:**	5.88

Sequence similarity is available as an NCBI BLAST search of WP_005605710.1 against nr.

Search parameters

**MS data file:**	LTQ_19B022_Kuweit_Sample-GA-EVS.mgf
**Enzyme:**	Trypsin: cuts C-term side of KR unless next residue is P.
**Fixed modifications:**	Carbamidomethyl (C)
**Variable modifications:**	Deamidated (NQ), Oxidation (M)

Protein sequence coverage: 8%

Matched peptides shown in ***bold red***.

```
  1 MSDNFNNEDL NKNEEQPWER KFGEDENLKN RQFSRSARNS GGKAVAPLSN
 51 VLLFVFLIVI VAPVLFMWWF SIVNSSNQVK PRTADDVMLT KTVETTTVAP
101 ETTVAPTTKQ EATTAAPEAT TTAPRQAETT QQTPTTAAQQ NGNYGTYVVK
151 QGDTLYRIAV NHGMDVATLK QINGLSGDNI APGTTLKVKQ
```

Unformatted sequence string: 190 residues (for pasting into other applications).

Sort by	residue number	increasing mass	decreasing mass
Show	matched peptides only	predicted peptides also	

Query	Start – End	Observed	Mr(expt)	Mr(calc)	ppm	M	Score	Expect	Rank	U	Peptide
11071	171 – 187	849.9533	1697.8920	1697.8948	-1.63	0	32	0.0019	1	U	K.QINGLSGDNIAPGTTLK.V
11072	171 – 187	849.9534	1697.8922	1697.8948	-1.51	0	53	1.5e-005	1	U	K.QINGLSGDNIAPGTTLK.V
11073	171 – 187	849.9548	1697.8950	1697.8948	0.098	0	47	6.7e-005	1	U	K.QINGLSGDNIAPGTTLK.V

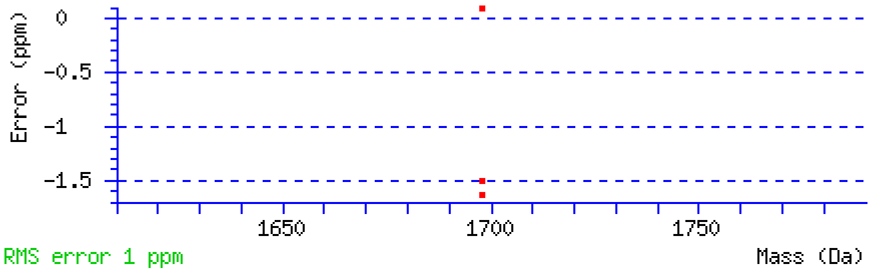

Mascot: http://www.matrixscience.com/

MATRIX SCIENCE Mascot Search Results

Peptide View

MS/MS Fragmentation of **QINGLSGDNIAPGTTLK**
Found in **WP_005605710.1** in **UB_target**, LysM peptidoglycan-binding domain-containing protein [Granulicatella adiacens]

Match to Query 11072: 1697.892242 from(849.953397,2+) index(11283)
Title: Elution from: 73.648 to 73.648 period: 0 experiment: 1 cycles: 1 precIntensity: 473844.0 FinneganScanNumber: 14747 MStype: enumIsNormalMS rawFile: 19B022_Kuweit_Sample-GA-EVS.raw
Data file LTQ_19B022_Kuweit_Sample-GA-EVS.mgf

Click mouse within plot area to zoom in by factor of two about that point

Or, Plot from 100 to 2000 Da Full range

Show Y-axis

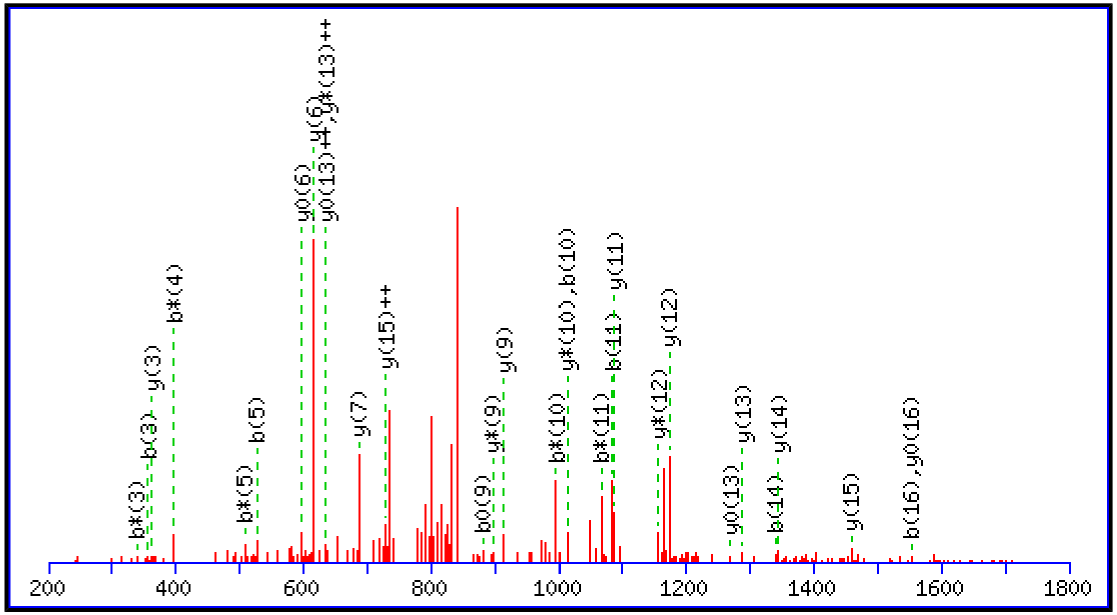

Label all possible matches   Label matches used for scoring

Monoisotopic mass of neutral peptide Mr(calc): 1697.8948
Fixed modifications: Carbamidomethyl (C) (apply to specified residues or termini only)
Ions Score: 53 **Expect:** 1.5e-005

Mascot Search Results: Peptide View

Matches : 30/178 fragment ions using 45 most intense peaks (help)

#	b	b^{++}	b*	b^{*++}	b^{0}	b^{0++}	Seq.	y	y^{++}	y*	y^{*++}	y^{0}	y^{0++}	#
1	129.0659	65.0366	112.0393	56.5233			Q							17
2	242.1499	121.5786	225.1234	113.0653			I	1570.8435	785.9254	1553.8170	777.4121	1552.8329	776.9201	16
3	**356.1928**	178.6001	339.1663	170.0868			N	***1457.7594***	729.3834	1440.7329	720.8701	1439.7489	720.3781	15
4	413.2143	207.1108	396.1878	198.5975			G	***1343.7165***	672.3619	1326.6900	663.8486	1325.7060	663.3566	14
5	**526.2984**	263.6528	509.2718	255.1396			L	***1286.6951***	643.8512	1269.6685	635.3379	1268.6845	634.8459	13
6	613.3304	307.1688	596.3039	298.6556	595.3198	298.1636	S	***1173.6110***	587.3091	1156.5844	578.7959	1155.6004	578.3039	12
7	670.3519	335.6796	653.3253	327.1663	652.3413	326.6743	G	***1086.5790***	543.7931	1069.5524	535.2798	1068.5684	534.7878	11
8	785.3788	393.1930	768.3523	384.6798	767.3682	384.1878	D	1029.5575	515.2824	1012.5310	506.7691	1011.5469	506.2771	10
9	899.4217	450.2145	882.3952	441.7012	881.4112	441.2092	N	***914.5306***	457.7689	897.5040	449.2556	896.5200	448.7636	9
10	**1012.5058**	506.7565	995.4792	498.2433	994.4952	497.7513	I	800.4876	400.7475	783.4611	392.2342	782.4771	391.7422	8
11	**1083.5429**	542.2751	1066.5164	533.7618	1065.5323	533.2698	A	***687.4036***	344.2054	670.3770	335.6921	669.3930	335.2001	7
12	1180.5957	590.8015	1163.5691	582.2882	1162.5851	581.7962	P	***616.3665***	308.6869	599.3399	300.1736	598.3559	299.6816	6
13	1237.6171	619.3122	1220.5906	610.7989	1219.6066	610.3069	G	519.3137	260.1605	502.2871	251.6472	501.3031	251.1552	5
14	**1338.6648**	669.8360	1321.6383	661.3228	1320.6543	660.8308	T	462.2922	231.6498	445.2657	223.1365	444.2817	222.6445	4
15	1439.7125	720.3599	1422.6860	711.8466	1421.7019	711.3546	T	***361.2445***	181.1259	344.2180	172.6126	343.2340	172.1206	3
16	**1552.7966**	776.9019	1535.7700	768.3886	1534.7860	767.8966	L	260.1969	130.6021	243.1703	122.0888			2
17							K	147.1128	74.0600	130.0863	65.5468			1

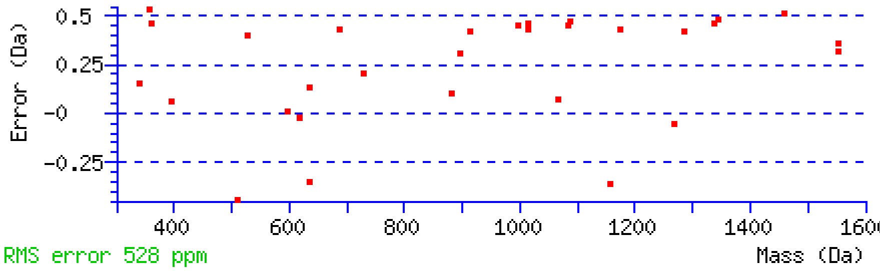

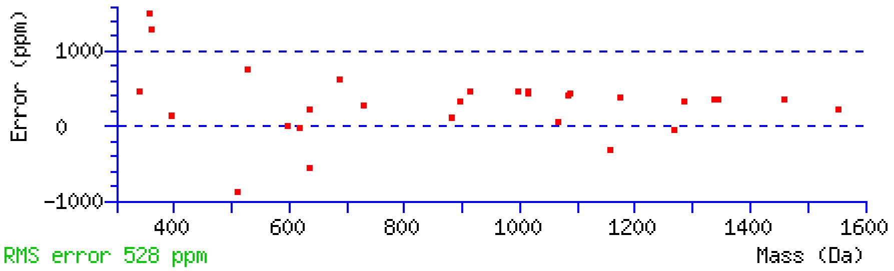

NCBI **BLAST** search of QINGLSGDNIAPGTTLK
(Parameters: blastp, nr protein database, expect=20000, no filter, PAM30)
Other BLAST web gateways

All matches to this query

Score	Mr(calc)	Delta	Sequence
53.2	1697.8948	-0.0026	QINGLSGDNIAPGTTLK
0.1	1697.8882	0.0040	NPIGSENMAGLARVIR

Mascot: http://www.matrixscience.com/

MASCOT Search Results

Protein View: RKW26695.1

DUF1307 domain-containing protein [Granulicatella sp.]

**Database:**	UB_target
**Score:**	73
**Monoisotopic mass (M_r):**	16653
**Calculated pI:**	7.68

Sequence similarity is available as an NCBI BLAST search of RKW26695.1 against nr.

Search parameters

**MS data file:**	LTQ_19B022_Kuweit_Sample-GA-EVS.mgf
**Enzyme:**	Trypsin: cuts C-term side of KR unless next residue is P.
**Fixed modifications:**	Carbamidomethyl (C)
**Variable modifications:**	Deamidated (NQ), Oxidation (M)

Protein sequence coverage: 9%

Matched peptides shown in ***bold red***.

```
  1 MNFMKKTLKL  IAVFFAALFV  LLGCGKEESS  SFELNQNGVT  SVLTYYYNND
 51 LVTKQTATNT  YDLKQLGVTE  EDAKKQIEGV  NNKYTAVDGV  TASIESKDGT
101 LIQTLTVDYT  KAKVSELRKA  FPQEFAGDGD  KISFKASKES  LLQAGYKEKK
```

Unformatted sequence string: 150 residues (for pasting into other applications).

Sort by	residue number	increasing mass	decreasing mass
Show	matched peptides only	predicted peptides also	

Query	Start – End	Observed	Mr(expt)	Mr(calc)	ppm	M	Score	Expect	Rank	U	Peptide
8839	84 – 97	720.8641	1439.7137	1439.7144	-0.48	0	45	8.7e-005	1	U	K.YTAVDGVTASIESK.D
8841	84 – 97	720.8652	1439.7159	1439.7144	1.07	0	58	6.4e-006	1	U	K.YTAVDGVTASIESK.D

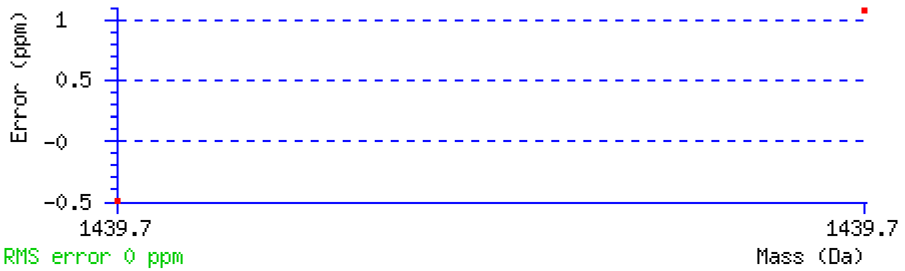

RMS error 0 ppm

Mascot: http://www.matrixscience.com/

MATRIX SCIENCE Mascot Search Results

Peptide View

MS/MS Fragmentation of **YTAVDGVTASIESK**
Found in **RKW26695.1** in **UB_target**, DUF1307 domain-containing protein [Granulicatella sp.]

Match to Query 8841: 1439.715930 from(720.865241,2+) index(10004)
Title: Elution from: 68.788 to 68.788 period: 0 experiment: 1 cycles: 1 precIntensity: 475581.0 FinneganScanNumber: 13256 MStype: enumIsNormalMS rawFile: 19B022_Kuweit_Sample-GA-EVS.raw
Data file LTQ_19B022_Kuweit_Sample-GA-EVS.mgf

Click mouse within plot area to zoom in by factor of two about that point
Or, [Plot from] 100 to 1400 Da [Full range]
Show Y-axis ☐

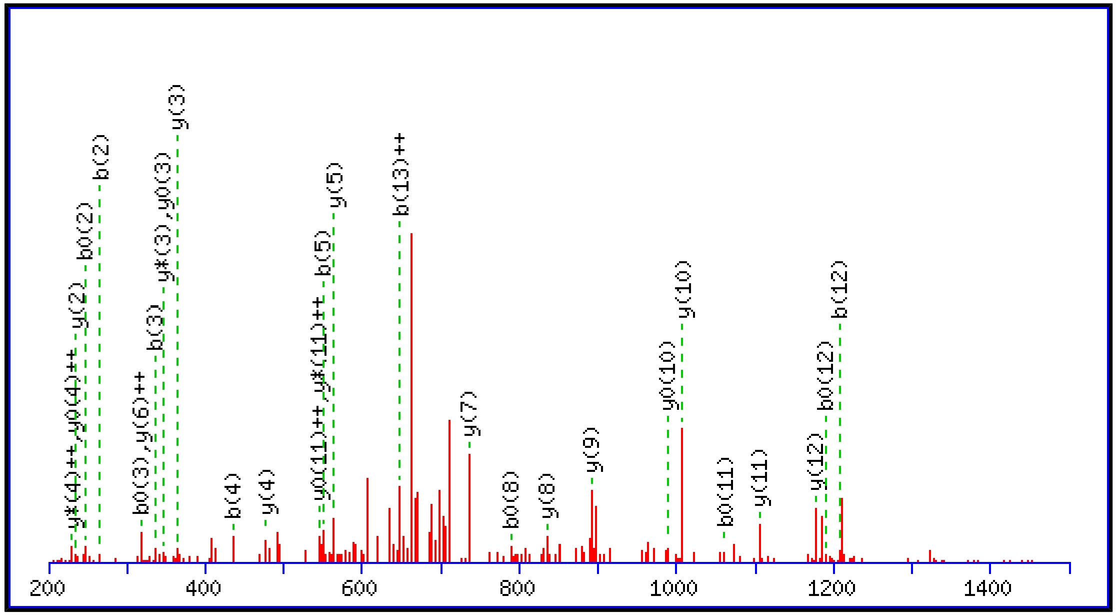

Label all possible matches   Label matches used for scoring

Monoisotopic mass of neutral peptide Mr(calc): 1439.7144
Fixed modifications: Carbamidomethyl (C) (apply to specified residues or termini only)
Ions Score: 58 **Expect:** 6.4e-006

Matches : 29/126 fragment ions using 51 most intense peaks (help)

#	b	b^{++}	b^0	b^{0++}	Seq.	y	y^{++}	y*	y^{*++}	y^0	y^{0++}	#
1	164.0706	82.5389			Y							14
2	**265.1183**	133.0628	247.1077	124.0575	T	1277.6583	639.3328	1260.6318	630.8195	1259.6478	630.3275	13
3	**336.1554**	168.5813	318.1448	159.5761	A	***1176.6107***	588.8090	1159.5841	580.2957	1158.6001	579.8037	12
4	**435.2238**	218.1155	417.2132	209.1103	V	***1105.5735***	553.2904	1088.5470	544.7771	1087.5630	544.2851	11
5	**550.2508**	275.6290	532.2402	266.6237	D	***1006.5051***	503.7562	989.4786	495.2429	988.4946	494.7509	10
6	607.2722	304.1397	589.2617	295.1345	G	***891.4782***	446.2427	874.4516	437.7295	873.4676	437.2374	9
7	706.3406	353.6740	688.3301	344.6687	V	***834.4567***	417.7320	817.4302	409.2187	816.4462	408.7267	8
8	807.3883	404.1978	789.3777	395.1925	T	***735.3883***	368.1978	718.3618	359.6845	717.3777	359.1925	7
9	878.4254	439.7164	860.4149	430.7111	A	634.3406	317.6740	617.3141	309.1607	616.3301	308.6687	6
10	965.4575	483.2324	947.4469	474.2271	S	***563.3035***	282.1554	546.2770	273.6421	545.2930	273.1501	5
11	1078.5415	539.7744	1060.5310	530.7691	I	***476.2715***	238.6394	459.2449	230.1261	458.2609	229.6341	4
12	**1207.5841**	604.2957	1189.5735	595.2904	E	***363.1874***	182.0974	346.1609	173.5841	345.1769	173.0921	3
13	1294.6161	647.8117	1276.6056	638.8064	S	***234.1448***	117.5761	217.1183	109.0628	216.1343	108.5708	2
14					K	147.1128	74.0600	130.0863	65.5468			1

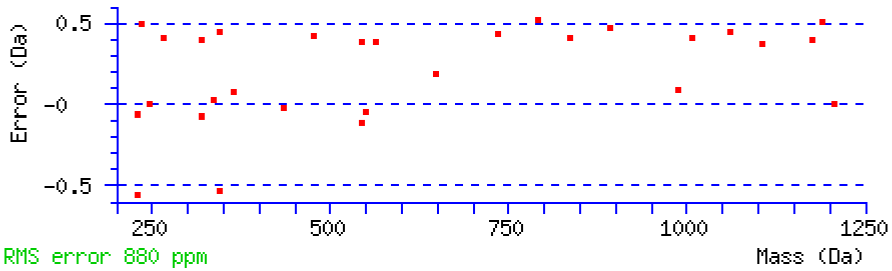

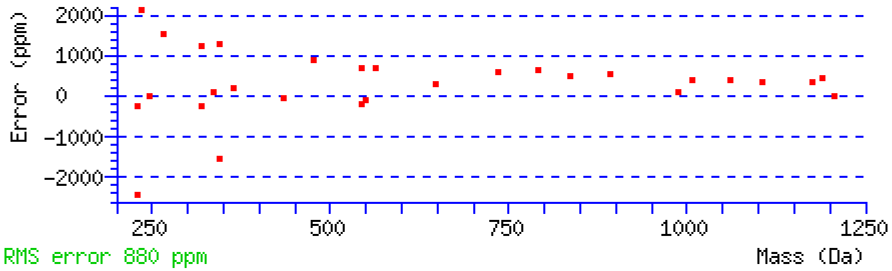

NCBI **BLAST** search of YTAVDGVTASIESK
(Parameters: blastp, nr protein database, expect=20000, no filter, PAM30)
Other BLAST web gateways

All matches to this query

Score	Mr(calc)	Delta	Sequence
57.6	1439.7144	0.0015	YTAVDGVTASIESK

Mascot: http://www.matrixscience.com/

MATRIX SCIENCE MASCOT Search Results

Protein View: RKW29417.1

thiol reductase thioredoxin, partial [Granulicatella sp.]

**Database:**	UB_target
**Score:**	73
**Monoisotopic mass (M_r):**	8479
**Calculated pI:**	4.93

Sequence similarity is available as an NCBI BLAST search of RKW29417.1 against nr.

Search parameters

**MS data file:**	LTQ_19B022_Kuweit_Sample-GA-EVS.mgf
**Enzyme:**	Trypsin: cuts C-term side of KR unless next residue is P.
**Fixed modifications:**	Carbamidomethyl (C)
**Variable modifications:**	Deamidated (NQ), Oxidation (M)

Protein sequence coverage: 28%

Matched peptides shown in ***bold red***.

```
 1 MEGFLMEQFA KDVSQFVETT AEKVEALIGE GKEVVLFVGR PTCPYCRRFA
51 PKMNEAREAL GKEMYFINSE DRT
```

Unformatted sequence string: 73 residues (for pasting into other applications).

Sort by	residue number	increasing mass	decreasing mass
Show	matched peptides only	predicted peptides also	

Query	Start – End	Observed	Mr(expt)	Mr(calc)	ppm	M	Score	Expect	Rank	U	Peptide
16668	12 – 32	1125.5794	2249.1443	2249.1427	0.70	1	73	2.3e-007	1	U	K.DVSQFVETTAEKVEALIGEGK.E

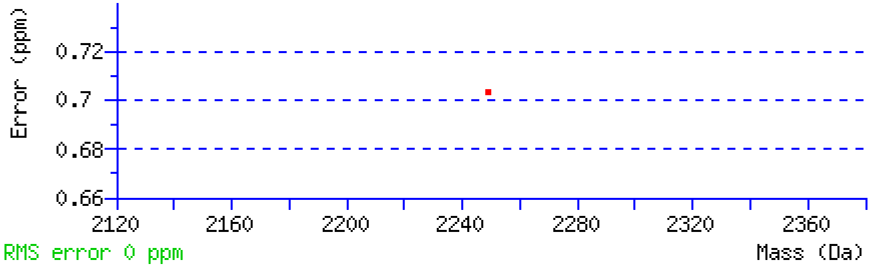

Mascot: http://www.matrixscience.com/

Mascot Search Results

Peptide View

MS/MS Fragmentation of **DVSQFVETTAEKVEALIGEGK**
Found in **RKW29417.1** in **UB_target**, thiol reductase thioredoxin, partial [Granulicatella sp.]

Match to Query 16668: 2249.144282 from(1125.579417,2+) index(22977)
Title: Elution from: 118.413 to 118.413 period: 0 experiment: 1 cycles: 1 precIntensity: 207367.0 FinneganScanNumber: 28511 MStype: enumIsNormalMS rawFile: 19B022_Kuweit_Sample-GA-EVS.raw
Data file LTQ_19B022_Kuweit_Sample-GA-EVS.mgf

Click mouse within plot area to zoom in by factor of two about that point

Or, Plot from 150 to 1100 Da Full range

Show Y-axis

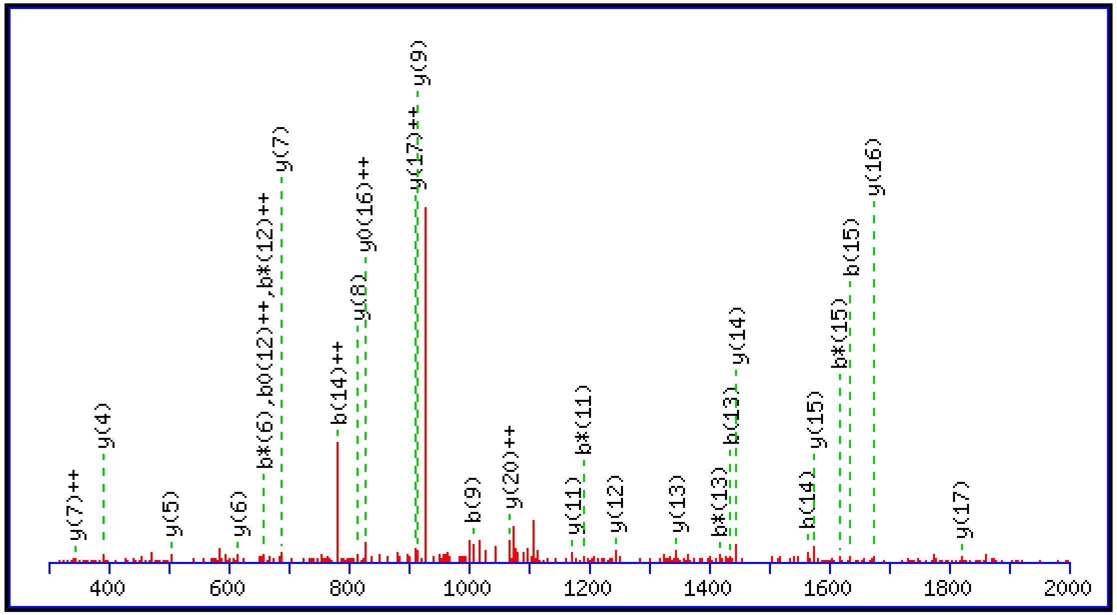

Label all possible matches Label matches used for scoring

Monoisotopic mass of neutral peptide Mr(calc): 2249.1427
Fixed modifications: Carbamidomethyl (C) (apply to specified residues or termini only)
Ions Score: 73 **Expect:** 2.3e-007

Mascot Search Results: Peptide View

Matches : 28/230 fragment ions using 51 most intense peaks (help)

#	b	b^{++}	b^{*}	b^{*++}	b^{0}	b^{0++}	Seq.	y	y^{++}	y^{*}	y^{*++}	y^{0}	y^{0++}	#
1	116.0342	58.5207			98.0237	49.5155	D							21
2	215.1026	108.0550			197.0921	99.0497	V	2135.1230	**1068.0652**	2118.0965	1059.5519	2117.1125	1059.0599	20
3	302.1347	151.5710			284.1241	142.5657	S	2036.0546	1018.5310	2019.0281	1010.0177	2018.0441	1009.5257	19
4	430.1932	215.6003	413.1667	207.0870	412.1827	206.5950	Q	1949.0226	975.0149	1931.9961	966.5017	1931.0120	966.0097	18
5	577.2617	289.1345	560.2351	280.6212	559.2511	280.1292	F	***1820.9640***	**910.9856**	1803.9375	902.4724	1802.9535	901.9804	17
6	676.3301	338.6687	659.3035	330.1554	658.3195	329.6634	V	***1673.8956***	837.4514	1656.8691	828.9382	1655.8850	828.4462	16
7	805.3727	403.1900	788.3461	394.6767	787.3621	394.1847	E	***1574.8272***	787.9172	1557.8006	779.4040	1556.8166	778.9120	15
8	906.4203	453.7138	889.3938	445.2005	888.4098	444.7085	T	***1445.7846***	723.3959	1428.7581	714.8827	1427.7740	714.3907	14
9	**1007.4680**	504.2376	990.4415	495.7244	989.4575	495.2324	T	***1344.7369***	672.8721	1327.7104	664.3588	1326.7264	663.8668	13
10	1078.5051	539.7562	1061.4786	531.2429	1060.4946	530.7509	A	***1243.6892***	622.3483	1226.6627	613.8350	1225.6787	613.3430	12
11	1207.5477	604.2775	1190.5212	595.7642	1189.5372	595.2722	E	***1172.6521***	586.8297	1155.6256	578.3164	1154.6416	577.8244	11
12	1335.6427	668.3250	1318.6161	659.8117	1317.6321	659.3197	K	1043.6095	522.3084	1026.5830	513.7951	1025.5990	513.3031	10
13	**1434.7111**	717.8592	1417.6846	709.3459	1416.7005	708.8539	V	***915.5146***	458.2609	898.4880	449.7477	897.5040	449.2556	9
14	**1563.7537**	782.3805	1546.7271	773.8672	1545.7431	773.3752	E	***816.4462***	408.7267	799.4196	400.2134	798.4356	399.7214	8
15	**1634.7908**	817.8990	1617.7643	809.3858	1616.7802	808.8938	A	***687.4036***	**344.2054**	670.3770	335.6921	669.3930	335.2001	7
16	1747.8749	874.4411	1730.8483	865.9278	1729.8643	865.4358	L	***616.3665***	308.6869	599.3399	300.1736	598.3559	299.6816	6
17	1860.9589	930.9831	1843.9324	922.4698	1842.9484	921.9778	I	***503.2824***	252.1448	486.2558	243.6316	485.2718	243.1395	5
18	1917.9804	959.4938	1900.9539	950.9806	1899.9698	950.4886	G	***390.1983***	195.6028	373.1718	187.0895	372.1878	186.5975	4
19	2047.0230	1024.0151	2029.9964	1015.5019	2029.0124	1015.0099	E	333.1769	167.0921	316.1503	158.5788	315.1663	158.0868	3
20	2104.0445	1052.5259	2087.0179	1044.0126	2086.0339	1043.5206	G	204.1343	102.5708	187.1077	94.0575			2
21							K	147.1128	74.0600	130.0863	65.5468			1

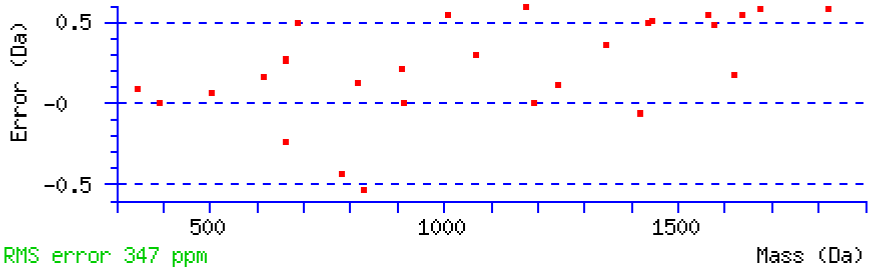

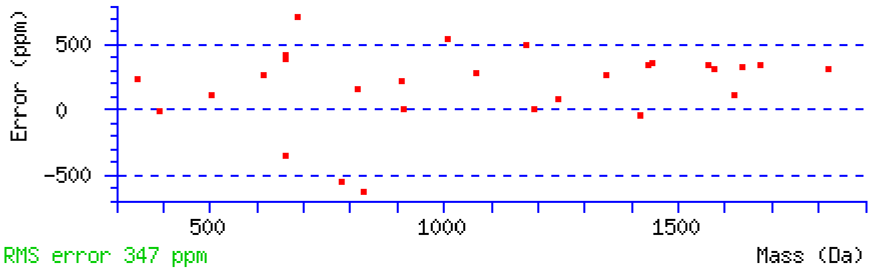

NCBI **BLAST** search of DVSQFVETTAEKVEALIGEGK
(Parameters: blastp, nr protein database, expect=20000, no filter, PAM30)
Other BLAST web gateways

All matches to this query

Score	Mr(calc)	Delta	Sequence
73.0	2249.1427	0.0016	DVSQFVETTAEKVEALIGEGK
4.5	2249.1508	-0.0065	SIMVQGTASDAGKSIIVAGLCR
4.5	2249.1508	-0.0065	SVMIQGTASDAGKSIIVAGLCR
4.4	2249.1395	0.0048	TKMMAAIESAEGVLNAREIAK

Mascot: http://www.matrixscience.com/

MASCOT Search Results

Protein View: WP_005605248.1

extracellular solute-binding protein [Granulicatella adiacens]

**Database:**	UB_target
**Score:**	72
**Monoisotopic mass (M_r):**	39499
**Calculated pI:**	5.19

Sequence similarity is available as an NCBI BLAST search of WP_005605248.1 against nr.

Search parameters

**MS data file:**	LTQ_19B022_Kuweit_Sample-GA-EVS.mgf
**Enzyme:**	Trypsin: cuts C-term side of KR unless next residue is P.
**Fixed modifications:**	Carbamidomethyl (C)
**Variable modifications:**	Deamidated (NQ), Oxidation (M)

Protein sequence coverage: 9%

Matched peptides shown in ***bold red***.

```
  1 MNKTLKKLAL LSVAILGAAC APSTSSSSES TQASSGSSGS SDEKIVIYSN
 51 SVSNGRGDWL KEKAAENGFN IEFVSINGGE LADRVIAEKN NAIADMIFGL
101 NNMEFNRLKD ENLLEKYEPS WKSEVDLSLG DADGLFYPLV VQPLVLIGNE
151 SSTMPKDWTD LAKPEYKGKY NIFKLSGGTS KVILGSIASR YRDDSGELGV
201 SKEGWDVIKG YAQNAKVVAS ETDYIGMIID KKDGIEYNMM WGSGYLQNKN
251 ERKYNFNVMY PEVGEPFVTE QIGILNTSKK KETVQKFINW FGSAEVQAEW
301 SKKFSSIPAN KKALEQANDD VKDFMSKVKS QKLDWEFISK NISSWVEKVE
351 LEFLK
```

Unformatted sequence string: 355 residues (for pasting into other applications).

Sort by	residue number	increasing mass	decreasing mass
Show	matched peptides only	predicted peptides also	

Query	Start – End	Observed	Mr(expt)	Mr(calc)	ppm	M	Score	Expect	Rank	U	Peptide
14961	90 – 107	1042.4862	2082.9579	2082.9615	-1.71	0	68	3.4e-007	1	U	K.NNAIADMIFGLNNMEFNR.L

11162	313 - 327	855.9027	1709.7909	1709.7930	-1.25	1	32	0.0018	1	U	K.ALEQANDDVKDFMSK.V

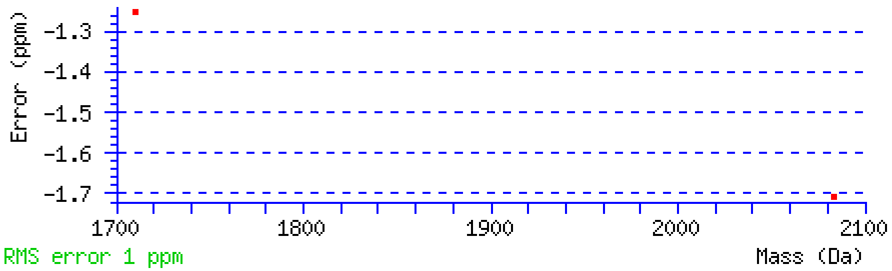

Mascot: http://www.matrixscience.com/

Mascot Search Results

Peptide View

MS/MS Fragmentation of **NNAIADMIFGLNNMEFNR**
Found in **WP_005605248.1** in **UB_target**, extracellular solute-binding protein [Granulicatella adiacens]

Match to Query 14961: 2082.957932 from(1042.486242,2+) index(21632)
Title: Elution from: 112.912 to 112.912 period: 0 experiment: 1 cycles: 1 precIntensity: 270026.0 FinneganScanNumber: 26893 MStype: enumIsNormalMS rawFile: 19B022_Kuweit_Sample-GA-EVS.raw
Data file LTQ_19B022_Kuweit_Sample-GA-EVS.mgf

Click mouse within plot area to zoom in by factor of two about that point
Or, Plot from 100 to 2000 Da Full range
Show Y-axis

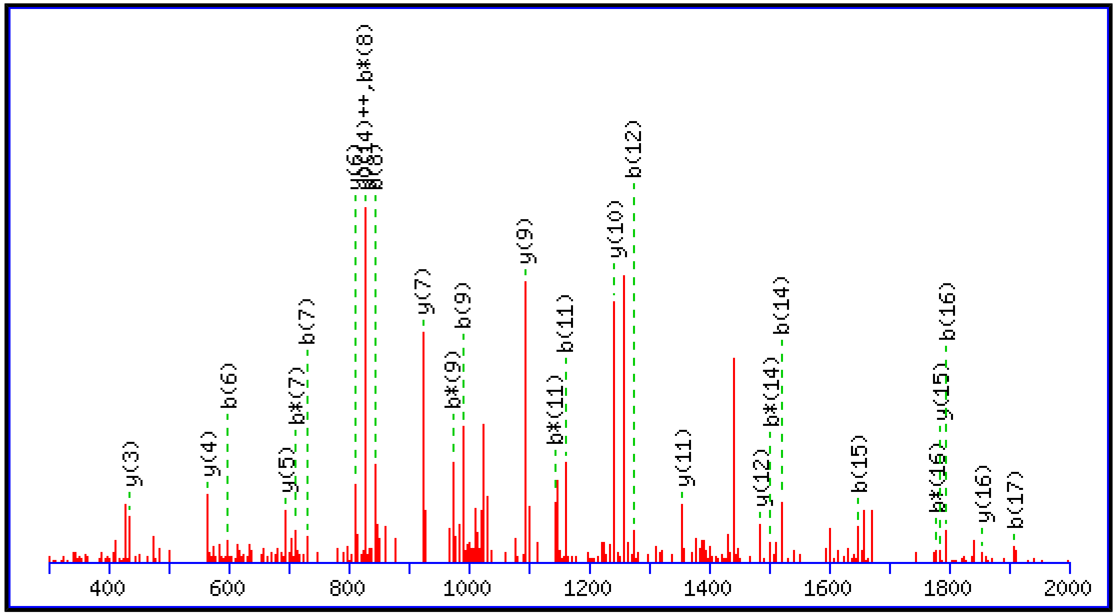

Label all possible matches Label matches used for scoring

Monoisotopic mass of neutral peptide Mr(calc): 2082.9615
Fixed modifications: Carbamidomethyl (C) (apply to specified residues or termini only)
Ions Score: 68 **Expect:** 3.4e-007

Matches : 28/188 fragment ions using 51 most intense peaks (help)

#	b	b^{++}	b*	b^{*++}	b^0	b^{0++}	Seq.	y	y^{++}	y*	y^{*++}	y^0	y^{0++}	#
1	115.0502	58.0287	98.0237	49.5155			N							18
2	229.0931	115.0502	212.0666	106.5369			N	1969.9259	985.4666	1952.8993	976.9533	1951.9153	976.4613	17
3	300.1302	150.5688	283.1037	142.0555			A	1855.8830	928.4451	1838.8564	919.9318	1837.8724	919.4398	16
4	413.2143	207.1108	396.1878	198.5975			I	1784.8458	892.9266	1767.8193	884.4133	1766.8353	883.9213	15
5	484.2514	242.6293	467.2249	234.1161			A	1671.7618	836.3845	1654.7352	827.8713	1653.7512	827.3792	14
6	599.2784	300.1428	582.2518	291.6295	581.2678	291.1375	D	1600.7247	800.8660	1583.6981	792.3527	1582.7141	791.8607	13
7	730.3189	365.6631	713.2923	357.1498	712.3083	356.6578	M	1485.6977	743.3525	1468.6712	734.8392	1467.6872	734.3472	12
8	843.4029	422.2051	826.3764	413.6918	825.3923	413.1998	I	1354.6572	677.8323	1337.6307	669.3190	1336.6467	668.8270	11
9	990.4713	495.7393	973.4448	487.2260	972.4608	486.7340	F	1241.5732	621.2902	1224.5466	612.7769	1223.5626	612.2849	10
10	1047.4928	524.2500	1030.4662	515.7368	1029.4822	515.2448	G	1094.5048	547.7560	1077.4782	539.2427	1076.4942	538.7507	9
11	1160.5769	580.7921	1143.5503	572.2788	1142.5663	571.7868	L	1037.4833	519.2453	1020.4567	510.7320	1019.4727	510.2400	8
12	1274.6198	637.8135	1257.5932	629.3003	1256.6092	628.8082	N	924.3992	462.7033	907.3727	454.1900	906.3887	453.6980	7
13	1388.6627	694.8350	1371.6362	686.3217	1370.6521	685.8297	N	810.3563	405.6818	793.3297	397.1685	792.3457	396.6765	6
14	1519.7032	760.3552	1502.6766	751.8420	1501.6926	751.3500	M	696.3134	348.6603	679.2868	340.1470	678.3028	339.6550	5
15	1648.7458	824.8765	1631.7192	816.3633	1630.7352	815.8712	E	565.2729	283.1401	548.2463	274.6268	547.2623	274.1348	4
16	1795.8142	898.4107	1778.7877	889.8975	1777.8036	889.4055	F	436.2303	218.6188	419.2037	210.1055			3
17	1909.8571	955.4322	1892.8306	946.9189	1891.8466	946.4269	N	289.1619	145.0846	272.1353	136.5713			2
18							R	175.1190	88.0631	158.0924	79.5498			1

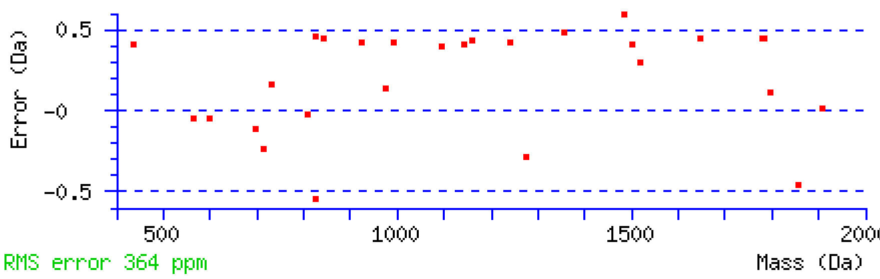

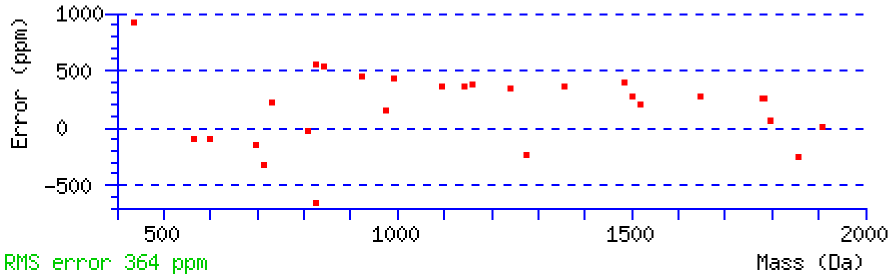

NCBI **BLAST** search of NNAIADMIFGLNNMEFNR
(Parameters: blastp, nr protein database, expect=20000, no filter, PAM30)
Other BLAST web gateways

All matches to this query

Score	Mr(calc)	Delta	Sequence
68.4	2082.9615	-0.0036	NNAIADMIFGLNNMEFNR

Mascot: http://www.matrixscience.com/

MASCOT Search Results

Protein View: WP_005604985.1

YlbF family regulator [Granulicatella]

**Database:**	UB_target
**Score:**	72
**Monoisotopic mass (M_r):**	13326
**Calculated pI:**	4.42

Sequence similarity is available as an NCBI BLAST search of WP_005604985.1 against nr.

Search parameters

**MS data file:**	LTQ_19B022_Kuweit_Sample-GA-EVS.mgf
**Enzyme:**	Trypsin: cuts C-term side of KR unless next residue is P.
**Fixed modifications:**	Carbamidomethyl (C)
**Variable modifications:**	Deamidated (NQ), Oxidation (M)

Protein sequence coverage: 10%

Matched peptides shown in ***bold red***.

```
  1 MSNIYDTANQ LERDLRDLAE FKTVKESFEA IEADETAKAL FDEFRQVNIE
 51 LQQKQYSGQE ITEEDIQKAQ ELGQKVSENE YIKALMEAEQ RLNTIMQDIN
101 RIITNPLQEL YNGK
```

Unformatted sequence string: 114 residues (for pasting into other applications).

Sort by	residue number	increasing mass	decreasing mass
Show	matched peptides only	predicted peptides also	

Query	Start – End	Observed	Mr(expt)	Mr(calc)	ppm	M	Score	Expect	Rank	U	Peptide
8689	2 – 13	712.3441	1422.6736	1422.6739	-0.21	0	49	2e-005	1	U	M.SNIYDTANQLER.D
8690	2 – 13	712.3442	1422.6739	1422.6739	-0.025	0	49	2.7e-005	1	U	M.SNIYDTANQLER.D

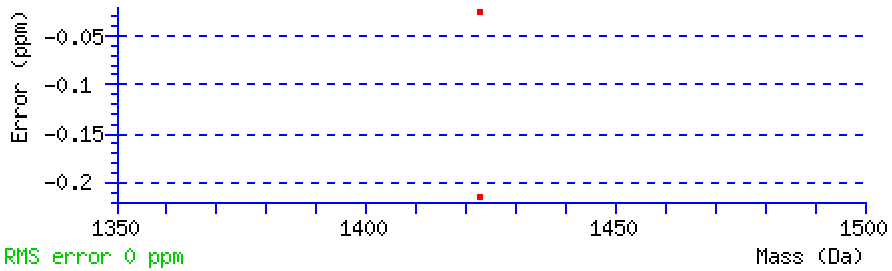

Mascot: http://www.matrixscience.com/

MATRIX SCIENCE Mascot Search Results

Peptide View

MS/MS Fragmentation of **SNIYDTANQLER**
Found in **WP_005604985.1** in **UB_target**, YlbF family regulator [Granulicatella]

Match to Query 8689: 1422.673584 from(712.344068,2+) index(8156)
Title: Elution from: 61.280 to 61.280 period: 0 experiment: 1 cycles: 1 precIntensity: 602795.0 FinneganScanNumber: 10989 MStype: enumIsNormalMS rawFile: 19B022_Kuweit_Sample-GA-EVS.raw
Data file LTQ_19B022_Kuweit_Sample-GA-EVS.mgf

Click mouse within plot area to zoom in by factor of two about that point
Or, Plot from 150 to 1100 Da Full range
Show Y-axis

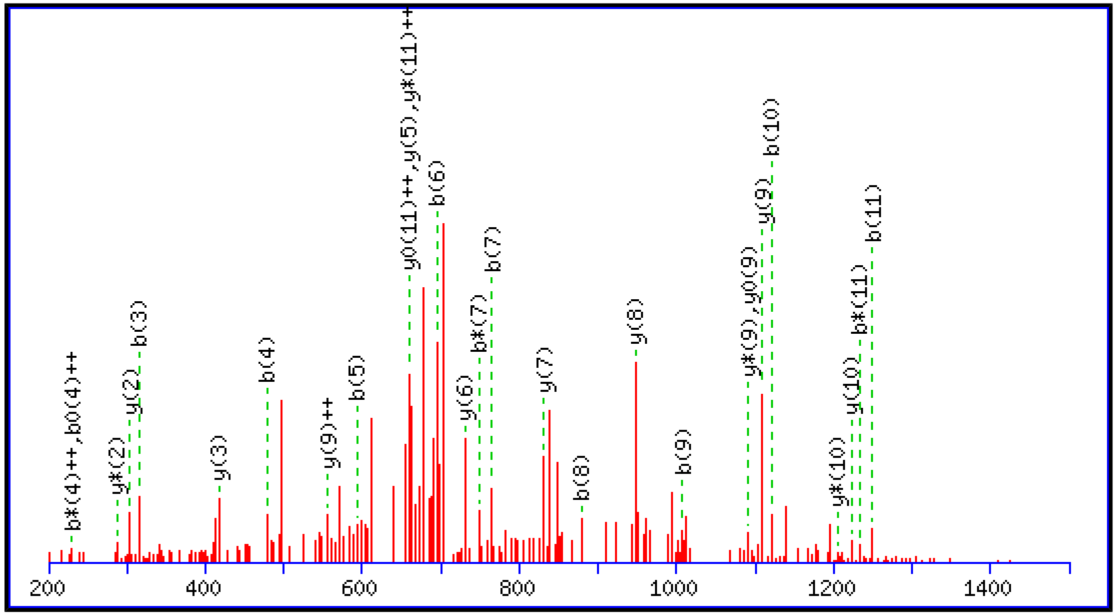

Label all possible matches Label matches used for scoring

Monoisotopic mass of neutral peptide Mr(calc): 1422.6739
Fixed modifications: Carbamidomethyl (C) (apply to specified residues or termini only)
Ions Score: 49 **Expect:** 2e-005

Matches : 28/128 fragment ions using 49 most intense peaks (help)

#	b	b^{++}	b*	b^{*++}	b^0	b^{0++}	Seq.	y	y^{++}	y*	y^{*++}	y^0	y^{0++}	#
1	88.0393	44.5233			70.0287	35.5180	S							12
2	202.0822	101.5448	185.0557	93.0315	184.0717	92.5395	N	1336.6492	668.8282	1319.6226	660.3149	1318.6386	659.8229	11
3	***315.1663***	158.0868	298.1397	149.5735	297.1557	149.0815	I	**1222.6062**	611.8068	1205.5797	603.2935	1204.5957	602.8015	10
4	***478.2296***	239.6185	461.2031	231.1052	460.2191	230.6132	Y	**1109.5222**	555.2647	1092.4956	546.7515	1091.5116	546.2594	9
5	***593.2566***	297.1319	576.2300	288.6186	575.2460	288.1266	D	**946.4588**	473.7331	929.4323	465.2198	928.4483	464.7278	8
6	***694.3042***	347.6558	677.2777	339.1425	676.2937	338.6505	T	**831.4319**	416.2196	814.4054	407.7063	813.4213	407.2143	7
7	***765.3414***	383.1743	748.3148	374.6610	747.3308	374.1690	A	**730.3842**	365.6958	713.3577	357.1825	712.3737	356.6905	6
8	***879.3843***	440.1958	862.3577	431.6825	861.3737	431.1905	N	**659.3471**	330.1772	642.3206	321.6639	641.3365	321.1719	5
9	***1007.4429***	504.2251	990.4163	495.7118	989.4323	495.2198	Q	545.3042	273.1557	528.2776	264.6425	527.2936	264.1504	4
10	***1120.5269***	560.7671	1103.5004	552.2538	1102.5164	551.7618	L	**417.2456**	209.1264	400.2191	200.6132	399.2350	200.1212	3
11	***1249.5695***	625.2884	1232.5430	616.7751	1231.5590	616.2831	E	**304.1615**	152.5844	287.1350	144.0711	286.1510	143.5791	2
12							R	175.1190	88.0631	158.0924	79.5498			1

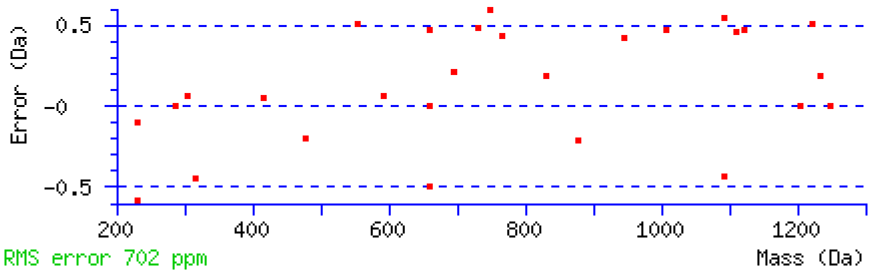

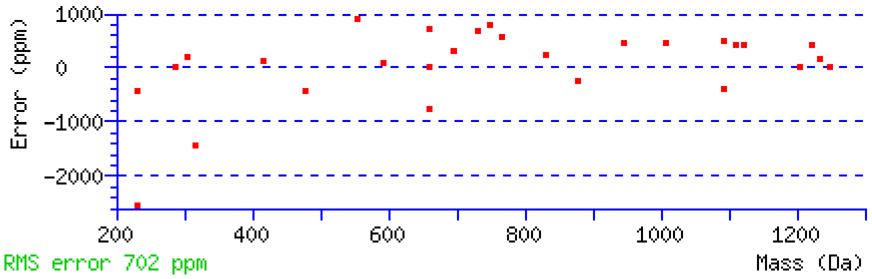

NCBI **BLAST** search of SNIYDTANQLER
(Parameters: blastp, nr protein database, expect=20000, no filter, PAM30)
Other BLAST web gateways

All matches to this query

Score	Mr(calc)	Delta	Sequence
49.2	1422.6739	-0.0003	SNIYDTANQLER
49.2	1422.6739	-0.0003	SNLYDTANQLER
13.2	1422.6739	-0.0003	ANEQFEKVSSQR

9.4	1422.6739	-0.0003	ANEQFEKVSSQR
3.6	1422.6739	-0.0003	ANEQFEKVSSQR

Mascot: http://www.matrixscience.com/

MASCOT Search Results

Protein View: EEW37316.1

cold-shock DNA-binding domain protein [Granulicatella adiacens ATCC 49175 ATCC 49175]

**Database:**	UB_target
**Score:**	65
**Monoisotopic mass (M_r):**	8506
**Calculated pI:**	4.74

Sequence similarity is available as an NCBI BLAST search of EEW37316.1 against nr.

Search parameters

**MS data file:**	LTQ_19B022_Kuweit_Sample-GA-EVS.mgf
**Enzyme:**	Trypsin: cuts C-term side of KR unless next residue is P.
**Fixed modifications:**	Carbamidomethyl (C)
**Variable modifications:**	Deamidated (NQ), Oxidation (M)

Protein sequence coverage: 22%

Matched peptides shown in ***bold red***.

```
 1 MLWHKEEMFM EQGTVKWFNS EKGFGFIERE GGNDVFVHFS AIQSEGFKTL
51 EEGQAVQFDV EEGARGPQAA NVVKL
```

Unformatted sequence string: 75 residues (for pasting into other applications).

Sort by	residue number	increasing mass	decreasing mass
Show	matched peptides only	predicted peptides also	

Query	Start – End	Observed	Mr(expt)	Mr(calc)	ppm	M	Score	Expect	Rank	U	Peptide
12819	49 – 65	939.4455	1876.8765	1876.8803	-2.02	0	65	4.9e-007	1	U	K.TLEEGQAVQFDVEEGAR.G

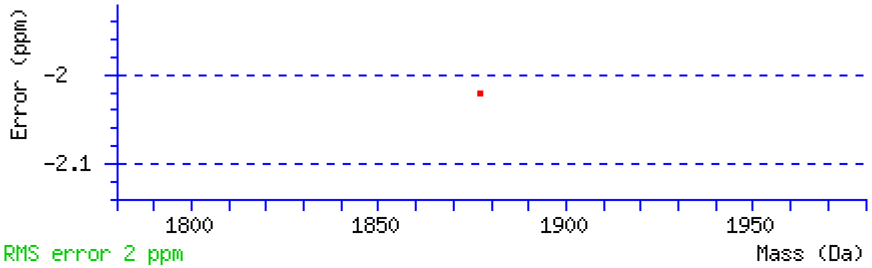

Mascot: http://www.matrixscience.com/

MATRIX SCIENCE Mascot Search Results

Peptide View

MS/MS Fragmentation of **TLEEGQAVQFDVEEGAR**
Found in **EEW37316.1** in **UB_target**, cold-shock DNA-binding domain protein [Granulicatella adiacens ATCC 49175 ATCC 49175]

Match to Query 12819: 1876.876486 from(939.445519,2+) index(12392)
Title: Elution from: 78.102 to 78.102 period: 0 experiment: 1 cycles: 1 precIntensity: 231872.0 FinneganScanNumber: 16081 MStype: enumIsNormalMS rawFile: 19B022_Kuweit_Sample-GA-EVS.raw
Data file LTQ_19B022_Kuweit_Sample-GA-EVS.mgf

Click mouse within plot area to zoom in by factor of two about that point
Or, [Plot from] 150 to 1100 Da [Full range]
Show Y-axis ☐

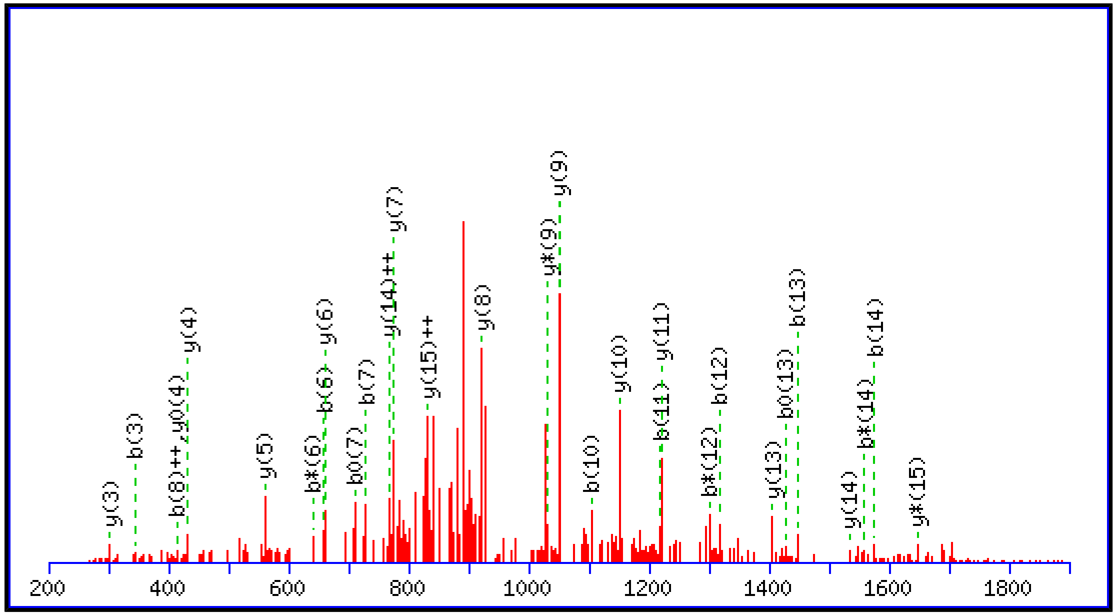

Label all possible matches   Label matches used for scoring

Monoisotopic mass of neutral peptide Mr(calc): 1876.8803
Fixed modifications: Carbamidomethyl (C) (apply to specified residues or termini only)
Ions Score: 65 **Expect:** 4.9e-007

Matches : 30/176 fragment ions using 49 most intense peaks (help)

#	b	b^{++}	b*	b^{*++}	b^{0}	b^{0++}	Seq.	y	y^{++}	y*	y^{*++}	y^{0}	y^{0++}	#
1	102.0550	51.5311			84.0444	42.5258	T							17
2	215.1390	108.0731			197.1285	99.0679	L	1776.8399	888.9236	1759.8133	880.4103	1758.8293	879.9183	16
3	344.1816	172.5944			326.1710	163.5892	E	1663.7558	832.3815	1646.7293	823.8683	1645.7453	823.3763	15
4	473.2242	237.1157			455.2136	228.1105	E	1534.7132	767.8603	1517.6867	759.3470	1516.7027	758.8550	14
5	530.2457	265.6265			512.2351	256.6212	G	1405.6706	703.3390	1388.6441	694.8257	1387.6601	694.3337	13
6	658.3042	329.6558	641.2777	321.1425	640.2937	320.6505	Q	1348.6492	674.8282	1331.6226	666.3149	1330.6386	665.8229	12
7	729.3414	365.1743	712.3148	356.6610	711.3308	356.1690	A	1220.5906	610.7989	1203.5640	602.2857	1202.5800	601.7937	11
8	828.4098	414.7085	811.3832	406.1953	810.3992	405.7032	V	1149.5535	575.2804	1132.5269	566.7671	1131.5429	566.2751	10
9	956.4684	478.7378	939.4418	470.2245	938.4578	469.7325	Q	1050.4851	525.7462	1033.4585	517.2329	1032.4745	516.7409	9
10	1103.5368	552.2720	1086.5102	543.7587	1085.5262	543.2667	F	922.4265	461.7169	905.3999	453.2036	904.4159	452.7116	8
11	1218.5637	609.7855	1201.5372	601.2722	1200.5531	600.7802	D	775.3581	388.1827	758.3315	379.6694	757.3475	379.1774	7
12	1317.6321	659.3197	1300.6056	650.8064	1299.6216	650.3144	V	660.3311	330.6692	643.3046	322.1559	642.3206	321.6639	6
13	1446.6747	723.8410	1429.6482	715.3277	1428.6642	714.8357	E	561.2627	281.1350	544.2362	272.6217	543.2522	272.1297	5
14	1575.7173	788.3623	1558.6908	779.8490	1557.7067	779.3570	E	432.2201	216.6137	415.1936	208.1004	414.2096	207.6084	4
15	1632.7388	816.8730	1615.7122	808.3598	1614.7282	807.8677	G	303.1775	152.0924	286.1510	143.5791			3
16	1703.7759	852.3916	1686.7493	843.8783	1685.7653	843.3863	A	246.1561	123.5817	229.1295	115.0684			2
17							R	175.1190	88.0631	158.0924	79.5498			1

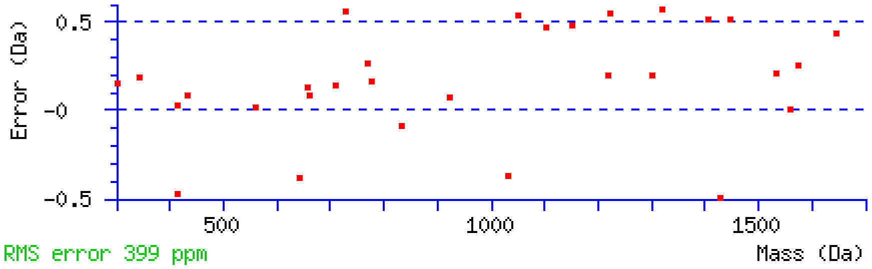

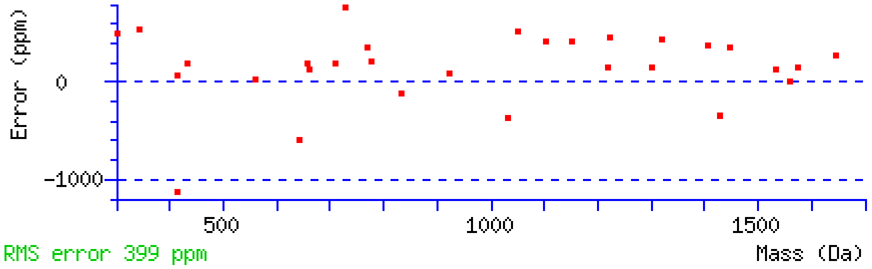

NCBI **BLAST** search of TLEEGQAVQFDVEEGAR
(Parameters: blastp, nr protein database, expect=20000, no filter, PAM30)
Other BLAST web gateways

All matches to this query

Score	Mr(calc)	Delta	Sequence
65.2	1876.8803	-0.0038	TLEEGQAVQFDVEEGAR
2.5	1876.8803	-0.0038	QSDTFNPTAKEPNQTAK

Mascot: http://www.matrixscience.com/

MATRIX SCIENCE MASCOT Search Results

Protein View: WP_005607452.1

30S ribosomal protein S5 [Granulicatella]

**Database:**	UB_target
**Score:**	63
**Monoisotopic mass (M_r):**	17422
**Calculated pI:**	9.60

Sequence similarity is available as an NCBI BLAST search of WP_005607452.1 against nr.

Search parameters

**MS data file:**	LTQ_19B022_Kuweit_Sample-GA-EVS.mgf
**Enzyme:**	Trypsin: cuts C-term side of KR unless next residue is P.
**Fixed modifications:**	Carbamidomethyl (C)
**Variable modifications:**	Deamidated (NQ), Oxidation (M)

Protein sequence coverage: 14%

Matched peptides shown in ***bold red***.

```
  1 MVNVDARNLE LEDRVVAINR VTKVVKGGRR LRFAALVVVG DHNGHVGFGT
 51 GKAQEVPEAI RKAIEDAKKN LIEVPTSGST IPHEVIGRFC GGNVLLKPAQ
101 AGSGIAAGGP VRAVVELAGI SDVTSKSLGS NTPVNMVRAT IEGLKQLKRV
151 EDVAALRGKS VEELLG
```

Unformatted sequence string: 166 residues (for pasting into other applications).

Sort by	residue number	increasing mass	decreasing mass
Show	matched peptides only	predicted peptides also	

Query	Start – End	Observed	Mr(expt)	Mr(calc)	ppm	M	Score	Expect	Rank	U	Peptide
16926	89 – 112	766.4103	2296.2090	2296.2110	-0.87	0	45	0.00012	1	U	R.FCGGNVLLKPAQAGSGIAAGGPVR.A
16927	89 – 112	766.4105	2296.2095	2296.2110	-0.65	0	47	7.6e-005	1	U	R.FCGGNVLLKPAQAGSGIAAGGPVR.A

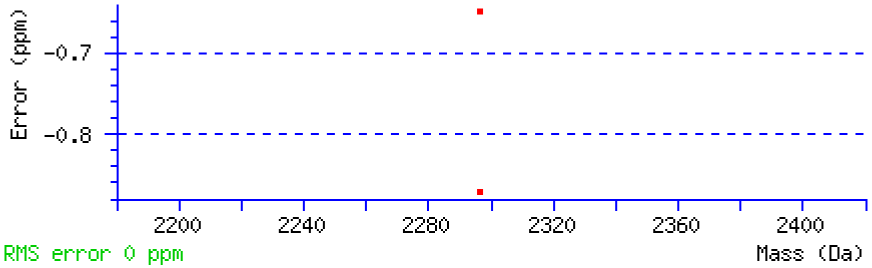

Mascot: http://www.matrixscience.com/

MATRIX SCIENCE Mascot Search Results

Peptide View

MS/MS Fragmentation of **FCGGNVLLKPAQAGSGIAAGGPVR**
Found in **WP_005607452.1** in **UB_target**, 30S ribosomal protein S5 [Granulicatella]

Match to Query 16927: 2296.209537 from(766.410455,3+) index(13569)
Title: Elution from: 82.660 to 82.660 period: 0 experiment: 1 cycles: 1 precIntensity: 1126535.0 FinneganScanNumber: 17475 MStype: enumIsNormalMS rawFile: 19B022_Kuweit_Sample-GA-EVS.raw
Data file LTQ_19B022_Kuweit_Sample-GA-EVS.mgf

Click mouse within plot area to zoom in by factor of two about that point
Or, Plot from 100 to 2000 Da Full range
Show Y-axis

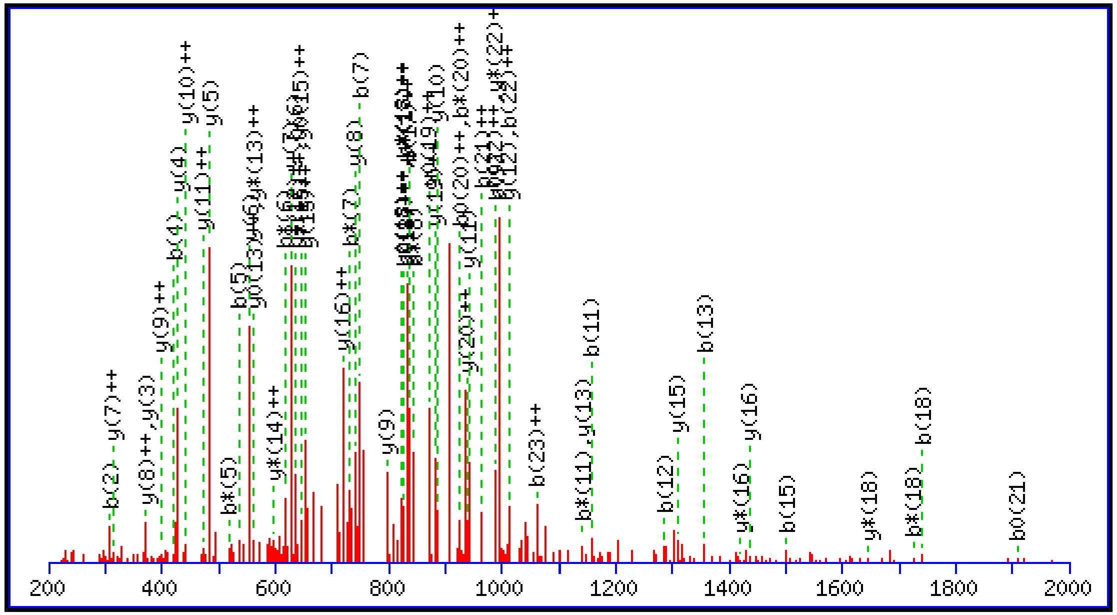

Label all possible matches Label matches used for scoring

Monoisotopic mass of neutral peptide Mr(calc): 2296.2110
Fixed modifications: Carbamidomethyl (C) (apply to specified residues or termini only)
Ions Score: 47 **Expect:** 7.6e-005

Matches : 62/222 fragment ions using 137 most intense peaks (help)

#	b	b^{++}	b^{*}	b^{*++}	b^{0}	b^{0++}	Seq.	y	y^{++}	y^{*}	y^{*++}	y^{0}	y^{0++}	#
1	148.0757	74.5415					F							24
2	308.1063	154.5568					C	2150.1499	1075.5786	2133.1233	1067.0653	2132.1393	1066.5733	23
3	365.1278	183.0675					G	1990.1192	995.5633	1973.0927	987.0500	1972.1087	986.5580	22
4	422.1493	211.5783					G	1933.0978	967.0525	1916.0712	958.5392	1915.0872	958.0472	21
5	536.1922	268.5997	519.1656	260.0865			N	1876.0763	938.5418	1859.0498	930.0285	1858.0657	929.5365	20
6	635.2606	318.1339	618.2341	309.6207			V	1762.0334	881.5203	1745.0068	873.0071	1744.0228	872.5150	19
7	748.3447	374.6760	731.3181	366.1627			L	1662.9650	831.9861	1645.9384	823.4728	1644.9544	822.9808	18
8	861.4287	431.2180	844.4022	422.7047			L	1549.8809	775.4441	1532.8544	766.9308	1531.8703	766.4388	17
9	989.5237	495.2655	972.4971	486.7522			K	1436.7968	718.9021	1419.7703	710.3888	1418.7863	709.8968	16
10	1086.5765	543.7919	1069.5499	535.2786			P	1308.7019	654.8546	1291.6753	646.3413	1290.6913	645.8493	15
11	1157.6136	579.3104	1140.5870	570.7972			A	1211.6491	606.3282	1194.6226	597.8149	1193.6385	597.3229	14
12	1285.6722	643.3397	1268.6456	634.8264			Q	1140.6120	570.8096	1123.5854	562.2964	1122.6014	561.8044	13
13	1356.7093	678.8583	1339.6827	670.3450			A	1012.5534	506.7803	995.5269	498.2671	994.5429	497.7751	12
14	1413.7307	707.3690	1396.7042	698.8557			G	941.5163	471.2618	924.4898	462.7485	923.5057	462.2565	11
15	1500.7628	750.8850	1483.7362	742.3717	1482.7522	741.8797	S	884.4948	442.7511	867.4683	434.2378	866.4843	433.7458	10
16	1557.7842	779.3958	1540.7577	770.8825	1539.7737	770.3905	G	797.4628	399.2350	780.4363	390.7218			9
17	1670.8683	835.9378	1653.8417	827.4245	1652.8577	826.9325	I	740.4413	370.7243	723.4148	362.2110			8
18	1741.9054	871.4563	1724.8789	862.9431	1723.8948	862.4511	A	627.3573	314.1823	610.3307	305.6690			7
19	1812.9425	906.9749	1795.9160	898.4616	1794.9320	897.9696	A	556.3202	278.6637	539.2936	270.1504			6
20	1869.9640	935.4856	1852.9374	926.9724	1851.9534	926.4803	G	485.2831	243.1452	468.2565	234.6319			5
21	1926.9854	963.9964	1909.9589	955.4831	1908.9749	954.9911	G	428.2616	214.6344	411.2350	206.1212			4
22	2024.0382	1012.5227	2007.0117	1004.0095	2006.0276	1003.5175	P	371.2401	186.1237	354.2136	177.6104			3
23	2123.1066	1062.0569	2106.0801	1053.5437	2105.0961	1053.0517	V	274.1874	137.5973	257.1608	129.0840			2
24							R	175.1190	88.0631	158.0924	79.5498			1

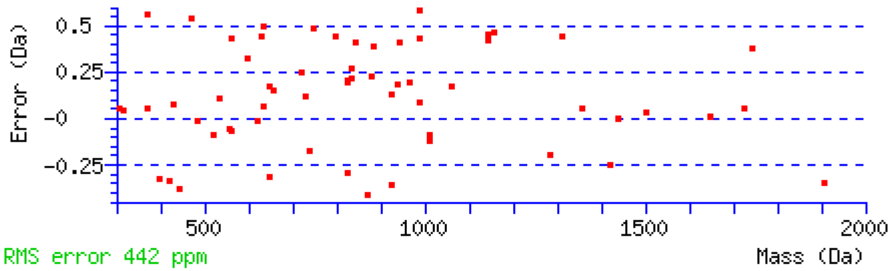

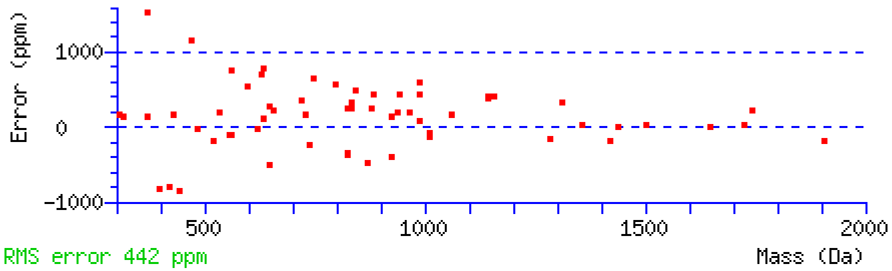

NCBI **BLAST** search of FCGGNVLLKPAQAGSGIAAGGPVR
(Parameters: blastp, nr protein database, expect=20000, no filter, PAM30)
Other BLAST web gateways

All matches to this query

Score	Mr(calc)	Delta	Sequence
47.5	2296.2110	-0.0015	FCGGNVLLKPAQAGSGIAAGGPVR

Mascot: http://www.matrixscience.com/

MASCOT Search Results

Protein View: RKW29368.1

hypothetical protein [Granulicatella sp.]

**Database:**	UB_target
**Score:**	60
**Monoisotopic mass (M_r):**	27190
**Calculated pI:**	5.80

Sequence similarity is available as an NCBI BLAST search of RKW29368.1 against nr.

Search parameters

**MS data file:**	LTQ_19B022_Kuweit_Sample-GA-EVS.mgf
**Enzyme:**	Trypsin: cuts C-term side of KR unless next residue is P.
**Fixed modifications:**	Carbamidomethyl (C)
**Variable modifications:**	Deamidated (NQ), Oxidation (M)

Protein sequence coverage: 6%

Matched peptides shown in ***bold red***.

```
  1 MIKQYLKPLL IAGCSLLMLA GCGNQTATTT TTSTAASSNP LEGKWEQIDF
 51 RSTLERGLGY LDFQDKEEIP RRLIYSDAFK DVKPTLTITG NSAVYEYTTS
101 IEAAMGNFYD YAKSKNLTSV KGTKEEYIKN QYNVLKQSLE KWNNQKGYLT
151 YGFNDEKNEV QETLTGITIN EGTGTMEFKY APNFLSLATF SVEKFVKPVS
201 YKYSIEDGIL TLTLEQQKIL DNDQKVTAYF TMRFKKVAE
```

Unformatted sequence string: 239 residues (for pasting into other applications).

Sort by	residue number	increasing mass	decreasing mass
Show	matched peptides only	predicted peptides also	

Query	Start – End	Observed	Mr(expt)	Mr(calc)	ppm	M	Score	Expect	Rank	U	Peptide
10952	180 – 194	843.9403	1685.8661	1685.8665	-0.19	0	60	3.4e-006	1	U	K.YAPNFLSLATFSVEK.F

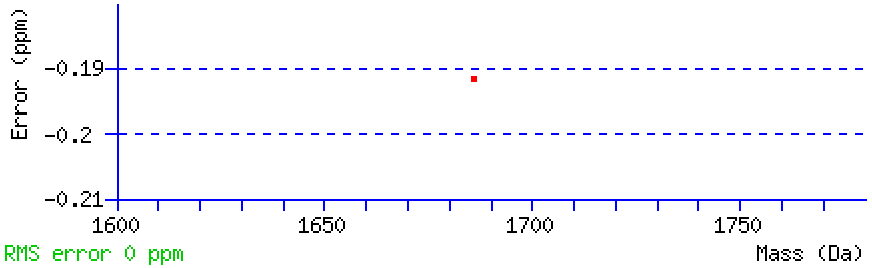

Mascot: http://www.matrixscience.com/

Mascot Search Results

Peptide View

MS/MS Fragmentation of **YAPNFLSLATFSVEK**
Found in **RKW29368.1** in **UB_target**, hypothetical protein [Granulicatella sp.]

Match to Query 10952: 1685.866132 from(843.940342,2+) index(20474)
Title: Elution from: 108.762 to 108.762 period: 0 experiment: 1 cycles: 1 precIntensity: 309380.0 FinneganScanNumber: 25593 MStype: enumIsNormalMS rawFile: 19B022_Kuweit_Sample-GA-EVS.raw
Data file LTQ_19B022_Kuweit_Sample-GA-EVS.mgf

Click mouse within plot area to zoom in by factor of two about that point

Or, Plot from 150 to 1100 Da Full range

Show Y-axis

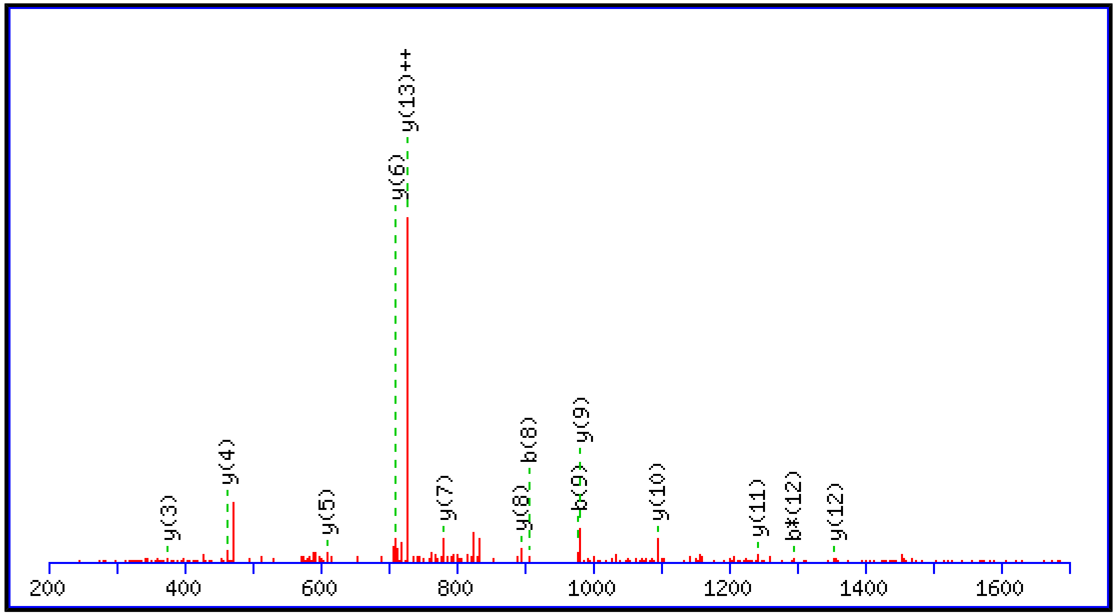

Label all possible matches Label matches used for scoring

Monoisotopic mass of neutral peptide Mr(calc): 1685.8665
Fixed modifications: Carbamidomethyl (C) (apply to specified residues or termini only)
Ions Score: 60 **Expect:** 3.4e-006

Matches : 14/148 fragment ions using 30 most intense peaks (help)

#	b	b^{++}	b*	b^{*++}	b^{0}	b^{0++}	Seq.	y	y^{++}	y*	y^{*++}	y^{0}	y^{0++}	#
1	164.0706	82.5389					Y							15
2	235.1077	118.0575					A	1523.8104	762.4088	1506.7839	753.8956	1505.7999	753.4036	14
3	332.1605	166.5839					P	1452.7733	726.8903	1435.7468	718.3770	1434.7627	717.8850	13
4	446.2034	223.6053	429.1769	215.0921			N	***1355.7205***	678.3639	1338.6940	669.8506	1337.7100	669.3586	12
5	593.2718	297.1396	576.2453	288.6263			F	***1241.6776***	621.3424	1224.6511	612.8292	1223.6671	612.3372	11
6	706.3559	353.6816	689.3293	345.1683			L	***1094.6092***	547.8082	1077.5827	539.2950	1076.5986	538.8030	10
7	793.3879	397.1976	776.3614	388.6843	775.3774	388.1923	S	***981.5251***	491.2662	964.4986	482.7529	963.5146	482.2609	9
8	**906.4720**	453.7396	889.4454	445.2264	888.4614	444.7343	L	***894.4931***	447.7502	877.4666	439.2369	876.4825	438.7449	8
9	**977.5091**	489.2582	960.4825	480.7449	959.4985	480.2529	A	***781.4090***	391.2082	764.3825	382.6949	763.3985	382.2029	7
10	1078.5568	539.7820	1061.5302	531.2688	1060.5462	530.7767	T	***710.3719***	355.6896	693.3454	347.1763	692.3614	346.6843	6
11	1225.6252	613.3162	1208.5986	604.8030	1207.6146	604.3109	F	***609.3243***	305.1658	592.2977	296.6525	591.3137	296.1605	5
12	1312.6572	656.8322	1295.6307	648.3190	1294.6467	647.8270	S	***462.2558***	231.6316	445.2293	223.1183	444.2453	222.6263	4
13	1411.7256	706.3665	1394.6991	697.8532	1393.7151	697.3612	V	***375.2238***	188.1155	358.1973	179.6023	357.2132	179.1103	3
14	1540.7682	770.8877	1523.7417	762.3745	1522.7577	761.8825	E	276.1554	138.5813	259.1288	130.0681	258.1448	129.5761	2
15							K	147.1128	74.0600	130.0863	65.5468			1

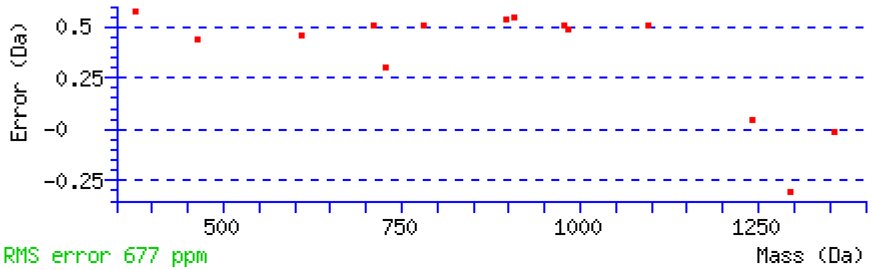

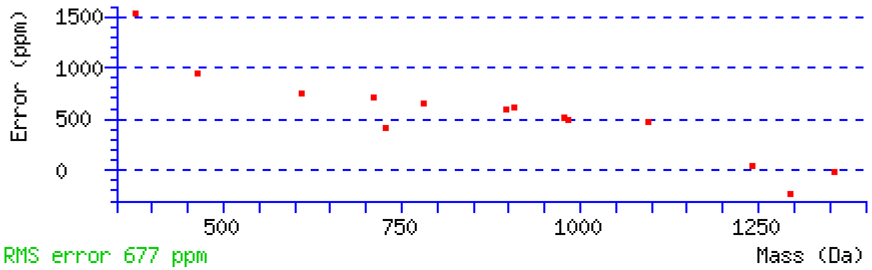

NCBI **BLAST** search of YAPNFLSLATFSVEK
(Parameters: blastp, nr protein database, expect=20000, no filter, PAM30)
Other BLAST web gateways

All matches to this query

Score	Mr(calc)	Delta	Sequence

60.1	1685.8665	-0.0003	YAPNFLSLATFSVEK
1.0	1685.8658	0.0003	LAGVMAGELGEDVNLAK
0.4	1685.8658	0.0004	MTQNPALVQLKENAK
0.4	1685.8658	0.0004	MTQNPALVQLKENAK

Mascot: http://www.matrixscience.com/

MATRIX SCIENCE MASCOT Search Results

Protein View: WP_005605054.1

ROK family glucokinase [Granulicatella]

**Database:**	UB_target
**Score:**	58
**Monoisotopic mass (M_r):**	34495
**Calculated pI:**	4.92

Sequence similarity is available as an NCBI BLAST search of WP_005605054.1 against nr.

Search parameters

**MS data file:**	LTQ_19B022_Kuweit_Sample-GA-EVS.mgf
**Enzyme:**	Trypsin: cuts C-term side of KR unless next residue is P.
**Fixed modifications:**	Carbamidomethyl (C)
**Variable modifications:**	Deamidated (NQ), Oxidation (M)

Protein sequence coverage: 7%

Matched peptides shown in ***bold red***.

```
  1 MEKKIIGIDL GGTSIKFAII SLEGEVQQKW SIPTNILDEG SHIVEDIIES
 51 IRHRLELLGL TNENFAGIGM GSPGVVDREN GTVIGAYNLN WKTLQPLKEK
101 IEGALSLPFF IDNDANVAAL GEKWMGAGGD QPDVTFFTLG TGVGGGIIAE
151 NRLIHGVAGA GGELGHITVD FSDRPFACTC GKKGCLETVA SATGIVNLAR
201 RYADEYAGDS ELKARIDDGQ DVSAKDVFDL AKQNDPLALI VIRHFSKYLG
251 IACSHVANML NPSFIVIGGG VSAAGEFLLE GVRKEYEQLV FPQVRETTHL
301 RLAELGNDAG VIGAASLVLL KD
```

Unformatted sequence string: 322 residues (for pasting into other applications).

Sort by	residue number	increasing mass	decreasing mass
Show	matched peptides only	predicted peptides also	

Query	Start – End	Observed	Mr(expt)	Mr(calc)	ppm	M	Score	Expect	Rank	U	Peptide
17694	101 – 123	1202.6261	2403.2376	2403.2322	2.26	0	58	8.3e-006	1	U	K.IEGALSLPFFIDNDANVAALGEK.W

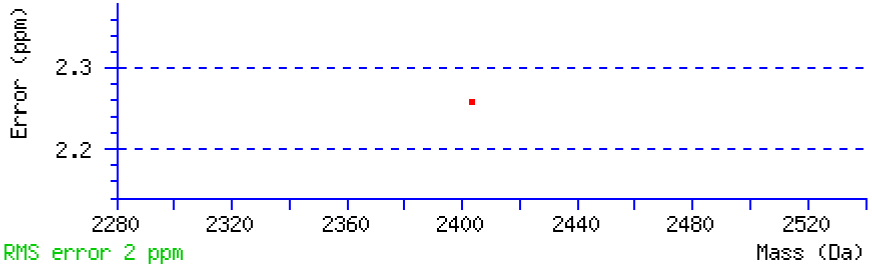

Mascot: http://www.matrixscience.com/

MATRIX SCIENCE Mascot Search Results

Peptide View

MS/MS Fragmentation of **IEGALSLPFFIDNDANVAALGEK**
Found in **WP_005605054.1** in **UB_target**, ROK family glucokinase [Granulicatella]

Match to Query 17694: 2403.237588 from(1202.626070,2+) index(21153)
Title: Elution from: 111.073 to 111.073 period: 0 experiment: 1 cycles: 1 precIntensity: 193377.0 FinneganScanNumber: 26336 MStype: enumIsNormalMS rawFile: 19B022_Kuweit_Sample-GA-EVS.raw
Data file LTQ_19B022_Kuweit_Sample-GA-EVS.mgf

Click mouse within plot area to zoom in by factor of two about that point
Or, [Plot from] 100 to 2000 Da [Full range]
Show Y-axis ☐

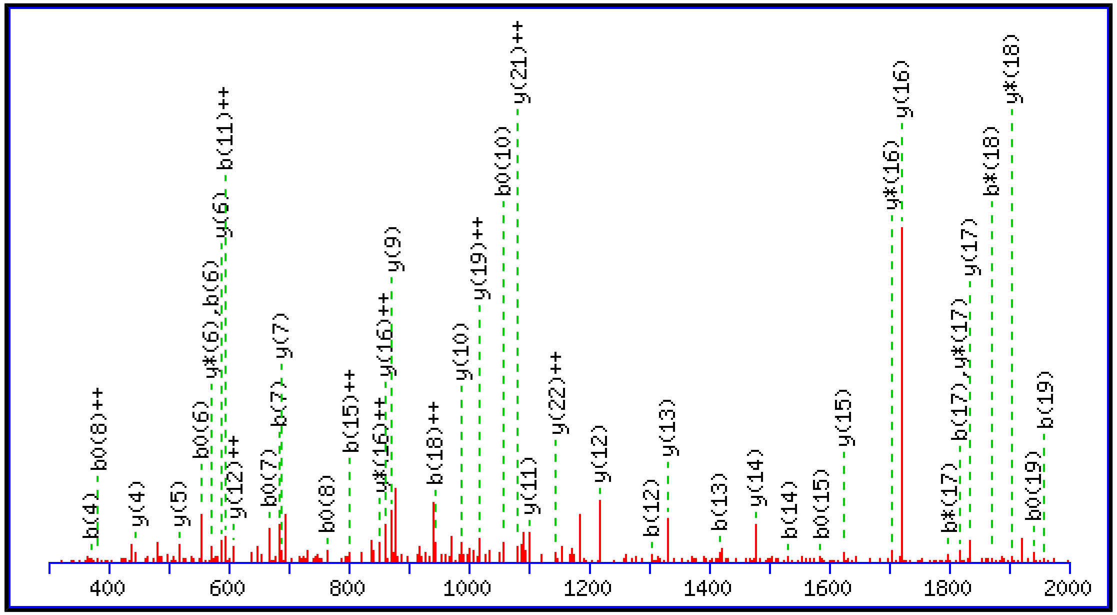

Label all possible matches   Label matches used for scoring

Monoisotopic mass of neutral peptide Mr(calc): 2403.2322
Fixed modifications: Carbamidomethyl (C) (apply to specified residues or termini only)
Ions Score: 58 **Expect:** 8.3e-006

Matches : 43/236 fragment ions using 85 most intense peaks (help)

#	b	b^{++}	b*	b^{*++}	b^0	b^{0++}	Seq.	y	y^{++}	y*	y^{*++}	y^0	y^{0++}	#
1	114.0913	57.5493					I							23
2	243.1339	122.0706			225.1234	113.0653	E	2291.1554	**1146.0813**	2274.1289	1137.5681	2273.1448	1137.0761	22
3	300.1554	150.5813			282.1448	141.5761	G	2162.1128	**1081.5600**	2145.0863	1073.0468	2144.1022	1072.5548	21
4	**371.1925**	186.0999			353.1819	177.0946	A	2105.0913	1053.0493	2088.0648	1044.5360	2087.0808	1044.0440	20
5	484.2766	242.6419			466.2660	233.6366	L	2034.0542	**1017.5308**	2017.0277	1009.0175	2016.0437	1008.5255	19
6	**571.3086**	286.1579			553.2980	277.1527	S	1920.9702	960.9887	1903.9436	952.4754	1902.9596	951.9834	18
7	**684.3927**	342.7000			666.3821	333.6947	L	***1833.9381***	917.4727	1816.9116	908.9594	1815.9276	908.4674	17
8	781.4454	391.2264			763.4349	382.2211	P	***1720.8541***	**860.9307**	1703.8275	852.4174	1702.8435	851.9254	16
9	928.5138	464.7606			910.5033	455.7553	F	***1623.8013***	812.4043	1606.7748	803.8910	1605.7907	803.3990	15
10	1075.5823	538.2948			1057.5717	529.2895	F	***1476.7329***	738.8701	1459.7063	730.3568	1458.7223	729.8648	14
11	1188.6663	594.8368			1170.6558	585.8315	I	***1329.6645***	665.3359	1312.6379	656.8226	1311.6539	656.3306	13
12	**1303.6933**	652.3503			1285.6827	643.3450	D	***1216.5804***	**608.7938**	1199.5539	600.2806	1198.5699	599.7886	12
13	**1417.7362**	709.3717	1400.7096	700.8585	1399.7256	700.3665	N	***1101.5535***	551.2804	1084.5269	542.7671	1083.5429	542.2751	11
14	**1532.7631**	766.8852	1515.7366	758.3719	1514.7526	757.8799	D	***987.5106***	494.2589	970.4840	485.7456	969.5000	485.2536	10
15	1603.8003	802.4038	1586.7737	793.8905	1585.7897	793.3985	A	***872.4836***	436.7454	855.4571	428.2322	854.4730	427.7402	9
16	1717.8432	859.4252	1700.8166	850.9120	1699.8326	850.4199	N	801.4465	401.2269	784.4199	392.7136	783.4359	392.2216	8
17	**1816.9116**	908.9594	1799.8850	900.4462	1798.9010	899.9542	V	***687.4036***	344.2054	670.3770	335.6921	669.3930	335.2001	7
18	1887.9487	944.4780	1870.9222	935.9647	1869.9381	935.4727	A	***588.3352***	294.6712	571.3086	286.1579	570.3246	285.6659	6
19	**1958.9858**	979.9965	1941.9593	971.4833	1940.9753	970.9913	A	***517.2980***	259.1527	500.2715	250.6394	499.2875	250.1474	5
20	2072.0699	1036.5386	2055.0433	1028.0253	2054.0593	1027.5333	L	***446.2609***	223.6341	429.2344	215.1208	428.2504	214.6288	4
21	2129.0913	1065.0493	2112.0648	1056.5360	2111.0808	1056.0440	G	333.1769	167.0921	316.1503	158.5788	315.1663	158.0868	3
22	2258.1339	1129.5706	2241.1074	1121.0573	2240.1234	1120.5653	E	276.1554	138.5813	259.1288	130.0681	258.1448	129.5761	2
23							K	147.1128	74.0600	130.0863	65.5468			1

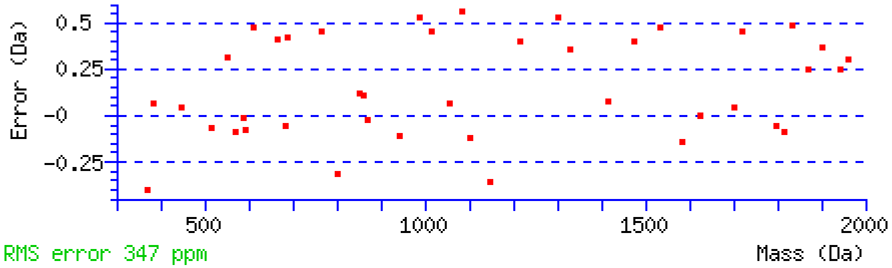

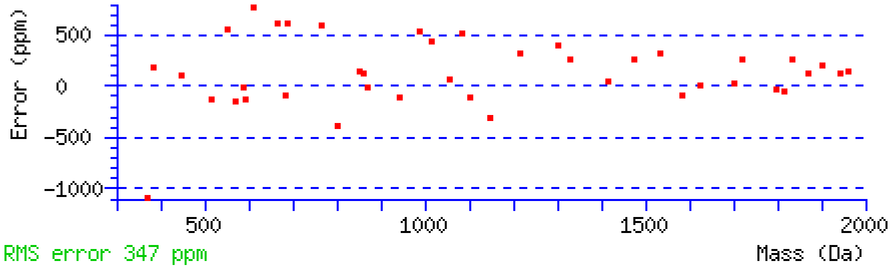

NCBI **BLAST** search of IEGALSLPFFIDNDANVAALGEK
(Parameters: blastp, nr protein database, expect=20000, no filter, PAM30)
Other BLAST web gateways

All matches to this query

Score	Mr(calc)	Delta	Sequence
57.8	2403.2322	0.0054	IEGALSLPFFIDNDANVAALGEK

Mascot: http://www.matrixscience.com/

MASCOT Search Results

Protein View: EEW38033.1

putative enoyl-[acyl-carrier-protein] reductase II [Granulicatella adiacens ATCC 49175 ATCC 49175]

**Database:**	UB_target
**Score:**	55
**Monoisotopic mass (M_r):**	32826
**Calculated pI:**	5.29

Sequence similarity is available as an NCBI BLAST search of EEW38033.1 against nr.

Search parameters

**MS data file:**	LTQ_19B022_Kuweit_Sample-GA-EVS.mgf
**Enzyme:**	Trypsin: cuts C-term side of KR unless next residue is P.
**Fixed modifications:**	Carbamidomethyl (C)
**Variable modifications:**	Deamidated (NQ), Oxidation (M)

Protein sequence coverage: 9%

Matched peptides shown in ***bold red***.

```
  1 MIGIEYPIIQ GGMAWVANPA LASAVSNAGG LGIVACGHAP GEVVKGFIEE
 51 MNRLTDKPYG VNIMLLSPFV DEVVDVVCQA GVKVVCTGAG SPGKYMAKFK
101 EAGITVIPVV ASVALAKRME KEGADAIVVE GMEAGGHIGK STTMALLPQV
151 VDAVSVPVIG AGGIGDGRGM AAALMLGADA VQLGTRFLVS TECTAHDNFK
201 ASVLKAKDID TVITGQITGH PVRVLRNKLT KIYLQAEKEE TSKENPDFER
251 LEEIGRGALR RIVVEGDTQM GSMMAGQIAG LISKEQSCSE IIQELMAESH
301 EVIQKELARF
```

Unformatted sequence string: 310 residues (for pasting into other applications).

Sort by	residue number	increasing mass	decreasing mass
Show	matched peptides only	predicted peptides also	

Query	Start – End	Observed	Mr(expt)	Mr(calc)	ppm	M	Score	Expect	Rank	U	Peptide
19361	141 – 168	1340.7180	2679.4214	2679.4266	-1.95	0	55	1.3e-005	1	U	K.STTMALLPQVVDAVSVPVIGAGGIGDGR.G

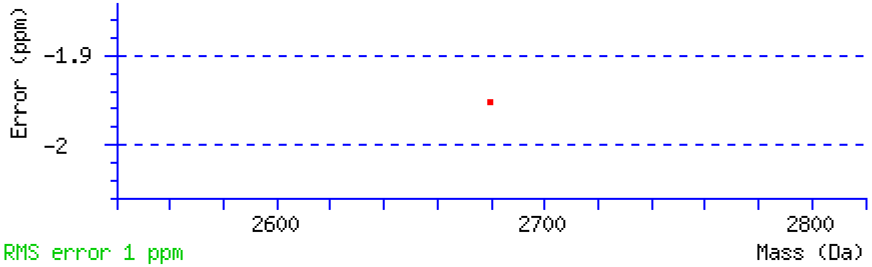

Mascot: http://www.matrixscience.com/

MATRIX SCIENCE Mascot Search Results

Peptide View

MS/MS Fragmentation of **STTMALLPQVVDAVSVPVIGAGGIGDGR**
Found in **EEW38033.1** in **UB_target**, putative enoyl-[acyl-carrier-protein] reductase II [Granulicatella adiacens ATCC 49175 ATCC 49175]

Match to Query 19361: 2679.421358 from(1340.717955,2+) index(21338)
Title: Elution from: 111.732 to 111.732 period: 0 experiment: 1 cycles: 1 precIntensity: 162463.0 FinneganScanNumber: 26544 MStype: enumIsNormalMS rawFile: 19B022_Kuweit_Sample-GA-EVS.raw
Data file LTQ_19B022_Kuweit_Sample-GA-EVS.mgf

Click mouse within plot area to zoom in by factor of two about that point
Or, Plot from 150 to 1100 Da Full range
Show Y-axis

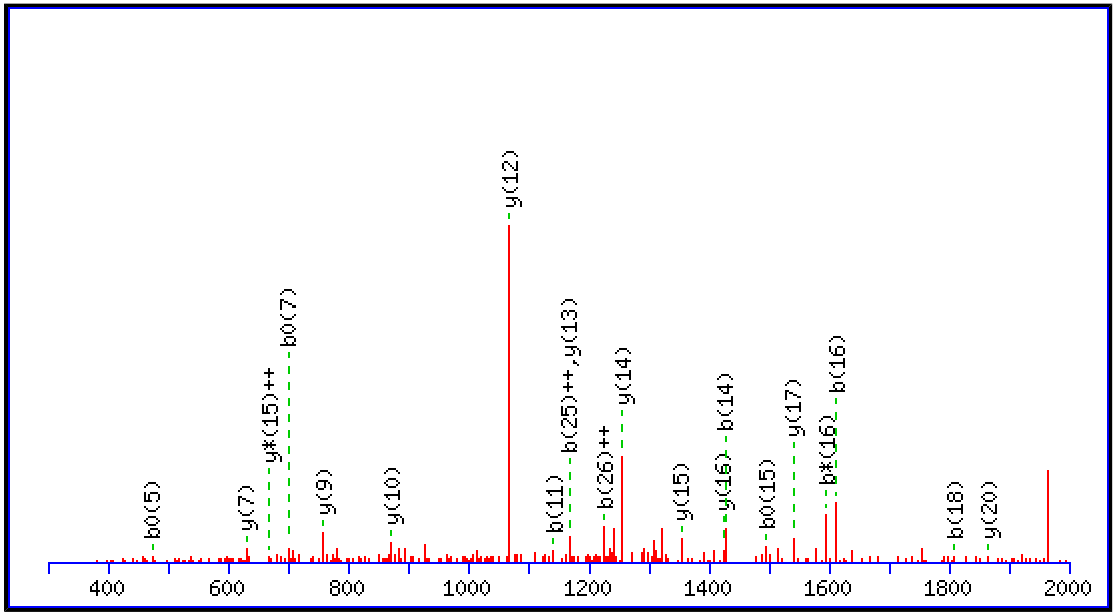

Label all possible matches Label matches used for scoring

Monoisotopic mass of neutral peptide Mr(calc): 2679.4266
Fixed modifications: Carbamidomethyl (C) (apply to specified residues or termini only)
Ions Score: 55 **Expect:** 1.3e-005

Matches : 21/304 fragment ions using 34 most intense peaks (help)

#	b	b^{++}	b^{*}	b^{*++}	b^{0}	b^{0++}	Seq.	y	y^{++}	y^{*}	y^{*++}	y^{0}	y^{0++}	#
1	88.0393	44.5233			70.0287	35.5180	S							28
2	189.0870	95.0471			171.0764	86.0418	T	2593.4018	1297.2045	2576.3753	1288.6913	2575.3912	1288.1993	27
3	290.1347	145.5710			272.1241	136.5657	T	2492.3541	1246.6807	2475.3276	1238.1674	2474.3436	1237.6754	26
4	421.1751	211.0912			403.1646	202.0859	M	2391.3064	1196.1569	2374.2799	1187.6436	2373.2959	1187.1516	25
5	492.2123	246.6098			474.2017	237.6045	A	2260.2660	1130.6366	2243.2394	1122.1233	2242.2554	1121.6313	24
6	605.2963	303.1518			587.2858	294.1465	L	2189.2288	1095.1181	2172.2023	1086.6048	2171.2183	1086.1128	23
7	718.3804	359.6938			700.3698	350.6886	L	2076.1448	1038.5760	2059.1182	1030.0628	2058.1342	1029.5707	22
8	815.4332	408.2202			797.4226	399.2149	P	1963.0607	982.0340	1946.0342	973.5207	1945.0502	973.0287	21
9	943.4917	472.2495	926.4652	463.7362	925.4812	463.2442	Q	***1866.0080***	933.5076	1848.9814	924.9943	1847.9974	924.5023	20
10	1042.5601	521.7837	1025.5336	513.2704	1024.5496	512.7784	V	1737.9494	869.4783	1720.9228	860.9651	1719.9388	860.4730	19
11	**1141.6286**	571.3179	1124.6020	562.8046	1123.6180	562.3126	V	1638.8810	819.9441	1621.8544	811.4308	1620.8704	810.9388	18
12	1256.6555	628.8314	1239.6290	620.3181	1238.6449	619.8261	D	***1539.8125***	770.4099	1522.7860	761.8966	1521.8020	761.4046	17
13	1327.6926	664.3499	1310.6661	655.8367	1309.6821	655.3447	A	***1424.7856***	712.8964	1407.7591	704.3832	1406.7750	703.8912	16
14	**1426.7610**	713.8842	1409.7345	705.3709	1408.7505	704.8789	V	***1353.7485***	677.3779	1336.7219	668.8646	1335.7379	668.3726	15
15	1513.7931	757.4002	1496.7665	748.8869	1495.7825	748.3949	S	***1254.6801***	627.8437	1237.6535	619.3304	1236.6695	618.8384	14
16	**1612.8615**	806.9344	1595.8349	798.4211	1594.8509	797.9291	V	***1167.6480***	584.3277	1150.6215	575.8144	1149.6375	575.3224	13
17	1709.9142	855.4608	1692.8877	846.9475	1691.9037	846.4555	P	***1068.5796***	534.7935	1051.5531	526.2802	1050.5691	525.7882	12
18	**1808.9827**	904.9950	1791.9561	896.4817	1790.9721	895.9897	V	971.5269	486.2671	954.5003	477.7538	953.5163	477.2618	11
19	1922.0667	961.5370	1905.0402	953.0237	1904.0561	952.5317	I	***872.4585***	436.7329	855.4319	428.2196	854.4479	427.7276	10
20	1979.0882	990.0477	1962.0616	981.5345	1961.0776	981.0424	G	***759.3744***	380.1908	742.3478	371.6776	741.3638	371.1856	9
21	2050.1253	1025.5663	2033.0987	1017.0530	2032.1147	1016.5610	A	702.3529	351.6801	685.3264	343.1668	684.3424	342.6748	8
22	2107.1468	1054.0770	2090.1202	1045.5637	2089.1362	1045.0717	G	***631.3158***	316.1615	614.2893	307.6483	613.3052	307.1563	7
23	2164.1682	1082.5877	2147.1417	1074.0745	2146.1577	1073.5825	G	574.2944	287.6508	557.2678	279.1375	556.2838	278.6455	6
24	2277.2523	1139.1298	2260.2257	1130.6165	2259.2417	1130.1245	I	517.2729	259.1401	500.2463	250.6268	499.2623	250.1348	5
25	2334.2737	**1167.6405**	2317.2472	1159.1272	2316.2632	1158.6352	G	404.1888	202.5980	387.1623	194.0848	386.1783	193.5928	4
26	2449.3007	**1225.1540**	2432.2741	1216.6407	2431.2901	1216.1487	D	347.1674	174.0873	330.1408	165.5740	329.1568	165.0820	3
27	2506.3222	1253.6647	2489.2956	1245.1514	2488.3116	1244.6594	G	232.1404	116.5738	215.1139	108.0606			2
28							R	175.1190	88.0631	158.0924	79.5498			1

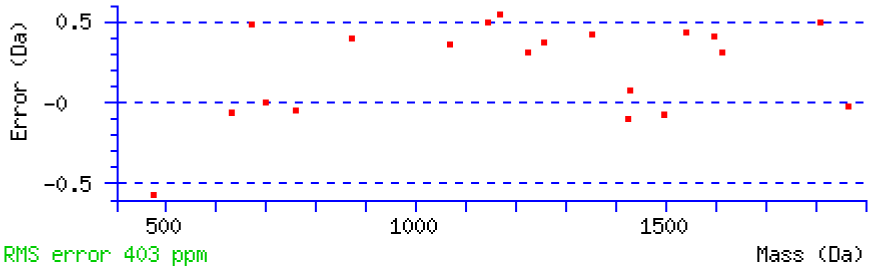

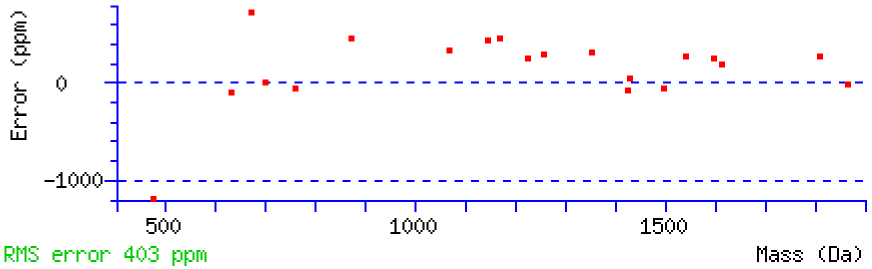

NCBI **BLAST** search of STTMALLPQVVDAVSVPVIGAGGIGDGR
(Parameters: blastp, nr protein database, expect=20000, no filter, PAM30)
Other BLAST web gateways

All matches to this query

Score	Mr(calc)	Delta	Sequence
54.7	2679.4266	-0.0052	STTMALLPQVVDAVSVPVIGAGGIGDGR
1.0	2679.4278	-0.0065	QRIIANETLEIYAPLAHRLGMNR

Mascot: http://www.matrixscience.com/

MATRIX SCIENCE MASCOT Search Results

Protein View: WP_005607387.1

zinc ABC transporter substrate-binding protein AdcA [Granulicatella adiacens]

**Database:**	UB_target
**Score:**	54
**Monoisotopic mass (M_r):**	57937
**Calculated pI:**	5.19

Sequence similarity is available as an NCBI BLAST search of WP_005607387.1 against nr.

Search parameters

**MS data file:**	LTQ_19B022_Kuweit_Sample-GA-EVS.mgf
**Enzyme:**	Trypsin: cuts C-term side of KR unless next residue is P.
**Fixed modifications:**	Carbamidomethyl (C)
**Variable modifications:**	Deamidated (NQ), Oxidation (M)

Protein sequence coverage: 4%

Matched peptides shown in ***bold red***.

```
  1 MKKTTFIGFL MLFLLVVAGC ATNNSNTSSS QTKQGKLKIS TTFYPIYDFT
 51 KNIVGDEADV SLVIGAGVEP HDYEPSAKEI AKMSEADALV YDSEYMETWI
101 PTVLKTLSDS KVKPISATKD MVLLPGGEEE EHDHDHSEEG HSHEYDPHVW
151 LSPERAIKMV QTITKQLVEA FPDRKEVFEK NANAYIEKLT ALHNDYTNAF
201 KDAKQKNFVT QHTAFRYLAL DYNLNQVGIT GISPEAEPSA SRLAELTKYV
251 KENDIKVIYF EENASEKIAK TLADETGVEL AVLNPIESLT KEQMDKGEDY
301 ISVMRENLAA LKKTTDQPGK DIQPEKTTDN KTVHNGYFED SAVKDRTLSD
351 YAGEWQSVYP YLVDGTLDQV FDYKAKLKKT MTKEEFKDYY TKGYKSDITN
401 INITDKTMEF KKEDGTTVKA EYKYVGYKIL TYKKGNRGVR FLFEAVTPVE
451 GAPKYVQFSD HNIAPVKAEH FHIFMGNESQ EKLLEEMDNW PTYYPTKLSG
501 LEIAQEMLAH
```

Unformatted sequence string: 510 residues (for pasting into other applications).

Sort by residue number increasing mass decreasing mass

Show matched peptides only predicted peptides also

Query	Start – End	Observed	Mr(expt)	Mr(calc)	ppm	M	Score	Expect	Rank	U	Peptide
16315	271 – 291	1107.0984	2212.1821	2212.1838	-0.76	0	42	0.00021	1	U	K.TLADETGVELAVLNPIESLTK.E
16316	271 – 291	1107.0987	2212.1828	2212.1838	-0.48	0	42	0.00024	1	U	K.TLADETGVELAVLNPIESLTK.E

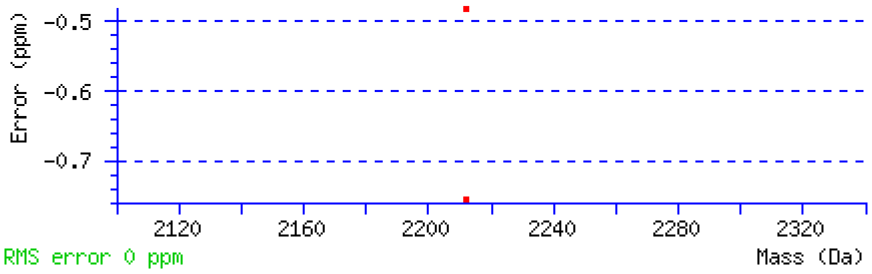

Mascot: http://www.matrixscience.com/

Mascot Search Results

Peptide View

MS/MS Fragmentation of **TLADETGVELAVLNPIESLTK**
Found in **WP_005607387.1** in **UB_target**, zinc ABC transporter substrate-binding protein AdcA [Granulicatella adiacens]

Match to Query 16315: 2212.182150 from(1107.098351,2+) index(21163)
Title: Elution from: 111.122 to 111.122 period: 0 experiment: 1 cycles: 1 precIntensity: 254357.0 FinneganScanNumber: 26349 MStype: enumIsNormalMS rawFile: 19B022_Kuweit_Sample-GA-EVS.raw
Data file LTQ_19B022_Kuweit_Sample-GA-EVS.mgf

Click mouse within plot area to zoom in by factor of two about that point

Or, Plot from 100 to 2000 Da Full range

Show Y-axis ☐

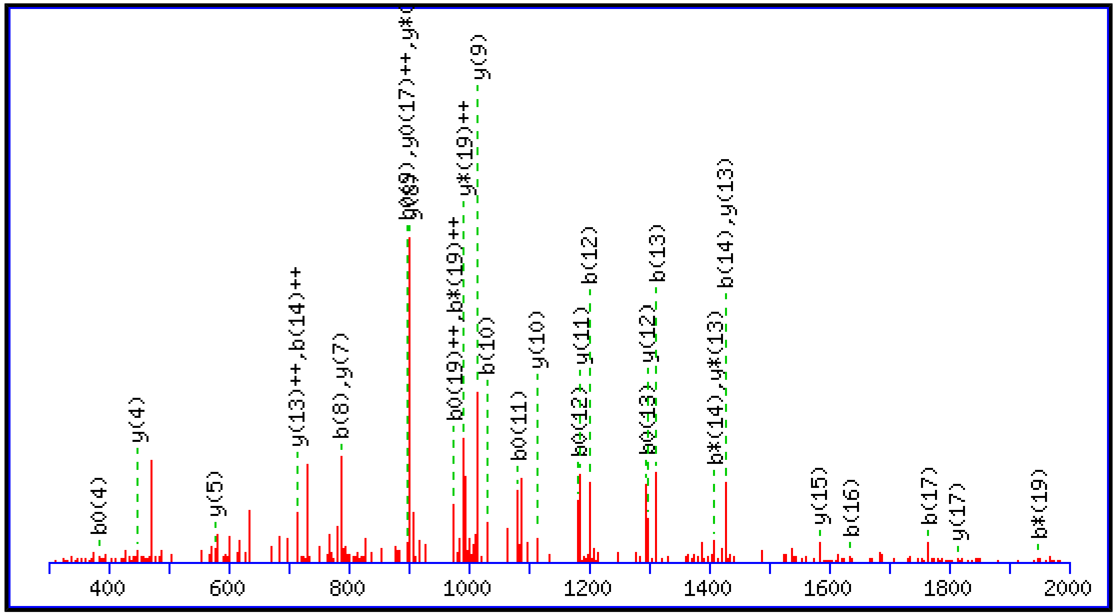

Label all possible matches Label matches used for scoring

Monoisotopic mass of neutral peptide Mr(calc): 2212.1838
Fixed modifications: Carbamidomethyl (C) (apply to specified residues or termini only)
Ions Score: 42 **Expect:** 0.00021

Mascot Search Results: Peptide View

Matches : 33/212 fragment ions using 68 most intense peaks (help)

#	b	b^{++}	b*	b^{*++}	b^0	b^{0++}	Seq.	y	y^{++}	y*	y^{*++}	y^0	y^{0++}	#
1	102.0550	51.5311			84.0444	42.5258	T							21
2	215.1390	108.0731			197.1285	99.0679	L	2112.1434	1056.5754	2095.1169	1048.0621	2094.1329	1047.5701	20
3	286.1761	143.5917			268.1656	134.5864	A	1999.0594	1000.0333	1982.0328	991.5201	1981.0488	991.0280	19
4	401.2031	201.1052			383.1925	192.0999	D	1928.0223	964.5148	1910.9957	956.0015	1910.0117	955.5095	18
5	530.2457	265.6265			512.2351	256.6212	E	1812.9953	907.0013	1795.9688	898.4880	1794.9848	897.9960	17
6	631.2933	316.1503			613.2828	307.1450	T	1683.9527	842.4800	1666.9262	833.9667	1665.9422	833.4747	16
7	688.3148	344.6610			670.3042	335.6558	G	1582.9050	791.9562	1565.8785	783.4429	1564.8945	782.9509	15
8	787.3832	394.1953			769.3727	385.1900	V	1525.8836	763.4454	1508.8570	754.9322	1507.8730	754.4401	14
9	916.4258	458.7165			898.4153	449.7113	E	1426.8152	713.9112	1409.7886	705.3979	1408.8046	704.9059	13
10	1029.5099	515.2586			1011.4993	506.2533	L	1297.7726	649.3899	1280.7460	640.8767	1279.7620	640.3846	12
11	1100.5470	550.7771			1082.5364	541.7719	A	1184.6885	592.8479	1167.6620	584.3346	1166.6779	583.8426	11
12	1199.6154	600.3113			1181.6048	591.3061	V	1113.6514	557.3293	1096.6249	548.8161	1095.6408	548.3241	10
13	1312.6995	656.8534			1294.6889	647.8481	L	1014.5830	507.7951	997.5564	499.2819	996.5724	498.7898	9
14	1426.7424	713.8748	1409.7159	705.3616	1408.7318	704.8696	N	901.4989	451.2531	884.4724	442.7398	883.4884	442.2478	8
15	1523.7952	762.4012	1506.7686	753.8879	1505.7846	753.3959	P	787.4560	394.2316	770.4294	385.7184	769.4454	385.2264	7
16	1636.8792	818.9433	1619.8527	810.4300	1618.8687	809.9380	I	690.4032	345.7053	673.3767	337.1920	672.3927	336.7000	6
17	1765.9218	883.4645	1748.8953	874.9513	1747.9113	874.4593	E	577.3192	289.1632	560.2926	280.6499	559.3086	280.1579	5
18	1852.9539	926.9806	1835.9273	918.4673	1834.9433	917.9753	S	448.2766	224.6419	431.2500	216.1287	430.2660	215.6366	4
19	1966.0379	983.5226	1949.0114	975.0093	1948.0273	974.5173	L	361.2445	181.1259	344.2180	172.6126	343.2340	172.1206	3
20	2067.0856	1034.0464	2050.0590	1025.5332	2049.0750	1025.0412	T	248.1605	124.5839	231.1339	116.0706	230.1499	115.5786	2
21							K	147.1128	74.0600	130.0863	65.5468			1

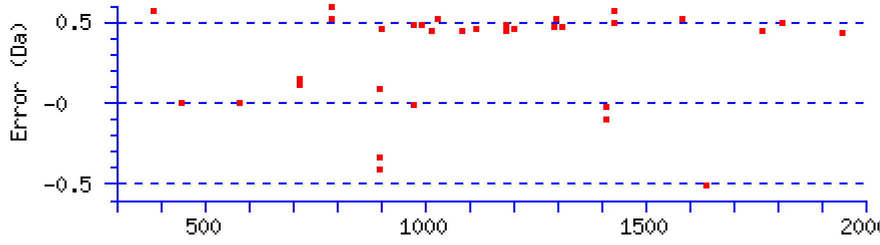

RMS error 447 ppm

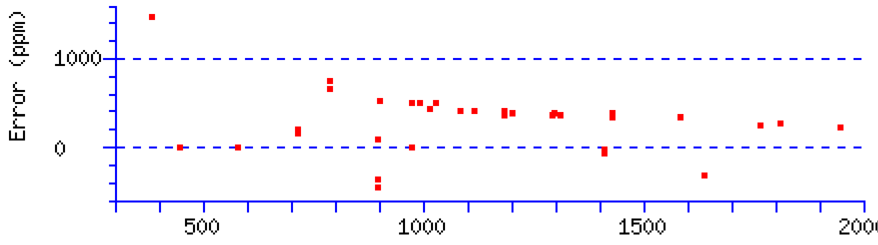

RMS error 447 ppm

NCBI **BLAST** search of TLADETGVELAVLNPIESLTK
(Parameters: blastp, nr protein database, expect=20000, no filter, PAM30)
Other BLAST web gateways

All matches to this query

Score	Mr(calc)	Delta	Sequence
42.1	2212.1838	-0.0017	TLADETGVELAVLNPIESLTK
2.6	2212.1826	-0.0005	TVYQHQKAMKPWIQPKTK

Mascot: http://www.matrixscience.com/

MASCOT Search Results

Protein View: RKW25673.1

hypothetical protein [Granulicatella sp.]

**Database:**	UB_target
**Score:**	54
**Monoisotopic mass (M_r):**	9792
**Calculated pI:**	7.77

Sequence similarity is available as an NCBI BLAST search of RKW25673.1 against nr.

Search parameters

**MS data file:**	LTQ_19B022_Kuweit_Sample-GA-EVS.mgf
**Enzyme:**	Trypsin: cuts C-term side of KR unless next residue is P.
**Fixed modifications:**	Carbamidomethyl (C)
**Variable modifications:**	Deamidated (NQ), Oxidation (M)

Protein sequence coverage: 15%

Matched peptides shown in ***bold red***.

1 MKNIEINVKE IVDYIEMNCY NRDTIGLHHP SMHQDLILNN R**LTEIDYING**
51 **AVVR**KGKKYG VPTPYCAFLT SLIHCKEQIL KAH

Unformatted sequence string: 83 residues (for pasting into other applications).

Sort by residue number increasing mass decreasing mass
Show matched peptides only predicted peptides also

Query	Start – End	Observed	Mr(expt)	Mr(calc)	ppm	M	Score	Expect	Rank	U	Peptide
8974	42 – 54	731.8974	1461.7802	1461.7827	-1.70	0	45	0.00017	1	U	R.LTEIDYINGAVVR.K
8975	42 – 54	731.8993	1461.7840	1461.7827	0.90	0	41	0.00048	1	U	R.LTEIDYINGAVVR.K

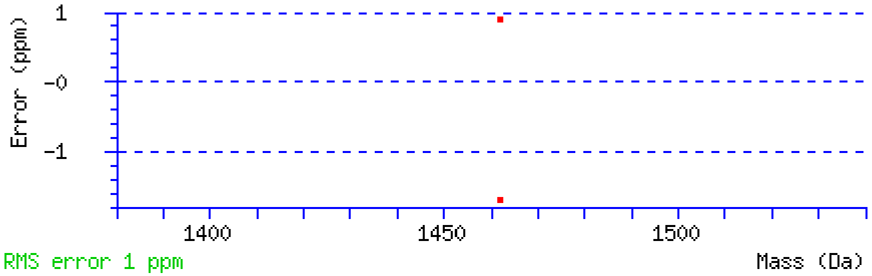

Mascot: http://www.matrixscience.com/

Mascot Search Results

Peptide View

MS/MS Fragmentation of **LTEIDYINGAVVR**
Found in **RKW25673.1** in **UB_target**, hypothetical protein [Granulicatella sp.]

Match to Query 8974: 1461.780250 from(731.897401,2+) index(15831)
Title: Elution from: 91.628 to 91.628 period: 0 experiment: 1 cycles: 1 precIntensity: 262104.0 FinneganScanNumber: 20187 MStype: enumIsNormalMS rawFile: 19B022_Kuweit_Sample-GA-EVS.raw
Data file LTQ_19B022_Kuweit_Sample-GA-EVS.mgf

Click mouse within plot area to zoom in by factor of two about that point
Or, Plot from 100 to 1400 Da Full range
Show Y-axis

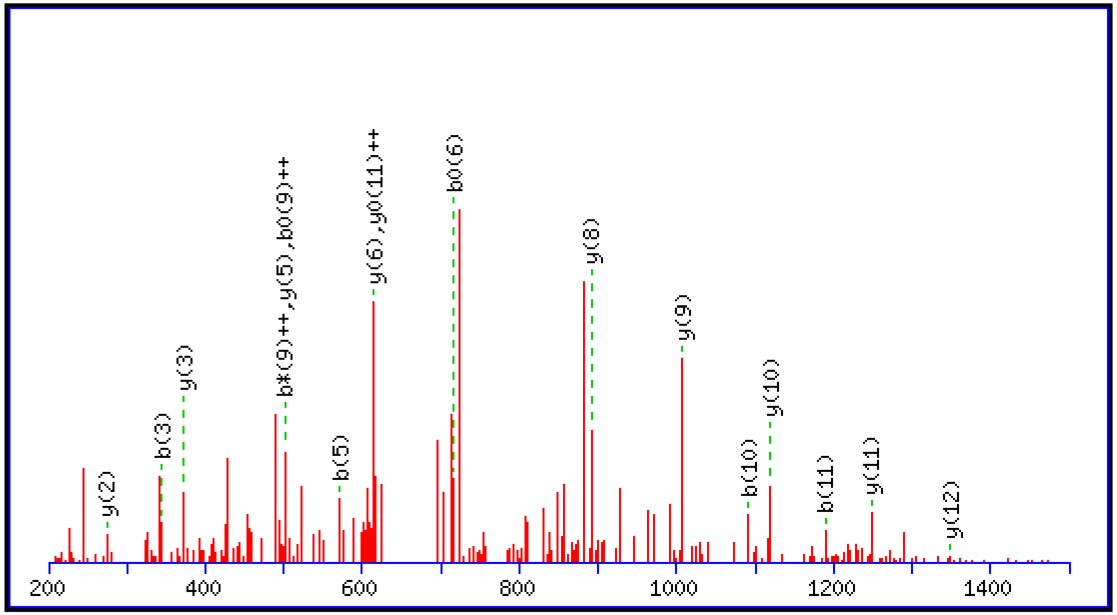

Label all possible matches Label matches used for scoring

Monoisotopic mass of neutral peptide Mr(calc): 1461.7827
Fixed modifications: Carbamidomethyl (C) (apply to specified residues or termini only)
Ions Score: 45 **Expect:** 0.00017

Matches : 17/112 fragment ions using 39 most intense peaks (help)

#	b	b^{++}	b*	b^{*++}	b^{0}	b^{0++}	Seq.	y	y^{++}	y*	y^{*++}	y^{0}	y^{0++}	#
1	114.0913	57.5493					L							13
2	215.1390	108.0731			197.1285	99.0679	T	***1349.7060***	675.3566	1332.6794	666.8433	1331.6954	666.3513	12
3	**344.1816**	172.5944			326.1710	163.5892	E	***1248.6583***	624.8328	1231.6317	616.3195	1230.6477	615.8275	11
4	457.2657	229.1365			439.2551	220.1312	I	***1119.6157***	560.3115	1102.5891	551.7982	1101.6051	551.3062	10
5	**572.2926**	286.6499			554.2821	277.6447	D	***1006.5316***	503.7694	989.5051	495.2562	988.5211	494.7642	9
6	735.3559	368.1816			717.3454	359.1763	Y	***891.5047***	446.2560	874.4781	437.7427			8
7	848.4400	424.7236			830.4294	415.7184	I	728.4413	364.7243	711.4148	356.2110			7
8	962.4829	481.7451	945.4564	473.2318	944.4724	472.7398	N	***615.3573***	308.1823	598.3307	299.6690			6
9	1019.5044	510.2558	1002.4779	501.7426	1001.4938	501.2506	G	***501.3144***	251.1608	484.2878	242.6475			5
10	**1090.5415**	545.7744	1073.5150	537.2611	1072.5310	536.7691	A	444.2929	222.6501	427.2663	214.1368			4
11	**1189.6099**	595.3086	1172.5834	586.7953	1171.5994	586.3033	V	***373.2558***	187.1315	356.2292	178.6183			3
12	1288.6783	644.8428	1271.6518	636.3295	1270.6678	635.8375	V	***274.1874***	137.5973	257.1608	129.0840			2
13							R	175.1190	88.0631	158.0924	79.5498			1

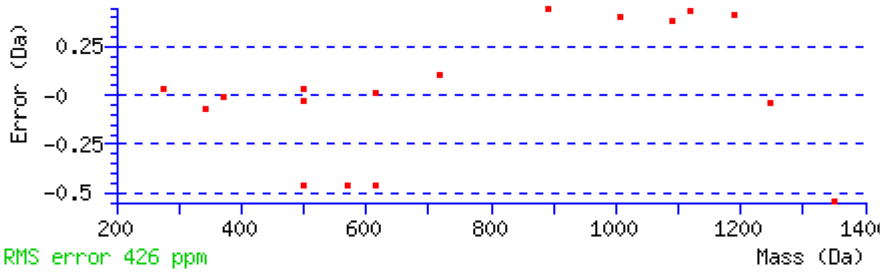

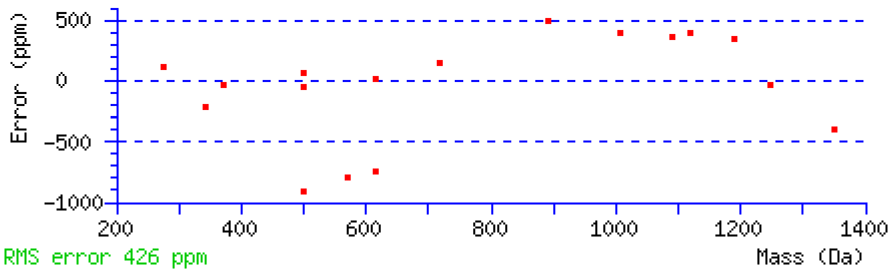

NCBI **BLAST** search of LTEIDYINGAVVR
(Parameters: blastp, nr protein database, expect=20000, no filter, PAM30)
Other BLAST web gateways

All matches to this query

Score	Mr(calc)	Delta	Sequence
44.6	1461.7827	-0.0025	LTEIDYINGAVVR
5.6	1461.7827	-0.0025	NRTSGYVLNLPVK

1.8	1461.7827	-0.0025	EVVGVYQGQKVVR

Mascot: http://www.matrixscience.com/

MATRIX SCIENCE MASCOT Search Results

Protein View: RKW28361.1

DUF1002 domain-containing protein [Granulicatella sp.]

**Database:**	UB_target
**Score:**	53
**Monoisotopic mass (M_r):**	34421
**Calculated pI:**	7.66

Sequence similarity is available as an NCBI BLAST search of RKW28361.1 against nr.

Search parameters

**MS data file:**	LTQ_19B022_Kuweit_Sample-GA-EVS.mgf
**Enzyme:**	Trypsin: cuts C-term side of KR unless next residue is P.
**Fixed modifications:**	Carbamidomethyl (C)
**Variable modifications:**	Deamidated (NQ), Oxidation (M)

Protein sequence coverage: 5%

Matched peptides shown in ***bold red***.

```
  1 MKKWVKGTAI VLALMGLSMK SAYAIDTTTI NEKWGKPTVV YGGGLNESQI
 51 KQTSELLGIK DPNTVKTEKA TGQDMIKYLG SGDGNTSVMI SSVMVQKKDK
101 GTGVKVRIKT PENITLVTAE QYANAAITAG VTDAEIEVAA VTKVTGESAL
151 TGVYKAFEAN GVQLDAKRTE VAQQELEVTN KIAQENANQK GFDSSKLDKA
201 MIEIKKELAE LKQKQGQLAT KEDIERIIND ALKNNSLQNV ISKDQINALV
251 AFAQNYQNTS AIDSKQVLEQ LNSLSKSVGE KINSLVEQAK NEGWLDKIAQ
301 FFTSIFESIK NLFNQQ
```

Unformatted sequence string: 316 residues (for pasting into other applications).

Sort by	residue number	increasing mass	decreasing mass
Show	matched peptides only	predicted peptides also	

Query	Start – End	Observed	Mr(expt)	Mr(calc)	ppm	M	Score	Expect	Rank	U	Peptide
13424	34 – 51	645.0107	1932.0103	1932.0105	-0.086	0	53	2e-005	1	U	K.WGKPTVVYGGGLNESQIK.Q

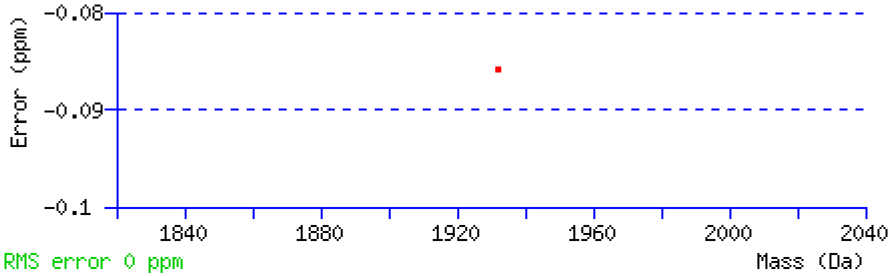

Mascot: http://www.matrixscience.com/

Mascot Search Results

Peptide View

MS/MS Fragmentation of **WGKPTVVYGGGLNESQIK**
Found in **RKW28361.1** in **UB_target**, DUF1002 domain-containing protein [Granulicatella sp.]

Match to Query 13424: 1932.010347 from(645.010725,3+) index(11102)
Title: Elution from: 72.983 to 72.983 period: 0 experiment: 1 cycles: 1 precIntensity: 255714.0 FinneganScanNumber: 14544 MStype: enumIsNormalMS rawFile: 19B022_Kuweit_Sample-GA-EVS.raw
Data file LTQ_19B022_Kuweit_Sample-GA-EVS.mgf

Click mouse within plot area to zoom in by factor of two about that point
Or, Plot from 150 to 1100 Da Full range
Show Y-axis

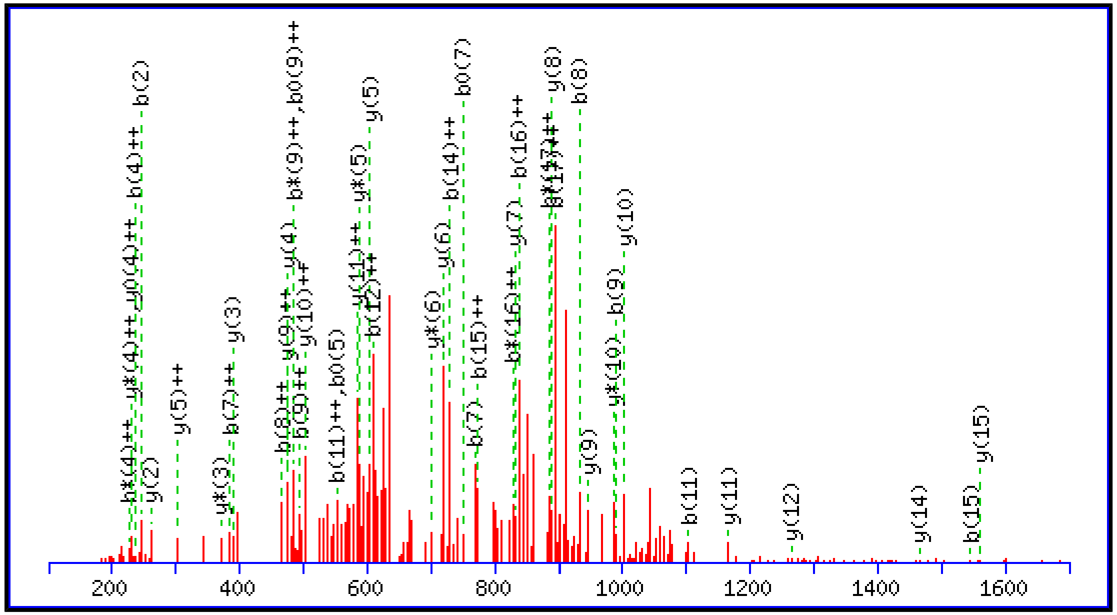

Label all possible matches Label matches used for scoring

Monoisotopic mass of neutral peptide Mr(calc): 1932.0105
Fixed modifications: Carbamidomethyl (C) (apply to specified residues or termini only)
Ions Score: 53 **Expect:** 2e-005

Matches : 46/186 fragment ions using 116 most intense peaks (help)

#	b	b^{++}	b*	b*$^{++}$	b^{0}	b^{0++}	Seq.	y	y^{++}	y*	y*$^{++}$	y^{0}	y^{0++}	#
1	187.0866	94.0469					W							18
2	244.1081	122.5577					G	1746.9385	873.9729	1729.9119	865.4596	1728.9279	864.9676	17
3	372.2030	186.6051	355.1765	178.0919			K	1689.9170	845.4621	1672.8905	836.9489	1671.9064	836.4569	16
4	469.2558	235.1315	452.2292	226.6183			P	1561.8221	781.4147	1544.7955	772.9014	1543.8115	772.4094	15
5	570.3035	285.6554	553.2769	277.1421	552.2929	276.6501	T	1464.7693	732.8883	1447.7427	724.3750	1446.7587	723.8830	14
6	669.3719	335.1896	652.3453	326.6763	651.3613	326.1843	V	1363.7216	682.3644	1346.6951	673.8512	1345.7110	673.3592	13
7	768.4403	384.7238	751.4137	376.2105	750.4297	375.7185	V	1264.6532	632.8302	1247.6266	624.3170	1246.6426	623.8250	12
8	931.5036	466.2554	914.4771	457.7422	913.4931	457.2502	Y	1165.5848	583.2960	1148.5582	574.7828	1147.5742	574.2907	11
9	988.5251	494.7662	971.4985	486.2529	970.5145	485.7609	G	1002.5215	501.7644	985.4949	493.2511	984.5109	492.7591	10
10	1045.5465	523.2769	1028.5200	514.7636	1027.5360	514.2716	G	945.5000	473.2536	928.4734	464.7404	927.4894	464.2483	9
11	1102.5680	551.7876	1085.5415	543.2744	1084.5574	542.7824	G	888.4785	444.7429	871.4520	436.2296	870.4680	435.7376	8
12	1215.6521	608.3297	1198.6255	599.8164	1197.6415	599.3244	L	831.4571	416.2322	814.4305	407.7189	813.4465	407.2269	7
13	1329.6950	665.3511	1312.6684	656.8379	1311.6844	656.3459	N	718.3730	359.6901	701.3464	351.1769	700.3624	350.6849	6
14	1458.7376	729.8724	1441.7110	721.3592	1440.7270	720.8672	E	604.3301	302.6687	587.3035	294.1554	586.3195	293.6634	5
15	1545.7696	773.3884	1528.7431	764.8752	1527.7591	764.3832	S	475.2875	238.1474	458.2609	229.6341	457.2769	229.1421	4
16	1673.8282	837.4177	1656.8016	828.9045	1655.8176	828.4125	Q	388.2554	194.6314	371.2289	186.1181			3
17	1786.9123	893.9598	1769.8857	885.4465	1768.9017	884.9545	I	260.1969	130.6021	243.1703	122.0888			2
18							K	147.1128	74.0600	130.0863	65.5468			1

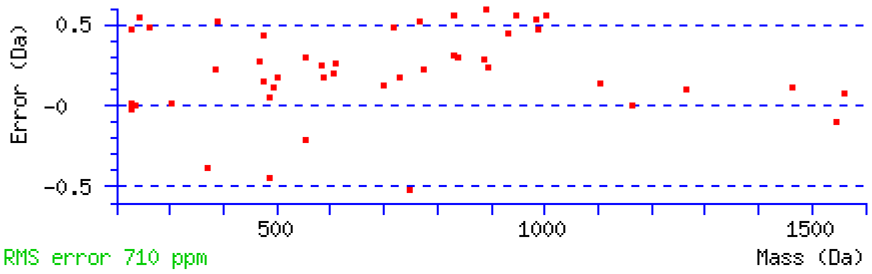

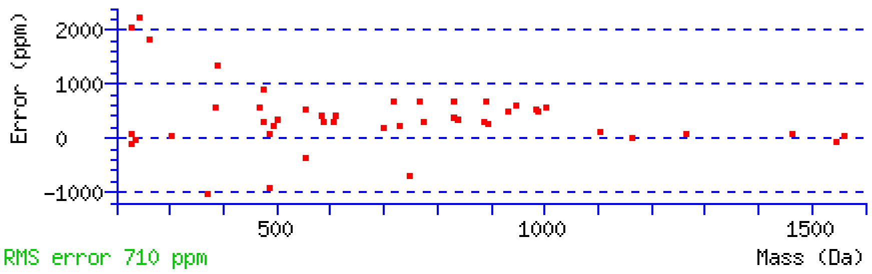

NCBI **BLAST** search of WGKPTVVYGGGLNESQIK
(Parameters: blastp, nr protein database, expect=20000, no filter, PAM30)
Other BLAST web gateways

All matches to this query

Score	Mr(calc)	Delta	Sequence
52.6	1932.0105	-0.0002	WGKPTVVYGGGLNESQIK
34.2	1932.0105	-0.0002	WGKPTVVYGGGLSDQQIK
11.6	1932.0138	-0.0035	AEAIAMAKQVWKDTNLK
7.4	1932.0139	-0.0035	NAICGFIGPNGAGKTTIIK
4.0	1932.0139	-0.0035	NAICGFIGPNGAGKTTIIK
2.2	1932.0065	0.0039	SRTNSSNVVPFQSPGVKK

Mascot: http://www.matrixscience.com/

MASCOT Search Results

Protein View: RKW27369.1

oxaloacetate decarboxylase subunit alpha, partial [Granulicatella sp.]

**Database:**	UB_target
**Score:**	48
**Monoisotopic mass (M_r):**	27936
**Calculated pI:**	5.30

Sequence similarity is available as an NCBI BLAST search of RKW27369.1 against nr.

Search parameters

**MS data file:**	LTQ_19B022_Kuweit_Sample-GA-EVS.mgf
**Enzyme:**	Trypsin: cuts C-term side of KR unless next residue is P.
**Fixed modifications:**	Carbamidomethyl (C)
**Variable modifications:**	Deamidated (NQ), Oxidation (M)

Protein sequence coverage: 3%

Matched peptides shown in ***bold red***.

```
  1 MTRKIGITDT ILRDAHQSLM ATRMRIEDML PVLDQLDEAG FASLECWGGA
 51 TFDACIRFLD EDPWDRLRTL KKHLKKTPLQ MLLRGQNILG YRHYADDVVE
101 KFVEKSAENG IDIFRIFDAL NDVRNLEASL NAVKKTGKEA QMTICYTISD
151 AHTIDYYKEL AAKMQEMGAD SICVKDMAGI LTPARGIELV RALKEVITVP
201 IVVHTHCTSG IAQLTYQAVI EAGADRIDTA LSPLSEGTSQ PPTESLVIA
```

Unformatted sequence string: 249 residues (for pasting into other applications).

Sort by	residue number	increasing mass	decreasing mass
Show	matched peptides only	predicted peptides also	

Query	Start – End	Observed	Mr(expt)	Mr(calc)	ppm	M	Score	Expect	Rank	U	Peptide
5322	116 – 124	531.7820	1061.5494	1061.5506	-1.09	0	30	0.0021	1	U	R.IFDALNDVR.N
5323	116 – 124	531.7828	1061.5511	1061.5506	0.47	0	46	9.5e-005	1	U	R.IFDALNDVR.N

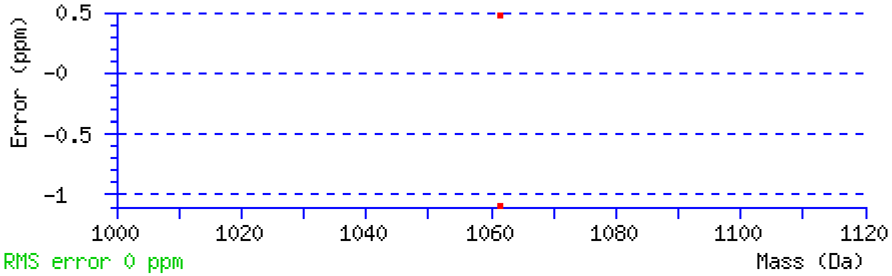

Mascot: http://www.matrixscience.com/

Mascot Search Results

Peptide View

MS/MS Fragmentation of **IFDALNDVR**
Found in **RKW27369.1** in **UB_target**, oxaloacetate decarboxylase subunit alpha, partial [Granulicatella sp.]

Match to Query 5323: 1061.551052 from(531.782802,2+) index(13987)
Title: Elution from: 84.260 to 84.260 period: 0 experiment: 1 cycles: 1 precIntensity: 274886.0 FinneganScanNumber: 17965 MStype: enumIsNormalMS rawFile: 19B022_Kuweit_Sample-GA-EVS.raw
Data file LTQ_19B022_Kuweit_Sample-GA-EVS.mgf

Click mouse within plot area to zoom in by factor of two about that point
Or, Plot from 100 to 1400 Da Full range
Show Y-axis

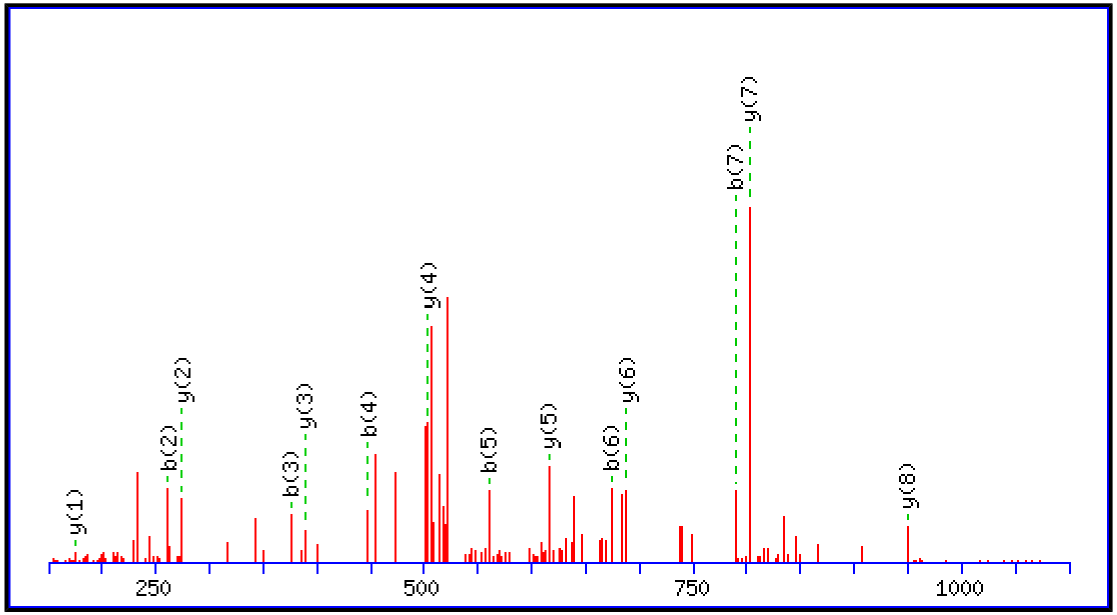

Label all possible matches Label matches used for scoring

Monoisotopic mass of neutral peptide Mr(calc): 1061.5506
Fixed modifications: Carbamidomethyl (C) (apply to specified residues or termini only)
Ions Score: 46 **Expect:** 9.5e-005

Matches : 14/78 fragment ions using 36 most intense peaks (help)

#	b	b^{++}	b*	b^{*++}	b^0	b^{0++}	Seq.	y	y^{++}	y*	y^{*++}	y^0	y^{0++}	#
1	114.0913	57.5493					I							9
2	**261.1598**	131.0835					F	***949.4738***	475.2405	932.4472	466.7272	931.4632	466.2352	8
3	**376.1867**	188.5970			358.1761	179.5917	D	***802.4054***	401.7063	785.3788	393.1930	784.3948	392.7010	7
4	**447.2238**	224.1155			429.2132	215.1103	A	***687.3784***	344.1928	670.3519	335.6796	669.3678	335.1876	6
5	**560.3079**	280.6576			542.2973	271.6523	L	***616.3413***	308.6743	599.3148	300.1610	598.3307	299.6690	5
6	**674.3508**	337.6790	657.3243	329.1658	656.3402	328.6738	N	***503.2572***	252.1323	486.2307	243.6190	485.2467	243.1270	4
7	**789.3777**	395.1925	772.3512	386.6792	771.3672	386.1872	D	***389.2143***	195.1108	372.1878	186.5975	371.2037	186.1055	3
8	888.4462	444.7267	871.4196	436.2134	870.4356	435.7214	V	***274.1874***	137.5973	257.1608	129.0840			2
9							R	***175.1190***	88.0631	158.0924	79.5498			1

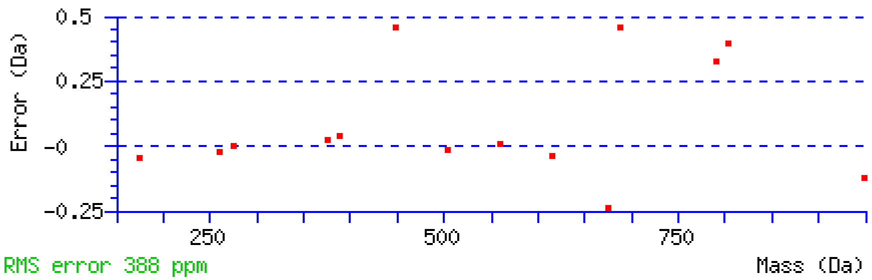

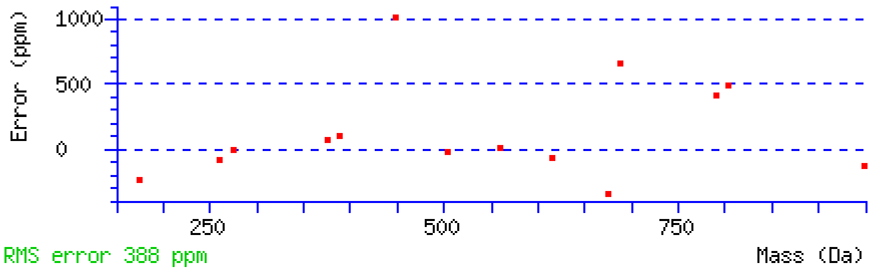

NCBI **BLAST** search of IFDALNDVR
(Parameters: blastp, nr protein database, expect=20000, no filter, PAM30)
Other BLAST web gateways

All matches to this query

Score	Mr(calc)	Delta	Sequence
46.3	1061.5506	0.0005	IFDALNDVR
0.4	1061.5539	-0.0028	VQMRAEIAK

Mascot: http://www.matrixscience.com/

MATRIX SCIENCE MASCOT Search Results

Protein View: WP_005607469.1

50S ribosomal protein L29 [Lactobacillales]

**Database:**	UB_target
**Score:**	40
**Monoisotopic mass (M_r):**	7960
**Calculated pI:**	9.69

Sequence similarity is available as an NCBI BLAST search of WP_005607469.1 against nr.

Search parameters

**MS data file:**	LTQ_19B022_Kuweit_Sample-GA-EVS.mgf
**Enzyme:**	Trypsin: cuts C-term side of KR unless next residue is P.
**Fixed modifications:**	Carbamidomethyl (C)
**Variable modifications:**	Deamidated (NQ), Oxidation (M)

Protein sequence coverage: 19%

Matched peptides shown in ***bold red***.

```
 1 MKVNELKKEL KGLSTAELVE KENELKQELF NLRFQLATGQ LEGTARIREV
51 RKQIARIKTA LRQEELQK
```

Unformatted sequence string: 68 residues (for pasting into other applications).

Sort by	residue number	increasing mass	decreasing mass
Show	matched peptides only	predicted peptides also	

Query	Start – End	Observed	Mr(expt)	Mr(calc)	ppm	M	Score	Expect	Rank	U	Peptide
8471	34 – 46	696.3673	1390.7201	1390.7205	-0.24	0	40	0.00038	1	U	R.FQLATGQLEGTAR.I

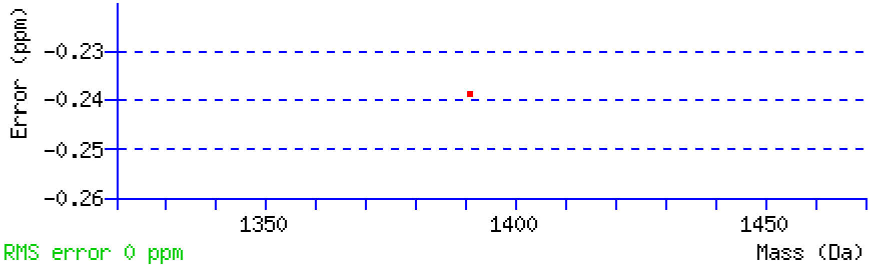

Mascot: http://www.matrixscience.com/

Mascot Search Results

Peptide View

MS/MS Fragmentation of **FQLATGQLEGTAR**
Found in **WP_005607469.1** in **UB_target**, 50S ribosomal protein L29 [Lactobacillales]

Match to Query 8471: 1390.720142 from(696.367347,2+) index(11465)
Title: Elution from: 74.348 to 74.348 period: 0 experiment: 1 cycles: 1 precIntensity: 372694.0 FinneganScanNumber: 14960 MStype: enumIsNormalMS rawFile: 19B022_Kuweit_Sample-GA-EVS.raw
Data file LTQ_19B022_Kuweit_Sample-GA-EVS.mgf

Click mouse within plot area to zoom in by factor of two about that point
Or, Plot from 150 to 1100 Da Full range
Show Y-axis ☐

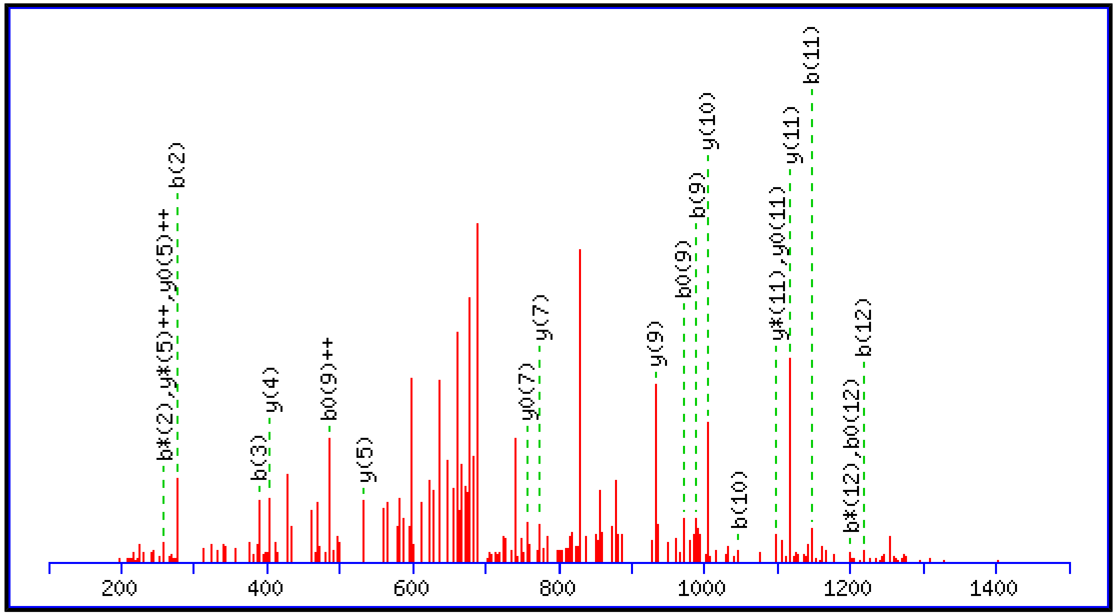

Label all possible matches Label matches used for scoring

Monoisotopic mass of neutral peptide Mr(calc): 1390.7205
Fixed modifications: Carbamidomethyl (C) (apply to specified residues or termini only)
Ions Score: 40 **Expect:** 0.00038

Matches : 22/130 fragment ions using 35 most intense peaks (help)

#	b	b^{++}	b*	b^{*++}	b^{0}	b^{0++}	Seq.	y	y^{++}	y*	y^{*++}	y^{0}	y^{0++}	#
1	148.0757	74.5415					F							13
2	***276.1343***	138.5708	259.1077	130.0575			Q	1244.6593	622.8333	1227.6328	614.3200	1226.6488	613.8280	12
3	***389.2183***	195.1128	372.1918	186.5995			L	***1116.6008***	558.8040	1099.5742	550.2907	1098.5902	549.7987	11
4	460.2554	230.6314	443.2289	222.1181			A	***1003.5167***	502.2620	986.4902	493.7487	985.5061	493.2567	10
5	561.3031	281.1552	544.2766	272.6419	543.2926	272.1499	T	***932.4796***	466.7434	915.4530	458.2302	914.4690	457.7381	9
6	618.3246	309.6659	601.2980	301.1527	600.3140	300.6607	G	831.4319	416.2196	814.4054	407.7063	813.4213	407.2143	8
7	746.3832	373.6952	729.3566	365.1819	728.3726	364.6899	Q	***774.4104***	387.7089	757.3839	379.1956	756.3999	378.7036	7
8	859.4672	430.2373	842.4407	421.7240	841.4567	421.2320	L	646.3519	323.6796	629.3253	315.1663	628.3413	314.6743	6
9	***988.5098***	494.7586	971.4833	486.2453	970.4993	485.7533	E	***533.2678***	267.1375	516.2413	258.6243	515.2572	258.1323	5
10	***1045.5313***	523.2693	1028.5047	514.7560	1027.5207	514.2640	G	***404.2252***	202.6162	387.1987	194.1030	386.2146	193.6110	4
11	***1146.5790***	573.7931	1129.5524	565.2798	1128.5684	564.7878	T	347.2037	174.1055	330.1772	165.5922	329.1932	165.1002	3
12	***1217.6161***	609.3117	1200.5895	600.7984	1199.6055	600.3064	A	246.1561	123.5817	229.1295	115.0684			2
13							R	175.1190	88.0631	158.0924	79.5498			1

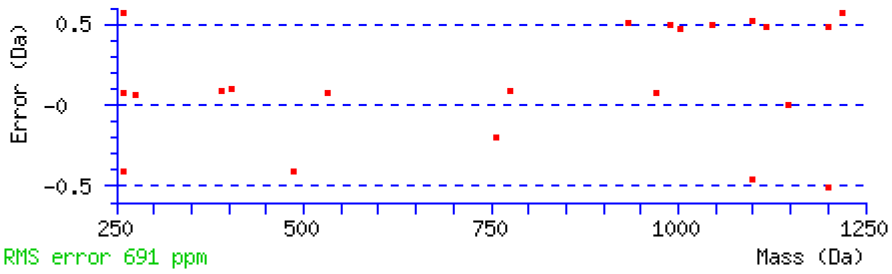

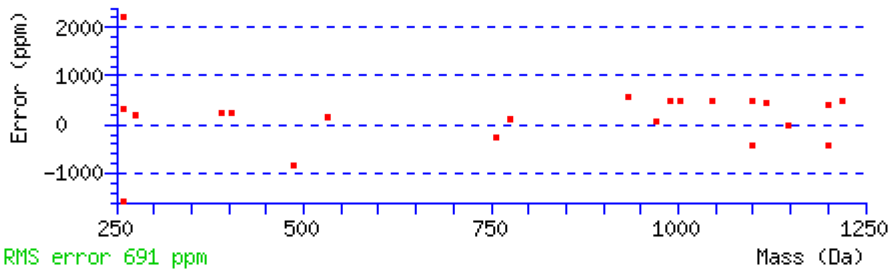

NCBI **BLAST** search of FQLATGQLEGTAR
(Parameters: blastp, nr protein database, expect=20000, no filter, PAM30)
Other BLAST web gateways

All matches to this query

Score	Mr(calc)	Delta	Sequence
40.3	1390.7205	-0.0003	FQLATGQLEGTAR
4.6	1390.7166	0.0035	IQQIPPMYSAVK

4.4	1390.7166	0.0035	KTYFLSMTGLSK
4.1	1390.7238	-0.0037	DQVEKRLTAGMK
3.9	1390.7166	0.0035	IQQIPPMYSAVK
1.7	1390.7238	-0.0037	CLNRLEKTTAGK

Mascot: http://www.matrixscience.com/

MASCOT Search Results

Protein View: EEW36998.1

ribosomal protein L2 [Granulicatella adiacens ATCC 49175 ATCC 49175]

**Database:**	UB_target
**Score:**	39
**Monoisotopic mass (M_r):**	28444
**Calculated pI:**	10.47

Sequence similarity is available as an NCBI BLAST search of EEW36998.1 against nr.

Search parameters

**MS data file:**	LTQ_19B022_Kuweit_Sample-GA-EVS.mgf
**Enzyme:**	Trypsin: cuts C-term side of KR unless next residue is P.
**Fixed modifications:**	Carbamidomethyl (C)
**Variable modifications:**	Deamidated (NQ), Oxidation (M)

Protein sequence coverage: 6%

Matched peptides shown in ***bold red***.

```
  1 MTGYDFSEIT KSTPEKTLLE SSKNNAGRNS QGKITVRHQG GGHKRAYRVI
 51 DFKRNKDNVE GVVHSIEYDP NRTANIALIH YVDGVKAYII APKGLQVGQR
101 IESGEHADIK VGNALPLANI PVGTVIHNIE TKPGKGGQLV RSAGTSAQVL
151 GQEGKYTLVR LNSGEVRMIL STCRATVGTV GNEQHELINF GKAGRSRWKR
201 KRPTVRGSVM NPNDHPHGGG EGRAPIGRPT PVSPWGKPAL GYKTRSKKAR
251 SNKLIVRGRK K
```

Unformatted sequence string: 261 residues (for pasting into other applications).

Sort by	residue number	increasing mass	decreasing mass
Show	matched peptides only	predicted peptides also	

Query	Start – End	Observed	Mr(expt)	Mr(calc)	ppm	M	Score	Expect	Rank	U	Peptide
13233	175 – 192	957.4895	1912.9645	1912.9643	0.12	0	39	0.00046	1	U	R.ATVGTVGNEQHELINFGK.A

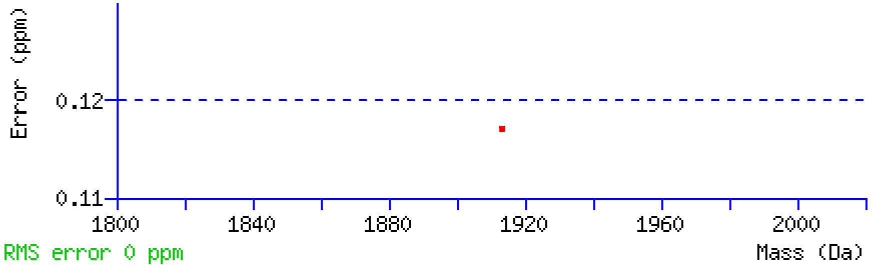

Mascot: http://www.matrixscience.com/

Mascot Search Results

Peptide View

MS/MS Fragmentation of **ATVGTVGNEQHELINFGK**
Found in **EEW36998.1** in **UB_target**, ribosomal protein L2 [Granulicatella adiacens ATCC 49175 ATCC 49175]

Match to Query 13233: 1912.964518 from(957.489535,2+) index(11977)
Title: Elution from: 76.498 to 76.498 period: 0 experiment: 1 cycles: 1 precIntensity: 204180.0 FinneganScanNumber: 15593 MStype: enumIsNormalMS rawFile: 19B022_Kuweit_Sample-GA-EVS.raw
Data file LTQ_19B022_Kuweit_Sample-GA-EVS.mgf

Click mouse within plot area to zoom in by factor of two about that point

Or, Plot from 150 to 1100 Da Full range

Show Y-axis

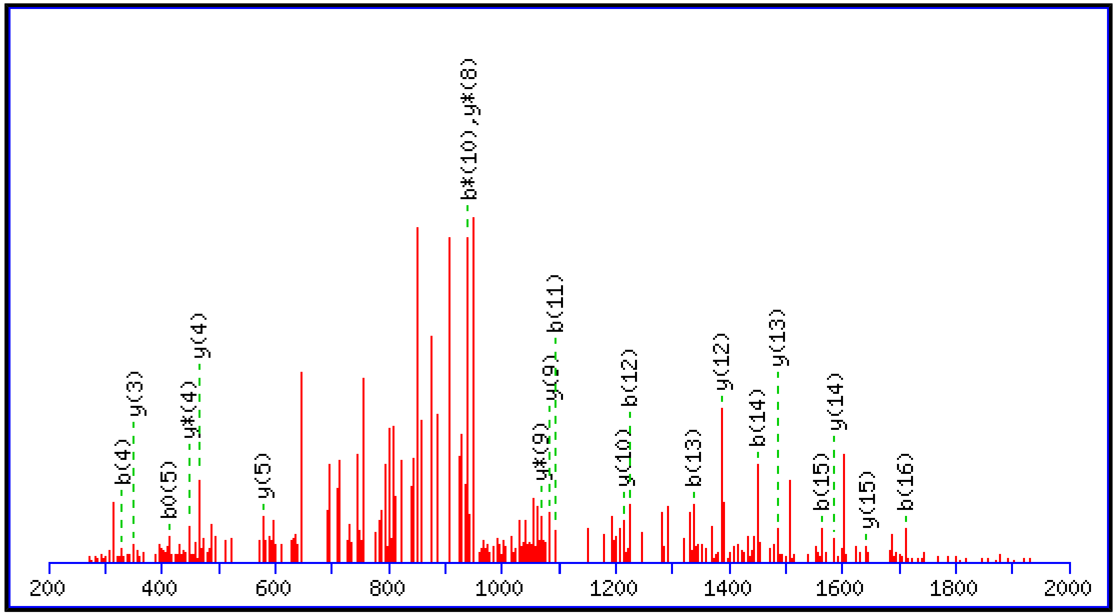

Label all possible matches Label matches used for scoring

Monoisotopic mass of neutral peptide Mr(calc): 1912.9643
Fixed modifications: Carbamidomethyl (C) (apply to specified residues or termini only)
Ions Score: 39 **Expect:** 0.00046

Matches : 21/176 fragment ions using 51 most intense peaks (help)

#	b	b^{++}	b^{*}	b^{*++}	b^{0}	b^{0++}	Seq.	y	y^{++}	y^{*}	y^{*++}	y^{0}	y^{0++}	#
1	72.0444	36.5258					A							18
2	173.0921	87.0497			155.0815	78.0444	T	1842.9345	921.9709	1825.9079	913.4576	1824.9239	912.9656	17
3	272.1605	136.5839			254.1499	127.5786	V	1741.8868	871.4470	1724.8602	862.9338	1723.8762	862.4417	16
4	***329.1819***	165.0946			311.1714	156.0893	G	***1642.8184***	821.9128	1625.7918	813.3995	1624.8078	812.9075	15
5	430.2296	215.6185			412.2191	206.6132	T	***1585.7969***	793.4021	1568.7703	784.8888	1567.7863	784.3968	14
6	529.2980	265.1527			511.2875	256.1474	V	***1484.7492***	742.8782	1467.7227	734.3650	1466.7387	733.8730	13
7	586.3195	293.6634			568.3089	284.6581	G	***1385.6808***	693.3440	1368.6543	684.8308	1367.6702	684.3388	12
8	700.3624	350.6849	683.3359	342.1716	682.3519	341.6796	N	1328.6593	664.8333	1311.6328	656.3200	1310.6488	655.8280	11
9	829.4050	415.2062	812.3785	406.6929	811.3945	406.2009	E	***1214.6164***	607.8118	1197.5899	599.2986	1196.6058	598.8066	10
10	957.4636	479.2354	940.4371	470.7222	939.4530	470.2302	Q	***1085.5738***	543.2905	1068.5473	534.7773	1067.5633	534.2853	9
11	***1094.5225***	547.7649	1077.4960	539.2516	1076.5119	538.7596	H	957.5152	479.2613	940.4887	470.7480	939.5047	470.2560	8
12	***1223.5651***	612.2862	1206.5386	603.7729	1205.5545	603.2809	E	820.4563	410.7318	803.4298	402.2185	802.4458	401.7265	7
13	***1336.6492***	668.8282	1319.6226	660.3149	1318.6386	659.8229	L	691.4137	346.2105	674.3872	337.6972			6
14	***1449.7332***	725.3703	1432.7067	716.8570	1431.7227	716.3650	I	***578.3297***	289.6685	561.3031	281.1552			5
15	***1563.7762***	782.3917	1546.7496	773.8784	1545.7656	773.3864	N	***465.2456***	233.1264	448.2191	224.6132			4
16	***1710.8446***	855.9259	1693.8180	847.4127	1692.8340	846.9206	F	***351.2027***	176.1050	334.1761	167.5917			3
17	1767.8660	884.4367	1750.8395	875.9234	1749.8555	875.4314	G	204.1343	102.5708	187.1077	94.0575			2
18							K	147.1128	74.0600	130.0863	65.5468			1

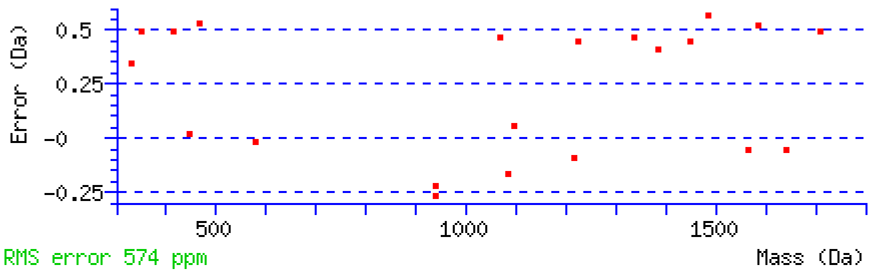

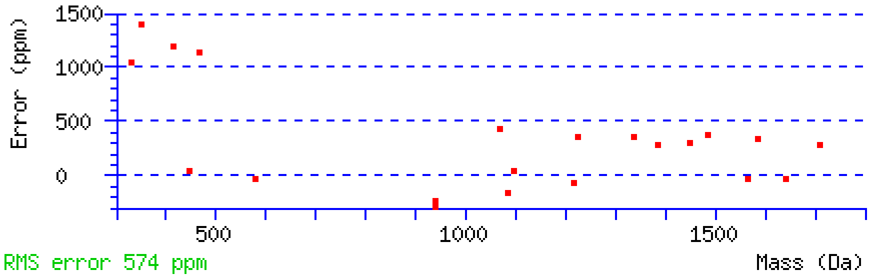

NCBI **BLAST** search of ATVGTVGNEQHELINFGK
(Parameters: blastp, nr protein database, expect=20000, no filter, PAM30)
Other BLAST web gateways

All matches to this query

Score	Mr(calc)	Delta	Sequence
39.0	1912.9643	0.0002	ATVGTVGNEQHELINFGK

Mascot: http://www.matrixscience.com/

MASCOT Search Results

Protein View: RKW29296.1

FMN-binding protein [Granulicatella sp.]

**Database:**	UB_target
**Score:**	36
**Monoisotopic mass (M_r):**	19355
**Calculated pI:**	8.97

Sequence similarity is available as an NCBI BLAST search of RKW29296.1 against nr.

Search parameters

**MS data file:**	LTQ_19B022_Kuweit_Sample-GA-EVS.mgf
**Enzyme:**	Trypsin: cuts C-term side of KR unless next residue is P.
**Fixed modifications:**	Carbamidomethyl (C)
**Variable modifications:**	Deamidated (NQ), Oxidation (M)

Protein sequence coverage: 11%

Matched peptides shown in ***bold red***.

```
  1 MVKTMTIKKS LSATAVLLSS VFVLAACGGN SKTDSSKTTT QAATTQTATT
 51 QAAAQKSDAA LKDGTYKLVS AADKRGWHVE FTITVEGGKI TKSDYDNLND
101 KGERKSADAA YEKSMKDKVG TGPAEYFKAY NEGLVSKQNP KDVEVVAGAT
151 NAHTSFVEYA NKLIEAAKKG DTTEIKVEAP KN
```

Unformatted sequence string: 182 residues (for pasting into other applications).

Sort by	residue number	increasing mass	decreasing mass
Show	matched peptides only	predicted peptides also	

Query	Start – End	Observed	Mr(expt)	Mr(calc)	ppm	M	Score	Expect	Rank	U	Peptide
16379	142 – 162	1111.5376	2221.0606	2221.0651	-2.03	0	36	0.0012	1	U	K.DVEVVAGATNAHTSFVEYANK.L

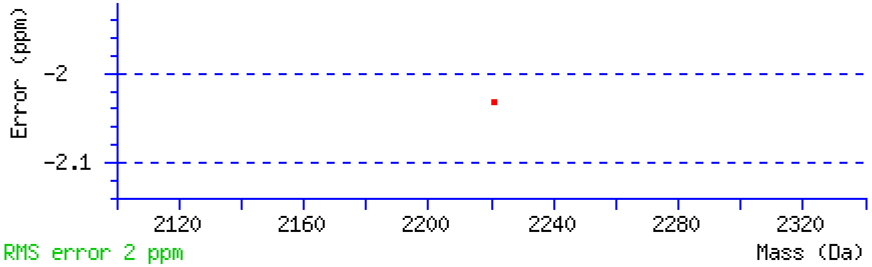

Mascot: http://www.matrixscience.com/

MATRIX SCIENCE Mascot Search Results

Peptide View

MS/MS Fragmentation of **DVEVVAGATNAHTSFVEYANK**
Found in **RKW29296.1** in **UB_target**, FMN-binding protein [Granulicatella sp.]

Match to Query 16379: 2221.060610 from(1111.537581,2+) index(12358)
Title: Elution from: 77.970 to 77.970 period: 0 experiment: 1 cycles: 1 precIntensity: 163212.0 FinneganScanNumber: 16041 MStype: enumIsNormalMS rawFile: 19B022_Kuweit_Sample-GA-EVS.raw
Data file LTQ_19B022_Kuweit_Sample-GA-EVS.mgf

Click mouse within plot area to zoom in by factor of two about that point
Or, Plot from 150 to 1100 Da Full range
Show Y-axis

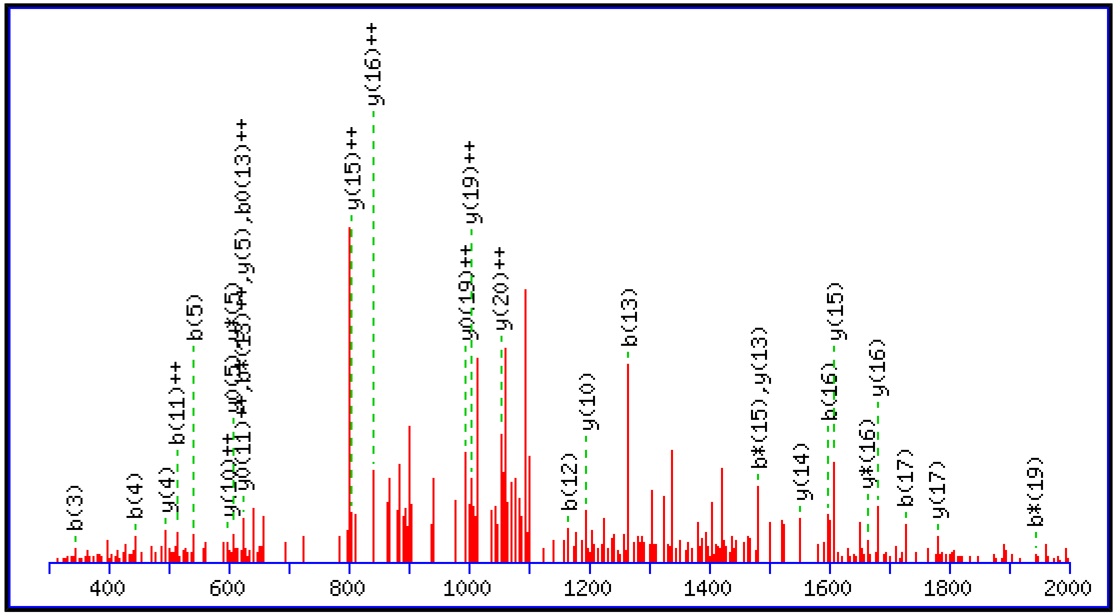

Label all possible matches Label matches used for scoring

Monoisotopic mass of neutral peptide Mr(calc): 2221.0651
Fixed modifications: Carbamidomethyl (C) (apply to specified residues or termini only)
Ions Score: 36 **Expect:** 0.0012

Matches : 31/214 fragment ions using 51 most intense peaks (help)

#	b	b^{++}	b*	b^{*++}	b^0	b^{0++}	Seq.	y	y^{++}	y*	y^{*++}	y^0	y^{0++}	#
1	116.0342	58.5207			98.0237	49.5155	D							21
2	215.1026	108.0550			197.0921	99.0497	V	2107.0455	1054.0264	2090.0189	1045.5131	2089.0349	1045.0211	20
3	344.1452	172.5763			326.1347	163.5710	E	2007.9770	1004.4922	1990.9505	995.9789	1989.9665	995.4869	19
4	443.2136	222.1105			425.2031	213.1052	V	1878.9345	939.9709	1861.9079	931.4576	1860.9239	930.9656	18
5	542.2821	271.6447			524.2715	262.6394	V	1779.8660	890.4367	1762.8395	881.9234	1761.8555	881.4314	17
6	613.3192	307.1632			595.3086	298.1579	A	1680.7976	840.9025	1663.7711	832.3892	1662.7871	831.8972	16
7	670.3406	335.6740			652.3301	326.6687	G	1609.7605	805.3839	1592.7340	796.8706	1591.7499	796.3786	15
8	741.3777	371.1925			723.3672	362.1872	A	1552.7390	776.8732	1535.7125	768.3599	1534.7285	767.8679	14
9	842.4254	421.7164			824.4149	412.7111	T	1481.7019	741.3546	1464.6754	732.8413	1463.6914	732.3493	13
10	956.4684	478.7378	939.4418	470.2245	938.4578	469.7325	N	1380.6543	690.8308	1363.6277	682.3175	1362.6437	681.8255	12
11	1027.5055	514.2564	1010.4789	505.7431	1009.4949	505.2511	A	1266.6113	633.8093	1249.5848	625.2960	1248.6008	624.8040	11
12	1164.5644	582.7858	1147.5378	574.2726	1146.5538	573.7805	H	1195.5742	598.2907	1178.5477	589.7775	1177.5636	589.2855	10
13	1265.6121	633.3097	1248.5855	624.7964	1247.6015	624.3044	T	1058.5153	529.7613	1041.4888	521.2480	1040.5047	520.7560	9
14	1352.6441	676.8257	1335.6175	668.3124	1334.6335	667.8204	S	957.4676	479.2374	940.4411	470.7242	939.4571	470.2322	8
15	1499.7125	750.3599	1482.6860	741.8466	1481.7019	741.3546	F	870.4356	435.7214	853.4090	427.2082	852.4250	426.7162	7
16	1598.7809	799.8941	1581.7544	791.3808	1580.7703	790.8888	V	723.3672	362.1872	706.3406	353.6740	705.3566	353.1819	6
17	1727.8235	864.4154	1710.7970	855.9021	1709.8129	855.4101	E	624.2988	312.6530	607.2722	304.1397	606.2882	303.6477	5
18	1890.8868	945.9471	1873.8603	937.4338	1872.8763	936.9418	Y	495.2562	248.1317	478.2296	239.6185			4
19	1961.9239	981.4656	1944.8974	972.9523	1943.9134	972.4603	A	332.1928	166.6001	315.1663	158.0868			3
20	2075.9669	1038.4871	2058.9403	1029.9738	2057.9563	1029.4818	N	261.1557	131.0815	244.1292	122.5682			2
21							K	147.1128	74.0600	130.0863	65.5468			1

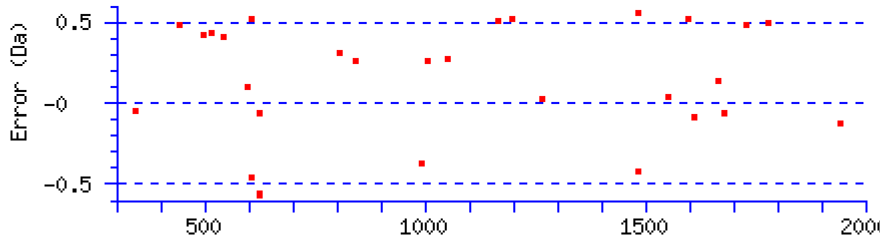

RMS error 499 ppm

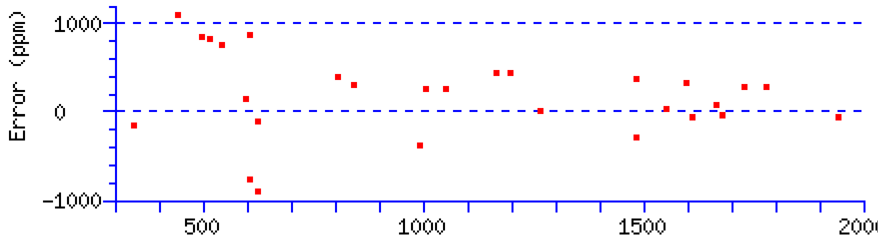

RMS error 499 ppm

NCBI **BLAST** search of DVEVVAGATNAHTSFVEYANK
(Parameters: blastp, nr protein database, expect=20000, no filter, PAM30)
Other BLAST web gateways

All matches to this query

Score	Mr(calc)	Delta	Sequence
35.5	2221.0651	-0.0045	DVEVVAGATNAHTSFVEYANK

Mascot: http://www.matrixscience.com/

MATRIX SCIENCE MASCOT Search Results

Protein View: RKW25792.1

acetate kinase, partial [Granulicatella sp.]

**Database:**	UB_target
**Score:**	35
**Monoisotopic mass (M_r):**	42369
**Calculated pI:**	5.04

Sequence similarity is available as an NCBI BLAST search of RKW25792.1 against nr.

Search parameters

**MS data file:**	LTQ_19B022_Kuweit_Sample-GA-EVS.mgf
**Enzyme:**	Trypsin: cuts C-term side of KR unless next residue is P.
**Fixed modifications:**	Carbamidomethyl (C)
**Variable modifications:**	Deamidated (NQ), Oxidation (M)

Protein sequence coverage: 3%

Matched peptides shown in ***bold red***.

```
  1 MSKSIAINAG SSSLKFQLFN MPQEEVVAKG LVERIGLENS IFSISYGDDQ
 51 KYEVVEDIPN HEVAVEKLLE QLVALNIIAS FDEITGVGHR VVAGGEIFKD
101 SALVDDTVIQ QVEDLAEFAP LHNKAEAIGM RAFKHILPDI TSVAVFDTSF
151 HTTMPKKAYL YSVPLEYYKQ FKARKYGAHG TSHRYVSHRA AELLGKPIEE
201 LKIITCHLGN GASITAVDGG KSVDTSMGFT PLAGVTMGTR SGDIDASLVA
251 FLMNKLNITD INEMVDILNK KSGLLGLSGL SSDMRDIDNA SATNEDAKVA
301 MEIFVDRVQK YIGQYIAVMN GVDAIVFTAG IGENSIKIRD LIISGITWFG
351 CDIDKERNNT RSEAIISSDN AKVTVLNIPT NEEVEIAR
```

Unformatted sequence string: 388 residues (for pasting into other applications).

Sort by	residue number	increasing mass	decreasing mass
Show	matched peptides only	predicted peptides also	

Query	Start – End	Observed	Mr(expt)	Mr(calc)	ppm	M	Score	Expect	Rank	U	Peptide
10889	16 – 29	840.4261	1678.8376	1678.8389	-0.74	0	35	0.0016	1	U	K.FQLFNMPQEEVVAK.G

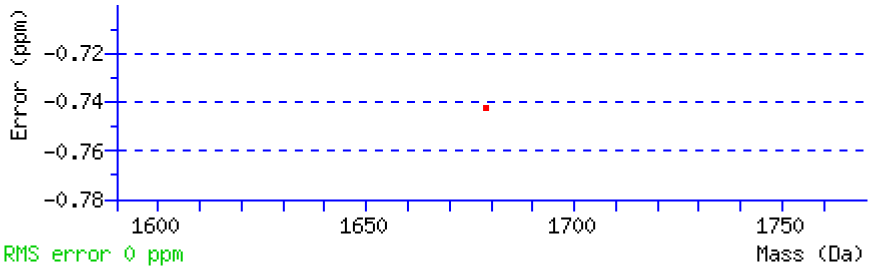

Mascot: http://www.matrixscience.com/

Mascot Search Results

Peptide View

MS/MS Fragmentation of **FQLFNMPQEEVVAK**
Found in **RKW25792.1** in **UB_target**, acetate kinase, partial [Granulicatella sp.]

Match to Query 10889: 1678.837620 from(840.426086,2+) index(19027)
Title: Elution from: 103.380 to 103.380 period: 0 experiment: 1 cycles: 1 precIntensity: 157541.0 FinneganScanNumber: 23912 MStype: enumIsNormalMS rawFile: 19B022_Kuweit_Sample-GA-EVS.raw
Data file LTQ_19B022_Kuweit_Sample-GA-EVS.mgf

Click mouse within plot area to zoom in by factor of two about that point
Or, Plot from 150 to 1100 Da Full range
Show Y-axis

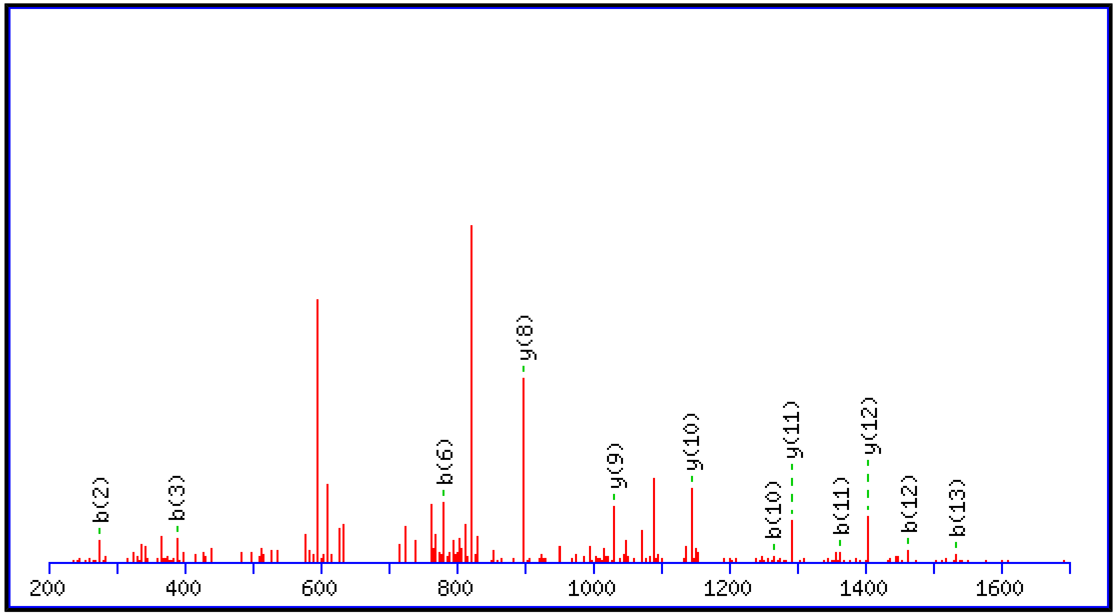

Label all possible matches Label matches used for scoring

Monoisotopic mass of neutral peptide Mr(calc): 1678.8389
Fixed modifications: Carbamidomethyl (C) (apply to specified residues or termini only)
Ions Score: 35 **Expect:** 0.0016

Matches : 12/130 fragment ions using 28 most intense peaks (help)

#	b	b^{++}	b*	b^{*++}	b^0	b^{0++}	Seq.	y	y^{++}	y*	y^{*++}	y^0	y^{0++}	#
1	148.0757	74.5415					F							14
2	***276.1343***	138.5708	259.1077	130.0575			Q	1532.7777	766.8925	1515.7512	758.3792	1514.7672	757.8872	13
3	***389.2183***	195.1128	372.1918	186.5995			L	***1404.7192***	702.8632	1387.6926	694.3499	1386.7086	693.8579	12
4	536.2867	268.6470	519.2602	260.1337			F	***1291.6351***	646.3212	1274.6086	637.8079	1273.6245	637.3159	11
5	650.3297	325.6685	633.3031	317.1552			N	***1144.5667***	572.7870	1127.5401	564.2737	1126.5561	563.7817	10
6	***781.3702***	391.1887	764.3436	382.6754			M	***1030.5238***	515.7655	1013.4972	507.2522	1012.5132	506.7602	9
7	878.4229	439.7151	861.3964	431.2018			P	***899.4833***	450.2453	882.4567	441.7320	881.4727	441.2400	8
8	1006.4815	503.7444	989.4550	495.2311			Q	802.4305	401.7189	785.4040	393.2056	784.4199	392.7136	7
9	1135.5241	568.2657	1118.4975	559.7524	1117.5135	559.2604	E	674.3719	337.6896	657.3454	329.1763	656.3614	328.6843	6
10	***1264.5667***	632.7870	1247.5401	624.2737	1246.5561	623.7817	E	545.3293	273.1683	528.3028	264.6550	527.3188	264.1630	5
11	***1363.6351***	682.3212	1346.6086	673.8079	1345.6245	673.3159	V	416.2867	208.6470	399.2602	200.1337			4
12	***1462.7035***	731.8554	1445.6770	723.3421	1444.6929	722.8501	V	317.2183	159.1128	300.1918	150.5995			3
13	***1533.7406***	767.3740	1516.7141	758.8607	1515.7301	758.3687	A	218.1499	109.5786	201.1234	101.0653			2
14							K	147.1128	74.0600	130.0863	65.5468			1

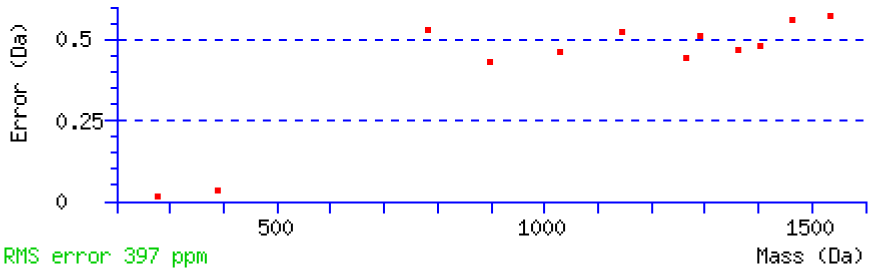

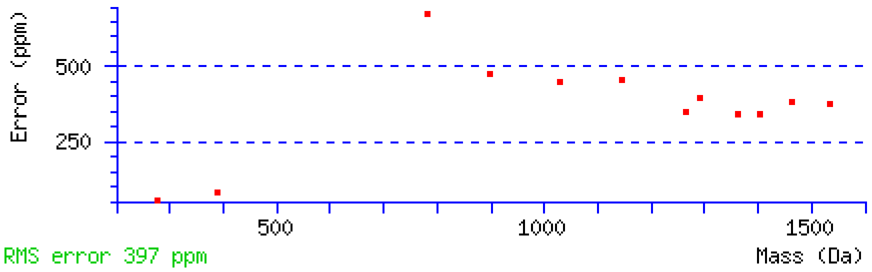

NCBI **BLAST** search of FQLFNMPQEEVVAK
(Parameters: blastp, nr protein database, expect=20000, no filter, PAM30)
Other BLAST web gateways

All matches to this query

Score	Mr(calc)	Delta	Sequence
34.8	1678.8389	-0.0012	FQLFNMPQEEVVAK

0.8	1678.8413	-0.0037	QFETEKALAKEQEK

**Mascot:** http://www.matrixscience.com/

MASCOT Search Results

Protein View: WP_005607235.1

Lrp/AsnC family transcriptional regulator [Granulicatella adiacens]

**Database:**	UB_target
**Score:**	34
**Monoisotopic mass (M_r):**	18963
**Calculated pI:**	6.13

Sequence similarity is available as an NCBI BLAST search of WP_005607235.1 against nr.

Search parameters

**MS data file:**	LTQ_19B022_Kuweit_Sample-GA-EVS.mgf
**Enzyme:**	Trypsin: cuts C-term side of KR unless next residue is P.
**Fixed modifications:**	Carbamidomethyl (C)
**Variable modifications:**	Deamidated (NQ), Oxidation (M)

Protein sequence coverage: 11%

Matched peptides shown in ***bold red***.

```
  1 MFKEVNEQIL KLIEKNSRLT PEEIASLLEM DVEEVTRRIK EMEEAKVICG
 51 YHTLINWEKT DNVNVSAIIE LKVNPQKGKG FDRIAEKIYH FPEVEAVYLM
101 SGGYDFMVQL KKAPMREIAN FVSSRLSVIE EVQSTKTHVV LKQYKDHGTM
151 FVGKADDKRQ VVTP
```

Unformatted sequence string: 164 residues (for pasting into other applications).

Sort by	residue number	increasing mass	decreasing mass
Show	matched peptides only	predicted peptides also	

Query	Start – End	Observed	Mr(expt)	Mr(calc)	ppm	M	Score	Expect	Rank	U	Peptide
15948	19 – 37	1087.5480	2173.0815	2173.0824	-0.40	0	34	0.0015	1	U	R.LTPEEIASLLEMDVEEVTR.R

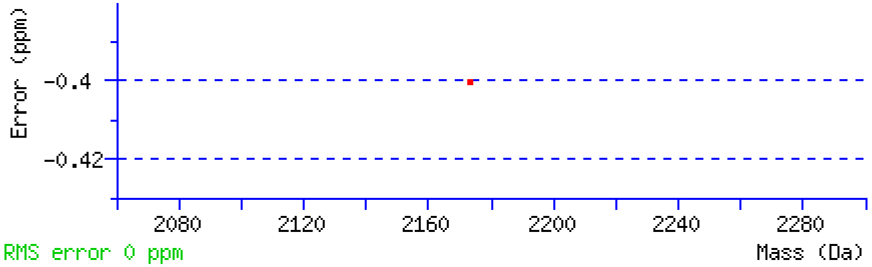

Mascot: http://www.matrixscience.com/

Mascot Search Results

Peptide View

MS/MS Fragmentation of **LTPEEIASLLEMDVEEVTR**
Found in **WP_005607235.1** in **UB_target**, Lrp/AsnC family transcriptional regulator [Granulicatella adiacens]

Match to Query 15948: 2173.081512 from(1087.548032,2+) index(23179)
Title: Elution from: 119.182 to 119.182 period: 0 experiment: 1 cycles: 1 precIntensity: 347968.0 FinneganScanNumber: 28741 MStype: enumIsNormalMS rawFile: 19B022_Kuweit_Sample-GA-EVS.raw
Data file LTQ_19B022_Kuweit_Sample-GA-EVS.mgf

Click mouse within plot area to zoom in by factor of two about that point
Or, Plot from 100 to 2000 Da Full range
Show Y-axis

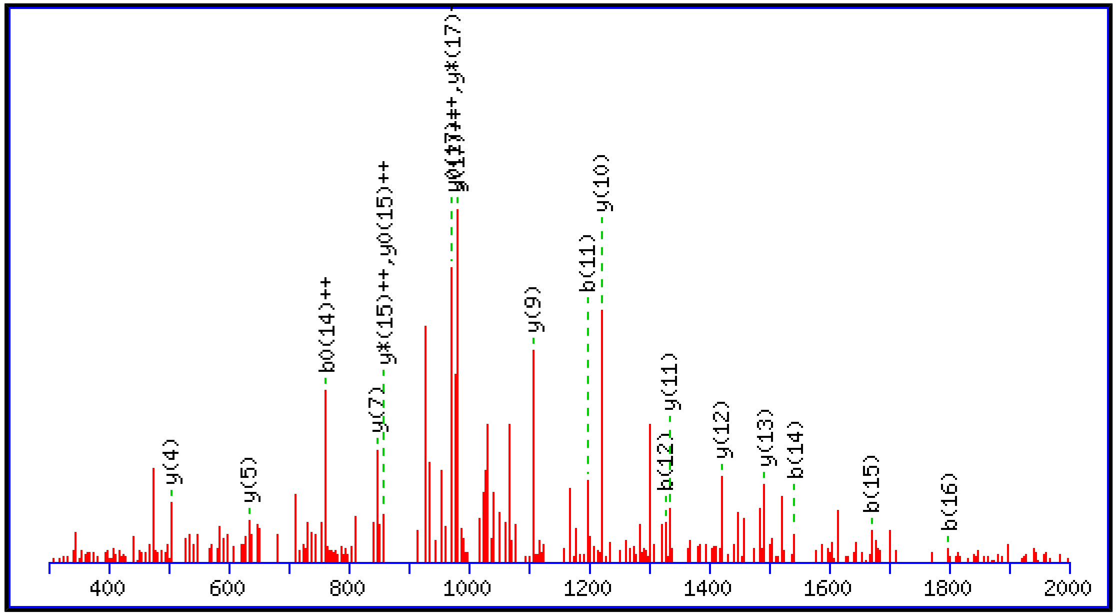

Label all possible matches Label matches used for scoring

```
Monoisotopic mass of neutral peptide Mr(calc): 2173.0824
Fixed modifications: Carbamidomethyl (C) (apply to specified residues or termini only)
Ions Score: 34  Expect: 0.0015
```

Matches : 19/176 fragment ions using 34 most intense peaks (help)

#	b	b++	b0	b0++	Seq.	y	y++	y*	y*++	y0	y0++	#
1	114.0913	57.5493			L							19
2	215.1390	108.0731	197.1285	99.0679	T	2061.0056	1031.0065	2043.9791	1022.4932	2042.9951	1022.0012	18
3	312.1918	156.5995	294.1812	147.5942	P	1959.9580	980.4826	1942.9314	971.9693	1941.9474	971.4773	17
4	441.2344	221.1208	423.2238	212.1155	E	1862.9052	931.9562	1845.8786	923.4430	1844.8946	922.9509	16
5	570.2770	285.6421	552.2664	276.6368	E	1733.8626	867.4349	1716.8360	858.9217	1715.8520	858.4297	15
6	683.3610	342.1842	665.3505	333.1789	I	1604.8200	802.9136	1587.7935	794.4004	1586.8094	793.9084	14
7	754.3981	377.7027	736.3876	368.6974	A	***1491.7359***	746.3716	1474.7094	737.8583	1473.7254	737.3663	13
8	841.4302	421.2187	823.4196	412.2134	S	***1420.6988***	710.8530	1403.6723	702.3398	1402.6883	701.8478	12
9	954.5142	477.7608	936.5037	468.7555	L	***1333.6668***	667.3370	1316.6402	658.8238	1315.6562	658.3318	11
10	1067.5983	534.3028	1049.5877	525.2975	L	***1220.5827***	610.7950	1203.5562	602.2817	1202.5722	601.7897	10
11	***1196.6409***	598.8241	1178.6303	589.8188	E	***1107.4987***	554.2530	1090.4721	545.7397	1089.4881	545.2477	9
12	***1327.6814***	664.3443	1309.6708	655.3390	M	978.4561	489.7317	961.4295	481.2184	960.4455	480.7264	8
13	1442.7083	721.8578	1424.6978	712.8525	D	***847.4156***	424.2114	830.3890	415.6982	829.4050	415.2061	7
14	***1541.7767***	771.3920	1523.7662	762.3867	V	732.3886	366.6980	715.3621	358.1847	714.3781	357.6927	6
15	***1670.8193***	835.9133	1652.8088	826.9080	E	***633.3202***	317.1638	616.2937	308.6505	615.3097	308.1585	5
16	***1799.8619***	900.4346	1781.8514	891.4293	E	***504.2776***	252.6425	487.2511	244.1292	486.2671	243.6372	4
17	1898.9303	949.9688	1880.9198	940.9635	V	375.2350	188.1212	358.2085	179.6079	357.2245	179.1159	3
18	1999.9780	1000.4926	1981.9675	991.4874	T	276.1666	138.5870	259.1401	130.0737	258.1561	129.5817	2
19					R	175.1190	88.0631	158.0924	79.5498			1

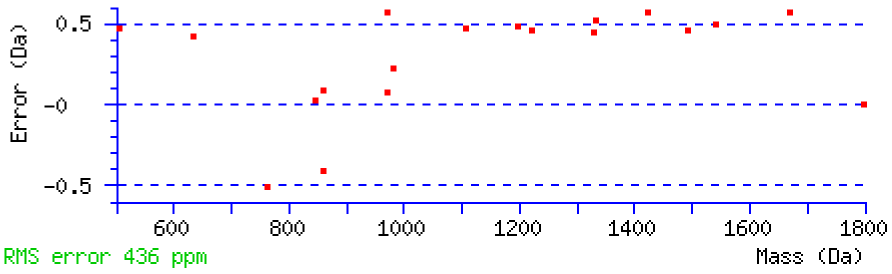

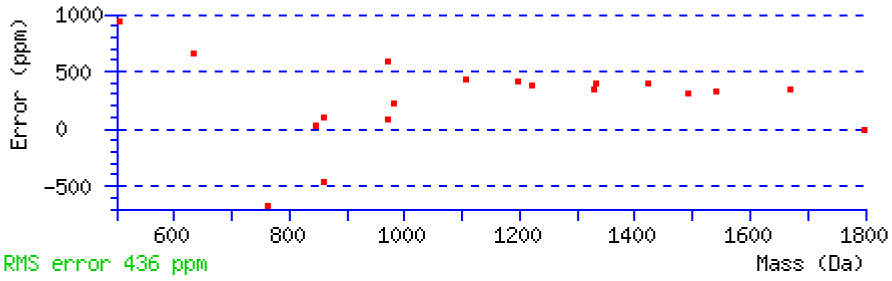

NCBI **BLAST** search of LTPEEIASLLEMDVEEVTR
(Parameters: blastp, nr protein database, expect=20000, no filter, PAM30)

Other BLAST web gateways

All matches to this query

Score	Mr(calc)	Delta	Sequence
34.4	2173.0824	-0.0009	LTPEEIASLLEMDVEEVTR

Mascot: http://www.matrixscience.com/

MATRIX SCIENCE MASCOT Search Results

Protein View: EEW36408.1

hypothetical protein [Granulicatella adiacens ATCC 49175 ATCC 49175]

**Database:**	UB_target
**Score:**	34
**Monoisotopic mass (M_r):**	10436
**Calculated pI:**	6.73

Sequence similarity is available as an NCBI BLAST search of EEW36408.1 against nr.

Search parameters

**MS data file:**	LTQ_19B022_Kuweit_Sample-GA-EVS.mgf
**Enzyme:**	Trypsin: cuts C-term side of KR unless next residue is P.
**Fixed modifications:**	Carbamidomethyl (C)
**Variable modifications:**	Deamidated (NQ), Oxidation (M)

Protein sequence coverage: 18%

Matched peptides shown in ***bold red***.

```
 1 MEVIIMIKEL VSLGKFAKKS GVFAGGVLFG SLGLKLLASK EAKHVYAKAV
51 ATSYKLKDGI DATVSTVKQH ADDVLEEAKD LYAEEKNAQL VVETSEK
```

Unformatted sequence string: 97 residues (for pasting into other applications).

Sort by	residue number	increasing mass	decreasing mass
Show	matched peptides only	predicted peptides also	

Query	Start – End	Observed	Mr(expt)	Mr(calc)	ppm	M	Score	Expect	Rank	U	Peptide
15229	69 – 86	701.6657	2101.9752	2101.9803	-2.45	1	34	0.001	1	U	K.QHADDVLEEAKDLYAEEK.N

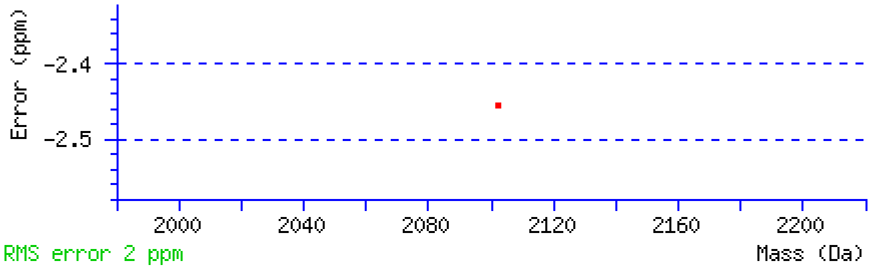

Mascot: http://www.matrixscience.com/

MATRIX SCIENCE Mascot Search Results

Peptide View

MS/MS Fragmentation of **QHADDVLEEAKDLYAEEK**
Found in **EEW36408.1** in **UB_target**, hypothetical protein [Granulicatella adiacens ATCC 49175 ATCC 49175]

Match to Query 15229: 2101.975188 from(701.665672,3+) index(16849)
Title: Elution from: 95.565 to 95.565 period: 0 experiment: 1 cycles: 1 precIntensity: 321062.0 FinneganScanNumber: 21413 MStype: enumIsNormalMS
rawFile: 19B022_Kuweit_Sample-GA-EVS.raw
Data file LTQ_19B022_Kuweit_Sample-GA-EVS.mgf

Click mouse within plot area to zoom in by factor of two about that point

Or, Plot from 150 to 1100 Da Full range

Show Y-axis ☐

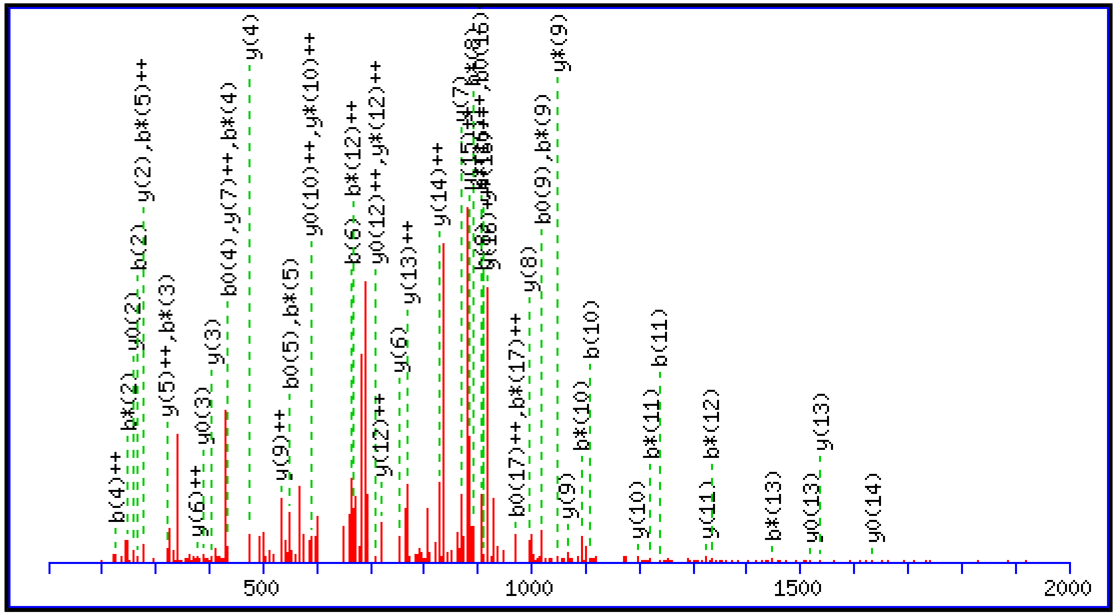

Label all possible matches Label matches used for scoring

Monoisotopic mass of neutral peptide Mr(calc): 2101.9803
Fixed modifications: Carbamidomethyl (C) (apply to specified residues or termini only)
Ions Score: 34 **Expect:** 0.001

Matches : 54/196 fragment ions using 136 most intense peaks (help)

#	b	b^{++}	b*	b^{*++}	b^{0}	b^{0++}	Seq.	y	y^{++}	y*	y^{*++}	y^{0}	y^{0++}	#
1	129.0659	65.0366	112.0393	56.5233			Q							18
2	266.1248	133.5660	249.0982	125.0527			H	1974.9291	987.9682	1957.9025	979.4549	1956.9185	978.9629	17
3	337.1619	169.0846	320.1353	160.5713			A	1837.8702	919.4387	1820.8436	910.9255	1819.8596	910.4334	16
4	452.1888	226.5980	435.1623	218.0848	434.1783	217.5928	D	1766.8331	883.9202	1749.8065	875.4069	1748.8225	874.9149	15
5	567.2158	284.1115	550.1892	275.5982	549.2052	275.1062	D	1651.8061	826.4067	1634.7796	817.8934	1633.7956	817.4014	14
6	666.2842	333.6457	649.2576	325.1325	648.2736	324.6404	V	1536.7792	768.8932	1519.7526	760.3800	1518.7686	759.8879	13
7	779.3682	390.1878	762.3417	381.6745	761.3577	381.1825	L	1437.7108	719.3590	1420.6842	710.8457	1419.7002	710.3537	12
8	908.4108	454.7091	891.3843	446.1958	890.4003	445.7038	E	1324.6267	662.8170	1307.6002	654.3037	1306.6161	653.8117	11
9	1037.4534	519.2304	1020.4269	510.7171	1019.4429	510.2251	E	1195.5841	598.2957	1178.5576	589.7824	1177.5735	589.2904	10
10	1108.4905	554.7489	1091.4640	546.2356	1090.4800	545.7436	A	1066.5415	533.7744	1049.5150	525.2611	1048.5310	524.7691	9
11	1236.5855	618.7964	1219.5590	610.2831	1218.5749	609.7911	K	995.5044	498.2558	978.4779	489.7426	977.4938	489.2506	8
12	1351.6125	676.3099	1334.5859	667.7966	1333.6019	667.3046	D	867.4094	434.2084	850.3829	425.6951	849.3989	425.2031	7
13	1464.6965	732.8519	1447.6700	724.3386	1446.6859	723.8466	L	752.3825	376.6949	735.3559	368.1816	734.3719	367.6896	6
14	1627.7598	814.3836	1610.7333	805.8703	1609.7493	805.3783	Y	639.2984	320.1529	622.2719	311.6396	621.2879	311.1476	5
15	1698.7970	849.9021	1681.7704	841.3888	1680.7864	840.8968	A	476.2351	238.6212	459.2086	230.1079	458.2245	229.6159	4
16	1827.8395	914.4234	1810.8130	905.9101	1809.8290	905.4181	E	405.1980	203.1026	388.1714	194.5894	387.1874	194.0974	3
17	1956.8821	978.9447	1939.8556	970.4314	1938.8716	969.9394	E	276.1554	138.5813	259.1288	130.0681	258.1448	129.5761	2
18							K	147.1128	74.0600	130.0863	65.5468			1

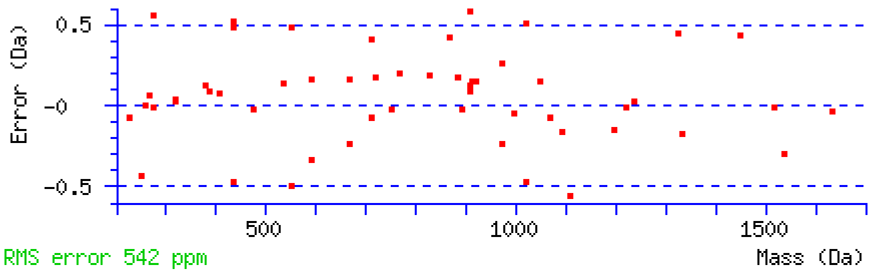

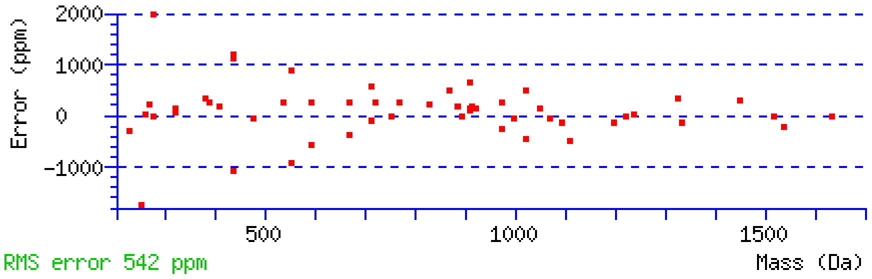

NCBI **BLAST** search of QHADDVLEEAKDLYAEEK
(Parameters: blastp, nr protein database, expect=20000, no filter, PAM30)
Other BLAST web gateways

All matches to this query

Score	Mr(calc)	Delta	Sequence
34.1	2101.9803	-0.0052	QHADDVLEEAKDLYAEEK

Mascot: http://www.matrixscience.com/

MASCOT Search Results

Protein View: RKW25990.1

methionine ABC transporter substrate-binding protein, partial [Granulicatella sp.]

**Database:**	UB_target
**Score:**	33
**Monoisotopic mass (M_r):**	27189
**Calculated pI:**	5.05

Sequence similarity is available as an NCBI BLAST search of RKW25990.1 against nr.

Search parameters

**MS data file:**	LTQ_19B022_Kuweit_Sample-GA-EVS.mgf
**Enzyme:**	Trypsin: cuts C-term side of KR unless next residue is P.
**Fixed modifications:**	Carbamidomethyl (C)
**Variable modifications:**	Deamidated (NQ), Oxidation (M)

Protein sequence coverage: 6%

Matched peptides shown in ***bold red***.

```
  1 MKIKHLFLTA IAAITLAACG SQTSKEEVKE STTEAAQTVK VAVVGSAAHE
 51 LWDYVAEKAK KENINIEVVE MNDYVLPNTA LEEGSVQMNA FQHRAYLAQW
101 NKDKGSDLKE IGTTFITPLY YFSTKYKSLK DLPEKAKVLV PKEVAIQGRA
151 LVALQTEGLI TLKEGVGTKA SLADITSNPR NLEIIEAESA QAPQMLQDVD
201 AASINGSMAQ DAGLKIEDNI FTDANHLDTI PKDRYNIIVV NGKDADNPV
```

Unformatted sequence string: 249 residues (for pasting into other applications).

Sort by	residue number	increasing mass	decreasing mass
Show	matched peptides only	predicted peptides also	

Query	Start – End	Observed	Mr(expt)	Mr(calc)	ppm	M	Score	Expect	Rank	U	Peptide
12865	110 – 125	940.9882	1879.9619	1879.9608	0.59	0	31	0.0026	1	U	K.EIGTTFITPLYYFSTK.Y
12866	110 – 125	940.9882	1879.9619	1879.9608	0.60	0	32	0.0025	1	U	K.EIGTTFITPLYYFSTK.Y

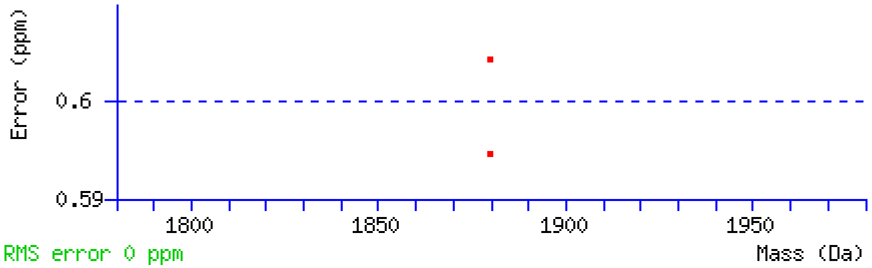

Mascot: http://www.matrixscience.com/

Mascot Search Results

Peptide View

MS/MS Fragmentation of **EIGTTFITPLYYFSTK**
Found in **RKW25990.1** in **UB_target**, methionine ABC transporter substrate-binding protein, partial [Granulicatella sp.]

Match to Query 12866: 1879.961906 from(940.988229,2+) index(21107)
Title: Elution from: 110.920 to 110.920 period: 0 experiment: 1 cycles: 1 precIntensity: 311080.0 FinneganScanNumber: 26286 MStype: enumIsNormalMS rawFile: 19B022_Kuweit_Sample-GA-EVS.raw
Data file LTQ_19B022_Kuweit_Sample-GA-EVS.mgf

Click mouse within plot area to zoom in by factor of two about that point

Or, Plot from 150 to 1100 Da Full range

Show Y-axis

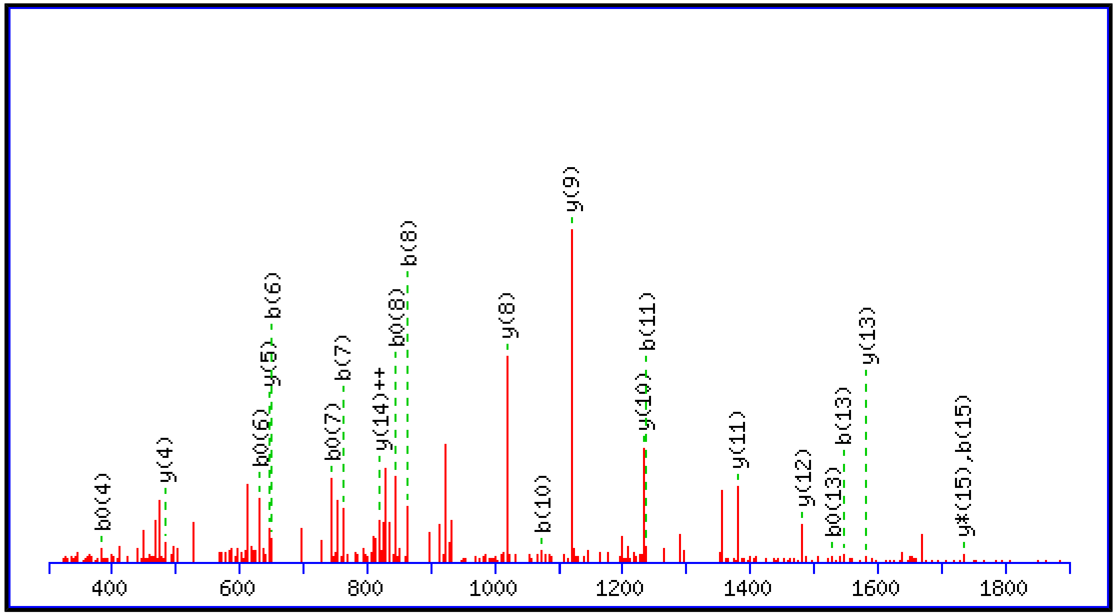

Label all possible matches Label matches used for scoring

Monoisotopic mass of neutral peptide Mr(calc): 1879.9608
Fixed modifications: Carbamidomethyl (C) (apply to specified residues or termini only)
Ions Score: 32 **Expect:** 0.0025

Matches : 22/148 fragment ions using 62 most intense peaks (help)

#	b	b^{++}	b^{0}	b^{0++}	Seq.	y	y^{++}	y^{*}	y^{*++}	y^{0}	y^{0++}	#
1	130.0499	65.5286	112.0393	56.5233	E							16
2	243.1339	122.0706	225.1234	113.0653	I	1751.9255	876.4664	1734.8989	867.9531	1733.9149	867.4611	15
3	300.1554	150.5813	282.1448	141.5761	G	1638.8414	819.9243	1621.8148	811.4111	1620.8308	810.9191	14
4	401.2031	201.1052	383.1925	192.0999	T	***1581.8199***	791.4136	1564.7934	782.9003	1563.8094	782.4083	13
5	502.2508	251.6290	484.2402	242.6237	T	***1480.7722***	740.8898	1463.7457	732.3765	1462.7617	731.8845	12
6	***649.3192***	325.1632	631.3086	316.1579	F	***1379.7246***	690.3659	1362.6980	681.8526	1361.7140	681.3606	11
7	***762.4032***	381.7053	744.3927	372.7000	I	***1232.6562***	616.8317	1215.6296	608.3184	1214.6456	607.8264	10
8	***863.4509***	432.2291	845.4403	423.2238	T	***1119.5721***	560.2897	1102.5455	551.7764	1101.5615	551.2844	9
9	960.5037	480.7555	942.4931	471.7502	P	***1018.5244***	509.7658	1001.4979	501.2526	1000.5138	500.7606	8
10	***1073.5877***	537.2975	1055.5772	528.2922	L	921.4716	461.2395	904.4451	452.7262	903.4611	452.2342	7
11	***1236.6511***	618.8292	1218.6405	609.8239	Y	808.3876	404.6974	791.3610	396.1842	790.3770	395.6921	6
12	1399.7144	700.3608	1381.7038	691.3556	Y	***645.3243***	323.1658	628.2977	314.6525	627.3137	314.1605	5
13	***1546.7828***	773.8950	1528.7722	764.8898	F	***482.2609***	241.6341	465.2344	233.1208	464.2504	232.6288	4
14	1633.8148	817.4111	1615.8043	808.4058	S	335.1925	168.0999	318.1660	159.5866	317.1819	159.0946	3
15	***1734.8625***	867.9349	1716.8520	858.9296	T	248.1605	124.5839	231.1339	116.0706	230.1499	115.5786	2
16					K	147.1128	74.0600	130.0863	65.5468			1

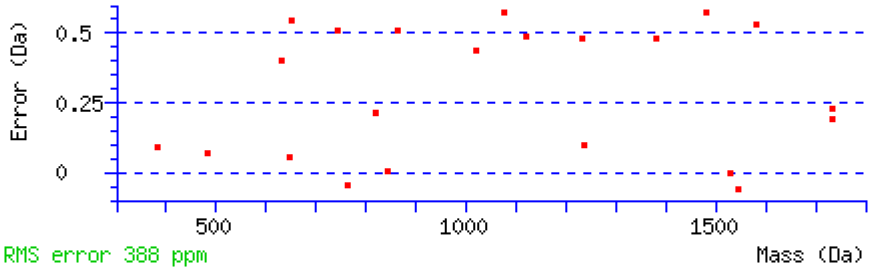

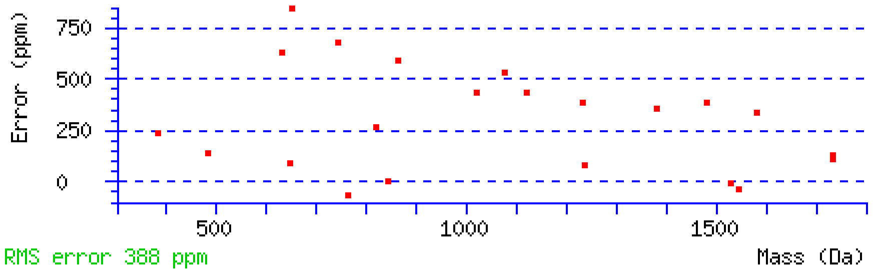

NCBI **BLAST** search of EIGTTFITPLYYFSTK
(Parameters: blastp, nr protein database, expect=20000, no filter, PAM30)
Other BLAST web gateways

All matches to this query

Score	Mr(calc)	Delta	Sequence
31.5	1879.9608	0.0011	EIGTTFITPLYYFSTK
1.8	1879.9601	0.0018	MFVSIQELVALATSQNK
1.8	1879.9601	0.0018	MFVSIQELVALATSQNK

Mascot: http://www.matrixscience.com/

MATRIX SCIENCE MASCOT Search Results

Protein View: WP_005605154.1

glutathione-disulfide reductase [Granulicatella adiacens]

**Database:**	UB_target
**Score:**	32
**Monoisotopic mass (M_r):**	48647
**Calculated pI:**	5.66

Sequence similarity is available as an NCBI BLAST search of WP_005605154.1 against nr.

Search parameters

**MS data file:**	LTQ_19B022_Kuweit_Sample-GA-EVS.mgf
**Enzyme:**	Trypsin: cuts C-term side of KR unless next residue is P.
**Fixed modifications:**	Carbamidomethyl (C)
**Variable modifications:**	Deamidated (NQ), Oxidation (M)

Protein sequence coverage: 3%

Matched peptides shown in ***bold red***.

```
  1 MKEFDLISIG GGSGGIATAN RAAMYGAKVA VVEGNLLGGT CVNIGCVPKK
 51 IMWYGAQIAE AIHAYGPDYG FTAENVKFDF KTLKKNREAY IDRSRNSYNG
101 TFERNNVTVI KGYARFVDAH TIEVNGEEYR AKHIVIATGA KPAIPNVEGK
151 ELGGTSDDVF AWDELPQSVA ILGAGYIAVE LAGVLHALGV KTDLFVRRDR
201 PLRNFDHSII EVLVAEMEKS GPTLHTNKVP QKLVQLENGS VEIQFEDGTI
251 FTAEKVIWAT GRVPHTAGLN LEAAGVELTE RGFIKVNEFQ ETTAEGVYAL
301 GDVSGEKELT PVAIKAGRTL AERLFNGQTN AKMDYTTIPT VVFSHPAIGT
351 VGLSEEQAVK EYGKENVKTY LSTFAGMYSA VTSHRQQARF KLITAGEDEK
401 VVGLHGIGYG VDEMIQGFAV AIKMGATKAD FDATVAIHPT GSEEFVTMR
```

Unformatted sequence string: 449 residues (for pasting into other applications).

Sort by	residue number	increasing mass	decreasing mass
Show	matched peptides only	predicted peptides also	

Query	Start – End	Observed	Mr(expt)	Mr(calc)	ppm	M	Score	Expect	Rank	U	Peptide

11850	116 - 130	593.6150	1777.8233	1777.8271	-2.16	0	32	0.0012	1	U	R.FVDAHTIEVNGEEYR.A

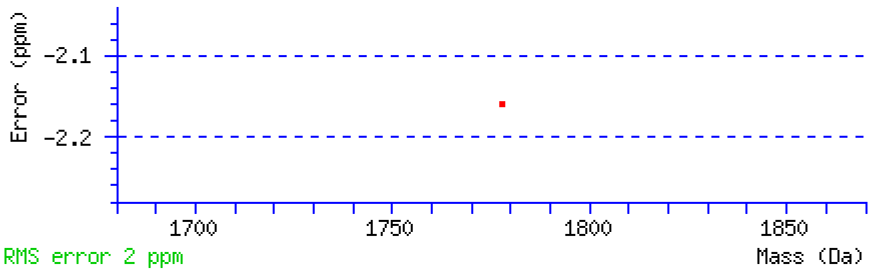

Mascot: http://www.matrixscience.com/

MATRIX SCIENCE Mascot Search Results

Peptide View

MS/MS Fragmentation of **FVDAHTIEVNGEEYR**
Found in **WP_005605154.1** in **UB_target**, glutathione-disulfide reductase [Granulicatella adiacens]

Match to Query 11850: 1777.823280 from(593.615036,3+) index(8969)
Title: Elution from: 64.725 to 64.725 period: 0 experiment: 1 cycles: 1 precIntensity: 410698.0 FinneganScanNumber: 12016 MStype: enumIsNormalMS rawFile: 19B022_Kuweit_Sample-GA-EVS.raw
Data file LTQ_19B022_Kuweit_Sample-GA-EVS.mgf

Click mouse within plot area to zoom in by factor of two about that point
Or, Plot from 100 to 2000 Da Full range
Show Y-axis

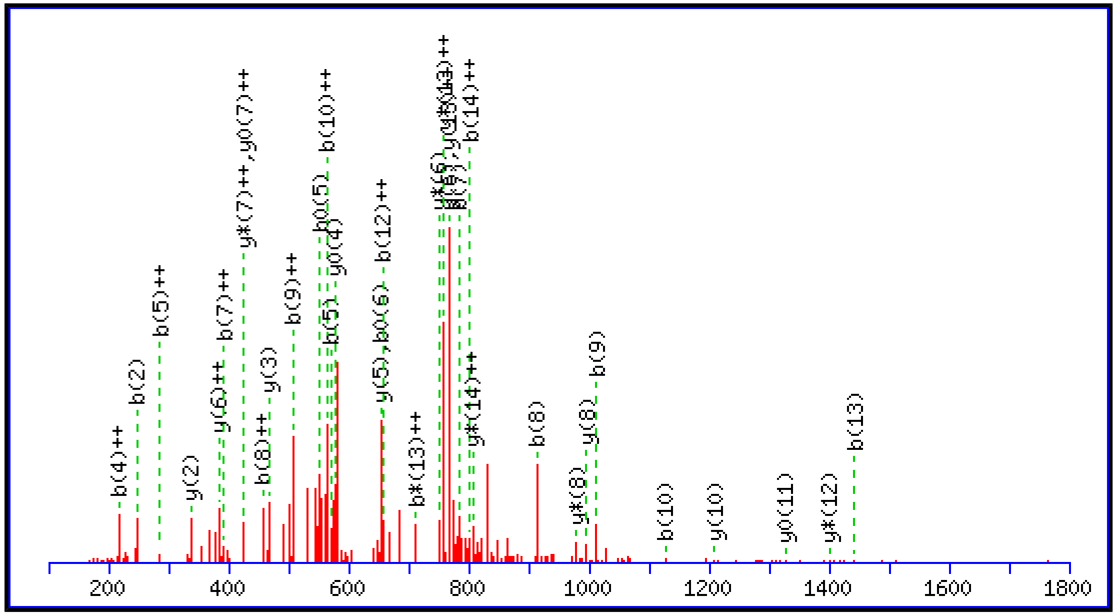

Label all possible matches   Label matches used for scoring

Monoisotopic mass of neutral peptide Mr(calc): 1777.8271
Fixed modifications: Carbamidomethyl (C) (apply to specified residues or termini only)
Ions Score: 32 **Expect:** 0.0012

Matches : 35/142 fragment ions using 74 most intense peaks (help)

#	b	b^{++}	b*	b^{*++}	b^0	b^{0++}	Seq.	y	y^{++}	y*	y^{*++}	y^0	y^{0++}	#
1	148.0757	74.5415					F							15
2	247.1441	124.0757					V	1631.7660	816.3866	1614.7394	807.8734	1613.7554	807.3814	14
3	362.1710	181.5892			344.1605	172.5839	D	1532.6976	766.8524	1515.6710	758.3392	1514.6870	757.8471	13
4	433.2082	217.1077			415.1976	208.1024	A	1417.6706	709.3390	1400.6441	700.8257	1399.6601	700.3337	12
5	570.2671	285.6372			552.2565	276.6319	H	1346.6335	673.8204	1329.6070	665.3071	1328.6230	664.8151	11
6	671.3148	336.1610			653.3042	327.1557	T	1209.5746	605.2909	1192.5481	596.7777	1191.5640	596.2857	10
7	784.3988	392.7030			766.3883	383.6978	I	1108.5269	554.7671	1091.5004	546.2538	1090.5164	545.7618	9
8	913.4414	457.2243			895.4308	448.2191	E	995.4429	498.2251	978.4163	489.7118	977.4323	489.2198	8
9	1012.5098	506.7585			994.4993	497.7533	V	866.4003	433.7038	849.3737	425.1905	848.3897	424.6985	7
10	1126.5528	563.7800	1109.5262	555.2667	1108.5422	554.7747	N	767.3319	384.1696	750.3053	375.6563	749.3213	375.1643	6
11	1183.5742	592.2907	1166.5477	583.7775	1165.5636	583.2855	G	653.2889	327.1481	636.2624	318.6348	635.2784	318.1428	5
12	1312.6168	656.8120	1295.5903	648.2988	1294.6062	647.8068	E	596.2675	298.6374	579.2409	290.1241	578.2569	289.6321	4
13	1441.6594	721.3333	1424.6329	712.8201	1423.6488	712.3281	E	467.2249	234.1161	450.1983	225.6028	449.2143	225.1108	3
14	1604.7227	802.8650	1587.6962	794.3517	1586.7122	793.8597	Y	338.1823	169.5948	321.1557	161.0815			2
15							R	175.1190	88.0631	158.0924	79.5498			1

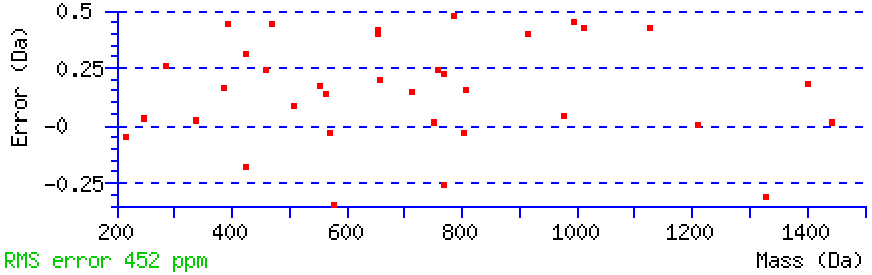

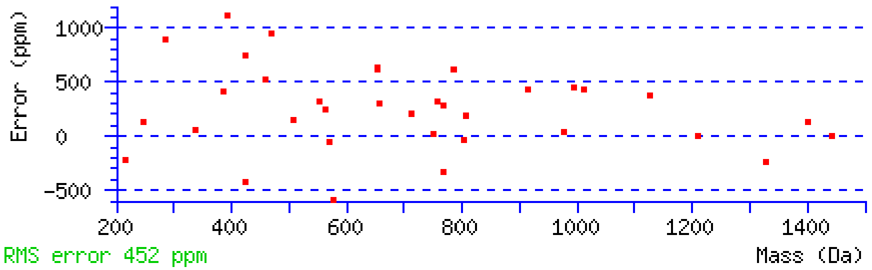

NCBI **BLAST** search of FVDAHTIEVNGEEYR
(Parameters: blastp, nr protein database, expect=20000, no filter, PAM30)
Other BLAST web gateways

All matches to this query

Score	Mr(calc)	Delta	Sequence

31.6	1777.8271	-0.0038	FVDAHTIEVNGEEYR

Mascot: http://www.matrixscience.com/

MATRIX SCIENCE MASCOT Search Results

Protein View: WP_005607237.1

phosphate acyltransferase PlsX [Granulicatella adiacens]

**Database:**	UB_target
**Score:**	30
**Monoisotopic mass (M_r):**	35756
**Calculated pI:**	5.32

Sequence similarity is available as an NCBI BLAST search of WP_005607237.1 against nr.

Search parameters

**MS data file:**	LTQ_19B022_Kuweit_Sample-GA-EVS.mgf
**Enzyme:**	Trypsin: cuts C-term side of KR unless next residue is P.
**Fixed modifications:**	Carbamidomethyl (C)
**Variable modifications:**	Deamidated (NQ), Oxidation (M)

Protein sequence coverage: 3%

Matched peptides shown in ***bold red***.

```
  1 MVRIAIDAMG GDNAPKEIVQ GVVLAAKEMP TVEFQLYGDE AKVNACLEES
 51 LPNIRVIHCS EKINSDDEPV KAIRSKKDAS MVVAAKAVKE GEADALFSCG
101 NTGALLTAGL LVVGRIKGID RPGLMPVLPV LGKDNRQFIM MDVGANAECK
151 PKNVHQFGIL GSYYSKYVLG YENPTVGLLN NGAEEGKGNE LAKEVYGLLK
201 EDDSLNFIGN VEARDILTGA ADVVVTDGFT GNAVLKTIEG TALAMMELLK
251 EGIKGQGIQG KLGALLLKNT FYGLKNTLDY SQFGGAVLFG LKGAVVKSHG
301 SSKADSVYHA MKQIDTIVSS GVINDLVAHF EQTAE
```

Unformatted sequence string: 335 residues (for pasting into other applications).

Sort by	residue number	increasing mass	decreasing mass
Show	matched peptides only	predicted peptides also	

Query	Start – End	Observed	Mr(expt)	Mr(calc)	ppm	M	Score	Expect	Rank	U	Peptide
9415	43 – 55	757.8848	1513.7550	1513.7558	-0.55	0	30	0.0032	1	U	K.VNACLEESLPNIR.V

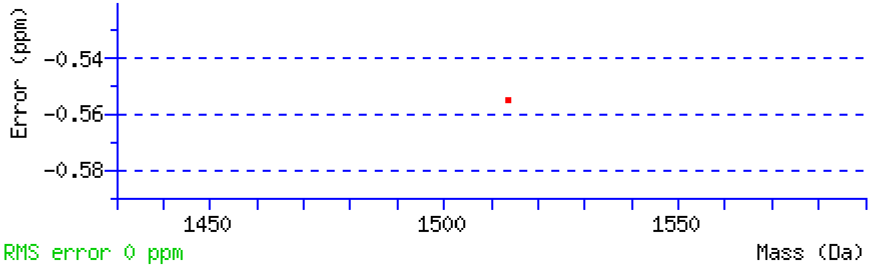

Mascot: http://www.matrixscience.com/

Mascot Search Results

Peptide View

MS/MS Fragmentation of **VNACLEESLPNIR**
Found in **WP_005607237.1** in **UB_target**, phosphate acyltransferase PlsX [Granulicatella adiacens]

Match to Query 9415: 1513.755004 from(757.884778,2+) index(14319)
Title: Elution from: 85.595 to 85.595 period: 0 experiment: 1 cycles: 1 precIntensity: 186855.0 FinneganScanNumber: 18369 MStype: enumIsNormalMS rawFile: 19B022_Kuweit_Sample-GA-EVS.raw
Data file LTQ_19B022_Kuweit_Sample-GA-EVS.mgf

Click mouse within plot area to zoom in by factor of two about that point

Or, Plot from 150 to 1100 Da Full range

Show Y-axis

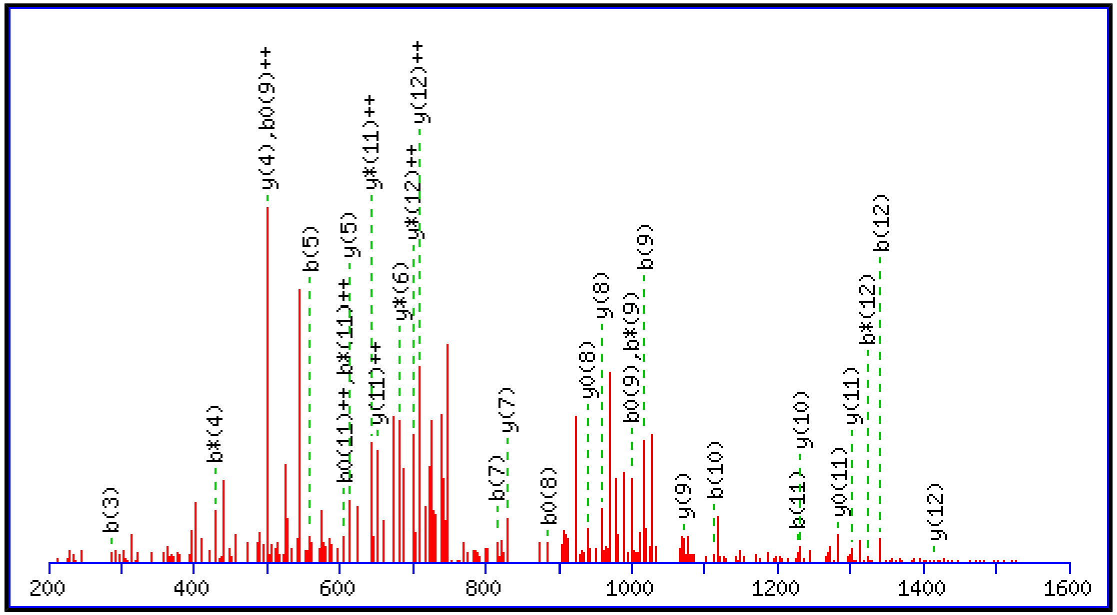

Label all possible matches Label matches used for scoring

Monoisotopic mass of neutral peptide Mr(calc): 1513.7558
Fixed modifications: Carbamidomethyl (C) (apply to specified residues or termini only)
Ions Score: 30 **Expect:** 0.0032

Matches : 30/122 fragment ions using 91 most intense peaks (help)

#	b	b^{++}	b*	b^{*++}	b^0	b^{0++}	Seq.	y	y^{++}	y*	y^{*++}	y^0	y^{0++}	#
**1**	100.0757	50.5415					**V**							**13**
**2**	214.1186	107.5629	197.0921	99.0497			**N**	***1415.6947***	708.3510	1398.6682	699.8377	1397.6842	699.3457	**12**
**3**	***285.1557***	143.0815	268.1292	134.5682			**A**	***1301.6518***	651.3295	1284.6253	642.8163	1283.6412	642.3243	**11**
**4**	445.1864	223.0968	428.1598	214.5836			**C**	***1230.6147***	615.8110	1213.5881	607.2977	1212.6041	606.8057	**10**
**5**	***558.2704***	279.6389	541.2439	271.1256			**L**	***1070.5840***	535.7957	1053.5575	527.2824	1052.5735	526.7904	**9**
**6**	687.3130	344.1602	670.2865	335.6469	669.3025	335.1549	**E**	***957.5000***	479.2536	940.4734	470.7404	939.4894	470.2483	**8**
**7**	***816.3556***	408.6815	799.3291	400.1682	798.3451	399.6762	**E**	***828.4574***	414.7323	811.4308	406.2191	810.4468	405.7271	**7**
**8**	903.3877	452.1975	886.3611	443.6842	885.3771	443.1922	**S**	699.4148	350.2110	682.3882	341.6978	681.4042	341.2058	**6**
**9**	***1016.4717***	508.7395	999.4452	500.2262	998.4612	499.7342	**L**	***612.3828***	306.6950	595.3562	298.1817			**5**
**10**	***1113.5245***	557.2659	1096.4979	548.7526	1095.5139	548.2606	**P**	***499.2987***	250.1530	482.2722	241.6397			**4**
**11**	***1227.5674***	614.2873	1210.5409	605.7741	1209.5568	605.2821	**N**	402.2459	201.6266	385.2194	193.1133			**3**
**12**	***1340.6515***	670.8294	1323.6249	662.3161	1322.6409	661.8241	**I**	288.2030	144.6051	271.1765	136.0919			**2**
**13**							**R**	175.1190	88.0631	158.0924	79.5498			**1**

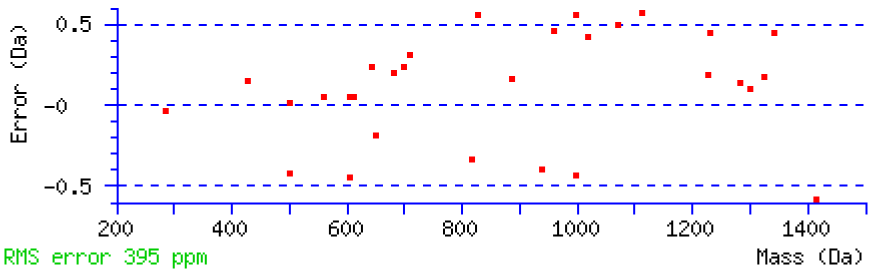

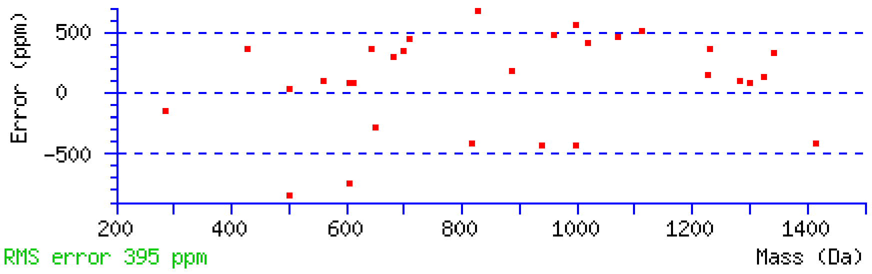

NCBI **BLAST** search of VNACLEESLPNIR
(Parameters: blastp, nr protein database, expect=20000, no filter, PAM30)
Other BLAST web gateways

All matches to this query

Score	Mr(calc)	Delta	Sequence
30.4	1513.7558	-0.0008	VNACLEESLPNIR
1.1	1513.7559	-0.0009	TSFGMQVVGSTTKR

Mascot: http://www.matrixscience.com/

MASCOT Search Results

Protein View: RKW29474.1

F0F1 ATP synthase subunit alpha [Granulicatella sp.]

**Database:**	UB_target
**Score:**	30
**Monoisotopic mass (M_r):**	55089
**Calculated pI:**	5.45

Sequence similarity is available as an NCBI BLAST search of RKW29474.1 against nr.

Search parameters

**MS data file:**	LTQ_19B022_Kuweit_Sample-GA-EVS.mgf
**Enzyme:**	Trypsin: cuts C-term side of KR unless next residue is P.
**Fixed modifications:**	Carbamidomethyl (C)
**Variable modifications:**	Deamidated (NQ), Oxidation (M)

Protein sequence coverage: 2%

Matched peptides shown in ***bold red***.

```
  1 MDTEFGNITA GIRNQINSFE AEQHIEEIGE VSFIGDGIAR VIGLTNVMAG
 51 ELVEFANGTY GMAQNLEKND VGVIIFGTYE NIHEGESVRR TGRILDVPVG
101 DALIGRVVDA LGRPIDGLGA LETTKRRPVE NEAPGVMQRK SVHQSLATGL
151 KVIDALVPIG KGQRELIIGD RKTGKTSIAI DAILNQKGKD TLCIYVAIGQ
201 KESTVKALVE TLKRYGAMDY TTVVSASASQ PAPMLYIAPY AGTAMGEEFM
251 YNGKDVLIVY DDLSKQAAAY REISLLLRRP PGREAYPGDV FYLHSRLLER
301 SAKLNDELGG GSLTALPIIE TQAGDISAYI PTNVISITDG QIFLESDLFY
351 SGVRPGLSAG LSVSRVGGSA QIKAMKKVSG TLRIDLASYR ELEAFTQFGS
401 DLDAATQQKL NRGKRTVEVL KQDVHRPLPI EHQVSILFAL THGVLDSIPI
451 ERLKDFEKAL YHHLEHEHAD ILASIRDEEK IPDEEAFYQV IDKFKQIHLY
501 ER
```

Unformatted sequence string: 502 residues (for pasting into other applications).

Sort by residue number increasing mass decreasing mass

Show matched peptides only predicted peptides also

Query	Start – End	Observed	Mr(expt)	Mr(calc)	ppm	M	Score	Expect	Rank	U	Peptide
8085	94 – 106	669.3914	1336.7682	1336.7715	-2.43	0	30	0.0011	1	U	R.ILDVPVGDALIGR.V

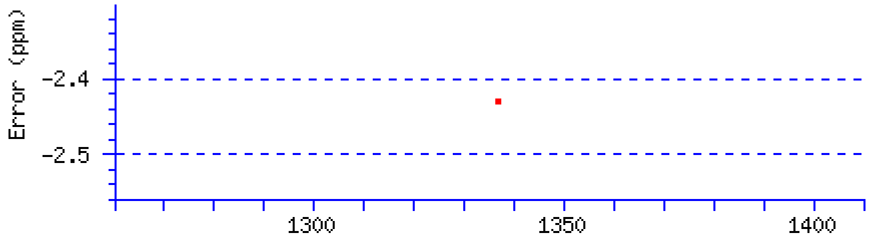

RMS error 2 ppm

Mascot: http://www.matrixscience.com/

MATRIX SCIENCE Mascot Search Results

Peptide View

MS/MS Fragmentation of **ILDVPVGDALIGR**
Found in **RKW29474.1** in **UB_target**, F0F1 ATP synthase subunit alpha [Granulicatella sp.]

Match to Query 8085: 1336.768204 from(669.391378,2+) index(18754)
Title: Elution from: 102.370 to 102.370 period: 0 experiment: 1 cycles: 1 precIntensity: 122875.0 FinneganScanNumber: 23597 MStype: enumIsNormalMS rawFile: 19B022_Kuweit_Sample-GA-EVS.raw
Data file LTQ_19B022_Kuweit_Sample-GA-EVS.mgf

Click mouse within plot area to zoom in by factor of two about that point
Or, Plot from 100 to 1400 Da Full range
Show Y-axis ☐

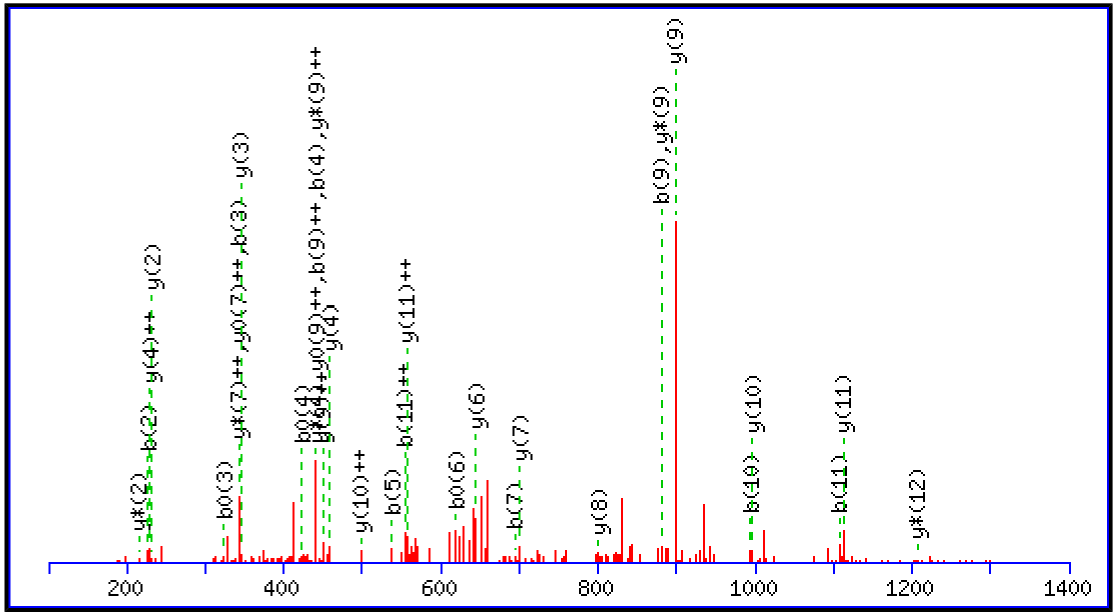

Label all possible matches Label matches used for scoring

Monoisotopic mass of neutral peptide Mr(calc): 1336.7715
Fixed modifications: Carbamidomethyl (C) (apply to specified residues or termini only)
Ions Score: 30 **Expect:** 0.0011

Matches : 34/106 fragment ions using 90 most intense peaks (help)

#	b	b^{++}	b^{0}	b^{0++}	Seq.	y	y^{++}	y*	y^{*++}	y^{0}	y^{0++}	#
1	114.0913	57.5493			I							13
2	**227.1754**	114.0913			L	1224.6947	612.8510	1207.6681	604.3377	1206.6841	603.8457	12
3	**342.2023**	171.6048	324.1918	162.5995	D	***1111.6106***	**556.3089**	1094.5841	547.7957	1093.6000	547.3037	11
4	**441.2708**	221.1390	423.2602	212.1337	V	***996.5837***	**498.7955**	979.5571	490.2822	978.5731	489.7902	10
5	**538.3235**	269.6654	520.3130	260.6601	P	***897.5152***	**449.2613**	880.4887	440.7480	879.5047	440.2560	9
6	637.3919	319.1996	619.3814	310.1943	V	***800.4625***	400.7349	783.4359	392.2216	782.4519	391.7296	8
7	**694.4134**	347.7103	676.4028	338.7051	G	***701.3941***	351.2007	684.3675	342.6874	683.3835	342.1954	7
8	809.4403	405.2238	791.4298	396.2185	D	***644.3726***	322.6899	627.3461	314.1767	626.3620	313.6847	6
9	**880.4775**	440.7424	862.4669	431.7371	A	529.3457	265.1765	512.3191	256.6632			5
10	**993.5615**	497.2844	975.5510	488.2791	L	***458.3085***	**229.6579**	441.2820	221.1446			4
11	**1106.6456**	553.8264	1088.6350	544.8211	I	***345.2245***	173.1159	328.1979	164.6026			3
12	1163.6671	582.3372	1145.6565	573.3319	G	***232.1404***	116.5738	215.1139	108.0606			2
13					R	175.1190	88.0631	158.0924	79.5498			1

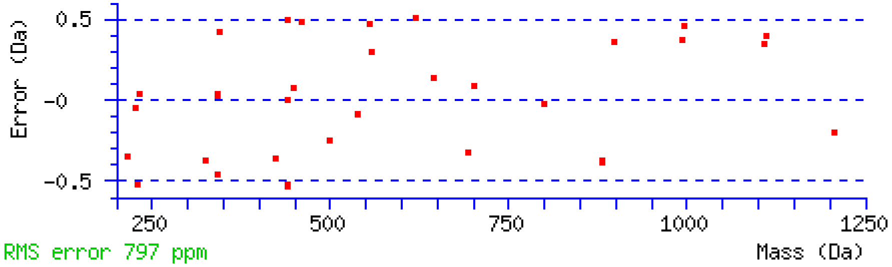

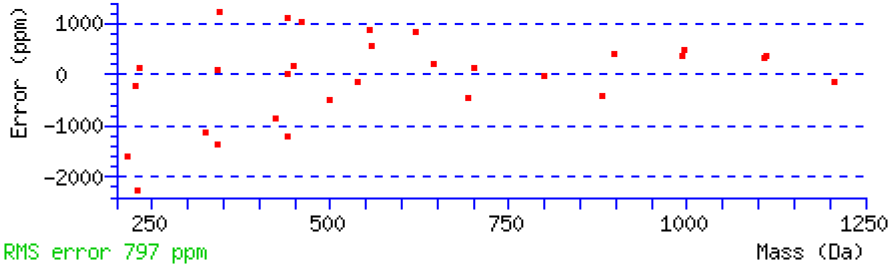

NCBI **BLAST** search of ILDVPVGDALIGR
(Parameters: blastp, nr protein database, expect=20000, no filter, PAM30)
Other BLAST web gateways

All matches to this query

Score	Mr(calc)	Delta	Sequence
29.5	1336.7715	-0.0032	ILDVPVGDALIGR

Mascot: http://www.matrixscience.com/

MATRIX SCIENCE MASCOT Search Results

Protein View: WP_005607463.1

50S ribosomal protein L24 [Granulicatella adiacens]

**Database:**	UB_target
**Score:**	28
**Monoisotopic mass (M_r):**	10851
**Calculated pI:**	10.15

Sequence similarity is available as an NCBI BLAST search of WP_005607463.1 against nr.

Search parameters

**MS data file:**	LTQ_19B022_Kuweit_Sample-GA-EVS.mgf
**Enzyme:**	Trypsin: cuts C-term side of KR unless next residue is P.
**Fixed modifications:**	Carbamidomethyl (C)
**Variable modifications:**	Deamidated (NQ), Oxidation (M)

Protein sequence coverage: 10%

Matched peptides shown in ***bold red***.

```
 1 MHVKTGDIVK VISGKDKGKE GKILKSFPKK DRVIVEGVNI VKKHQKPSQA
51 NQTGGIVEVE APIHVSNVMF VDPTTGKASR TGFKVENGEK VRVPKGRNKA
```

(Matched: residues 33–42, VIVEGVNI VK)

Unformatted sequence string: 100 residues (for pasting into other applications).

Sort by residue number increasing mass decreasing mass
Show matched peptides only predicted peptides also

Query	Start – End	Observed	Mr(expt)	Mr(calc)	ppm	M	Score	Expect	Rank	U	Peptide
5402	33 – 42	535.3340	1068.6534	1068.6543	-0.82	0	28	0.0029	1	U	R.VIVEGVNIVK.K

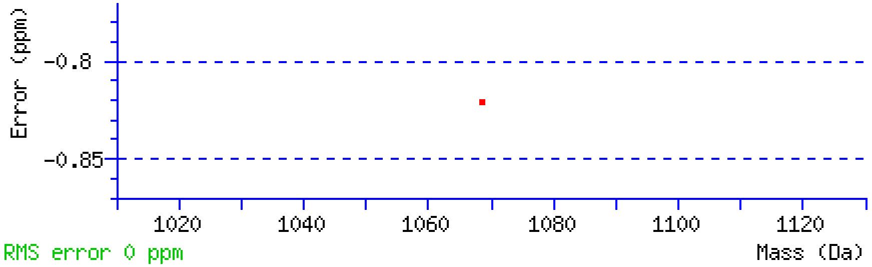

Mascot: http://www.matrixscience.com/

Mascot Search Results

Peptide View

MS/MS Fragmentation of **VIVEGVNIVK**
Found in **WP_005607463.1** in **UB_target**, 50S ribosomal protein L24 [Granulicatella adiacens]

Match to Query 5402: 1068.653420 from(535.333986,2+) index(11825)
Title: Elution from: 75.840 to 75.840 period: 0 experiment: 1 cycles: 1 precIntensity: 199905.0 FinneganScanNumber: 15404 MStype: enumIsNormalMS rawFile: 19B022_Kuweit_Sample-GA-EVS.raw
Data file LTQ_19B022_Kuweit_Sample-GA-EVS.mgf

Click mouse within plot area to zoom in by factor of two about that point
Or, Plot from 150 to 1100 Da Full range
Show Y-axis

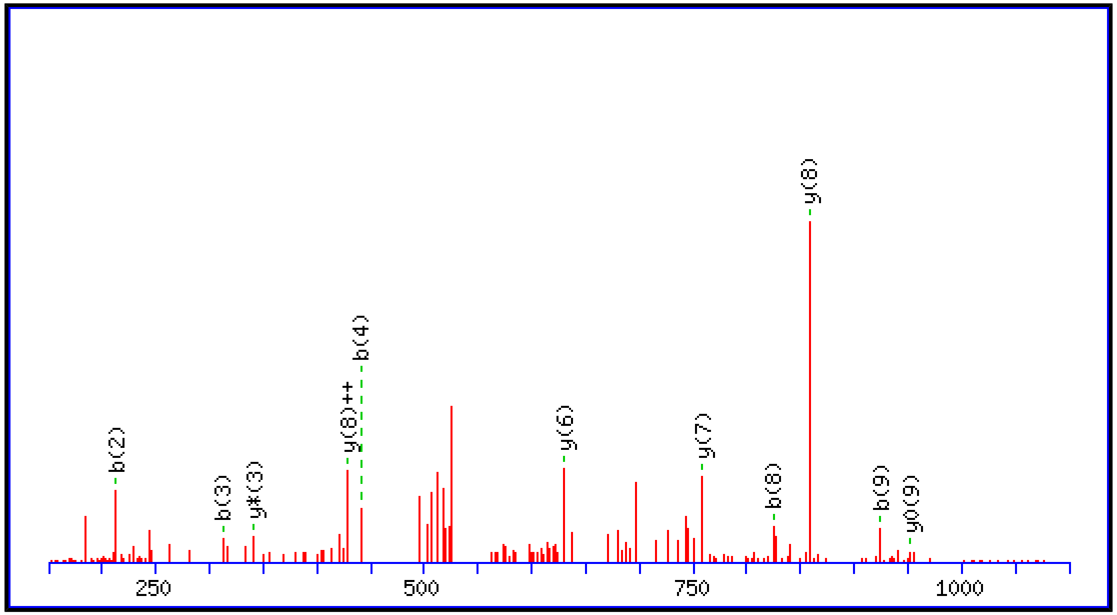

Label all possible matches Label matches used for scoring

Monoisotopic mass of neutral peptide Mr(calc): 1068.6543
Fixed modifications: Carbamidomethyl (C) (apply to specified residues or termini only)
Ions Score: 28 **Expect:** 0.0029

Matches : 11/78 fragment ions using 20 most intense peaks (help)

#	b	b^{++}	b*	b^{*++}	b^0	b^{0++}	Seq.	y	y^{++}	y*	y^{*++}	y^0	y^{0++}	#
1	100.0757	50.5415					V							10
2	213.1598	107.0835					I	970.5932	485.8002	953.5666	477.2869	952.5826	476.7949	9
3	312.2282	156.6177					V	857.5091	429.2582	840.4825	420.7449	839.4985	420.2529	8
4	441.2708	221.1390			423.2602	212.1337	E	758.4407	379.7240	741.4141	371.2107	740.4301	370.7187	7
5	498.2922	249.6498			480.2817	240.6445	G	629.3981	315.2027	612.3715	306.6894			6
6	597.3606	299.1840			579.3501	290.1787	V	572.3766	286.6919	555.3501	278.1787			5
7	711.4036	356.2054	694.3770	347.6921	693.3930	347.2001	N	473.3082	237.1577	456.2817	228.6445			4
8	824.4876	412.7475	807.4611	404.2342	806.4771	403.7422	I	359.2653	180.1363	342.2387	171.6230			3
9	923.5560	462.2817	906.5295	453.7684	905.5455	453.2764	V	246.1812	123.5942	229.1547	115.0810			2
10							K	147.1128	74.0600	130.0863	65.5468			1

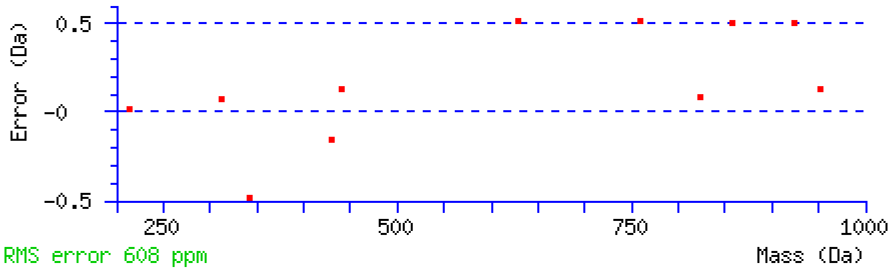

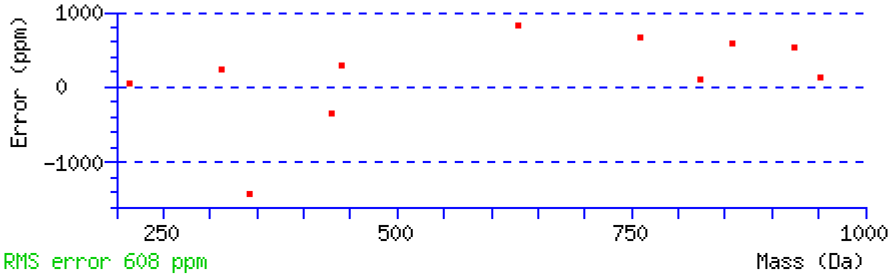

NCBI **BLAST** search of VIVEGVNIVK
(Parameters: blastp, nr protein database, expect=20000, no filter, PAM30)
Other BLAST web gateways

All matches to this query

Score	Mr(calc)	Delta	Sequence
28.0	1068.6543	-0.0009	VIVEGVNIVK
28.0	1068.6543	-0.0009	VLVEGVNIVK

Mascot: http://www.matrixscience.com/

MATRIX SCIENCE MASCOT Search Results

Protein View: WP_005607461.1

50S ribosomal protein L5 [Granulicatella adiacens]

**Database:**	UB_target
**Score:**	26
**Monoisotopic mass (M_r):**	20147
**Calculated pI:**	9.42

Sequence similarity is available as an NCBI BLAST search of WP_005607461.1 against nr.

Search parameters

**MS data file:**	LTQ_19B022_Kuweit_Sample-GA-EVS.mgf
**Enzyme:**	Trypsin: cuts C-term side of KR unless next residue is P.
**Fixed modifications:**	Carbamidomethyl (C)
**Variable modifications:**	Deamidated (NQ), Oxidation (M)

Protein sequence coverage: 10%

Matched peptides shown in ***bold red***.

```
  1 MNRLNAKYKN EVVPSLVEKF NYKSIMEVPK VEKIVINMGV GDATSNAKNL
 51 EKAVEELTLI SGQKPVVTTA KKSIAGFRLR EGMPIGTKVT LRGERMYDFL
101 DKLVTVSLPR VRDFRGISKK SFDGRGNYTL GVKEQLIFPE IDYDRVDKVR
151 GMDIVIVTTA NTDEEAKELL TQLGMPFQK
```

Unformatted sequence string: 179 residues (for pasting into other applications).

Sort by	residue number	increasing mass	decreasing mass
Show	matched peptides only	predicted peptides also	

Query	Start – End	Observed	Mr(expt)	Mr(calc)	ppm	M	Score	Expect	Rank	U	Peptide
13909	53 – 71	662.0495	1983.1267	1983.1252	0.78	0	26	0.0023	1	U	K.AVEELTLISGQKPVVTTAK.K

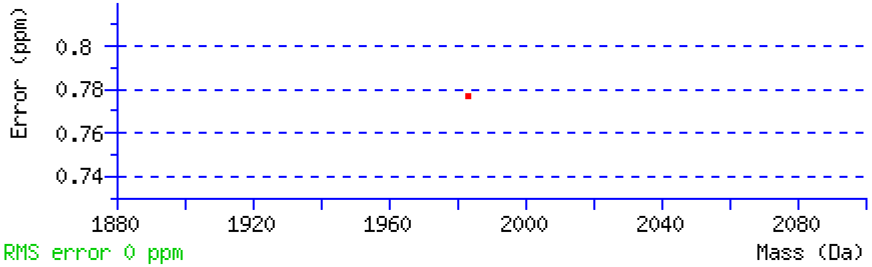

Mascot: http://www.matrixscience.com/

Mascot Search Results

Peptide View

MS/MS Fragmentation of **AVEELTLISGQKPVVTTAK**
Found in **WP_005607461.1** in **UB_target**, 50S ribosomal protein L5 [Granulicatella adiacens]

Match to Query 13909: 1983.126738 from(662.049522,3+) index(13774)
Title: Elution from: 83.418 to 83.418 period: 0 experiment: 1 cycles: 1 precIntensity: 305572.0 FinneganScanNumber: 17712 MStype: enumIsNormalMS rawFile: 19B022_Kuweit_Sample-GA-EVS.raw
Data file LTQ_19B022_Kuweit_Sample-GA-EVS.mgf

Click mouse within plot area to zoom in by factor of two about that point
Or, Plot from 100 to 2000 Da Full range
Show Y-axis

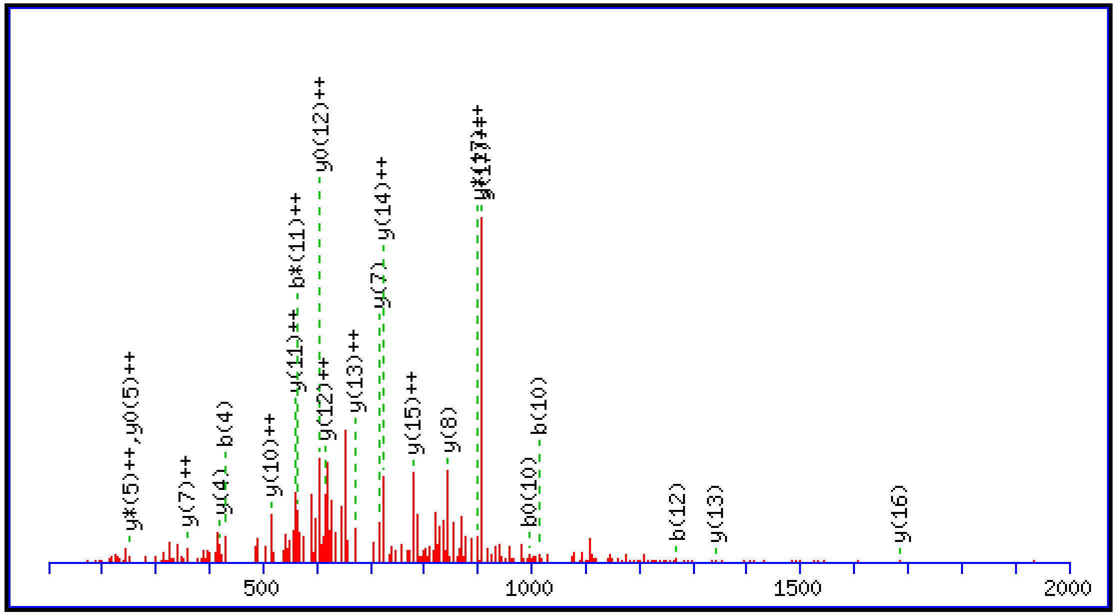

Label all possible matches Label matches used for scoring

Monoisotopic mass of neutral peptide Mr(calc): 1983.1252
Fixed modifications: Carbamidomethyl (C) (apply to specified residues or termini only)
Ions Score: 26 **Expect:** 0.0023

Matches : 22/188 fragment ions using 59 most intense peaks (help)

#	b	b^{++}	b^{*}	b^{*++}	b^{0}	b^{0++}	Seq.	y	y^{++}	y^{*}	y^{*++}	y^{0}	y^{0++}	#
1	72.0444	36.5258					A							19
2	171.1128	86.0600					V	1913.0954	957.0513	1896.0688	948.5380	1895.0848	948.0460	18
3	300.1554	150.5813			282.1448	141.5761	E	1814.0270	***907.5171***	1797.0004	899.0038	1796.0164	898.5118	17
4	**429.1980**	215.1026			411.1874	206.0974	E	***1684.9844***	842.9958	1667.9578	834.4825	1666.9738	833.9905	16
5	542.2821	271.6447			524.2715	262.6394	L	1555.9418	***778.4745***	1538.9152	769.9612	1537.9312	769.4692	15
6	643.3297	322.1685			625.3192	313.1632	T	1442.8577	***721.9325***	1425.8312	713.4192	1424.8471	712.9272	14
7	756.4138	378.7105			738.4032	369.7053	L	***1341.8100***	***671.4087***	1324.7835	662.8954	1323.7995	662.4034	13
8	869.4979	435.2526			851.4873	426.2473	I	1228.7260	***614.8666***	1211.6994	606.3533	1210.7154	605.8613	12
9	956.5299	478.7686			938.5193	469.7633	S	1115.6419	***558.3246***	1098.6154	549.8113	1097.6313	549.3193	11
10	**1013.5514**	507.2793			995.5408	498.2740	G	1028.6099	***514.8086***	1011.5833	506.2953	1010.5993	505.8033	10
11	1141.6099	571.3086	1124.5834	562.7953	1123.5994	562.3033	Q	971.5884	486.2978	954.5619	477.7846	953.5778	477.2926	9
12	**1269.7049**	635.3561	1252.6783	626.8428	1251.6943	626.3508	K	***843.5298***	422.2686	826.5033	413.7553	825.5193	413.2633	8
13	1366.7577	683.8825	1349.7311	675.3692	1348.7471	674.8772	P	***715.4349***	***358.2211***	698.4083	349.7078	697.4243	349.2158	7
14	1465.8261	733.4167	1448.7995	724.9034	1447.8155	724.4114	V	618.3821	309.6947	601.3556	301.1814	600.3715	300.6894	6
15	1564.8945	782.9509	1547.8679	774.4376	1546.8839	773.9456	V	519.3137	260.1605	502.2871	251.6472	501.3031	251.1552	5
16	1665.9422	833.4747	1648.9156	824.9614	1647.9316	824.4694	T	***420.2453***	210.6263	403.2187	202.1130	402.2347	201.6210	4
17	1766.9898	883.9986	1749.9633	875.4853	1748.9793	874.9933	T	319.1976	160.1024	302.1710	151.5892	301.1870	151.0972	3
18	1838.0270	919.5171	1821.0004	911.0038	1820.0164	910.5118	A	218.1499	109.5786	201.1234	101.0653			2
19							K	147.1128	74.0600	130.0863	65.5468			1

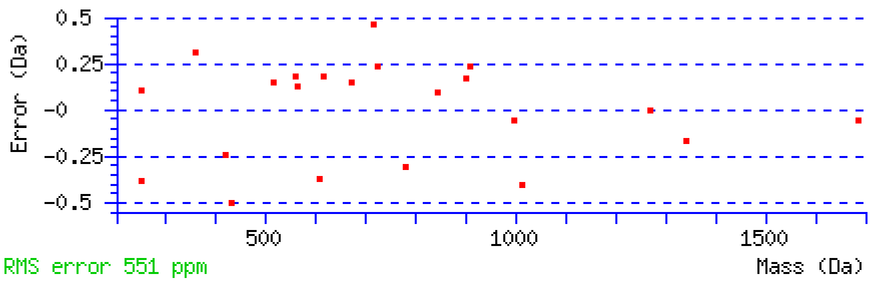

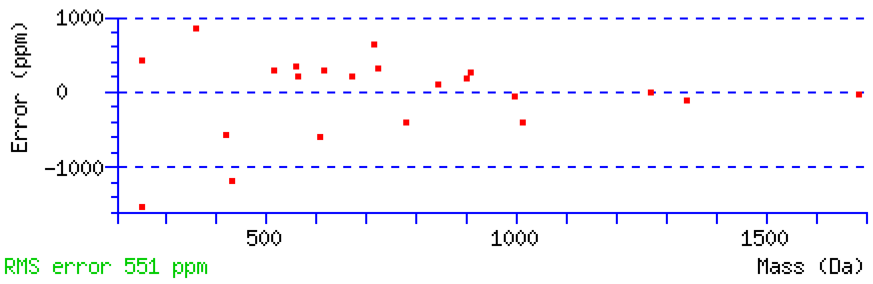

NCBI **BLAST** search of AVEELTLISGQKPVVTTAK
(Parameters: blastp, nr protein database, expect=20000, no filter, PAM30)

Other BLAST web gateways

All matches to this query

Score	Mr(calc)	Delta	Sequence
26.5	1983.1252	0.0015	AVEELTLISGQKPVVTTAK

Mascot: http://www.matrixscience.com/
