# Supplementary material for: Proteomics of extracellular vesicles produced by Granulicatella adiacens, which causes infective endocarditis
Source: PLoS One. 2020 Nov 20;15(11):e0227657. doi: 10.1371/journal.pone.0227657 (PMC7679012; doi:10.1371/journal.pone.0227657)
Supplement: S1 Raw images — (PDF) [file pone.0227657.s004.pdf]

Original gel images used in Figure 2A in the revised manuscript

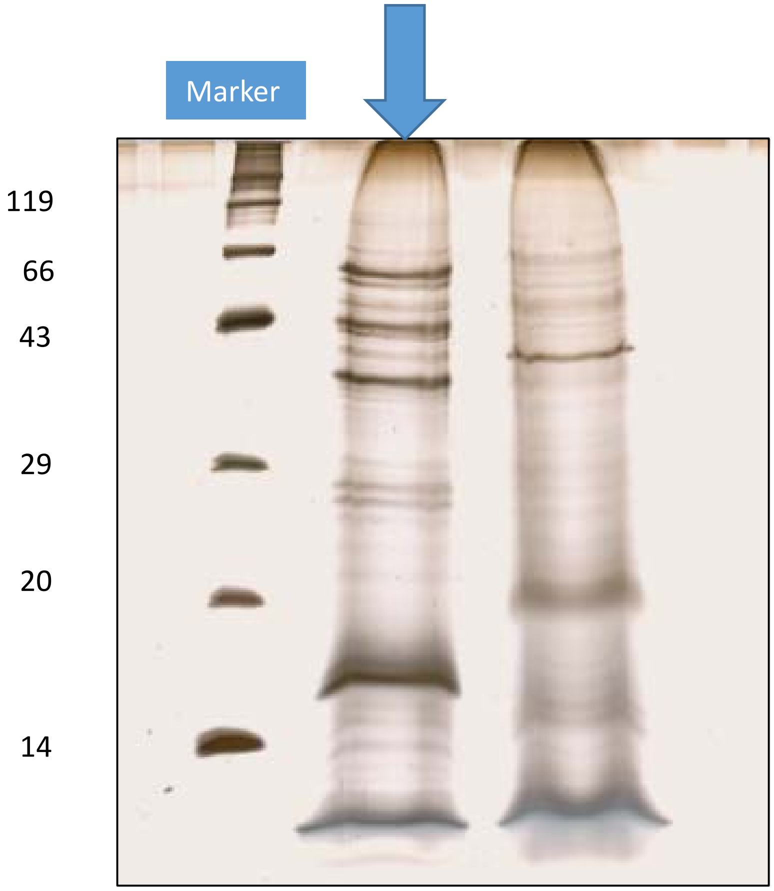

The lane with the arrow is the lane “EVs” presented in Figure 2A in the revised manuscript.

This is the original gel image we obtained from the proteomics facility in Germany. The gel was run before running the EVs samples on LC/MS/MS.

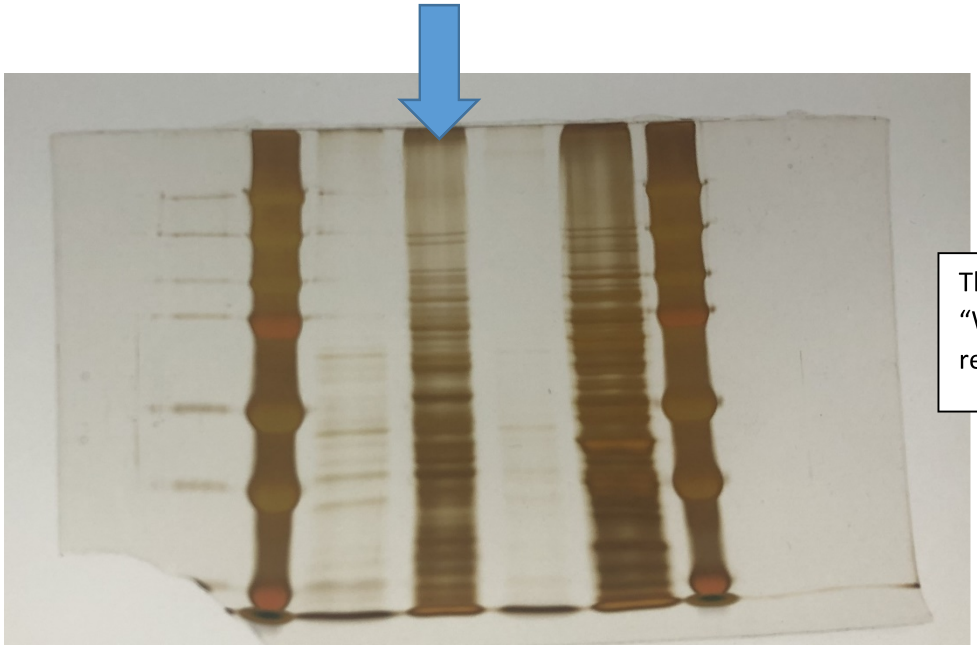

The lane with the arrow is the lane “WCP” presented in Figure 2A in the revised manuscript.
